# A Dinuclear Ruthenium-Based Water Oxidation Catalyst: Use of Non-Innocent Ligand Frameworks for Promoting Multi-Electron Reactions

**DOI:** 10.1002/chem.201406613

**Published:** 2015-04-29

**Authors:** Tanja M Laine, Markus D Kärkäs, Rong-Zhen Liao, Per E M Siegbahn, Björn Åkermark

**Affiliations:** [a] Department of Organic Chemistry, Arrhenius LaboratoryStockholm University, 106 91 Stockholm (Sweden); [b] Key Laboratory for Large-Format Battery Materials and SystemMinistry of Education, School of Chemistry and Chemical Engineering, Huazhong University of Science and Technology, Wuhan 430074 (P.R. China)

**Keywords:** density functional calculations, electrochemistry, ligands, ruthenium, water splitting

## Abstract

Insight into how H_2_O is oxidized to O_2_ is envisioned to facilitate the rational design of artificial water oxidation catalysts, which is a vital component in solar-to-fuel conversion schemes. Herein, we report on the mechanistic features associated with a dinuclear Ru-based water oxidation catalyst. The catalytic action of the designed Ru complex was studied by the combined use of high-resolution mass spectrometry, electrochemistry, and quantum chemical calculations. Based on the obtained results, it is suggested that the designed ligand scaffold in Ru complex **1** has a non-innocent behavior, in which metal–ligand cooperation is an important part during the four-electron oxidation of H_2_O. This feature is vital for the observed catalytic efficiency and highlights that the preparation of catalysts housing non-innocent molecular frameworks could be a general strategy for accessing efficient catalysts for activation of H_2_O.

## Introduction

The production of solar fuels, such as H_2_, by the splitting of H_2_O constitutes an appealing strategy for attaining sustainable systems for energy conversion. In such systems, the catalytic conversion of H_2_O to O_2_ [Eq. (1)], which will generate the necessary electrons and protons, is considered to be the essential step.[1–4] Due to the thermodynamic and catalytic complexity connected with the four-electron oxidation of H_2_O, the development of artificial water oxidation catalysts (WOCs) has become an important research field.[5]

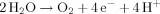


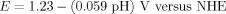
(1)

**Figure 1:**
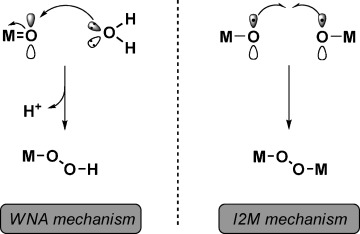
Depiction of the different mechanistic pathways by which H_2_O is oxidized. Left: Water nucleophilic attack (WNA) pathway; Right: Interaction of two M=O units (I2M mechanism) pathway.

Although there has been considerable progress during the last couple of years with the construction of molecular catalysts based on Ru,[6,7] Ir,[8] Mn,[9] Fe,[10] Co,[11] and Cu,[12] the mechanistic description associated with such WOCs is usually not well defined. It is therefore important to understand how H_2_O is oxidized by artificial WOCs. This is an intricate scientific goal, which, if accomplished, is expected to facilitate the design of more-efficient catalysts. Through a number of experimental[13] and theoretical[14] studies, impressive progress has been made in unravelling the different molecular mechanisms by which H_2_O is activated by artificial WOCs. It has thus been established that H_2_O oxidation and O=O bond formation mainly take place through two different pathways: 1) Water nucleophilic attack on a high-valent metal-oxo unit (WNA mechanism; Figure 1, left), and 2) the interaction of two M=O units (I2M mechanism; Figure 1, right), which can occur either intermolecularly between two catalyst entities or intramolecularly for dinuclear catalysts.[15] A third pathway has recently been suggested for a Mn-based WOC, in which O=O bond formation occurs by coupling a Mn^IV^-bound terminal oxyl radical and a di-Mn bridging oxo group within a tetranuclear Mn species.[16]

It is essential that the catalysts are able of reaching high redox states within a relatively narrow potential window. For this to occur efficiently, it is thus important that the designed catalysts undergo concerted removal of protons and electrons to avoid Coulombic charge build-up. The coordinated movement of protons and electrons through proton-coupled electron transfer (PCET) ensures pathways with low-energy profiles, which is particularly beneficial for a multi-electron reaction such as H_2_O oxidation.[17,18]

**Figure 2:**
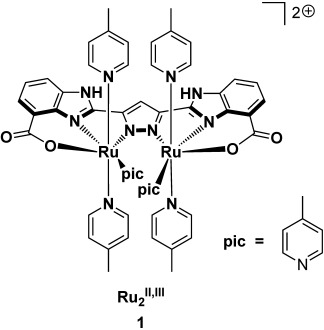
Structure of the dinuclear Ru_2_^II,III^ complex 1.

We have recently developed the dinuclear Ru_2_^II,III^ complex **1** (Figure 2), which efficiently catalyzes the photochemical conversion of H_2_O into O_2_.[19] The designed electron-rich anionic pyrazole-based ligand scaffold (H_5_L) was found to afford a suitable environment for the Ru centers by placing them in close proximity (4.93 Å, see the Supporting Information, Figure S6), to give a complex with favorable low redox potentials, thus allowing access to multiple redox states. Based on these attractive catalytic features it was decided to study this catalytic system in more detail by combining experimental measurements and quantum chemical calculations.

In this work, we further explore the mechanistic details associated with the dinuclear Ru complex **1** by using a combination of experimental measurements and quantum chemical calculations. In addition to stabilizing the metal centers in high-valent redox states, it was revealed that the constructed ligand motif facilitates proton movement and actively participates during the four-electron oxidation of H_2_O. These features collectively account for the observed catalytic efficiency of Ru complex **1** and highlight the concept of using non-innocent ligand scaffolds for carrying out multi-electron reactions, such as oxidation of H_2_O.

## Results and Discussion

### High-resolution mass spectrometry analysis of intermediates involved in H_2_O oxidation

**Figure 3:**
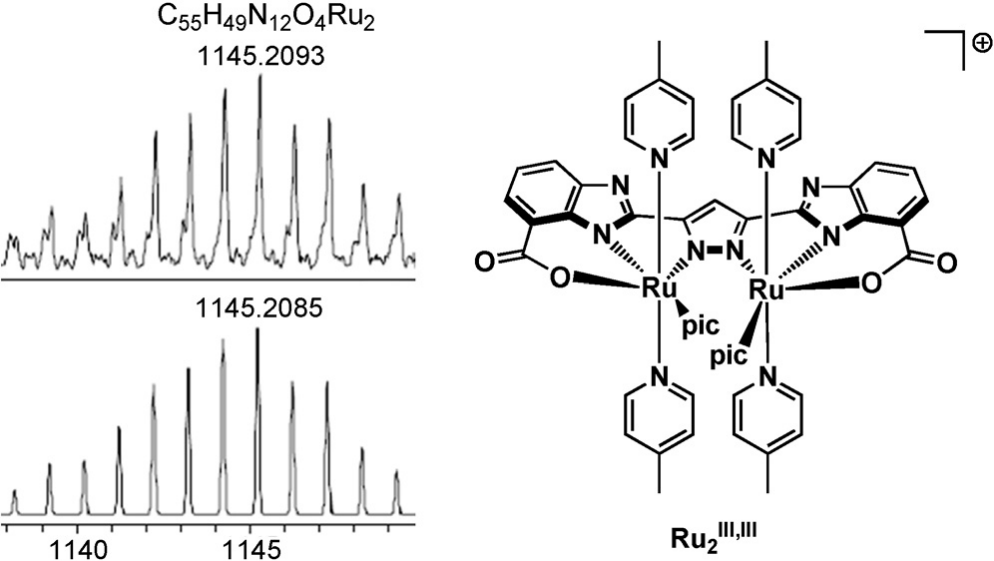
Top: High-resolution mass spectrum of the [(L)Ru_2_^III,III^(pic)_6_]^+^ species {[(HL)Ru_2_^III,III^(pic)_6_]^2+^−H^+^} recorded in positive mode. Bottom: Simulated spectrum. pic=4-picoline.

High-resolution mass spectrometry (HRMS) has previously been employed as a tool for studying and detecting intermediates during H_2_O oxidation catalysis.[20,21] HRMS was therefore initially assessed as a technique for investigating the different intermediates involved in the catalytic system for Ru complex **1**. An aqueous solution containing the dinuclear Ru complex **1** was analyzed in positive mode. This resulted in the observation of a peak at *m*/*z* 1146.2155, which was assigned to the starting complex **1**, {[(H_2_L)Ru_2_^II,III^(pic)_6_]^2+^−H^+^}^+^.[19] The one-electron oxidized species of complex **1**, the corresponding Ru_2_^III,III^ complex, was also possible to detect at *m*/*z* 1145.2093 (Figure 3 and [Eq. (2)]). In accordance with the Pourbaix diagram (see below) the reaction is proton-coupled at pH>3 and the product from this oxidation (at pH 7.2) thus results in [(HL)Ru_2_^III,III^(pic)_6_]^2+^.

**Figure 4:**
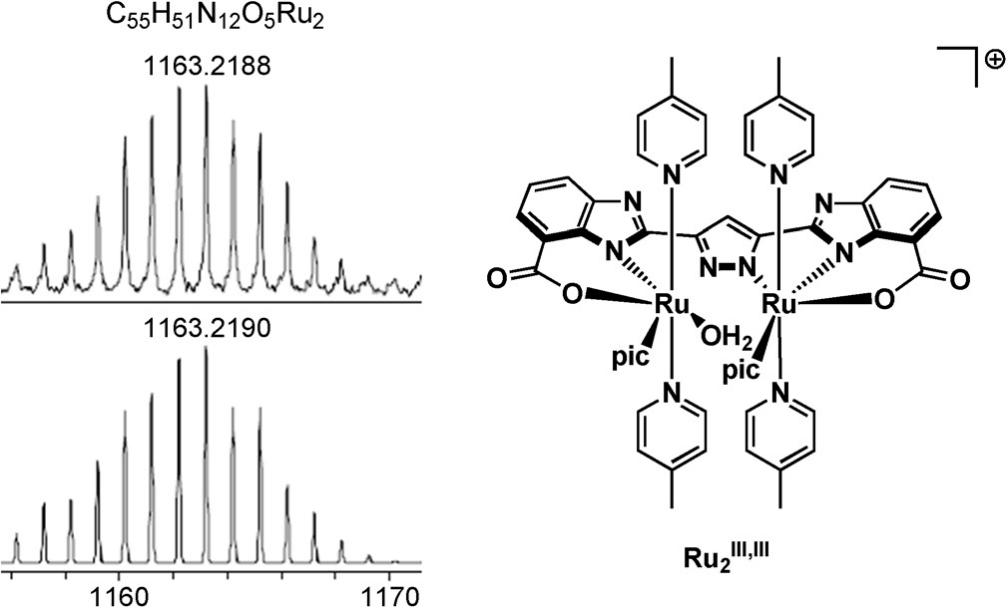
Top: High-resolution mass spectrum of the formal [(L)Ru_2_^III,III^(pic)_6_(OH_2_)]^+^ species {[(HL)Ru_2_^III,III^(pic)_6_(OH_2_)]^2+^−H^+^} recorded in positive mode. Bottom: Simulated spectrum.

Access to the corresponding Ru-aqua complexes is essential in H_2_O oxidation catalysis since it permits PCET redox events to occur, allowing high-valent redox states to be generated. Allowing the Ru_2_^III,III^ oxidized species to stand for a short period of time in aqueous solutions resulted in the formation of the formal Ru_2_^III,III^ aqua species [(HL)Ru_2_^III,III^(pic)_6_(OH_2_)]^2+^ [Eq. (3)] shown in Figure 4. This is in accordance with quantum chemical calculations (see below, see also Figure 8), which show that the oxidized Ru complex **1** and the aqua species have comparable stabilities, Figure S47 (the Supporting Information).

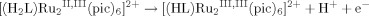
(2)

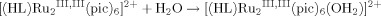
(3)

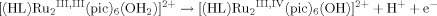
(4)

**Figure 5:**
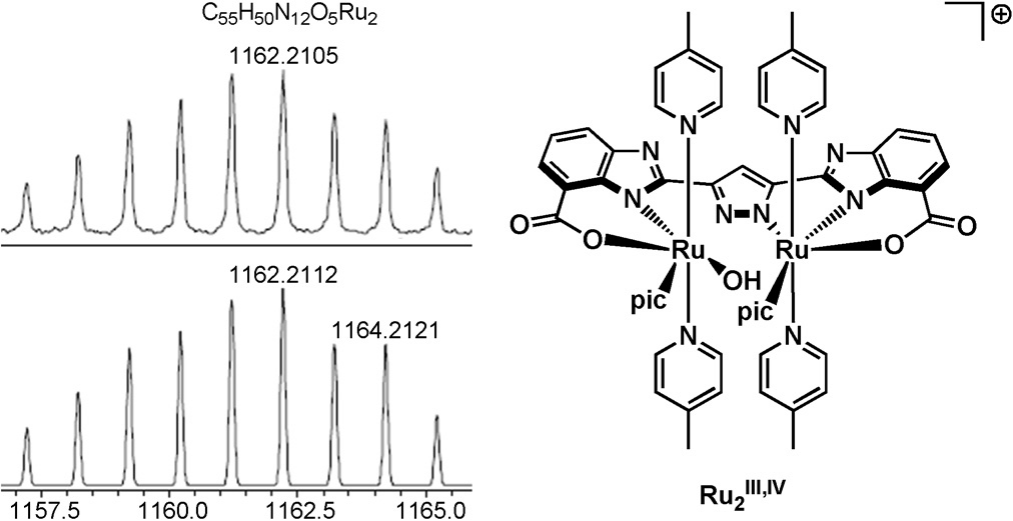
Top: High-resolution mass spectrum of the formal [(L)Ru_2_^III,IV^(pic)_6_(OH)]^+^ ([(HL)Ru_2_^III,IV^(pic)_6_(OH)]^2+^−H^+^) species recorded in positive mode. The actual structure is probably [(H_2_L)Ru_2_^III,IV^(pic)_6_(O)]^2+^, see Figure S28. Bottom: Simulated spectrum.

It was also possible to detect a peak at *m*/*z* 1162.2106 with an isotope pattern matching the Ru_2_^III,IV^ species [(L)Ru_2_^III,IV^(pic)_6_(OH)]^+^ (Figure 5), which is generated according to Equation (4). The [(HL)Ru_2_^III,III^(pic)_6_(OH_2_)]^2+^ and [(L)Ru_2_^III,IV^(pic)_6_(OH)]^+^ species were initially believed to contain a seven-coordinated Ru center. However, quantum chemical calculations suggest intermediates in which one of the coordinating pyrazole nitrogen atoms has been detached from the Ru center (see below). Thus, the dissociation of one of the Ru centers from the bridging pyrazole ligand scaffold creates an open coordination site for the incoming aqua ligand, thereby bypassing the formation of the unfavorable seven-coordinated intermediate. These results suggest that the designed pyrazole ligand motif actively promotes coordination of H_2_O during catalysis and that this metal–ligand cooperation contributes to the catalytic activity of Ru complex **1**.

**Figure 6:**
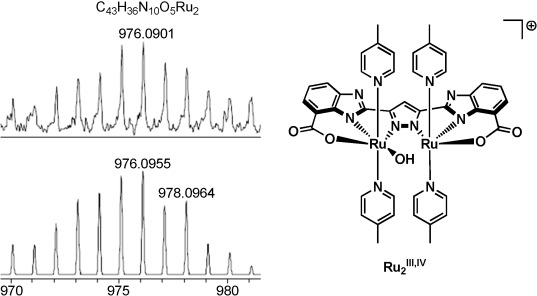
Top: High-resolution mass spectrum of the formal [(L)Ru_2_^III,IV^(pic)_4_(OH)]^+^ ([(HL)Ru_2_^III,IV^(pic)_4_(OH)]^2+^−H^+^) species recorded in positive mode. Bottom: Simulated spectrum.

A peak at *m*/*z* 976.0901 matching the Ru_2_^III,IV^ intermediate [(L)Ru_2_^III,IV^(pic)_4_(OH)]^+^ (Figure 6) was also observed and is assumed to arise from the conversion of [(L)Ru_2_^III,IV^(pic)_6_(OH)]^+^ (*m*/*z* 1162.2106). This highlights that oxidation of the Ru_2_^III,III^ species [(HL)Ru_2_^III,III^(pic)_6_(OH_2_)]^2+^ to the Ru_2_^III,IV^ state is accompanied by loss of picoline. The [(L)Ru_2_^III,IV^(pic)_4_(OH)]^+^ species thus contains a free coordination site, which should enable the coordination of a second aqua ligand. Facilitated by the electron-rich ligand scaffold, such a diaqua Ru species should readily be able to access even higher redox states, which ultimately triggers O=O bond formation. The fundamental catalytic features, which are thus provided by HRMS, are important for understanding the properties of the dinuclear Ru complex **1** as illustrated by the intricate Pourbaix diagram, see Figure 11.

### Quantum chemical description of the key intermediates

**Figure 7:**
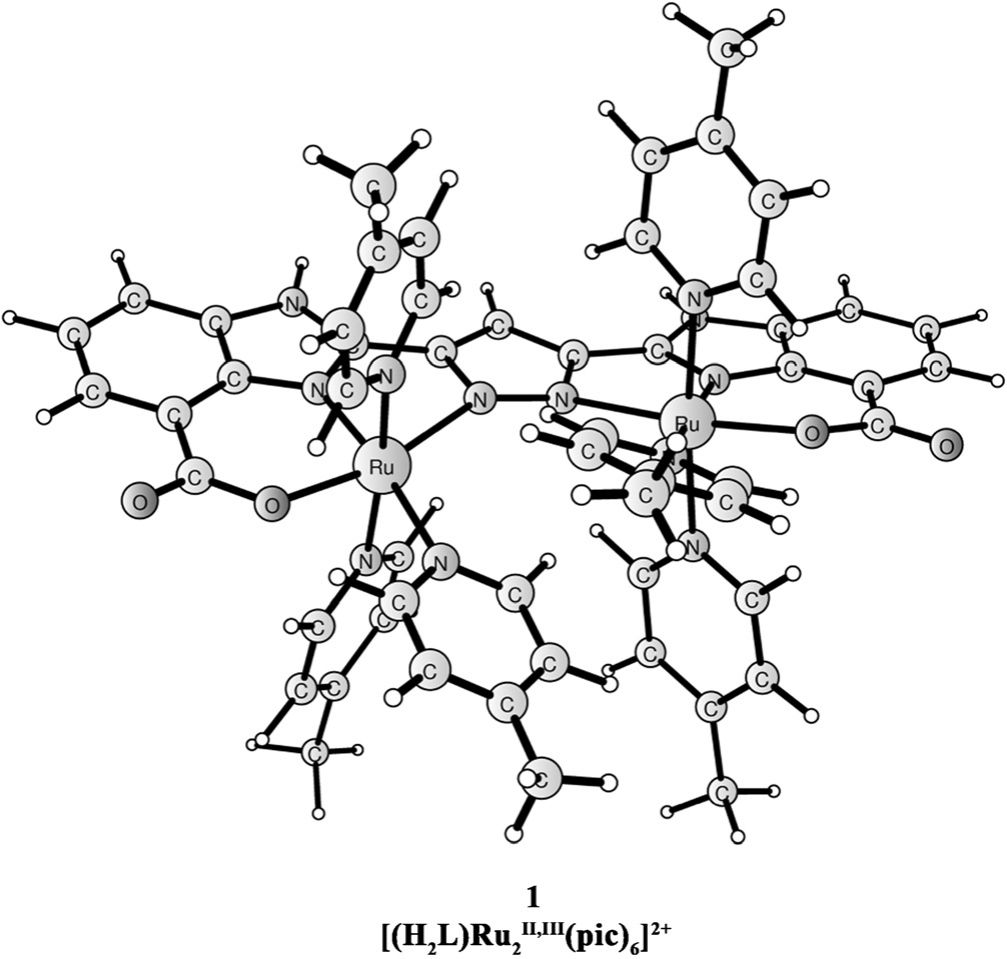
Calculated structure of the dinuclear Ru complex 1 ([(H_2_L)Ru_2_^II,III^(pic)_6_]^2+^).

Density functional theory (DFT) calculations[22] have proven to be essential for understanding the processes leading to H_2_O oxidation and to study the influence of the ligand on its environment in artificially developed WOCs at different redox states. Aiming to understand the different redox processes that are involved for Ru complex **1**, the redox potentials for the three complexes ([(H_2_L)Ru_2_(pic)_6_], [(H_2_L)Ru_2_(pic)_6_(OH_2_)], and [(H_2_L)Ru_2_(pic)_4_(OH_2_)_2_]) were calculated (Table 1). By calculating a large number of relevant structures housing different numbers of picoline and aqua/hydroxo/oxo ligands in different redox states, different protonation states, isomers, and possible spin states, detailed insight was gained. All the relevant structures are shown in the Supporting Information (Figures S1–S44). The calculated structures of the Ru_2_^II,III^ species are depicted in Figure 7 and the Supporting Information (Figures S6–S12). In acidic aqueous solutions, in the Ru_2_^II,II^ and Ru_2_^II,III^ redox states, the dominant form of the Ru complex should be [(H_2_L)Ru_2_(pic)_6_]^2+^ (see Table 1), which is also supported experimentally (see Figure 11).

**Table 1:** Comparison of experimental redox potentials for Ru complex 1 with calculated redox potentials for different Ru species with varying picoline ligands at different pH.

Experimental potential at pH 3				Calculated potential at pH 3			
[V vs. NHE]				[V vs. NHE]			
		[(H_2_L)Ru_2_(pic)_6_]	[(H_2_L)Ru_2_(pic)_6_(OH_2_)]	[(H_2_L)Ru_2_(pic)_4_(OH_2_)_2_]			
Redox couple	*E*	Redox couple	*E*	Redox couple	*E*	Redox couple	*E*
1st	0.24	Ru_2_^III,II^/Ru_2_^II,II^	0.19	Ru_2_^III,II^/Ru_2_^II,II^	0.05	Ru_2_^III,II^/Ru_2_^II,II^	0.09
2nd	0.38, 0.54	Ru_2_^III,III^/Ru_2_^II,III^	0.63	Ru_2_^III,III^/Ru_2_^II,III^	0.48	Ru_2_^III,III^/Ru_2_^II,III^	0.27
3rd	0.78	Ru_2_^III,IV^/Ru_2_^III,III^	1.32	Ru_2_^III,IV^/Ru_2_^III,III^	0.94		
4th	0.97					Ru_2_^III,IV^/Ru_2_^III,III^	1.05
5th	1.16					Ru_2_^IV,IV^/Ru_2_^III,IV^	1.14
6th	1.33					Ru_2_^IV,V^/Ru_2_^IV,IV^	1.33

Experimental potential at pH 5				Calculated potential at pH 5			
[V vs. NHE]				[V vs. NHE]			
		[(H_2_L)Ru_2_(pic)_6_]	[(H_2_L)Ru_2_(pic)_6_(OH_2_)]	[(H_2_L)Ru_2_(pic)_4_(OH_2_)_2_]			
Redox couple	*E*	Redox couple	*E*	Redox couple	*E*	Redox couple	*E*
1st	0.14	Ru_2_^III,II^/Ru_2_^II,II^	0.19	Ru_2_^III,II^/Ru_2_^II,II^	0.05	Ru_2_^III,II^/Ru_2_^II,II^	−0.03
2nd	0.39, 0.42	Ru_2_^III,III^/Ru_2_^II,III^	0.56	Ru_2_^III,III^/Ru_2_^II,III^	0.48	Ru_2_^III,III^/Ru_2_^II,III^	0.27
3rd	0.58	Ru_2_^III,IV^/Ru_2_^III,III^	1.16	Ru_2_^III,IV^/Ru_2_^III,III^	0.70		
4th	0.81					Ru_2_^III,IV^/Ru_2_^III,III^	0.84
5th	1.03					Ru_2_^IV,IV^/Ru_2_^III,IV^	1.01
6th	1.24					Ru_2_^IV,V^/Ru_2_^IV,IV^	1.20

Experimental potential at pH 7.2				Calculated potential at pH 7.2			
[V vs. NHE][a]				[V vs. NHE]			
		[(H_2_L)Ru_2_(pic)_6_]	[(H_2_L)Ru_2_(pic)_6_(OH_2_)]	[(H_2_L)Ru_2_(pic)_4_(OH_2_)_2_]			
Redox couple	*E*	Redox couple	*E*	Redox couple	*E*	Redox couple	*E*
1st	0.02	Ru_2_^III,II^/Ru_2_^II,II^	0.19	Ru_2_^III,II^/Ru_2_^II,II^	0.05	Ru_2_^III,II^/Ru_2_^II,II^	−0.15
2nd	0.35[b]	Ru_2_^III,III^/Ru_2_^II,III^	0.39	Ru_2_^III,III^/Ru_2_^II,III^	0.43	Ru_2_^III,III^/Ru_2_^II,III^	0.14
3rd	0.47	Ru_2_^III,IV^/Ru_2_^III,III^	1.07	Ru_2_^III,IV^/Ru_2_^III,III^	0.49		
4th	0.64					Ru_2_^III,IV^/Ru_2_^III,III^	0.66
5th	0.86					Ru_2_^IV,IV^/Ru_2_^III,IV^	0.85
6th	1.12[c]					Ru_2_^IV,V^/Ru_2_^IV,IV^	1.14

[a] Electrochemical potentials were obtained from DPV in an aqueous Britton–Robinson buffer solution (0.1 m). Conditions: Scan rate 0.1 V s^−1,^ glassy carbon disk as working electrode, a platinum spiral as counter electrode and a saturated calomel electrode (SCE) as reference electrode. Potentials were converted to NHE by using the [Ru(bpy)_3_]^3+^/[Ru(bpy)_3_]^2+^ couple as a standard (*E*_1/2_=1.26 V vs. NHE).

[b] This is most likely two separate redox processes that cannot be distinguished due to overlap of the two peaks.

[c] A catalytic current for H_2_O oxidation (onset potential) occurs after this redox process at 1.20 V versus NHE.

Since the Ru_2_^III,III^ state can easily be accessed under neutral conditions, at which catalysis occurs (see Table 1 and Figures S13–S25), the possible ligand exchange processes were studied at different redox states. The various structures are shown in the Supporting Information (Figures S13–S38). Calculations show that the Ru_2_^III,III^ complex prefers to coordinate six picoline ligands ([(L)Ru_2_^III,III^(pic)_6_]), and therefore the energy of this complex was set to be zero. At pH 7.2, this complex has a total charge of +1 and the insertion of an aqua ligand could lead to a seven-coordinated Ru center, which has previously been reported for Ru-based WOCs.[6e] However, the calculations suggest that this does not occur for Ru complex **1**, instead one Ru center appears to dissociate from the bridging pyrazole ligand, thus resulting in an open coordination site for an aqua ligand to occupy (Figure 8).

**Figure 8:**
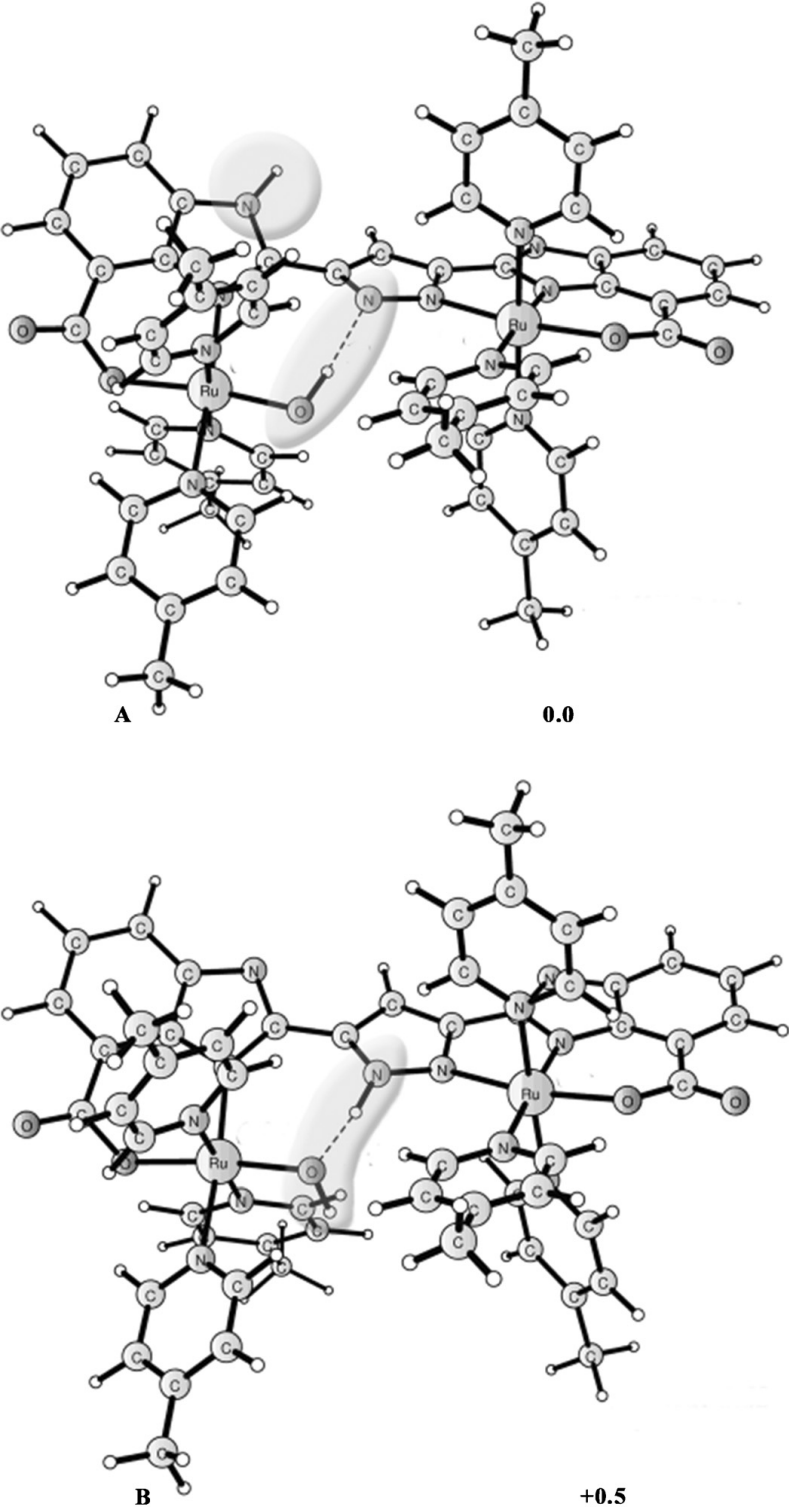
Calculated structures of two of the isomers (A and B) of the [(L)Ru_2_^III,III^(pic)_6_(OH_2_)]^+^ complex with the non-innocent role of the ligand framework highlighted.

An additional feature of the developed ligand scaffold, which was seen from calculations, is that several different isomeric structures can co-exist. Transfer of a proton from the Ru-bound aqua ligand to the imidazole nitrogen to yield structure A (see Figure 8 and the Supporting Information, Figure S15) was found to result in the most stable isomer. Structure B was found to be slightly higher in energy and have the proton at the pyrazole moiety. The overall energetics of this aqua addition process was thus revealed to be endergonic by 2.5 kcal mol^−1^ (see the Supporting Information, Figure S47). The active participation of the engineered ligand framework in ligand exchange in Ru complex **1** is an intriguing feature that might facilitate coordination processes during the catalytic cycle and enhance proton-coupled events. From the generated formal [(L)Ru_2_^III,III^(pic)_6_(OH_2_)]^+^ intermediate, one picoline ligand can dissociate from the Ru center housing the “hydroxide” ligand (structure A in Figure 8), which is concomitant with recoordination of the pyrazole nitrogen to the Ru center. This step was calculated to be close to isogonic (see the Supporting Information, Figure S47). The picoline-aqua ligand exchange thus takes place through an associative-type of mechanism, in which the bridging pyrazole ligand plays a key role in providing access for coordination of the aqua ligand.

**Figure 9:**
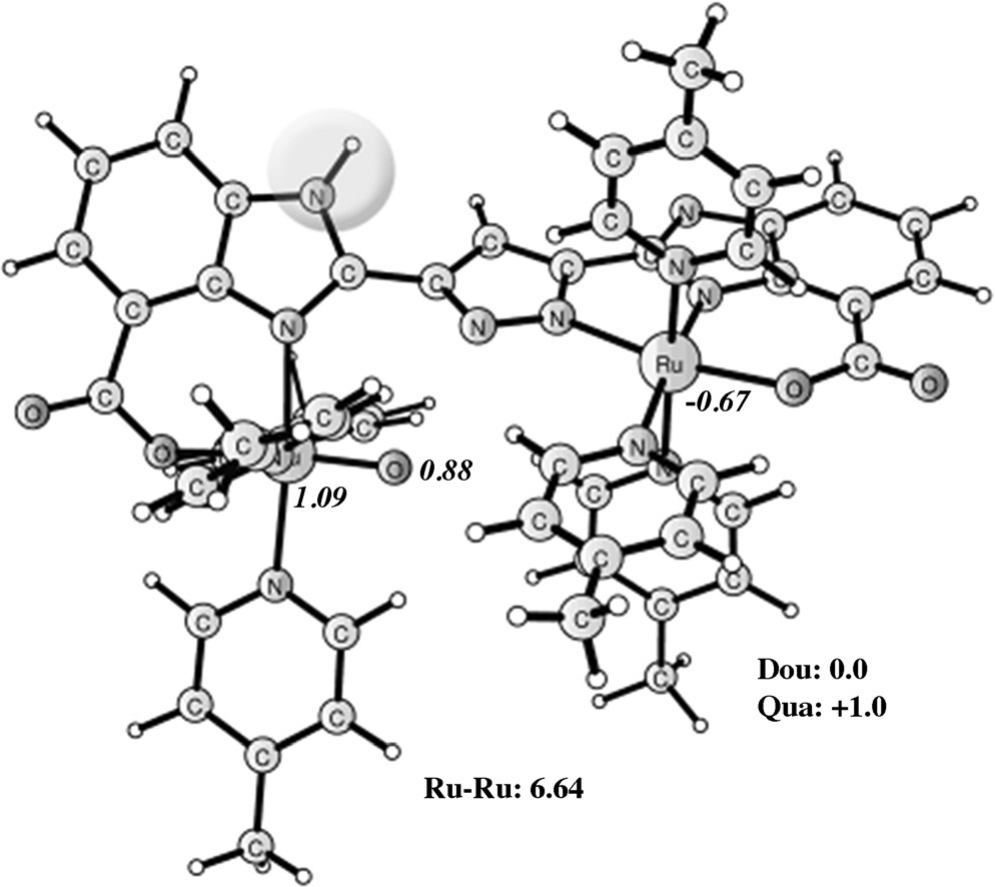
Calculated structure of the most stable isomer of the formal [(L)Ru_2_^III,IV^(pic)_6_(OH)]^+^ complex.

A second aqua ligand can then be coordinated to the other Ru center in a similar fashion. Here it was also shown that the bridging pyrazole has a non-innocent role in affording an open coordination site by dissociating from the metal center. The resulting complex (the Supporting Information, Figure S21) thus has five picoline ligands and two aqua ligands ([(L)Ru_2_^III,III^(pic)_5_(OH_2_)_2_]^+^) and is the least favorable species in the ligand exchange Scheme as its energy is +5.4 kcal mol^−1^ relative to the starting complex [(L)Ru_2_^III,III^(pic)_6_]^+^ (see the Supporting Information, Figure S47). Finally, picoline dissociation from [(L)Ru_2_^III,III^(pic)_5_(OH_2_)_2_]^+^ leads to the expected [(L)Ru_2_^III,III^(pic)_4_(OH_2_)_2_]^+^ complex that may initiate H_2_O oxidation. A similar ligand exchange analysis was performed for the developed Ru complex in its Ru_2_^III,IV^ state. Here it was found that the oxidation of the [(L)Ru_2_^III,III^(pic)_6_]^+^ complex to its Ru_2_^III,IV^ state was associated with a relatively high redox potential of 1.07 V versus NHE at pH 7.2 (see Table 1), as a result of the fact that proton-coupled oxidations are prohibited for [(L)Ru_2_^III,III^(pic)_6_]^+^. However, the introduction of an aqua ligand to yield the formal mono-aqua complex [(L)Ru_2_^III,III^(pic)_6_(OH_2_)]^+^, makes proton-coupled electron transfer (PCET) oxidation(s) possible, leading to a significantly lowering of the redox potential to merely 0.49 V versus NHE. This suggests that the Ru_2_^III,IV^ state can be accessed under oxygen (air) atmosphere and for a proton-coupled redox process the [(HL)Ru_2_^III,IV^(pic)_6_(O)]^+^ species was found to be the most stable form of the different isomers (Figure 9 and the Supporting Information, Figure S27).

As previously noted, HRMS measurements showed a peak at *m*/*z* 1162.2106 (see Figure 5), corresponding to the formal species [(L)Ru_2_^III,IV^(pic)_6_(OH)]^+^ ([{(L)Ru_2_^III,IV^(pic)_6_(OH_2_)}^2+^−H^+^]^+^), which further supports the non-innocence of the developed ligand. According to the calculations (the Supporting Information, Figure S28), the most stable isomer below pH 9 is [(H_2_L)Ru_2_^III,IV^(pic)_6_(O)]^2+^. The overall ligand exchange process to generate the formal [(L)Ru_2_^III,IV^(pic)_4_(OH)_2_] complex is endergonic by 3.2 kcal mol^−1^ (the Supporting Information, Figure S48). However, considering that the Ru complex concentration (μm) is much lower than the H_2_O concentration (55.5 m), [(L)Ru_2_^III,IV^(pic)_4_(OH)_2_] should also be an existing species at the Ru_2_^III,IV^ state.

From Table 1 it is obvious that for the two highest redox processes for the [(L)Ru_2_(pic)_4_(OH_2_)_2_] species, namely the Ru_2_^IV,IV^/Ru_2_^III,IV^, and Ru_2_^IV,V^/Ru_2_^IV,IV^ redox events, the agreement between the calculated and experimentally obtained potentials is excellent and highlights that access to the higher redox states requires the generation of the diaqua complex [(L)Ru_2_(pic)_4_(OH_2_)_2_].

**Figure 10:**
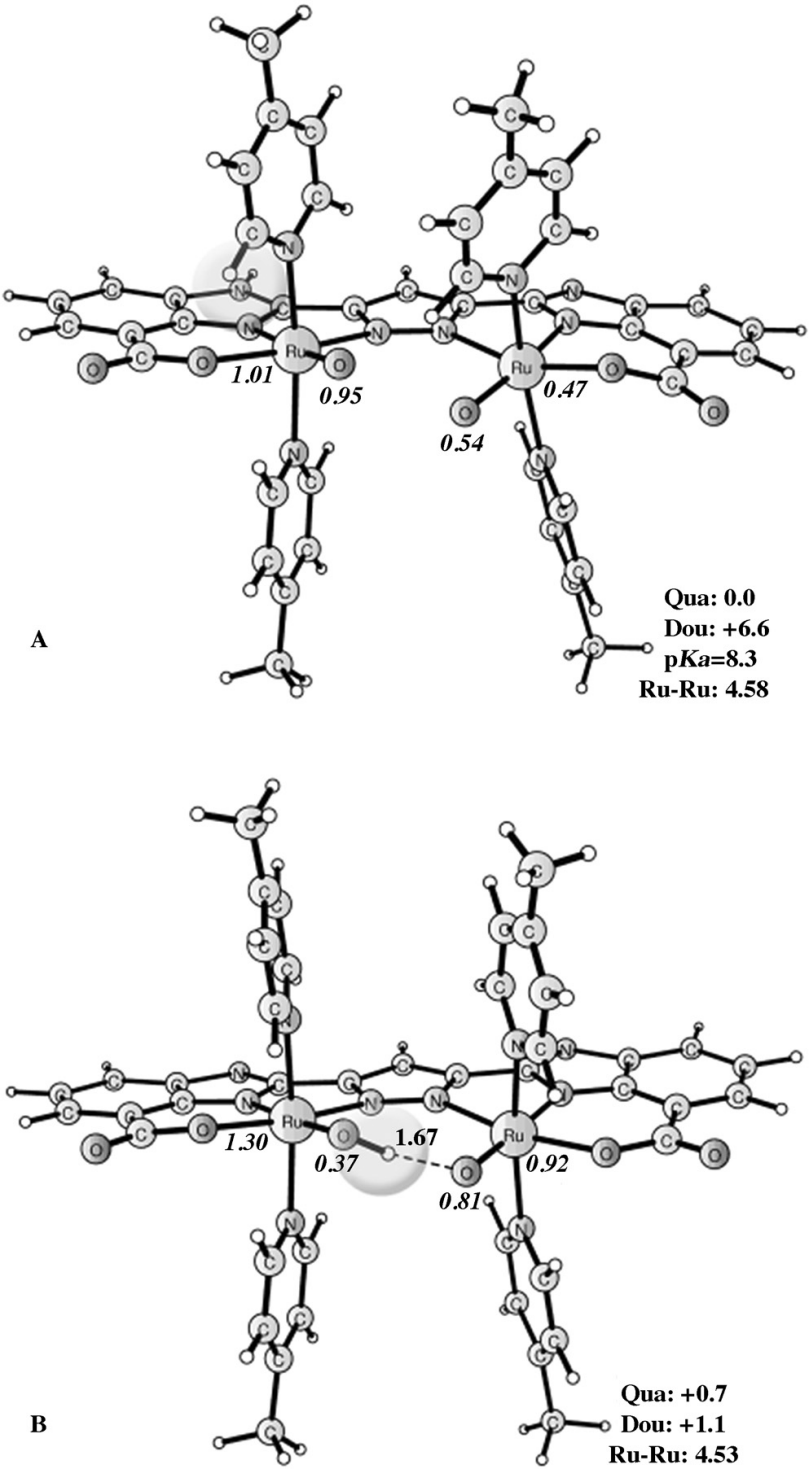
Calculated structures of the two isomers of the [(L)Ru_2_^IV,V^(pic)_4_(O)(OH)]^+^ complex.

The calculations further revealed that the formal [(L)Ru_2_^III,IV^(pic)_4_(OH)_2_] complex is better described as [(L)Ru_2_^III,IV^(pic)_4_(O)(OH_2_)], in which the oxo moiety has significant oxyl character (Ru_2_^III,III^=O**^.^**), with a spin density of 0.70 (see the Supporting Information, Figure S35 A). The subsequent redox process, which has a redox potential of 0.85 V, was also found to be proton-coupled and results in the formation of [(L)Ru_2_^IV,IV^(pic)_4_(O)(OH)]. Finally, the highest redox process under consideration was a one-electron oxidation that generates a formal Ru_2_^IV,V^ complex at a modest potential of 1.14 V thanks to the crafted ligand framework in complex **1**. However, here the electronic structure is better described as a mixture of Ru_2_^III,V^(O**^.^**)(O) and Ru^III,IV^(O**^.^**)_2_, in which the spin densities are ≈1.0 on each of the Ru^III^ center and the connecting oxygen, and ≈0.5 on the Ru^IV^ center and the connected oxygen (structure A in Figure 10). From this high-valent species, O=O bond formation should readily occur, thereby affording a pathway for activation of H_2_O in which the cooperative involvement of the ligand motif has a vital role during catalysis.

### Mechanistic analysis from electrochemical measurements

To obtain further insight into the catalytic properties of the dinuclear Ru complex **1**, electrochemical measurements were conducted to construct the Pourbaix diagram. Pourbaix diagrams are important tools for highlighting possible stable redox states of the studied catalytic system. Here, the Nernst Equation ([Eq. (5)]:

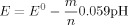
(5)

in which *m* is the number of protons transferred and *n* is the number of electrons transferred) describes the potential dependencies for reactions involving proton transfer. Thus the potential of a one-electron-one-proton redox event will decrease by ≈59 mV per pH unit, whereas a two-electron-one-proton process will decrease by ≈29 mV per pH unit. These features make it possible to distinguish between the different processes and to gain insight into the catalytic properties of the examined system.

**Figure 11:**
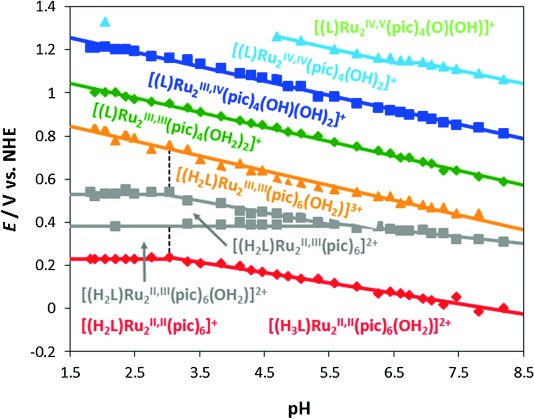
Pourbaix diagram for the dinuclear Ru complex 1 in the range 1.5<pH<8.5 with the formal species at different redox states shown. At the Ru_2_^II,II,^ Ru_2_^II,III^, and Ru_2_^III,III^ states the complex exists as a mixture of different species, see the main text for further discussion. Conditions: The Pourbaix diagram was obtained in a 0.1 m Britton-Robinson buffer solution in the range of 1.5<pH<8.5. The pH of the solution was changed by using a 0.2 m aq. NaOH solution.

Table 1 summarizes the electrochemical properties for Ru complex **1** at different pH. None of the oxidations appear as reversible peaks in the cyclic voltammogram at pH 7.2. However, reasonably resolved peaks were obtained by differential pulse voltammetry (DPV). The Pourbaix diagram of the dinuclear Ru complex **1** was subsequently constructed by measuring the redox potentials by DPV at different pH, 1.5<pH<8.5, and is depicted in Figure 11. This diagram is quite complicated, however, by the use of quantum chemical calculations and MS it was possible to assign the different redox steps. It was found that Ru complex **1** exhibited several redox events involving PCET, as evident by the decrease of the redox potentials with increasing pH.

On dissolution of Ru complex **1** in aqueous solutions, HRMS suggest that [(H_2_L)Ru_2_^II,III^(pic)_6_]^2+^ (**1**) is the initial species at the Ru_2_^II,III^ state,[19] which is further supported by the quantum chemical calculations. The Ru_2_^II,III^/Ru_2_^II,II^ and Ru_2_^III,III^/Ru_2_^II,III^ redox couples should therefore correspond to the oxidation of this picoline species. In Table 1, the calculations show that this is true for Ru_2_^II,III^/Ru_2_^II,II^ at pH<3, in which the experimentally measured and calculated potentials, 0.24 and 0.19 V, respectively, agree. However, the calculations suggest that the redox process should be pH-independent at pH>3, which does not agree with what is observed experimentally. Instead, the redox process becomes proton-coupled at higher pH, with a slope corresponding to a one-electron-one-proton process. Although it was only possible to observe [(H_2_L)Ru_2_^II,III^(pic)_6_]^2+^ (**1**) at the Ru_2_^II,III^ state by HRMS, both the [(H_2_L)Ru_2_^II,III^(pic)_6_(OH_2_)]^2+^ and [(H_2_L)Ru_2_^II,III^(pic)_4_(OH_2_)_2_]^2+^ species can form relatively readily, see Figure S46 (the Supporting Information). In its reduced form (Ru_2_^II,II^ state), the latter species should undergo proton-coupled electron transfer (Table 1), which might explain the experimental results. Alternatively, [(H_2_L)Ru_2_^II,II^(pic)_6_(OH_2_)]^+^ could be protonated to give [(H_3_L)Ru_2_^II,III^(pic)_6_(OH_2_)]^2+^, which would be capable of undergoing PCET, as suggested in the Pourbaix diagram (Figure 11).

At acidic pH (pH 3), the redox process at 0.54 V most likely involves the oxidation of [(H_2_L)Ru_2_^II,III^(pic)_6_]^2+^ (**1**) to [(H_2_L)Ru_2_^III,III^(pic)_6_]^3+^ (Figure 3), which was calculated to occur at 0.63 V (see Table 1). This process becomes proton-coupled at pH>3 and probably involves the oxidation of [(H_2_L)Ru_2_^II,III^(pic)_6_]^2+^ to [(HL)Ru_2_^III,III^(pic)_6_]^2+^, and merges at pH≈6 with a second event at 0.38 V, which corresponds to the [(H_2_L)Ru_2_^III,III^(pic)_6_(OH_2_)]^3+^/[(H_2_L)Ru_2_^II,III^(pic)_6_(OH_2_)]^2+^ redox couple. The combined redox process is observed as a formal two-electron-one-proton event with a decrease of potential by ≈29 mV per pH unit after pH 6 (see Figure 11). At the Ru_2_^III,III^ state, one would thus initially expect the formation of a mixture of [(H_2_L)Ru_2_^III,III^(pic)_6_]^3+^ and [(H_2_L)Ru_2_^III,III^(pic)_6_(OH_2_)]^3+^ at pH<3, and [(HL)Ru_2_^III,III^(pic)_6_]^2+^ and [(HL)Ru_2_^III,III^(pic)_6_(OH_2_)]^2+^ at neutral pH. This is further supported by HRMS in which the [(HL)Ru_2_^III,III^(pic)_6_]^2+^ species could be detected (Figure 3). This species is then converted to [(HL)Ru_2_^III,III^(pic)_6_(OH_2_)]^2+^ (Figure 4 and the Supporting Information, Figure S47). Also, [(HL)Ru_2_^III,III^(pic)_4_(OH)(OH_2_)]^+^ can be generated at the Ru_2_^III,III^ state (Figure S47), although there is no HRMS evidence for its formation at this redox state.

According to the calculations (see Table 1) the next level in the Pourbaix diagram, which occurs at 0.47 V at neutral pH, corresponds to the proton-coupled oxidation of [(H_2_L)Ru_2_^III,III^(pic)_6_(OH_2_)]^3+^ to the formal [(HL)Ru_2_^III,IV^(pic)_6_(OH_2_)]^3+^ species. The oxidation of the related formal diaqua complex [(L)Ru_2_^III,III^(pic)_4_(OH_2_)_2_]^+^ requires a slightly higher potential (0.64 V at neutral conditions) and results in the formation of the formal [(L)Ru_2_^III,IV^(pic)_4_(OH)(OH_2_)]^+^ species (see Figure 11). This assignment is further supported by HRMS after a two-electron oxidation of Ru complex **1**, see Figures 5 and 6.

The two subsequent redox processes, occurring at 0.86, and 1.12 V at neutral conditions, are also pH-dependent with a slope of −59 mV per pH unit over the whole studied pH range and correspond to the formal oxidations [(L)Ru_2_^III,IV^(pic)_4_(OH)(OH_2_)]^+^→[(L)Ru_2_^IV,IV^(pic)_4_(OH)_2_]^+^→[(L)Ru_2_^IV,V^(pic)_4_(O)(OH)]^+^ (Table 1). This suggests that it is necessary to access the high-valent Ru_2_^IV,V^ state before O=O bond formation can be triggered.

**Figure 12:**
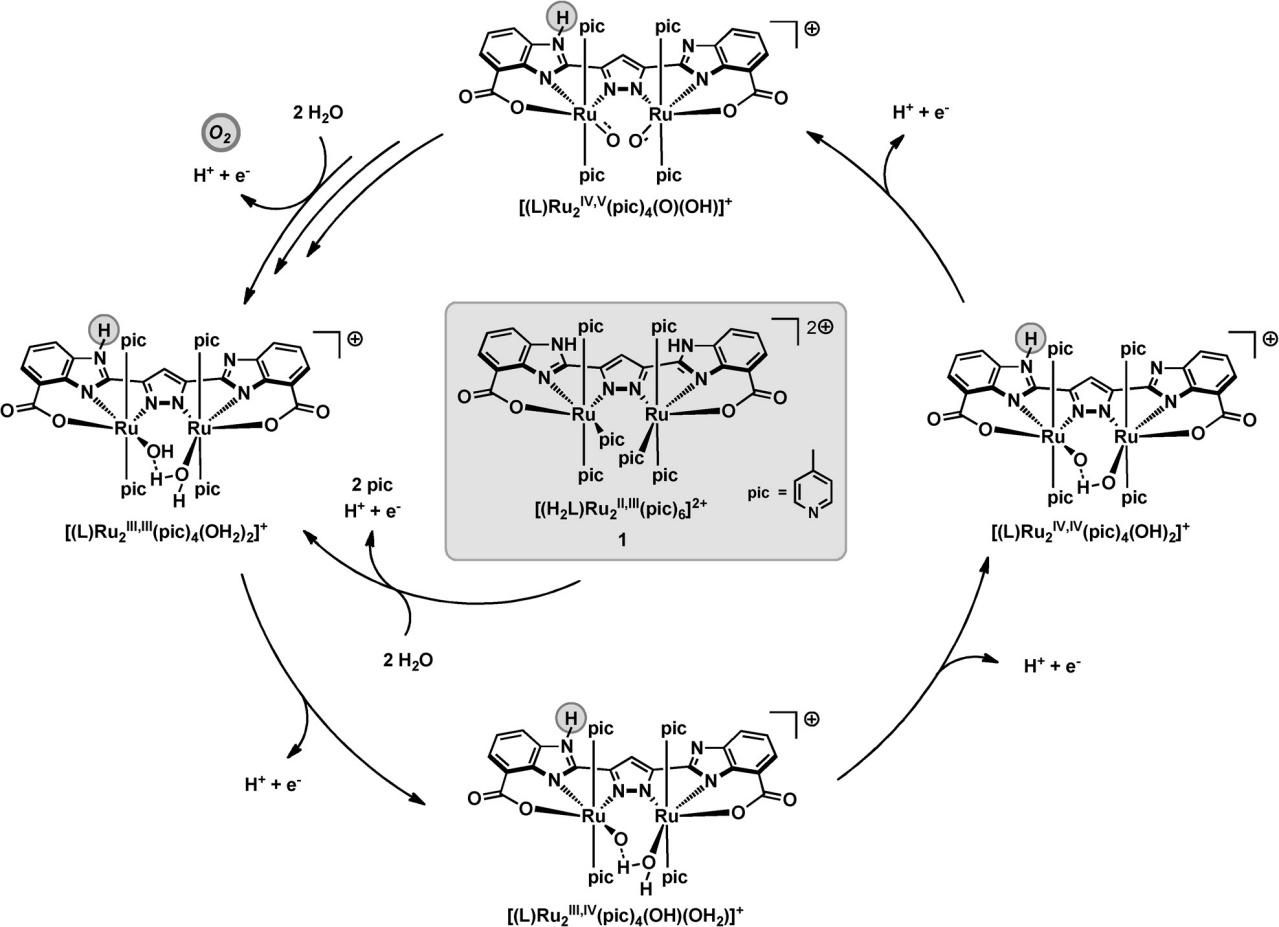
Proposed mechanistic cycle for O_2_ formation at neutral pH for Ru complex 1. The calculated isomers with the lowest energies are shown at the different redox states.

A striking feature that is apparent from Table 1, is that when comparing the calculated and experimental Ru_2_^III,III^/Ru_2_^II,III^ redox potentials at pH≈5, the Ru complex **1** seems to exist in two forms, namely as [(H_2_L)Ru_2_(pic)_6_]^2+^ and [(H_2_L)Ru_2_(pic)_6_(OH_2_)]^2+^. This phenomenon also seems to prevail at pH 7, in which the complex appears to co-exist as [(H_2_L)Ru_2_(pic)_6_]^2+^ and [(H_2_L)Ru_2_(pic)_6_(OH_2_)]^2+^ (see Table 1). Collectively, this shows that aquation of Ru complex **1** occurs more easily at neutral pH, thus causing the complex to exist as different species containing varying amounts of picoline ligands, which explains that more redox peaks are observed in the Pourbaix diagram than are intuitively expected. During the redox processes the ligand scaffold actively participates, which facilitates access to the higher redox states and ultimately triggers O=O bond formation (see Figure 12).

## Conclusion

Introduction of the designed electron-rich pentacoordinating ligand to generate Ru complex **1** significantly lowers the redox potentials for the complex and allows access to a wide variety of redox states. This work uncovers the fundamental mechanistic details by which the dinuclear Ru complex **1** mediates H_2_O oxidation, which is essential for the development of more efficient WOCs. The combination of experimental analysis and quantum chemical calculations resulted in the discovery of vital features associated with Ru complex **1**. One vital feature of the ligand motif in Ru complex **1** is the non-innocent behavior, since it participates in both proton transfer and accepting/donating electrons. This metal–ligand cooperation plays a key role during the multi-electron oxidation of H_2_O. These results highlight that the engineering of catalysts for the activation of H_2_O using non-innocent molecular scaffolds may be of value for the future construction of efficient WOCs.

## Experimental Section

### Materials and methods

Ru complex **1**[19] and [Ru(bpy)_3_](PF_6_)_3_[23] were prepared according to previously reported procedures. High-resolution mass spectra measurements were recorded on a Bruker Daltonics microTOF spectrometer equipped with an electrospray ionization (ESI) source, and the experimental parameters were set to the following: Capillary temperature, 180 °C; capillary voltage, 4500 V; flow rate, 4.0 L min^−1^; capillary exit, 160 V; skimmer, 53.3 V; hexapole, 24 V.

### Electrochemistry

Electrochemical measurements were carried out with an Autolab potentiostat with a GPES electrochemical interface (Eco Chemie), using a glassy carbon disk (diameter 3 mm) as the working electrode, and platinum as the counter-electrode. The reference electrode was a saturated calomel electrode (SCE). All potentials reported herein are converted to normal hydrogen electrode (NHE) by using the [Ru(bpy)_3_]^3+^/[Ru(bpy)_3_]^2+^ couple (*E*_1/2_=1.26 V vs. NHE) as reference. The Pourbaix diagram of Ru complex **1** was obtained in 0.1 m Britton–Robinson buffer solutions in the range of 1.5<pH<8.5. The pH of the solution was changed by using a 0.2 m NaOH aqueous solution.

### Computational details

The geometry optimizations in the present study were performed by using the Gaussian 09[24] package and the B3LYP[25] functional. The 6-31G(d,p) basis set was applied for the C, N, O, H elements and the SDD[26] pseudopotential for Ru. Frequencies were calculated analytically at the same level of theory as the geometry optimization to obtain the Gibbs free energy corrections and to confirm the nature of various stationary points. Solvation effects from the H_2_O solvent were calculated by using the SMD[27] continuum solvation model with the larger basis set in which all elements, except Ru, were described by 6-311+G(2df,2p) at the B3LYP* (15 % exact exchange) level.[28] It has been shown that B3LYP* gives a better description of relative energies in transition metal complexes.[28] For H_2_O, the experimental solvation free energy (−6.3 kcal mol^−1^) was used.[29] The concentration correction of 1.9 kcal mol^−1^ at room temperature (derived from the free-energy change of 1 mol of an ideal gas from 1 atm (24.5 L mol^−1^, 298.15 K) to 1 m was added for all species except H_2_O, for which the corresponding value is 4.3 kcal mol^−1^ as the standard state of H_2_O is 55.6 m. Unless otherwise specified, the B3LYP*-D2 energies are reported, including Gibbs free energy corrections from B3LYP and dispersion corrections proposed by Grimme.[30]

To calculate the redox potential, the absolute redox potential of the standard hydrogen electrode (4.281 V) was used as the reference,[31] which corresponds to 127.8 kcal mol^−1^ for a one-electron oxidation and 407.9 kcal mol^−1^ for a proton-coupled one-electron oxidation at pH 7.2. For the latter case, the gas phase Gibbs free energy of a proton is −6.3 kcal mol^−1^ and the experimental solvation free energy of the proton (−264.0 kcal mol^−1^) was used.[29] These values were also used for calculating the absolute p*K*_a_.
