# Supplementary material for: A Dinuclear Ruthenium-Based Water Oxidation Catalyst: Use of Non-Innocent Ligand Frameworks for Promoting Multi-Electron Reactions
Source: Chemistry. 2015 Apr 29;21(28):10039–48. doi: 10.1002/chem.201406613 (PMC4517172; doi:10.1002/chem.201406613)
Supplement: Supplementary file 1 [file chem0021-10039-sd1.pdf]

CHEMISTRY
A European Journal

Supporting Information

# A Dinuclear Ruthenium-Based Water Oxidation Catalyst: Use of Non-Innocent Ligand Frameworks for Promoting Multi-Electron Reactions

Tanja M. Laine,[a] Markus D. Kärkäs,*[a] Rong-Zhen Liao,*[a, b] Per E. M. Siegbahn,[a] and Björn Åkermark*[a]

chem_201406613_sm_miscellaneous_information.pdf

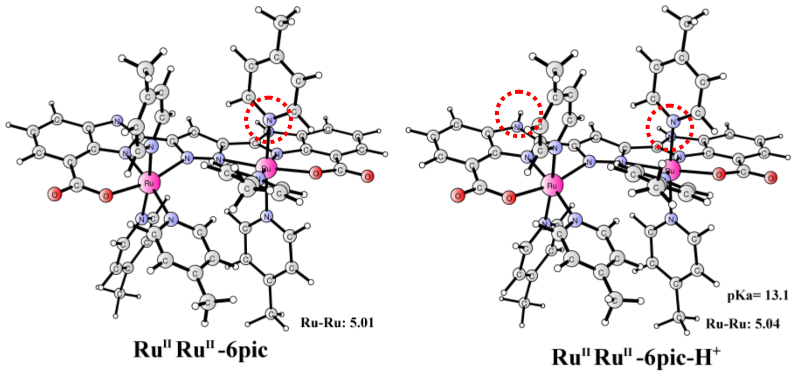


**Figure S1.** Optimized singlet structure of the $Ru_2^{II,II}$ complexes with six picoline ligands ($[Ru_2^{II,II}(pic)_6]$) in different protonation states (total charge from left to right: 0 and +1, respectively). The Ru-Ru distances are given in Ångström. Crucial protons are highlighted with red dotted circles.

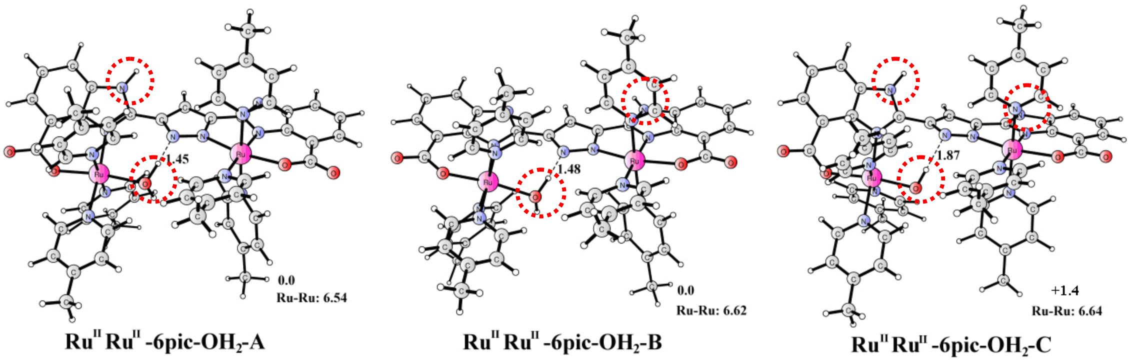


**Figure S2.** Optimized singlet structure of the $Ru_2^{II,II}$ complexes with six picoline ligands and an aqua ligand ($[(HL)Ru_2^{II,II}(pic)_6(OH_2)]$; total charge: 0). The Ru-Ru distances are given in Ångström. Crucial protons are highlighted with red dotted circles.

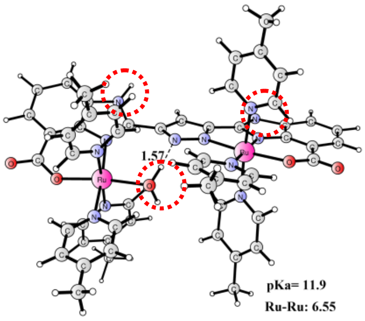


Ru$^{II}$ Ru$^{II}$ -6pic-H$^{+}$-OH$_2$

**Figure S3.** Optimized singlet structure of the protonated Ru$_2^{II,II}$ complex with six picoline ligands and an aqua ligand ([(H$_2$L)Ru$_2^{II,II}$(pic)$_6$(OH$_2$)]; total charge: +1). The Ru-Ru distances are given in Ångström. Crucial protons are highlighted with red dotted circles.

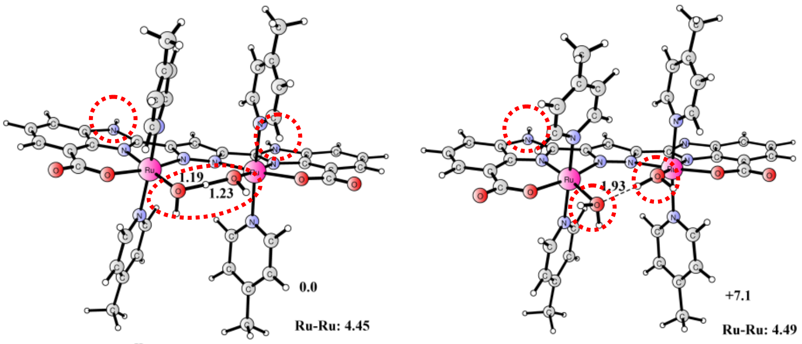


Ru$^{II}$ Ru$^{II}$ -4pic-2OH$_2$-A

Ru$^{II}$ Ru$^{II}$ -4pic-2OH$_2$-B

**Figure S4.** Optimized singlet structures of the Ru$_2^{II,II}$ complexes with four picoline ligands and two aqua ligand ([(HL)Ru$_2^{II,II}$(pic)$_4$(OH$_2$)$_2$]; total charge: 0). The Ru-Ru distances are given in Ångström. Crucial protons are highlighted with red dotted circles.

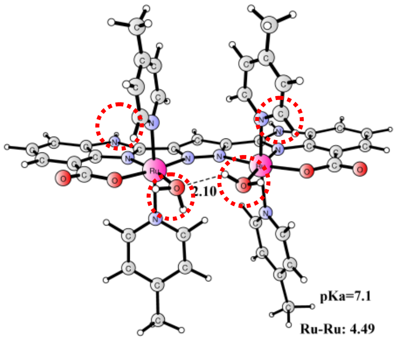


Ru$^{II}$ Ru$^{II}$-4pic-H$^{+}$-2OH$_2$

**Figure S5.** Optimized singlet structures of the protonated $Ru_2^{II,II}$ complex with four picoline ligands and two aqua ligands ($[(H_2L)Ru_2^{II,II}(pic)_4(OH_2)_2]$; total charge: +1). The Ru-Ru distances are given in Ångström. Crucial protons are highlighted with red dotted circles.

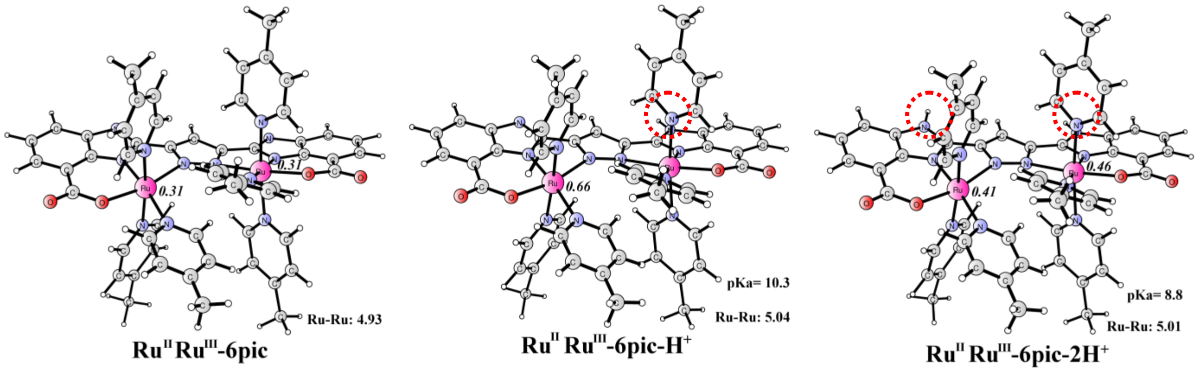


**Figure S6.** Optimized doublet structures of the $Ru_2^{II,III}$ complexes with six picoline ligands ($[Ru_2^{II,III}(pic)_6]$) in different protonation states (total charge from left to right: 0, +1 and +2, respectively). Spin densities on Ru are shown in italic and the Ru-Ru distances are given in Ångström. Crucial protons are highlighted with red dotted circles.

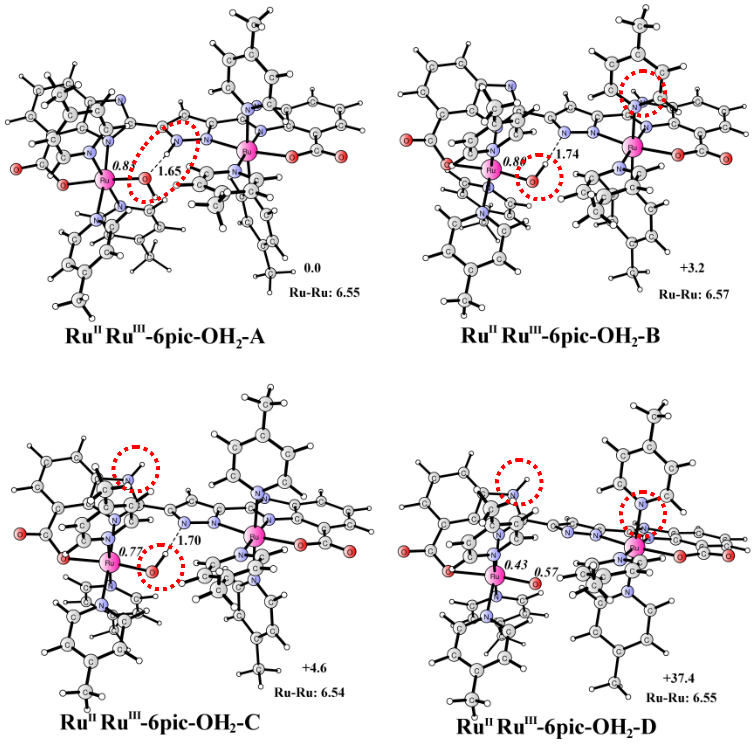

**Figure S7.** Optimized doublet structure of the $Ru_2^{II,III}$ complexes with six picoline ligands and an aqua ligand ($[(L)Ru_2^{II,III}(pic)_6(OH_2)]$; total charge: 0). Spin densities on Ru are shown in italic and the Ru-Ru distances are given in Ångström. Crucial protons are highlighted with red dotted circles.

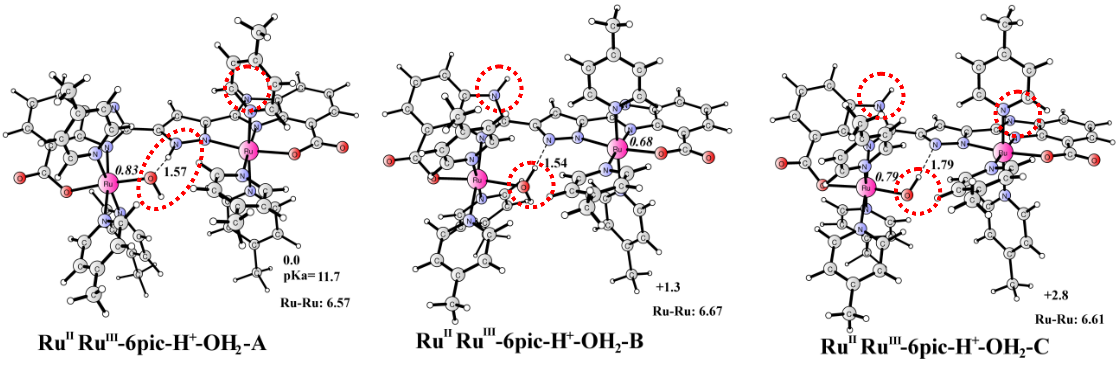


**Figure S8.** Optimized doublet structure of the protonated $Ru_2^{II,III}$ complexes with six picoline ligands and an aqua ligand ($[(HL)Ru_2^{II,III}(pic)_6(OH_2)]$; total charge: +1). Spin densities on Ru are shown in italic and the Ru-Ru distances are given in Ångström. Crucial protons are highlighted with red dotted circles.

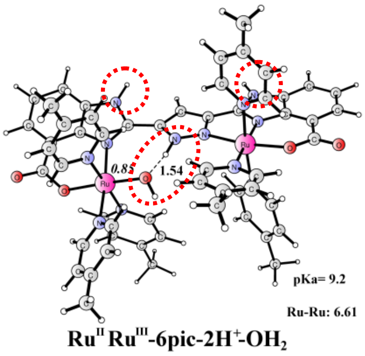


**Figure S9.** Optimized doublet structure of the diprotonated $Ru_2^{II,III}$ complex with six picoline ligands and an aqua ligand ($[(H_2L)Ru_2^{II,III}(pic)_6(OH_2)]$; total charge: +2). Spin densities on Ru are shown in italic and the Ru-Ru distances are given in Ångström. Crucial protons are highlighted with red dotted circles.

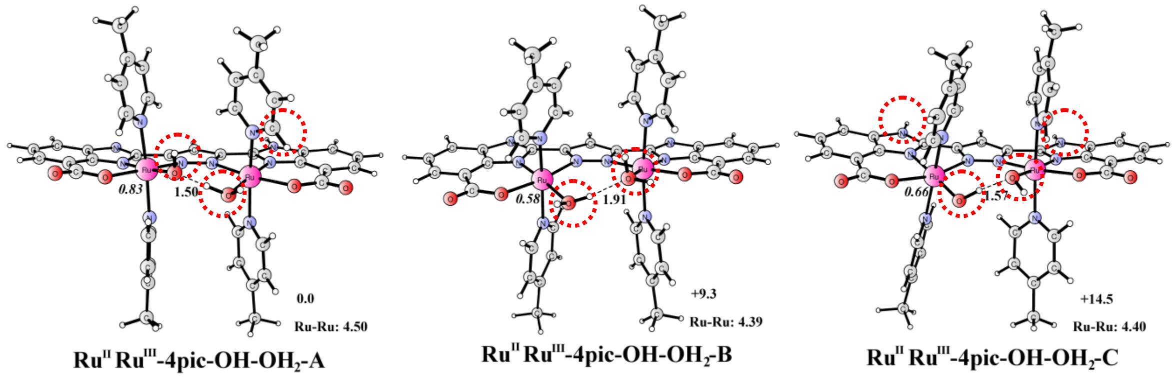


**Figure S10.** Optimized doublet structures of the $Ru_2^{II,III}$ complexes with four picoline ligands, a hydroxo ligand and an aqua ligand ($[(HL)Ru_2^{II,III}(pic)_4(OH)(OH_2)]$; total charge: 0). Spin densities on Ru are shown in italic and the Ru-Ru distances are given in Ångström. Crucial protons are highlighted with red dotted circles.

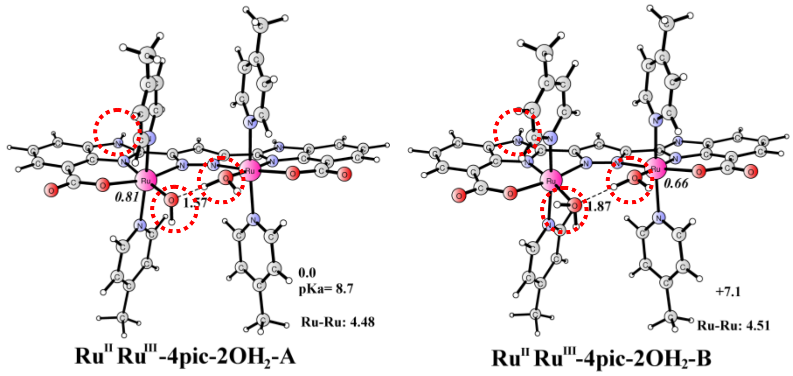


**Figure S11.** Optimized doublet structures of the $Ru_2^{II,III}$ complexes with four picoline ligands and two aqua ligands ($[(HL)Ru_2^{II,III}(pic)_4(OH_2)_2]$; total charge: +1). Spin densities on Ru are shown in italic, the calculated $pK_a$ is indicated and the Ru-Ru distances are given in Ångström. Crucial protons are highlighted with red dotted circles.

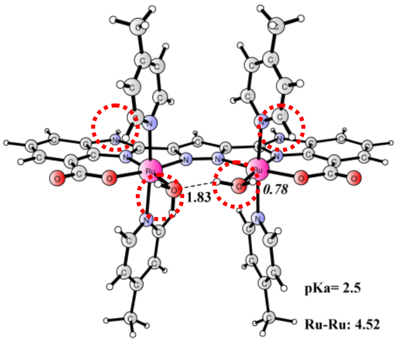


**$Ru^{II}Ru^{III}$-4pic-$H^+$-$2OH_2$**

**Figure S12.** Optimized doublet structures of the protonated $Ru_2^{II,III}$ complex with four picoline ligands and two aqua ligands ($[(H_2L)Ru_2^{II,III}(pic)_4(OH_2)_2]$; total charge: +2). Spin densities on Ru are shown in italic and the Ru-Ru distances are given in Ångström. Crucial protons are highlighted with red dotted circles.

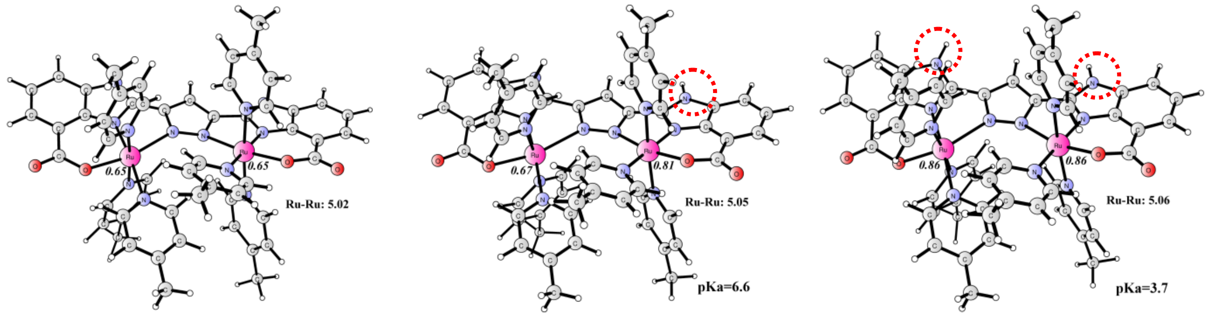


**$Ru^{III}Ru^{III}$-6pic** **$Ru^{III}Ru^{III}$-6pic-$H^+$** **$Ru^{III}Ru^{III}$-6pic-$2H^+$**

**Figure S13.** Optimized triplet structures of the $Ru_2^{III,III}$ complexes with six picoline ligands ($[Ru_2^{III,III}(pic)_6]$) in different protonation states (total charge from left to right: +1, +2 and +3, respectively) with the calculated $pK_a$ values indicated. Spin densities on Ru are shown in italic and the Ru-Ru distances are given in Ångström. Crucial protons are highlighted with red dotted circles.

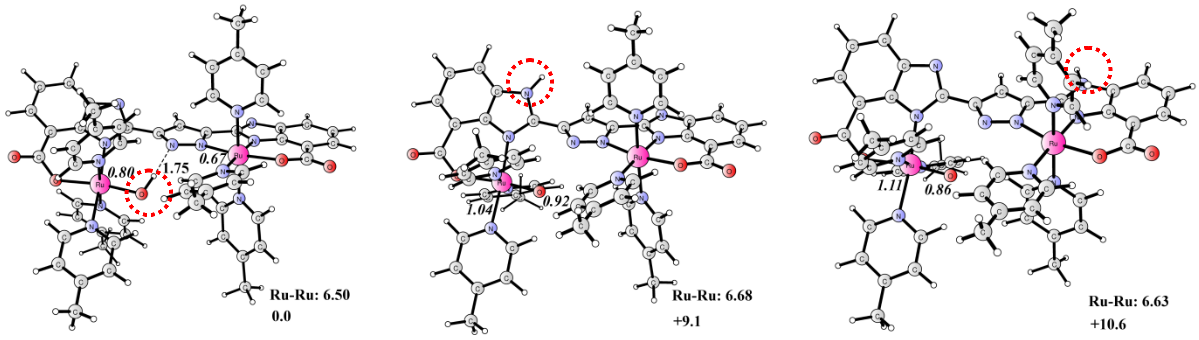


$Ru^{III}Ru^{III}$-6pic-OH-A $Ru^{II}Ru^{IV}$-6pic-OH-B $Ru^{II}Ru^{IV}$-6pic-OH-C

**Figure S14.** Optimized triplet structures of the $Ru_2^{III,III}$ complexes with six picoline ligands and a hydroxo ligand ($[(L)Ru_2^{III,III}(pic)_6(OH)]$; charge: +0). Spin densities are shown in italic and the distances are given in Ångström. The relative energies are given in kcal mol$^{-1}$. Crucial protons are highlighted with red dotted circles.

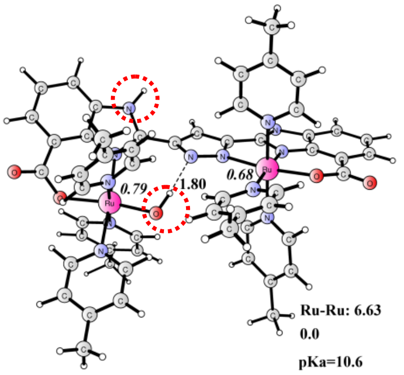


Ru$^{III}$Ru$^{III}$-6pic-OH$_2$ -A

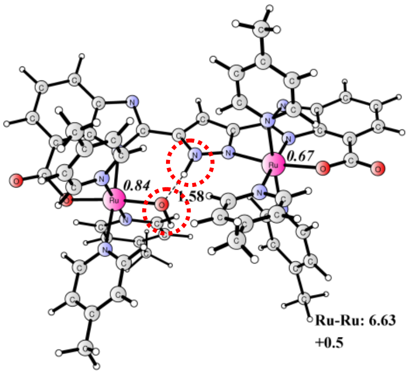


Ru$^{III}$Ru$^{III}$-6pic-OH$_2$ -B

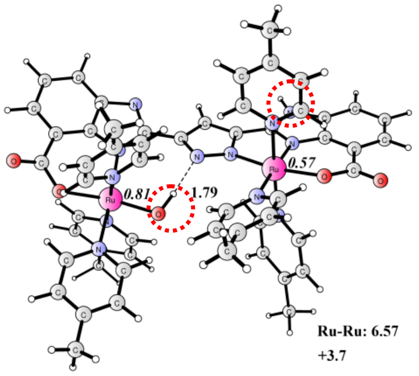


Ru$^{III}$Ru$^{III}$-6pic-OH$_2$ -C

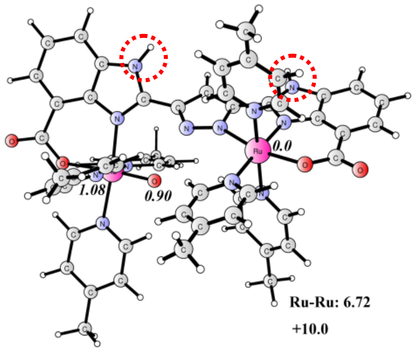


Ru$^{II}$ Ru$^{IV}$-6pic-OH$_2$ -D

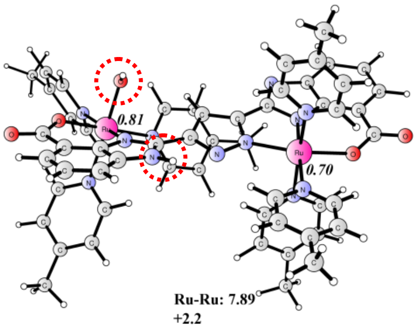


Ru$^{III}$Ru$^{III}$-6pic-OH$_2$ -E

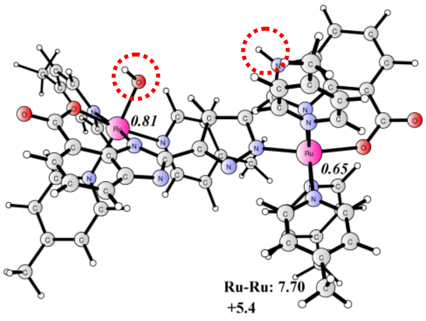


Ru$^{III}$Ru$^{III}$-6pic-OH$_2$ -F

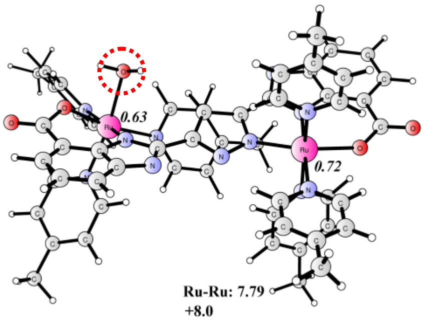


Ru$^{III}$Ru$^{III}$-6pic-OH$_2$ -G

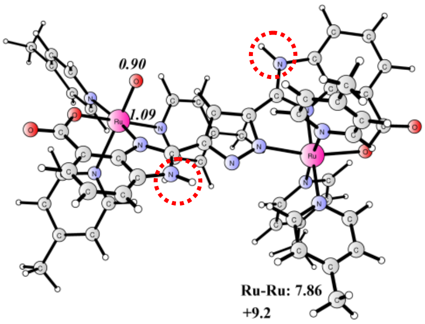


Ru$^{II}$ Ru$^{IV}$-6pic-OH$_2$ -H

**Figure S15.** Optimized triplet structures of the $Ru_2^{III,III}$ complexes with six picoline ligands and an aqua ligand ([(L)$Ru_2^{III,III}$(pic)$_6$(OH$_2$)]; charge: +1). The calculated p$K_a$ value is indicated for the isomer

with the lowest energy. Spin densities are shown in italic and the distances are given in Ångström. The relative energies are given in kcal mol$^{-1}$. Crucial protons are highlighted with red dotted circles.

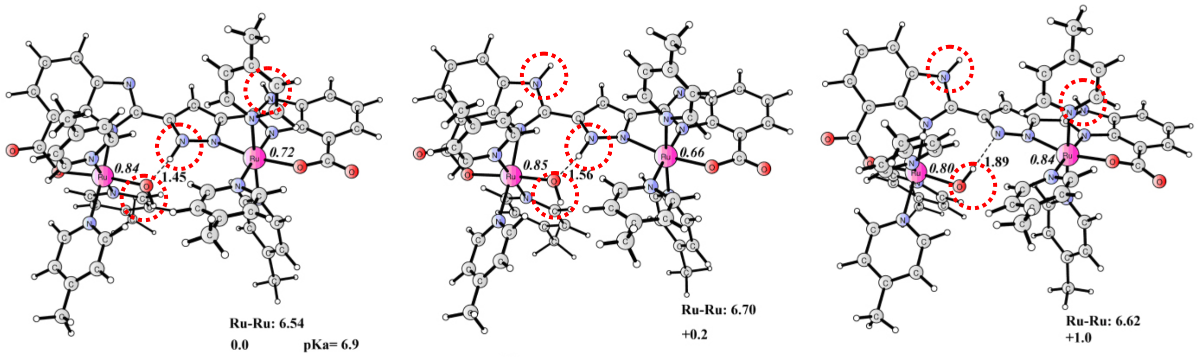


Ru$^{III}$Ru$^{III}$-6pic-H$^{+}$-OH$_2$-A Ru$^{III}$Ru$^{III}$-6pic-H$^{+}$-OH$_2$-B Ru$^{III}$Ru$^{III}$-6pic-H$^{+}$-OH$_2$-C

**Figure S16.** Optimized triplet structures of the protonated $Ru_2^{III,III}$ complexes with six picoline ligands and an aqua ligand ($[(HL)Ru_2^{III,III}(pic)_6(OH_2)]$; charge: +2). The calculated p$K_a$ value is indicated for the isomer with the lowest energy. Spin densities are shown in italic and the distances are given in Ångström. The relative energies are given in kcal mol$^{-1}$. Crucial protons are highlighted with red dotted circles.

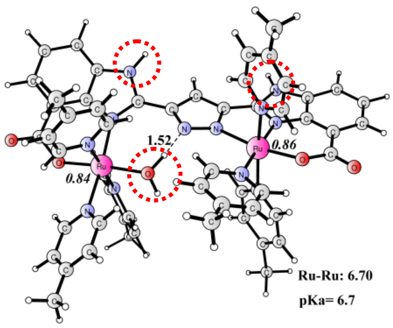


Ru$^{III}$Ru$^{III}$-6pic-2H$^{+}$-OH$_2$

**Figure S17.** Optimized triplet structure of the diprotonated $Ru_2^{III,III}$ complex with six picoline ligands and an aqua ligand ($[(H_2L)Ru_2^{III,III}(pic)_6(OH_2)]$; charge: +3). The calculated pKa is indicated. Spin densities are shown in italic, and the distances are given in Ångström. Crucial protons are highlighted with red dotted circles.

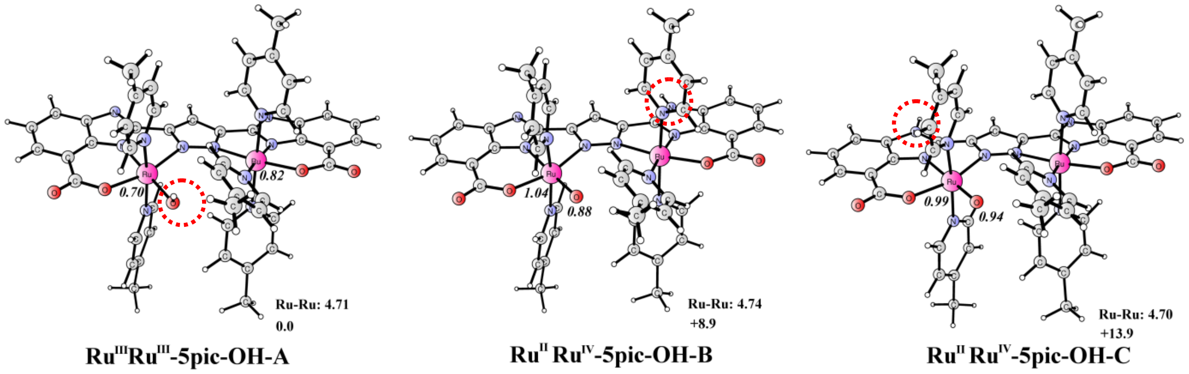


**Figure S18.** Optimized triplet structure of the $Ru_2^{III,III}$ complexes with five picoline ligands and a hydroxo ligand ($[(L)Ru_2^{III,III}(pic)_5(OH)]$; charge: +0). Spin densities are shown in italic and the distances are given in Ångström. The relative energies are given in kcal mol$^{-1}$. Crucial protons are highlighted with red dotted circles.

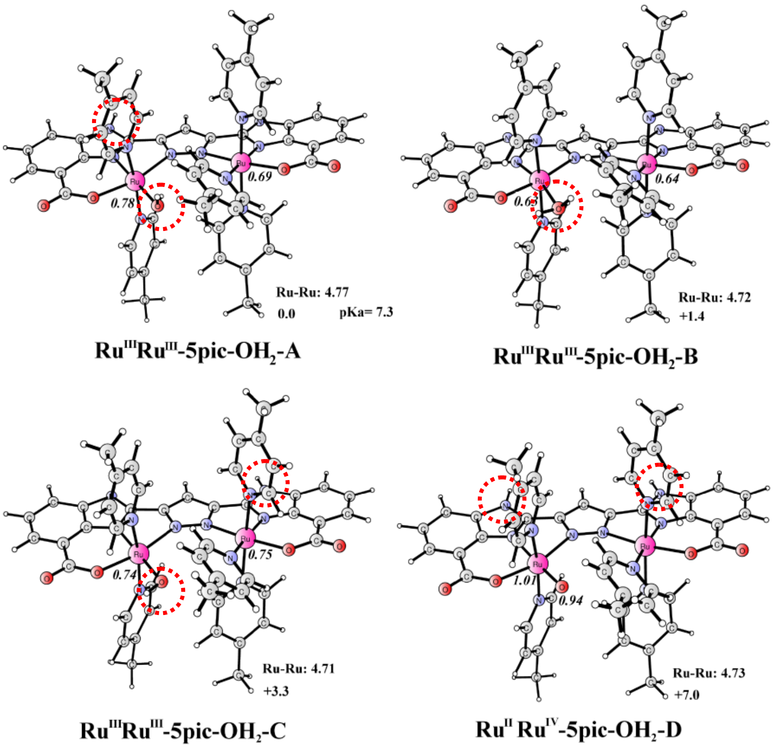


**Figure S19.** Optimized triplet structure of the $Ru_2^{III,III}$ complexes with five picoline ligands and an aqua ligand ($[(L)Ru_2^{III,III}(pic)_5(OH_2)]$; charge: +1). The calculated pKa is indicated. Spin densities are shown in italic and the distances are given in Ångström. The relative energies are given in kcal mol$^{-1}$. Crucial protons are highlighted with red dotted circles.

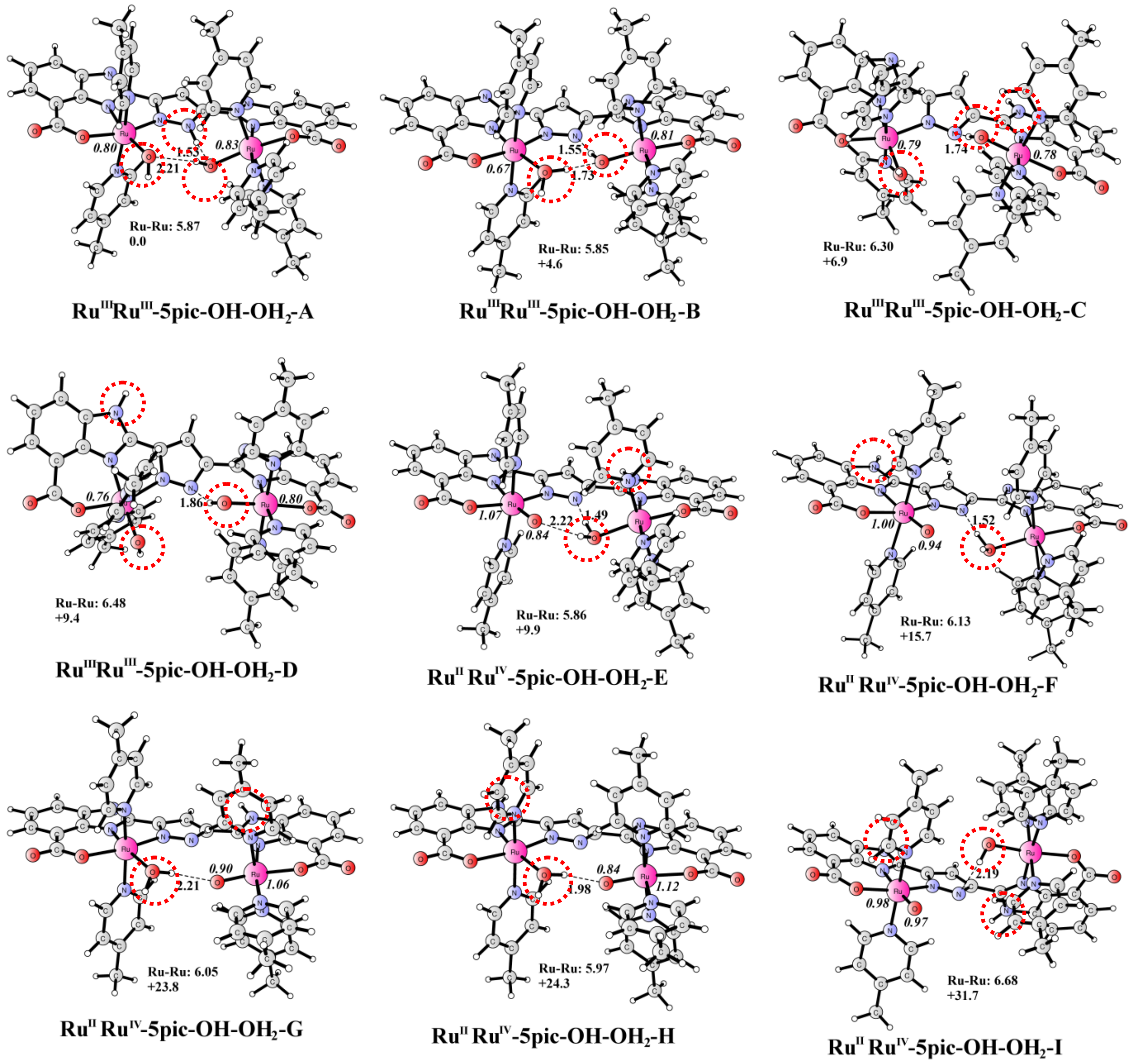


**Figure S20.** Optimized triplet structure of the $Ru_2^{III,III}$ complexes with five picoline ligands, an aqua ligand and a hydroxo ligand ($[(L)Ru_2^{III,III}(pic)_5(OH)(OH_2)]$; charge: +0). Spin densities are shown in italic and the distances are given in Ångström. The relative energies are given in kcal mol$^{-1}$. Crucial protons are highlighted with red dotted circles.

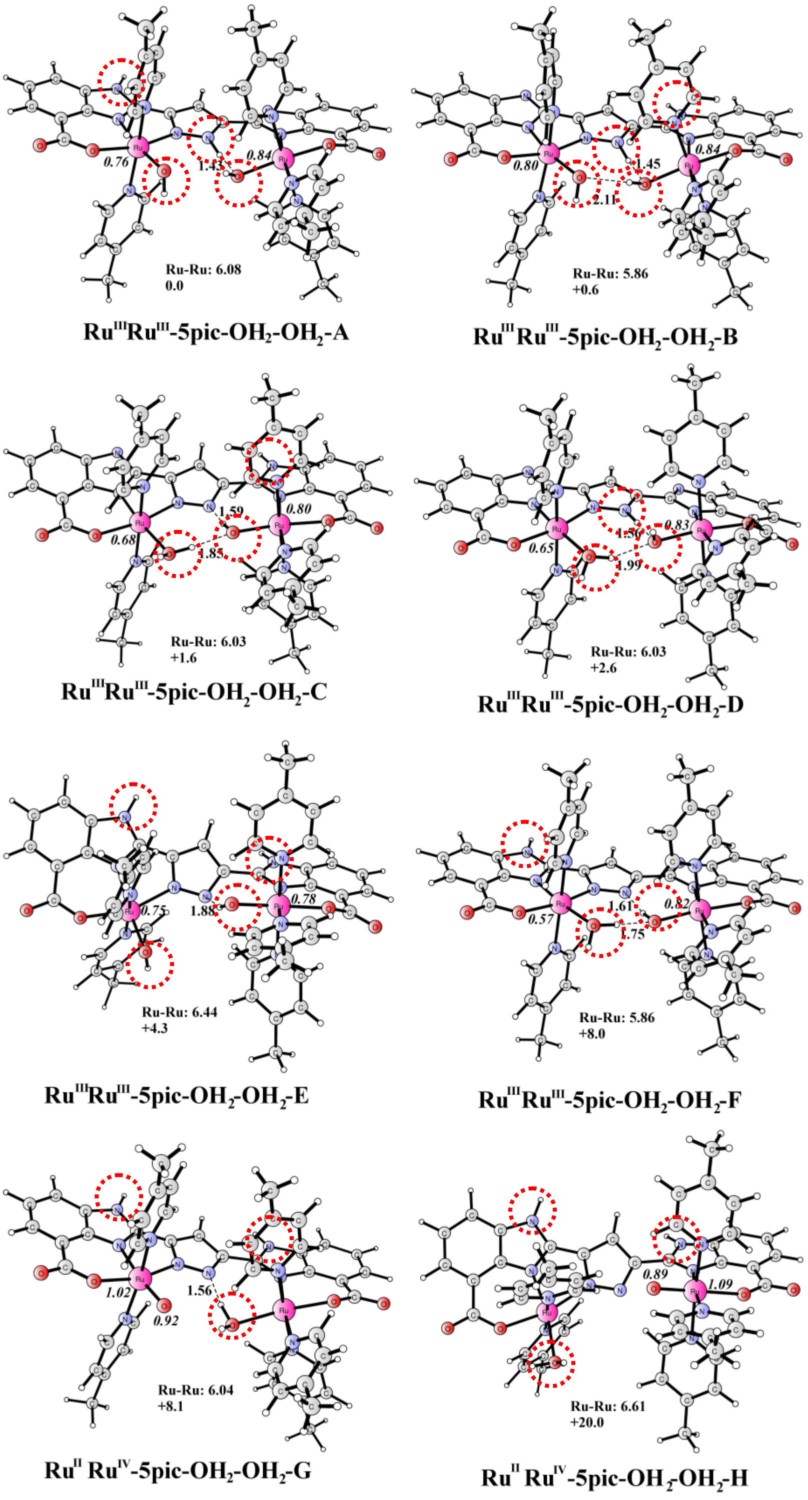


**Figure S21.** Optimized triplet structure of the $Ru_2^{III,III}$ complexes with five picoline ligands and two aqua ligands ($[(L)Ru_2^{III,III}(pic)_5(OH_2)_2]$; charge: +1). Spin densities are shown in italic and the distances are given in Ångström. The relative energies are given in kcal $mol^{-1}$. Crucial protons are highlighted with red dotted circles.

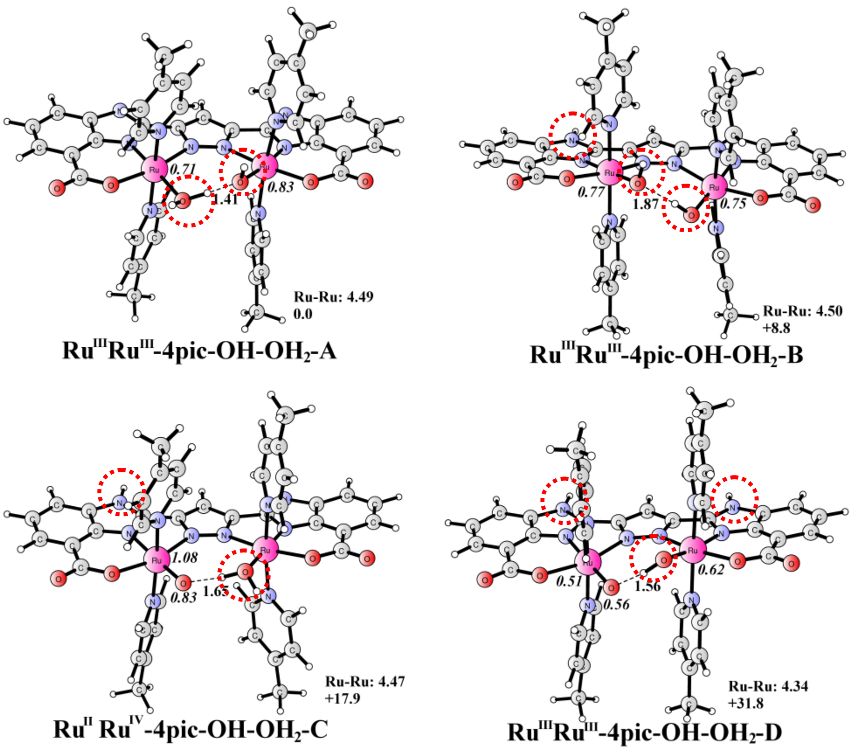


**Figure S22.** Optimized triplet structure of the $Ru_2^{III,III}$ complex with four picoline ligands, an aqua ligand and a hydroxo ligand ($[(L)Ru_2^{III,III}(pic)_4(OH)(OH_2)]$; charge: 0). Spin densities are shown in italic and the distances are given in Ångström. The relative energies are given in kcal $mol^{-1}$. Crucial protons are highlighted with red dotted circles.

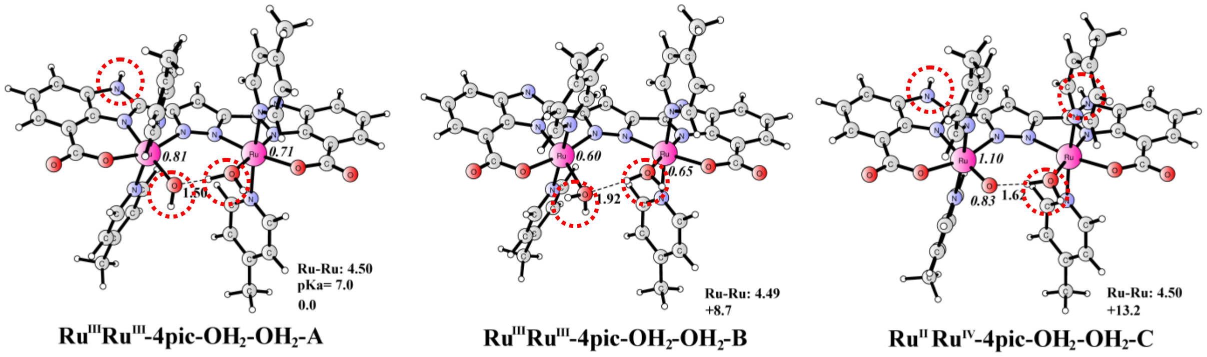


**Figure S23.** Optimized triplet structure of the $Ru_2^{III,III}$ complex with four picoline ligands and two aqua ligands ($[(L)Ru_2^{III,III}(pic)_4(OH_2)_2]$; charge: +1). Spin densities are shown in italic, the distances

are given in Ångström and the $pK_a$ value is also indicated. The relative energies are given in kcal $mol^{-1}$. Crucial protons are highlighted with red dotted circles.

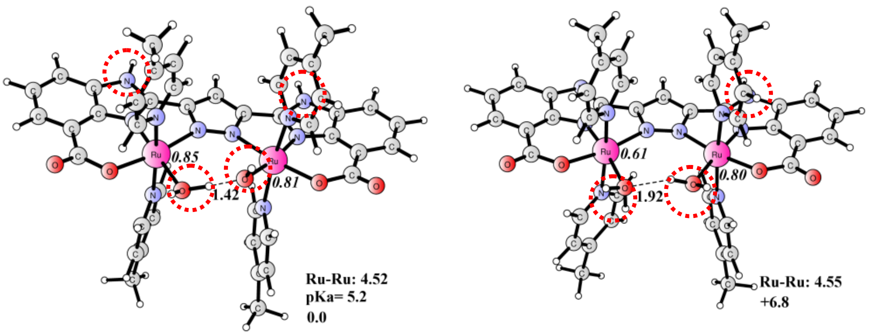


**$Ru^{III}Ru^{III}$-4pic-$H^+$-$OH_2$-$OH_2$-A** **$Ru^{III}Ru^{III}$-4pic-$H^+$-$OH_2$-$OH_2$-B**

**Figure S24.** Optimized triplet structure of the protonated $Ru_2^{III,III}$ complex with four picoline ligands and two aqua ligands ($[(HL)Ru_2^{III,III}(pic)_4(OH_2)_2]$; charge: +2). Spin densities are shown in italic, the distances are given in Ångström and the $pK_a$ value is also indicated. The relative energies are given in kcal $mol^{-1}$. Crucial protons are highlighted with red dotted circles.

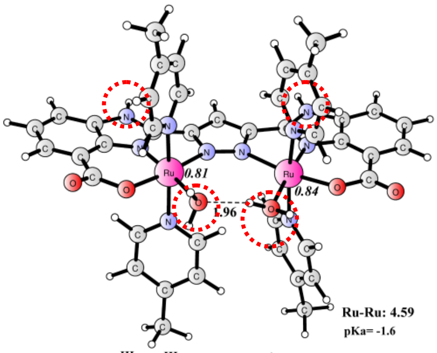


**$Ru^{III}Ru^{III}$-4pic-$2H^+$-$OH_2$-$OH_2$**

**Figure S25.** Optimized triplet structure of the diprotonated $Ru_2^{III,III}$ complex with four picoline ligands and two aqua ligands ($[(H_2L)Ru_2^{III,III}(pic)_4(OH_2)_2]$; charge: +3). Spin densities are shown in italic, the distances are given in Ångström and the $pK_a$ value is also indicated. Crucial protons are highlighted with red dotted circles.

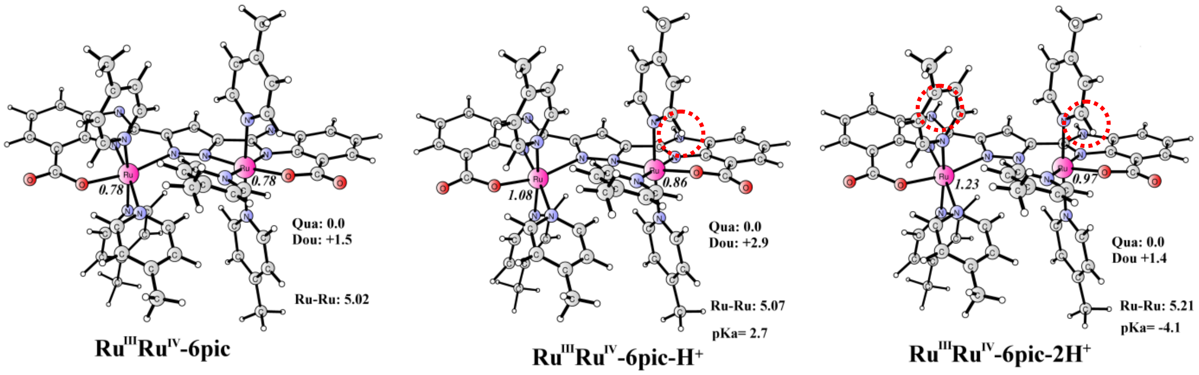


**Figure S26.** Optimized structures of the $Ru_2^{III,IV}$ complexes with six picoline ligands ($[Ru_2^{III,IV}(pic)_6]$) in different protonation states (total charge from left to right: +2, +3 and +4, respectively). The calculated $pK_a$ values are indicated. For the spin state with lower energy, spin densities on Ru are shown in italic, and the Ru-Ru distances are given in Ångström. The relative energies of two different spin states (Quartet and Doublet) are also shown. Crucial protons are highlighted with red dotted circles.

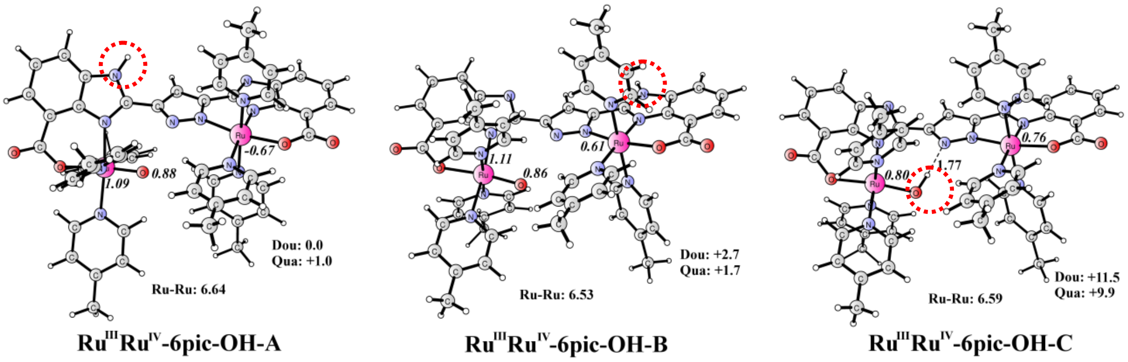


**Figure S27.** Optimized structures of the $Ru_2^{III,IV}$ complexes with six picoline ligands and a hydroxo ligand ($[(L)Ru_2^{III,IV}(pic)_6(OH)]$; total charge: +1). For the spin state with lower energy, spin densities on Ru are shown in italic. The Ru-Ru distances are given in Ångström and the relative energies of two different spin states (Quartet and Doublet) are shown. Crucial protons are highlighted with red dotted circles.

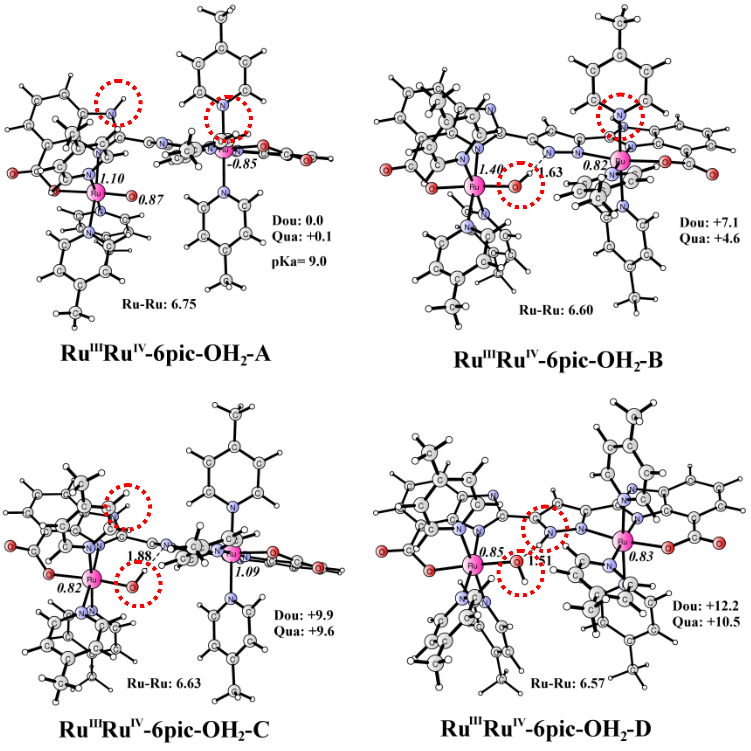


**Figure S28.** Optimized structures of the $Ru_2^{III,IV}$ complexes with six picoline ligands and an aqua ligand ([(L)$Ru_2^{III,IV}$(pic)$_6$($OH_2$)]; total charge: +2). For the spin state with lower energy, spin densities on Ru are shown in italic, the Ru-Ru distances are given in Ångström and the calculated p$K_a$ value is indicated. The relative energies of two different spin states (Quartet and Doublet) are shown. Crucial protons are highlighted with red dotted circles.

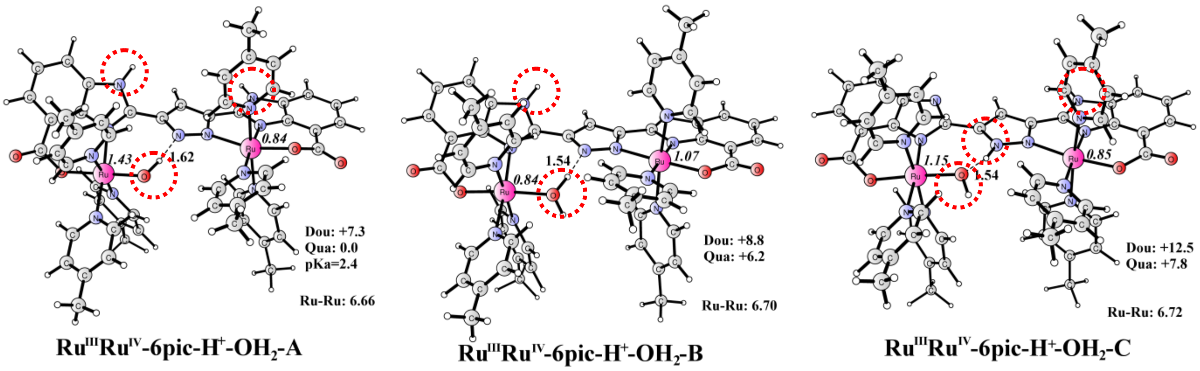


**Figure S29.** Optimized structures of the protonated $Ru_2^{III,IV}$ complexes with six picoline ligands and an aqua ligand ([(HL)$Ru_2^{III,IV}$(pic)$_6$($OH_2$)]; total charge: +3). For the spin state with lower energy, spin densities on Ru are shown in italic. The calculated p$K_a$ is indicated, the Ru-Ru distances are given in

Ångström and the relative energies of two different spin states (Quartet and Doublet) are shown. Crucial protons are highlighted with red dotted circles.

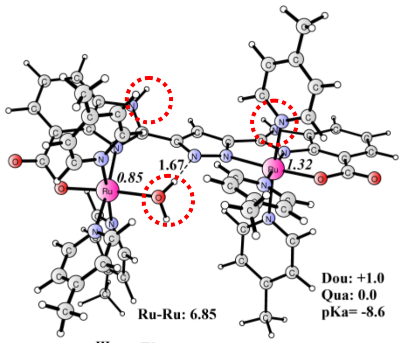


$Ru^{III}Ru^{IV}$-6pic-2H$^{+}$-$OH_2$

**Figure S30.** Optimized structures of the diprotonated $Ru_2^{III,IV}$ complex with six picoline ligands and an aqua ligand ([$(H_2L)Ru_2^{III,IV}(pic)_6(OH_2)$]; total charge: +4). For the spin state with lower energy, spin densities on Ru are shown in italic. The calculated $pK_a$ is indicated, the Ru-Ru distances are given in Ångström and the relative energies of two different spin states (Quartet and Doublet) are shown. Crucial protons are highlighted with red dotted circles.

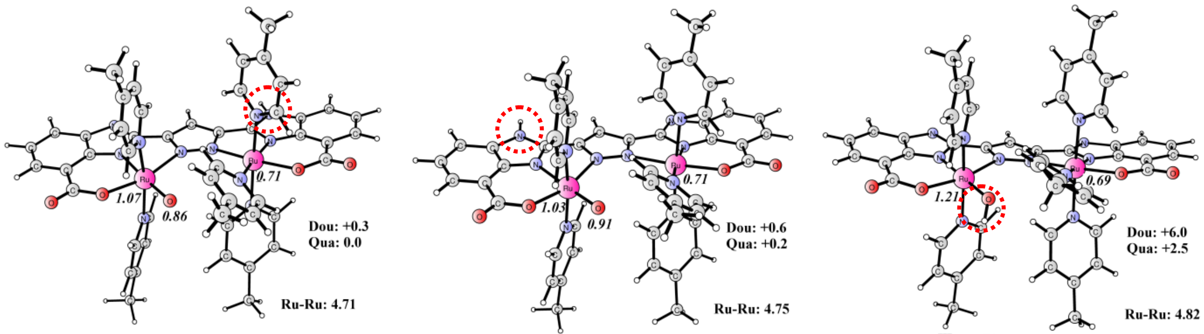


$Ru^{III}Ru^{IV}$-5pic-OH-A $Ru^{III}Ru^{IV}$-5pic-OH-B $Ru^{III}Ru^{IV}$-5pic-OH-C

**Figure S31.** Optimized structures of the $Ru_2^{III,IV}$ complexes with five picoline ligands and a hydroxo ligand ([$(L)Ru_2^{III,IV}(pic)_5(OH)$]; total charge: +1). For the spin state with lower energy, spin densities on Ru are shown in italic, the Ru-Ru distances are given in Ångström and the relative energies of two different spin states (Quartet and Doublet) are shown. Crucial protons are highlighted with red dotted circles.

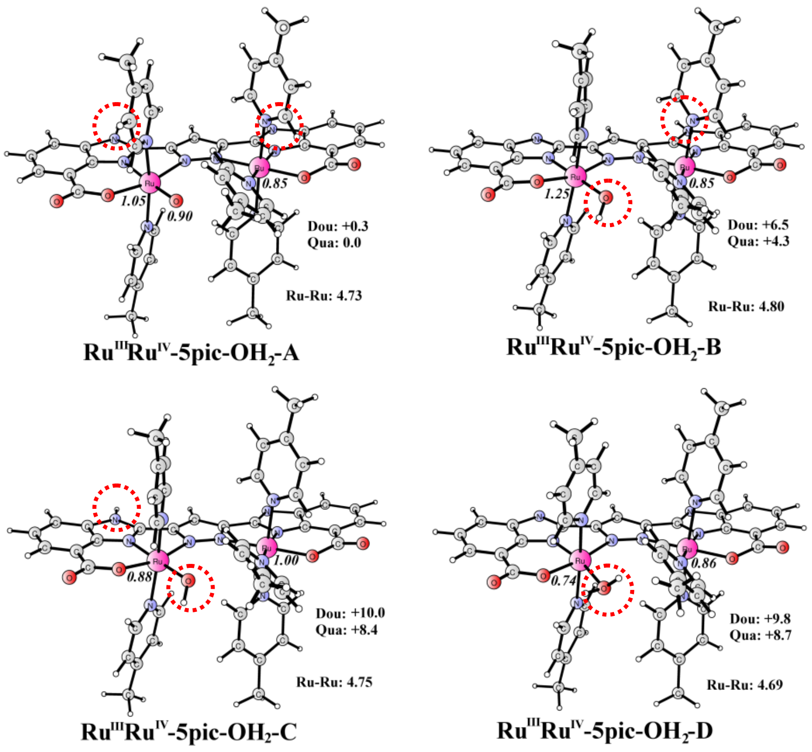


**Figure S32.** Optimized structures of the $Ru_2^{III,IV}$ complexes with five picoline ligands and an aqua ligand ($[(L)Ru_2^{III,IV}(pic)_5(OH_2)]$; total charge: +2). For the spin state with lower energy, spin densities on Ru are shown in italic. The calculated $pK_a$ is indicated, the Ru-Ru distances are given in Ångström and the relative energies of two different spin states (Quartet and Doublet) are shown. Crucial protons are highlighted with red dotted circles.

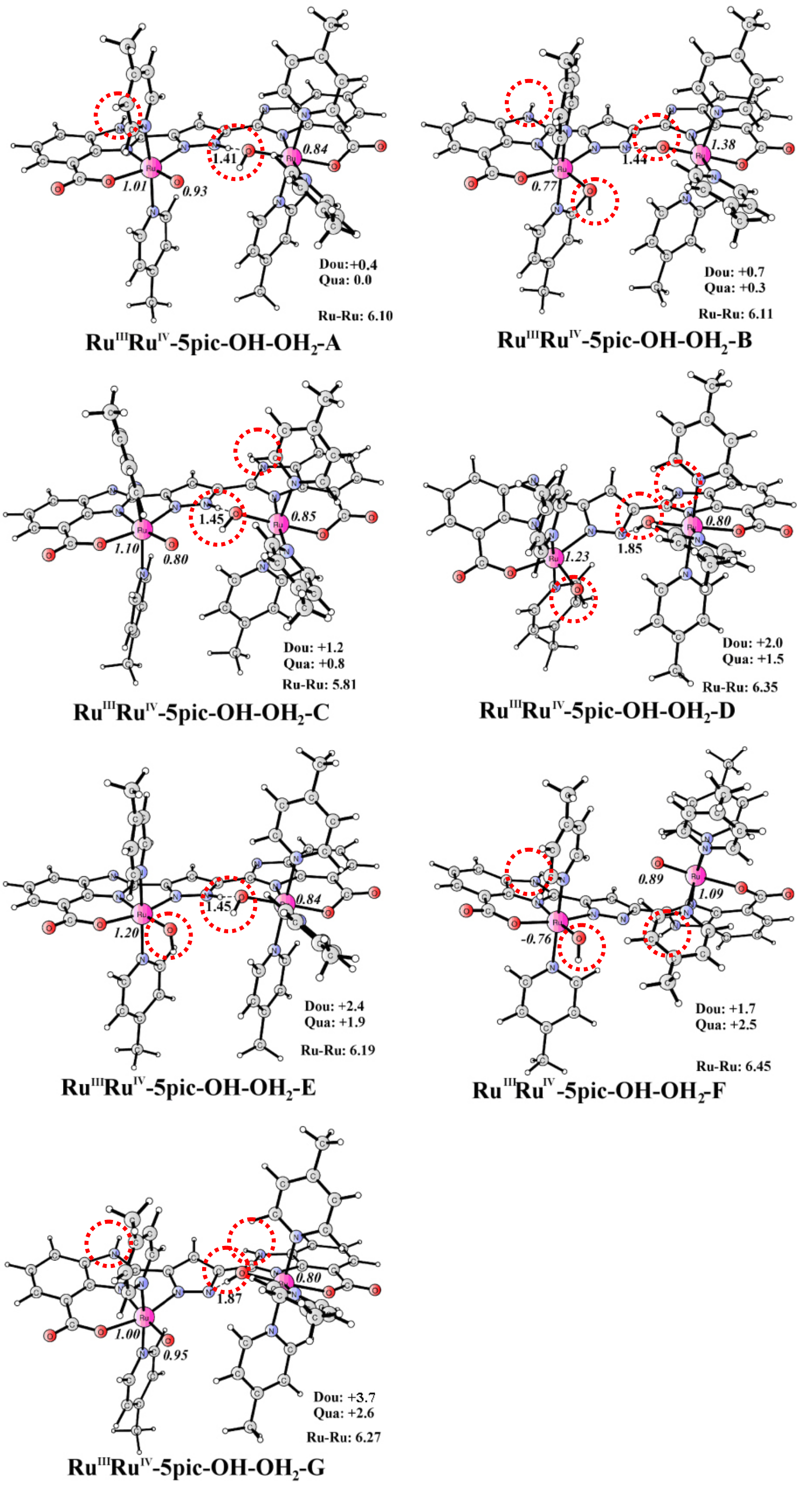


**Figure S33.** Optimized structures of the $Ru_2^{III,IV}$ complexes with five picoline ligands, an aqua ligand and a hydroxo ligand ($[(L)Ru_2^{III,IV}(pic)_5(OH)(OH_2)]$; total charge: +1). For the spin state with lower energy, spin densities on Ru are shown in italic. The Ru-Ru distances are given in Ångström and the

relative energies of two different spin states (Quartet and Doublet) are shown. Crucial protons are highlighted with red dotted circles.

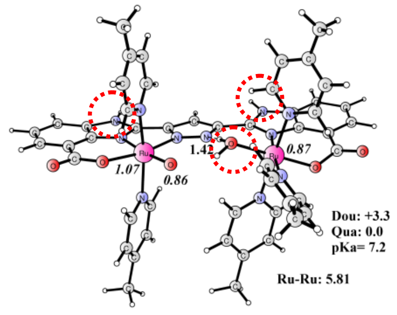


**$Ru^{III}Ru^{IV}$-5pic-2$OH_2$-A**

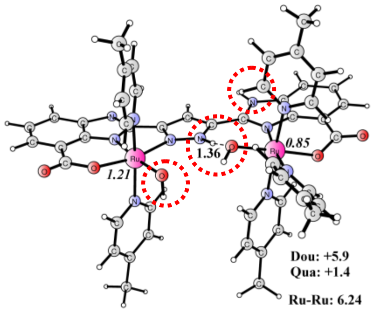


**$Ru^{III}Ru^{IV}$-5pic-2$OH_2$-B**

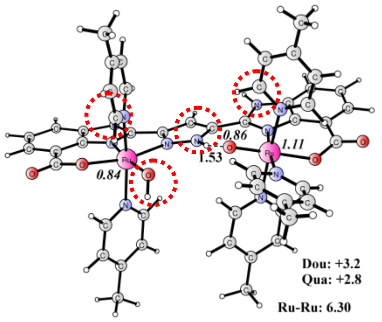


**$Ru^{III}Ru^{IV}$-5pic-2$OH_2$-C**

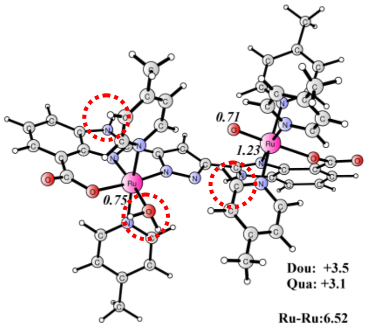


**$Ru^{III}Ru^{IV}$-5pic-2$OH_2$-D**

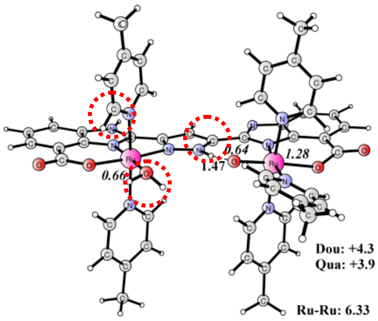


**$Ru^{III}Ru^{IV}$-5pic-2$OH_2$-E**

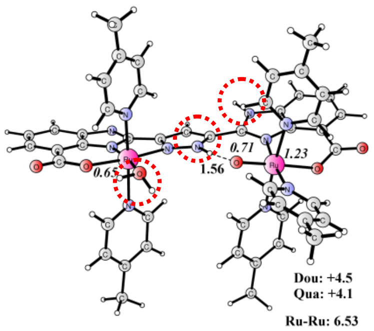


**$Ru^{III}Ru^{IV}$-5pic-2$OH_2$-F**

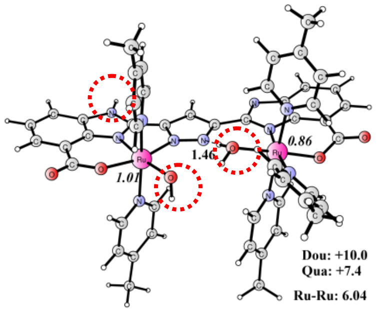


**$Ru^{III}Ru^{IV}$-5pic-2$OH_2$-G**

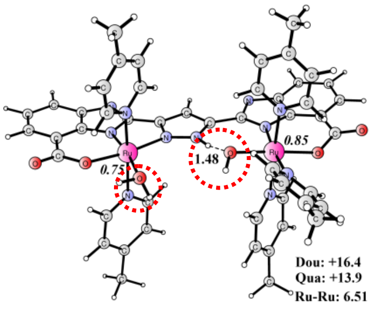


**$Ru^{III}Ru^{IV}$-5pic-2$OH_2$-H**

**Figure S34.** Optimized structures of the $Ru_2^{III,IV}$ complexes with five picoline ligands and two aqua ligands ($[(L)Ru_2^{III,IV}(pic)_5(OH_2)_2]$; total charge: +2). For the spin state with lower energy, spin densities on Ru are shown in italic. The calculated $pK_a$ is indicated, the Ru-Ru distances are given in Ångström and the relative energies of two different spin states (Quartet and Doublet) are shown. Crucial protons are highlighted with red dotted circles.

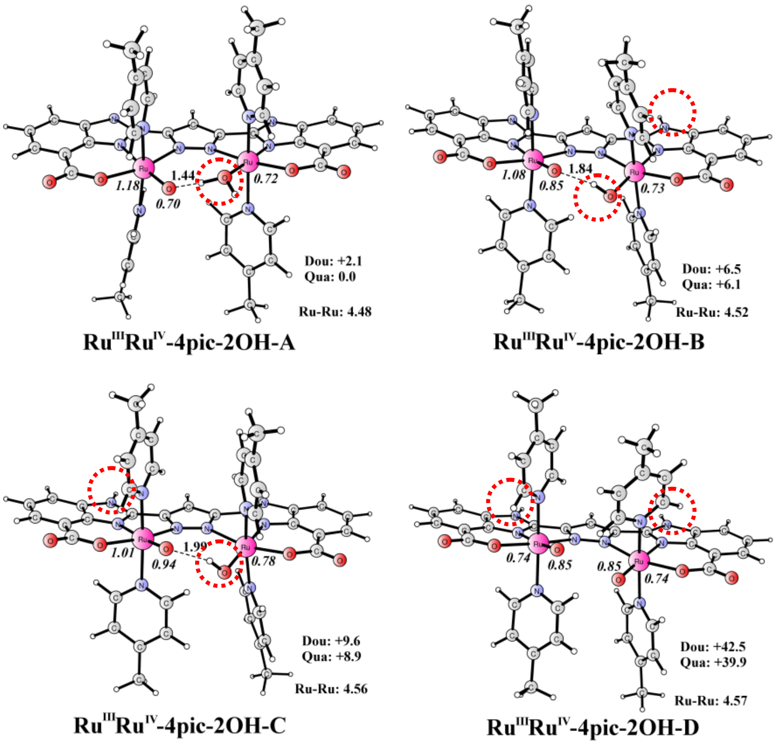


**Figure S35.** Optimized structures of the $Ru_2^{III,IV}$ complexes with four picoline ligands and two hydroxo ligands ($[(L)Ru_2^{III,IV}(pic)_4(OH)_2]$; total charge: 0). For the spin state with lower energy, spin densities on Ru are shown in italic. The Ru-Ru distances are given in Ångström and the relative energies of two different spin states (Quartet and Doublet) are shown. Crucial protons are highlighted with red dotted circles.

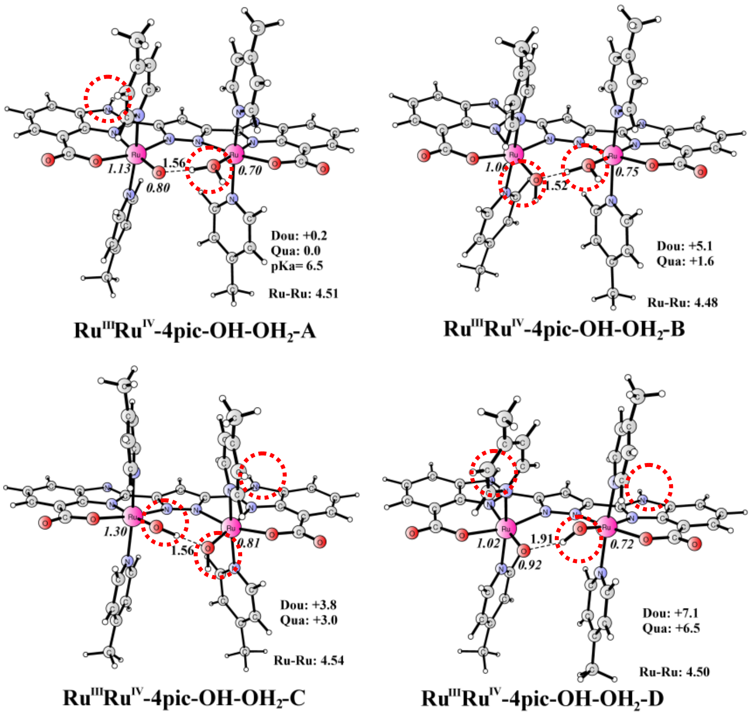


**Figure S36.** Optimized structures of the $Ru_2^{III,IV}$ complexes with four picoline ligands, a hydroxo ligand and an aqua ligand ($[(L)Ru_2^{III,IV}(pic)_4(OH)(OH_2)]$; total charge: +1). For the spin state with lower energy, spin densities on Ru are shown in italic. The calculated $pK_a$ is indicated, the Ru-Ru distances are given in Ångström and the relative energies of two different spin states (Quartet and Doublet) are shown. Crucial protons are highlighted with red dotted circles.

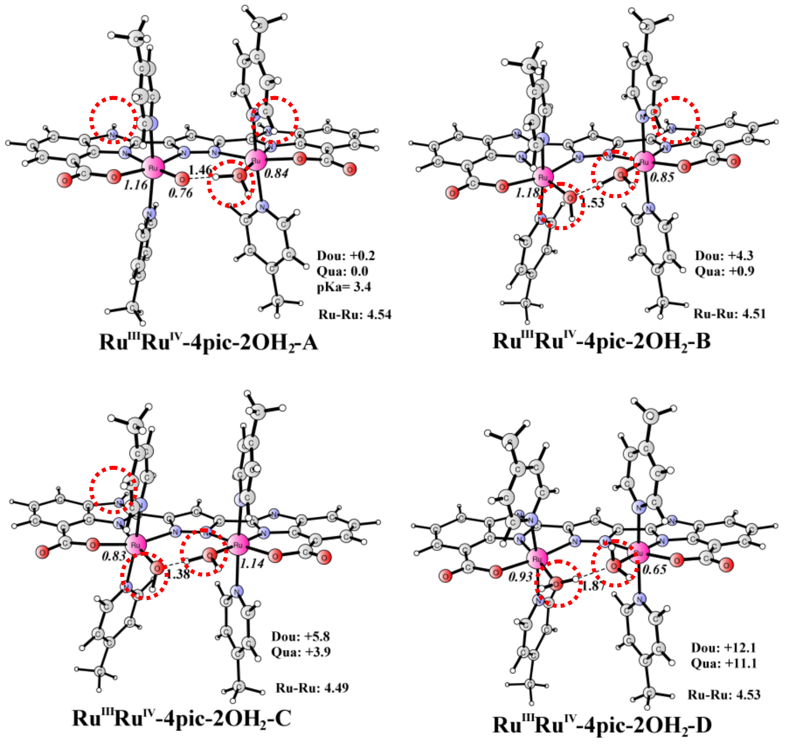


**Figure S37.** Optimized structures of the $Ru_2^{III,IV}$ complexes with four picoline ligands and two aqua ligands ([(L)$Ru_2^{III,IV}$(pic)$_4$($OH_2$)$_2$]; total charge: +2). For the spin state with lower energy, spin densities on Ru are shown in italic. The calculated $pK_a$ is indicated, the Ru-Ru distances are given in Ångström and the relative energies of two different spin states (Quartet and Doublet) are shown. Crucial protons are highlighted with red dotted circles.

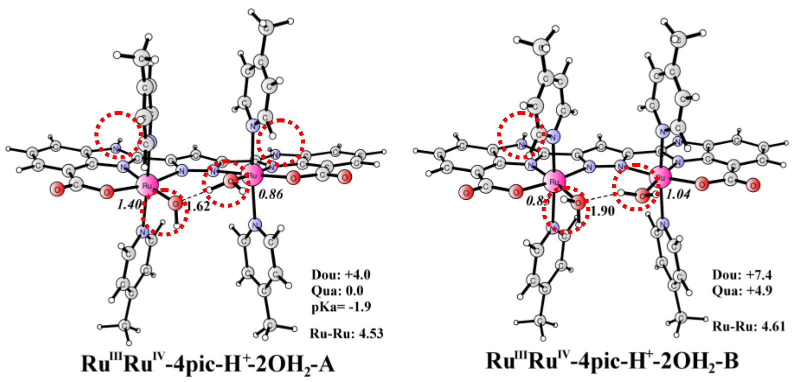


**Figure S38.** Optimized structures of the protonated $Ru_2^{III,IV}$ complexes with four picoline ligands and two aqua ligands ([(HL)$Ru_2^{III,IV}$(pic)$_4$($OH_2$)$_2$]; total charge: +3). For the spin state with lower energy, spin densities on Ru are shown in italic. The calculated $pK_a$ is indicated, the Ru-Ru distances are given

in Ångström and the relative energies of two different spin states (Quartet and Doublet) are shown. Crucial protons are highlighted with red dotted circles.

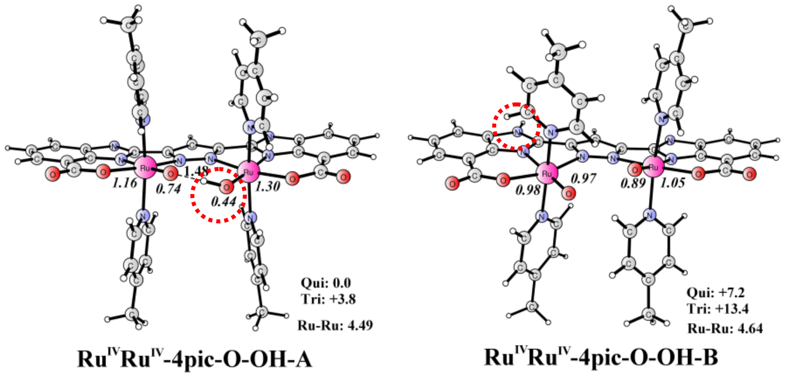


**Figure S39.** Optimized structures of the $Ru_2^{IV,IV}$ complexes with four picoline ligands, a hydroxo ligand and an oxo ligand ($[(L)Ru_2^{IV,IV}(pic)_4(O)(OH)]$; total charge: 0). For the spin state with lower energy, the spin densities on Ru and selected oxygen atoms are shown in italic. The Ru-Ru distances are given in Ångström and the relative energies of two different spin states (Triplet and Quintet) are shown. Crucial protons are highlighted with red dotted circles.

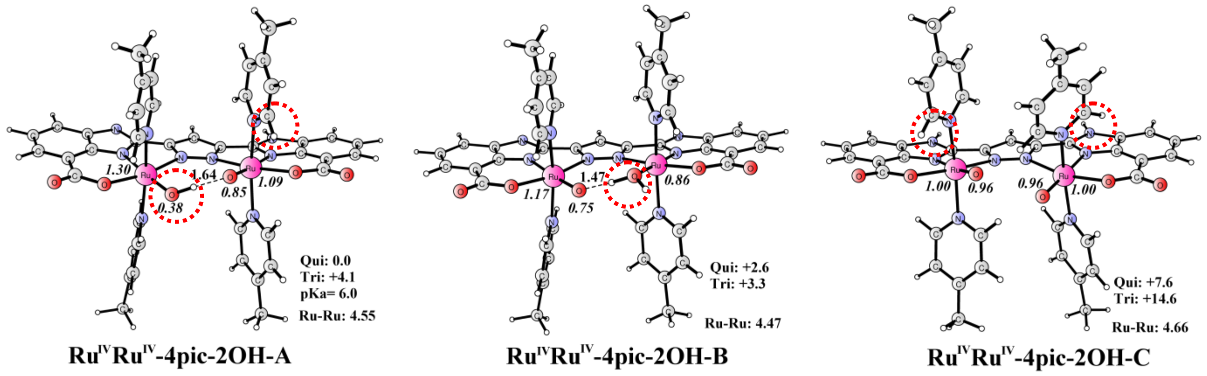


**Figure S40.** Optimized structures of the $Ru_2^{IV,IV}$ complexes with four picoline ligands and two hydroxo ligands ($[(L)Ru_2^{IV,IV}(pic)_4(OH)_2]$; total charge: +1). For the spin state with lower energy, the spin densities on Ru and selected oxygen atoms are shown in italic. The calculated $pK_a$ is indicated, the Ru-Ru distances are given in Ångström and the relative energies of two different spin states (Triplet and Quintet) are shown. Crucial protons are highlighted with red dotted circles.

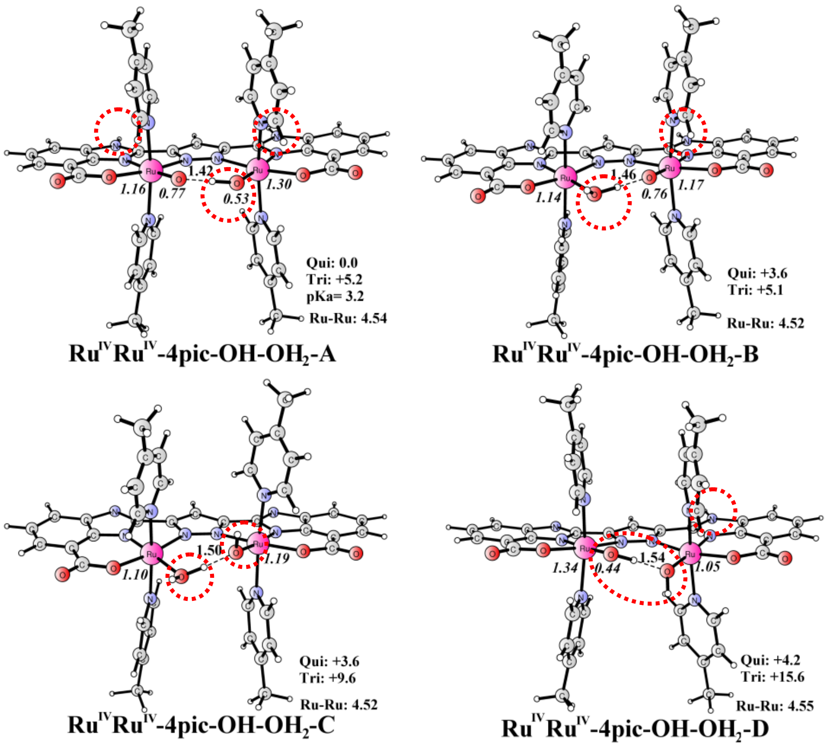


**Figure S41.** Optimized structures of the $Ru_2^{IV,IV}$ complexes with four picoline ligands, an aqua ligand and a hydroxo ligand ($[(L)Ru_2^{IV,IV}(pic)_4(OH)(OH_2)]$; total charge: +2). For the spin state with lower energy, spin densities on Ru are shown in italic. The calculated pKa is indicated, the Ru-Ru distances are given in Ångström and the relative energies of two different spin states (Triplet and Quintet) are shown. Crucial protons are highlighted with red dotted circles.

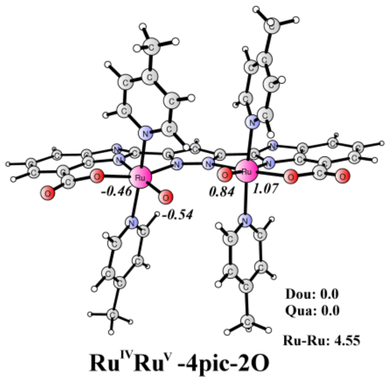


**Figure S42.** Optimized structures of the $Ru_2^{IV,V}$ complex with four picoline ligands and two oxo ligands ($[(L)Ru_2^{IV,V}(pic)_4(O)_2]$; total charge: 0). For the spin state with lower energy, spin densities on

Ru are shown in italic. The Ru-Ru distances are given in Ångström and the relative energies of three different spin states (Doublet and Quartet) are shown. Crucial protons are highlighted with red dotted circles.

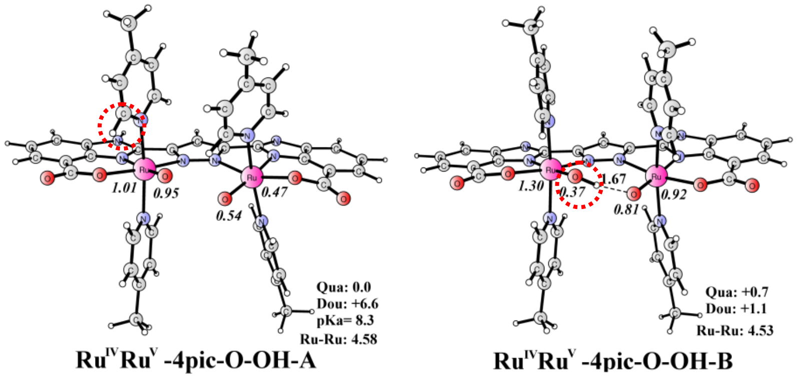


**Figure S43.** Optimized structures of the $Ru_2^{IV,V}$ complexes with four picoline ligands, a hydroxo ligand and an oxo ligand ($[(L)Ru_2^{IV,V}(pic)_4(O)(OH)]$; total charge: +1). For the spin state with lower energy, spin densities on Ru are shown in italic. The calculated $pK_a$ is indicated, the Ru-Ru distances are given in Ångström and the relative energies of three different spin states (Doublet and Quartet) are shown. Crucial protons are highlighted with red dotted circles.

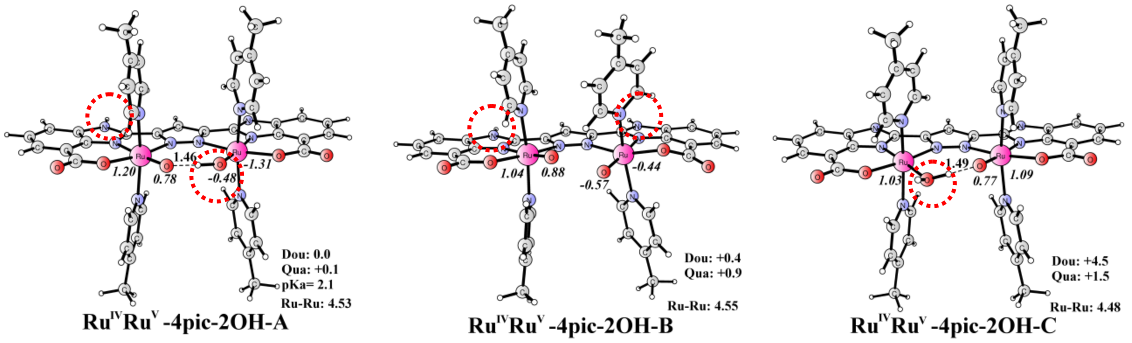


**Figure S44.** Optimized structures of $Ru_2^{IV,V}$ complexes with four picoline ligands and two hydroxo ligands ($[(L)Ru_2^{IV,V}(pic)_4(OH)_2]$; total charge: +2). For the spin state with lower energy, spin densities on Ru are shown in italic. The calculated $pK_a$ is indicated, the Ru-Ru distances are given in Ångström and the relative energies of three different spin states (Doublet and Quartet) are shown. Crucial protons are highlighted with red dotted circles.

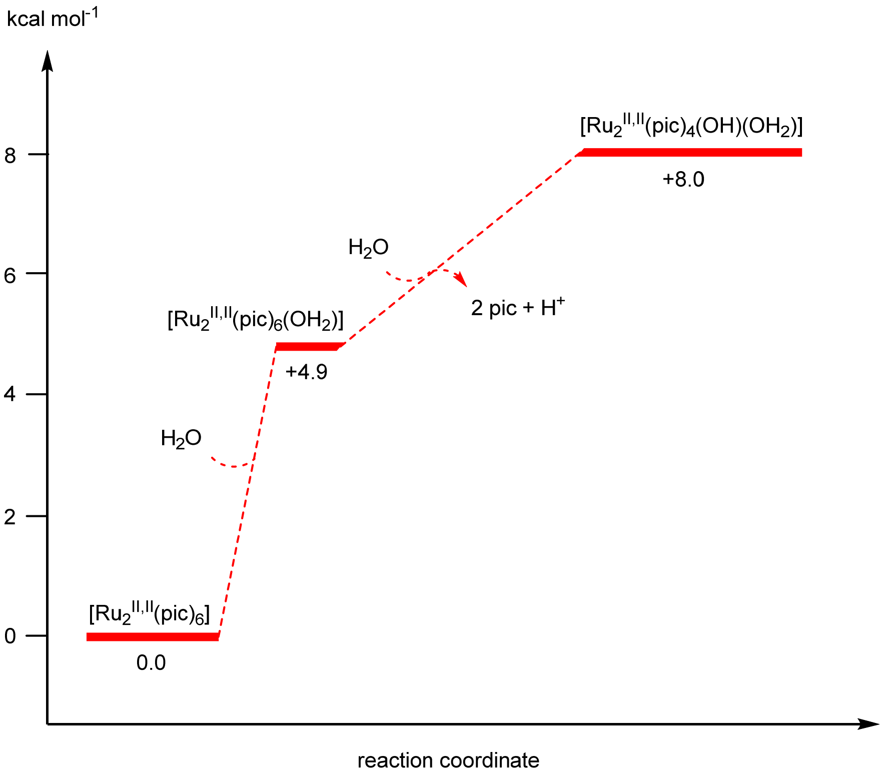


**Figure S45.** Energy diagram for the ligand exchange at the $Ru_2^{II,II}$ state for Ru complex **1** at pH 7.2. The energy for the structure with the lowest energy is set to zero.

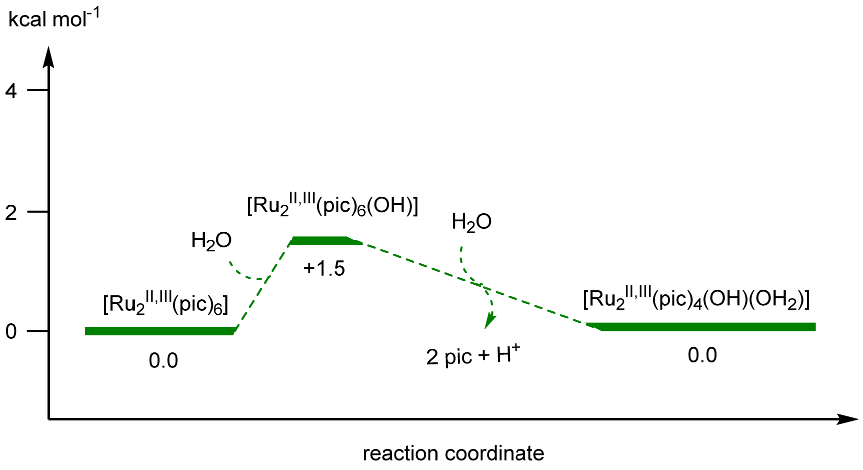


**Figure S46.** Energy diagram for the ligand exchange at the $Ru_2^{II,III}$ state for Ru complex **1** at pH 7.2. The energy for the structure with the lowest energy is set to zero.

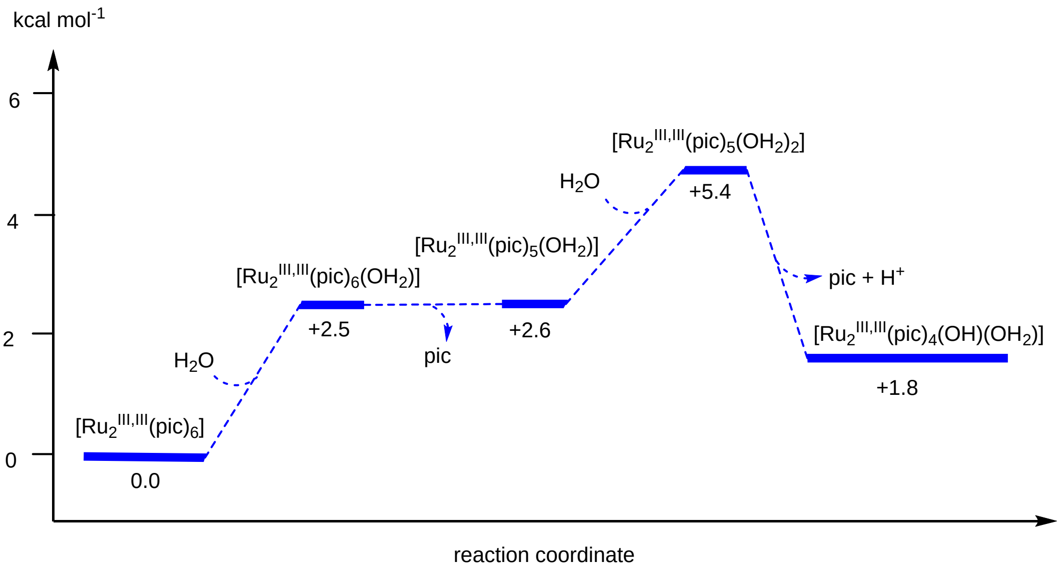


**Figure S47.** Energy diagram for the ligand exchange at the $Ru_2^{III,III}$ state for Ru complex **1** at pH 7.2. The energy for the structure with the lowest energy is set to zero.

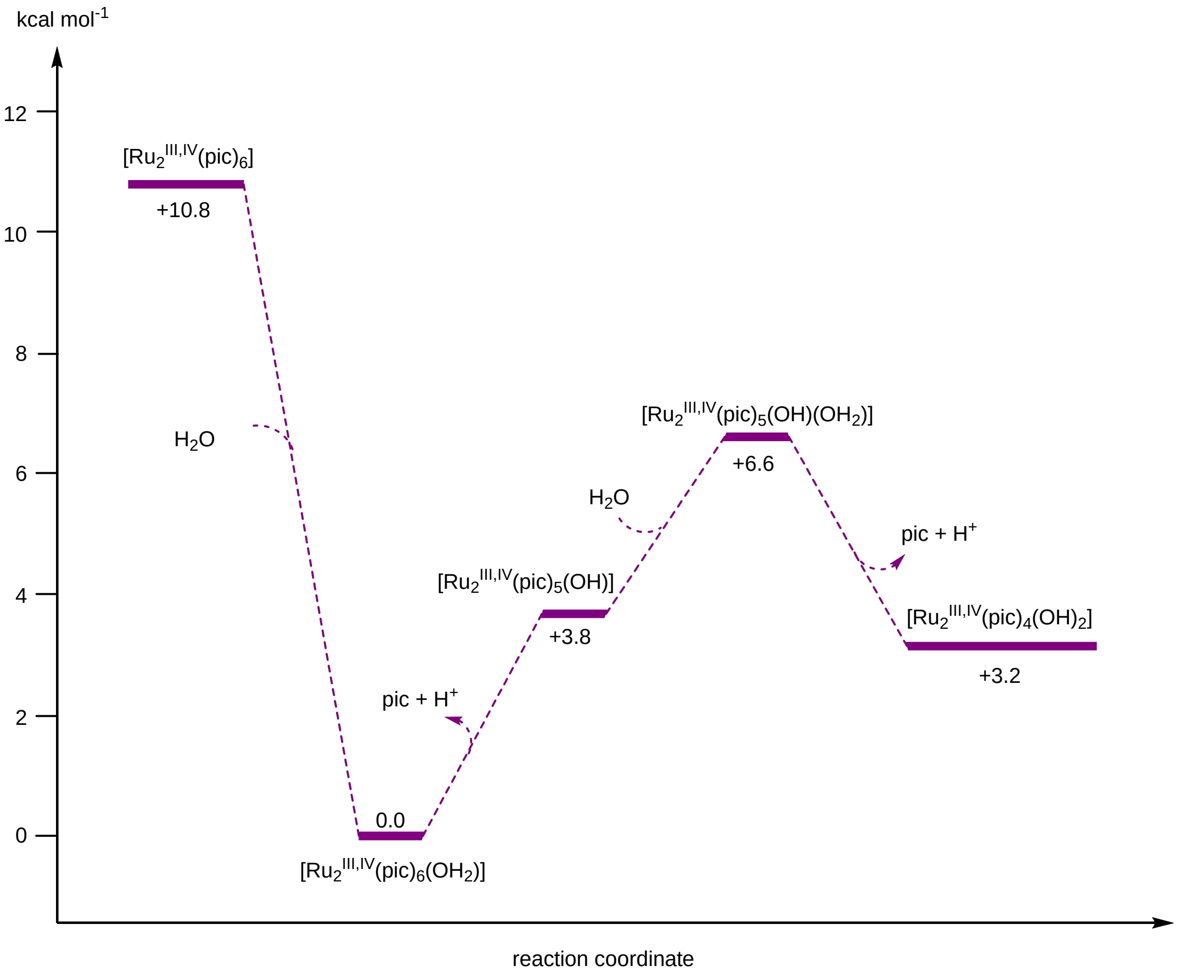

**Figure S48.** Energy diagram for the ligand exchange at the $Ru_2^{III,IV}$ state for Ru complex **1** at pH 7.2. The energy for the structure with the lowest energy is set to zero.

**Table S1.** Cartesian coordinates for $Ru^{II}Ru^{II}$-6pic.

| Center number | Atomic number | Atomic type | Coordinates (Ångström) | | |
|---|---|---|---|---|---|
| | | | X | Y | Z |
| 1 | 6 | 0 | -4.440178 | 0.684709 | -2.133953 |
| 2 | 6 | 0 | -4.403653 | 0.894337 | -3.546155 |
| 3 | 6 | 0 | -5.622834 | 1.116108 | -4.205584 |
| 4 | 6 | 0 | -6.793488 | 1.100472 | -3.44245 |
| 5 | 6 | 0 | -6.792453 | 0.861451 | -2.050271 |
| 6 | 6 | 0 | -5.603568 | 0.636568 | -1.352322 |
| 7 | 6 | 0 | -2.410695 | 0.574157 | -2.870412 |
| 8 | 6 | 0 | -5.594663 | 0.327896 | 0.127525 |
| 9 | 8 | 0 | -6.651357 | 0.370614 | 0.759159 |
| 10 | 8 | 0 | -4.46137 | -0.009908 | 0.730222 |
| 11 | 7 | 0 | -3.159982 | 0.508266 | -1.743349 |
| 12 | 7 | 0 | -3.093627 | 0.814715 | -3.996836 |
| 13 | 6 | 0 | 0.078674 | 0.124736 | -3.482754 |
| 14 | 6 | 0 | 1.148712 | -0.134458 | -2.62414 |
| 15 | 7 | 0 | -0.599893 | 0.171471 | -1.308832 |
| 16 | 7 | 0 | 0.737046 | -0.069518 | -1.30427 |
| 17 | 6 | 0 | -1.001262 | 0.32433 | -2.62522 |
| 18 | 6 | 0 | 4.605743 | -0.55751 | -2.072961 |
| 19 | 6 | 0 | 4.673027 | -0.6762 | -3.478234 |
| 20 | 6 | 0 | 5.901168 | -0.860051 | -4.110891 |
| 21 | 6 | 0 | 7.029276 | -0.904118 | -3.282818 |
| 22 | 6 | 0 | 6.948827 | -0.759592 | -1.884964 |
| 23 | 6 | 0 | 5.725506 | -0.572141 | -1.2357 |
| 24 | 6 | 0 | 2.553511 | -0.371028 | -2.813674 |
| 25 | 7 | 0 | 3.296543 | -0.394578 | -1.718849 |
| 26 | 7 | 0 | 3.350186 | -0.551106 | -3.921532 |
| 27 | 6 | 0 | 5.653223 | -0.368146 | 0.273535 |
| 28 | 8 | 0 | 6.69781 | -0.482406 | 0.914282 |
| 29 | 8 | 0 | 4.509407 | -0.058869 | 0.83963 |
| 30 | 44 | 0 | 2.528522 | 0.018366 | 0.075646 |
| 31 | 44 | 0 | -2.481992 | -0.027089 | 0.039288 |
| 32 | 6 | 0 | 3.195036 | -2.614742 | 1.452731 |
| 33 | 6 | 0 | 1.569263 | -2.888787 | -0.159439 |
| 34 | 6 | 0 | 3.178652 | -3.973936 | 1.747304 |
| 35 | 6 | 0 | 1.50281 | -4.253908 | 0.083435 |
| 36 | 6 | 0 | 2.320362 | -4.838246 | 1.059378 |
| 37 | 6 | 0 | 3.770173 | 2.763914 | 0.360912 |
| 38 | 6 | 0 | 2.100139 | 2.80217 | -1.228413 |
| 39 | 6 | 0 | 4.028966 | 4.115516 | 0.172807 |
| 40 | 6 | 0 | 2.311397 | 4.154888 | -1.470848 |
| 41 | 6 | 0 | 3.297125 | 4.853134 | -0.765405 |
| 42 | 6 | 0 | -3.169274 | 2.477806 | 1.606506 |
| 43 | 6 | 0 | -1.78301 | 2.939486 | -0.175714 |

| | | | | | |
|---|---|---|---|---|---|
| 44 | 6 | 0 | -3.258168 | 3.825105 | 1.932679 |
| 45 | 6 | 0 | -1.829906 | 4.302041 | 0.093169 |
| 46 | 6 | 0 | -2.580153 | 4.784922 | 1.170611 |
| 47 | 6 | 0 | -3.711429 | -2.783594 | 0.136382 |
| 48 | 6 | 0 | -2.126633 | -2.67841 | -1.53236 |
| 49 | 6 | 0 | -4.005518 | -4.104812 | -0.177518 |
| 50 | 6 | 0 | -2.372284 | -3.99604 | -1.901302 |
| 51 | 6 | 0 | -3.334319 | -4.751365 | -1.221139 |
| 52 | 6 | 0 | 2.678477 | 0.615151 | 3.076688 |
| 53 | 6 | 0 | 0.677877 | 1.317009 | 2.169438 |
| 54 | 6 | 0 | 2.358008 | 1.177802 | 4.307996 |
| 55 | 6 | 0 | 0.30161 | 1.917597 | 3.364098 |
| 56 | 6 | 0 | 1.144127 | 1.851214 | 4.480991 |
| 57 | 6 | 0 | -2.834856 | -0.833508 | 2.99597 |
| 58 | 6 | 0 | -0.757665 | -1.442198 | 2.212746 |
| 59 | 6 | 0 | -2.59782 | -1.463801 | 4.213865 |
| 60 | 6 | 0 | -0.458852 | -2.105978 | 3.396052 |
| 61 | 6 | 0 | -1.388058 | -2.126792 | 4.444379 |
| 62 | 7 | 0 | -2.773229 | -2.063023 | -0.519399 |
| 63 | 7 | 0 | -2.428975 | 2.016711 | 0.571625 |
| 64 | 7 | 0 | -1.927888 | -0.804139 | 1.993551 |
| 65 | 7 | 0 | 1.852037 | 0.669051 | 2.010161 |
| 66 | 7 | 0 | 2.396072 | -2.056855 | 0.515456 |
| 67 | 7 | 0 | 2.813667 | 2.096332 | -0.324872 |
| 68 | 1 | 0 | 5.990527 | -0.958257 | -5.1883 |
| 69 | 1 | 0 | 8.004049 | -1.045205 | -3.74011 |
| 70 | 1 | 0 | 7.844449 | -0.782666 | -1.273619 |
| 71 | 1 | 0 | 0.060035 | 0.160803 | -4.561862 |
| 72 | 1 | 0 | -5.655759 | 1.286197 | -5.278345 |
| 73 | 1 | 0 | -7.746031 | 1.267417 | -3.939085 |
| 74 | 1 | 0 | -7.723845 | 0.837485 | -1.493813 |
| 75 | 1 | 0 | 0.935747 | -2.416814 | -0.898857 |
| 76 | 1 | 0 | 3.88167 | -1.937612 | 1.944776 |
| 77 | 1 | 0 | 3.850845 | -4.352544 | 2.511592 |
| 78 | 1 | 0 | 0.800777 | -4.853265 | -0.488684 |
| 79 | 1 | 0 | 4.344283 | 2.166668 | 1.059159 |
| 80 | 1 | 0 | 1.334537 | 2.257253 | -1.764402 |
| 81 | 1 | 0 | 4.812604 | 4.585675 | 0.759568 |
| 82 | 1 | 0 | 1.699299 | 4.656624 | -2.214612 |
| 83 | 1 | 0 | -3.878174 | 4.121079 | 2.774 |
| 84 | 1 | 0 | -1.28203 | 4.983738 | -0.550706 |
| 85 | 1 | 0 | -1.215072 | 2.550514 | -1.010072 |
| 86 | 1 | 0 | -3.729708 | 1.729403 | 2.149999 |
| 87 | 1 | 0 | -4.252503 | -2.248679 | 0.907265 |
| 88 | 1 | 0 | -1.397264 | -2.081717 | -2.063818 |
| 89 | 1 | 0 | -4.775457 | -4.619248 | 0.390014 |
| 90 | 1 | 0 | -1.817776 | -4.421902 | -2.732823 |
| 91 | 1 | 0 | 3.638488 | 0.142836 | 2.899619 |
| 92 | 1 | 0 | 0.022619 | 1.331358 | 1.307273 |
| 93 | 1 | 0 | 3.0705 | 1.099915 | 5.124112 |
| 94 | 1 | 0 | -0.655381 | 2.427815 | 3.413443 |
| 95 | 1 | 0 | -3.790663 | -0.373053 | 2.766283 |
| 96 | 1 | 0 | -0.040957 | -1.39875 | 1.403236 |
| 97 | 1 | 0 | -3.373288 | -1.445459 | 4.974534 |
| 98 | 1 | 0 | 0.503605 | -2.599967 | 3.493126 |

| | | | | | |
|---|---|---|---|---|---|
| 99 | 6 | 0 | -2.689424 | 6.257326 | 1.470861 |
| 100 | 1 | 0 | -3.594601 | 6.677958 | 1.015947 |
| 101 | 1 | 0 | -2.752068 | 6.44295 | 2.547441 |
| 102 | 1 | 0 | -1.8345 | 6.812286 | 1.074307 |
| 103 | 6 | 0 | 3.577437 | 6.311232 | -1.020272 |
| 104 | 1 | 0 | 4.401186 | 6.426427 | -1.735392 |
| 105 | 1 | 0 | 2.705398 | 6.820163 | -1.439828 |
| 106 | 1 | 0 | 3.871194 | 6.827618 | -0.101709 |
| 107 | 6 | 0 | -3.660927 | -6.167468 | -1.61936 |
| 108 | 1 | 0 | -4.454177 | -6.180968 | -2.376897 |
| 109 | 1 | 0 | -2.792166 | -6.674978 | -2.048979 |
| 110 | 1 | 0 | -4.016078 | -6.751767 | -0.765627 |
| 111 | 6 | 0 | 2.292869 | -6.319854 | 1.330832 |
| 112 | 1 | 0 | 1.269928 | -6.708456 | 1.315961 |
| 113 | 1 | 0 | 2.858005 | -6.864331 | 0.564573 |
| 114 | 1 | 0 | 2.738408 | -6.558186 | 2.300216 |
| 115 | 6 | 0 | -1.108003 | -2.851733 | 5.735939 |
| 116 | 1 | 0 | -1.774519 | -2.517369 | 6.535608 |
| 117 | 1 | 0 | -1.253768 | -3.932521 | 5.616454 |
| 118 | 1 | 0 | -0.074421 | -2.700164 | 6.063141 |
| 119 | 6 | 0 | 0.750312 | 2.458717 | 5.802492 |
| 120 | 1 | 0 | 1.614867 | 2.587473 | 6.459077 |
| 121 | 1 | 0 | 0.272573 | 3.433723 | 5.665342 |
| 122 | 1 | 0 | 0.029414 | 1.816912 | 6.323509 |
| 123 | 1 | 0 | 3.029004 | -0.587613 | -4.875742 |

**Table S2.** Cartesian coordinates for $Ru^{II}Ru^{II}$-6pic-$H^+$.

| Center number | Atomic number | Atomic type | Coordinates (Ångström) | | |
|---|---|---|---|---|---|
| | | | X | Y | Z |
| 1 | 6 | 0 | -4.541202 | 0.627823 | -2.126251 |
| 2 | 6 | 0 | -4.588839 | 0.800986 | -3.52503 |
| 3 | 6 | 0 | -5.806391 | 1.024904 | -4.168433 |
| 4 | 6 | 0 | -6.941977 | 1.05309 | -3.353745 |
| 5 | 6 | 0 | -6.882283 | 0.857382 | -1.959285 |
| 6 | 6 | 0 | -5.67178 | 0.630112 | -1.302763 |
| 7 | 6 | 0 | -2.483824 | 0.453845 | -2.846524 |
| 8 | 6 | 0 | -5.622962 | 0.378109 | 0.197688 |
| 9 | 8 | 0 | -6.669334 | 0.478986 | 0.831322 |
| 10 | 8 | 0 | -4.486585 | 0.040507 | 0.77159 |
| 11 | 7 | 0 | -3.236569 | 0.436114 | -1.760241 |
| 12 | 7 | 0 | -3.262591 | 0.679947 | -3.957595 |
| 13 | 6 | 0 | -0.000614 | -0.000745 | -3.510661 |
| 14 | 6 | 0 | 1.075163 | -0.213821 | -2.648215 |
| 15 | 7 | 0 | -0.668363 | 0.10729 | -1.329966 |
| 16 | 7 | 0 | 0.667563 | -0.109027 | -1.330087 |
| 17 | 6 | 0 | -1.076239 | 0.21223 | -2.648017 |
| 18 | 6 | 0 | 4.540834 | -0.625637 | -2.12795 |
| 19 | 6 | 0 | 4.587928 | -0.799367 | -3.52667 |
| 20 | 6 | 0 | 5.805347 | -1.022189 | -4.170703 |
| 21 | 6 | 0 | 6.941443 | -1.048707 | -3.356678 |

| | | | | | |
|---|---|---|---|---|---|
| 22 | 6 | 0 | 6.882298 | -0.852498 | -1.962268 |
| 23 | 6 | 0 | 5.671918 | -0.626351 | -1.305134 |
| 24 | 6 | 0 | 2.482894 | -0.454257 | -2.847215 |
| 25 | 7 | 0 | 3.236165 | -0.435211 | -1.761325 |
| 26 | 7 | 0 | 3.261261 | -0.680215 | -3.958544 |
| 27 | 6 | 0 | 5.62372 | -0.374012 | 0.195248 |
| 28 | 8 | 0 | 6.670628 | -0.473178 | 0.828265 |
| 29 | 8 | 0 | 4.487175 | -0.038126 | 0.769818 |
| 30 | 44 | 0 | 2.519734 | 0.018447 | 0.044156 |
| 31 | 44 | 0 | -2.519566 | -0.018646 | 0.044681 |
| 32 | 6 | 0 | 3.20707 | -2.568622 | 1.502677 |
| 33 | 6 | 0 | 1.630118 | -2.926456 | -0.138087 |
| 34 | 6 | 0 | 3.227118 | -3.919626 | 1.826269 |
| 35 | 6 | 0 | 1.602029 | -4.289073 | 0.130989 |
| 36 | 6 | 0 | 2.414442 | -4.827489 | 1.136092 |
| 37 | 6 | 0 | 3.772059 | 2.765688 | 0.289482 |
| 38 | 6 | 0 | 2.147668 | 2.777723 | -1.344111 |
| 39 | 6 | 0 | 4.059738 | 4.105014 | 0.062386 |
| 40 | 6 | 0 | 2.387466 | 4.117721 | -1.627226 |
| 41 | 6 | 0 | 3.36709 | 4.823788 | -0.919916 |
| 42 | 6 | 0 | -3.201287 | 2.567676 | 1.506382 |
| 43 | 6 | 0 | -1.629545 | 2.925806 | -0.139349 |
| 44 | 6 | 0 | -3.220329 | 3.918618 | 1.830257 |
| 45 | 6 | 0 | -1.600647 | 4.288392 | 0.129852 |
| 46 | 6 | 0 | -2.409936 | 4.826628 | 1.137558 |
| 47 | 6 | 0 | -3.777119 | -2.763953 | 0.28676 |
| 48 | 6 | 0 | -2.148337 | -2.77897 | -1.342399 |
| 49 | 6 | 0 | -4.066276 | -4.10324 | 0.058813 |
| 50 | 6 | 0 | -2.389095 | -4.118302 | -1.625898 |
| 51 | 6 | 0 | -3.372436 | -4.822863 | -0.921364 |
| 52 | 6 | 0 | 2.771697 | 0.662196 | 3.040249 |
| 53 | 6 | 0 | 0.747356 | 1.362263 | 2.192786 |
| 54 | 6 | 0 | 2.494755 | 1.239824 | 4.274529 |
| 55 | 6 | 0 | 0.411535 | 1.977434 | 3.392227 |
| 56 | 6 | 0 | 1.291978 | 1.925076 | 4.480355 |
| 57 | 6 | 0 | -2.770981 | -0.666764 | 3.040345 |
| 58 | 6 | 0 | -0.746065 | -1.363089 | 2.191211 |
| 59 | 6 | 0 | -2.493669 | -1.245688 | 4.273189 |
| 60 | 6 | 0 | -0.409576 | -1.980075 | 3.3903 |
| 61 | 6 | 0 | -1.289477 | -1.930385 | 4.477994 |
| 62 | 7 | 0 | -2.824406 | -2.089905 | -0.39869 |
| 63 | 7 | 0 | -2.409787 | 2.053349 | 0.536277 |
| 64 | 7 | 0 | -1.90987 | -0.70878 | 1.998905 |
| 65 | 7 | 0 | 1.911088 | 0.706723 | 1.999388 |
| 66 | 7 | 0 | 2.412446 | -2.054085 | 0.535247 |
| 67 | 7 | 0 | 2.821814 | 2.09002 | -0.398595 |
| 68 | 1 | 0 | 5.878878 | -1.163551 | -5.244135 |
| 69 | 1 | 0 | 7.908327 | -1.218921 | -3.819838 |
| 70 | 1 | 0 | 7.78689 | -0.866124 | -1.364145 |
| 71 | 1 | 0 | -0.000701 | -0.000651 | -4.591233 |
| 72 | 1 | 0 | -5.880401 | 1.165901 | -5.241882 |
| 73 | 1 | 0 | -7.908928 | 1.224231 | -3.816423 |
| 74 | 1 | 0 | -7.786525 | 0.872281 | -1.360668 |
| 75 | 1 | 0 | 1.001222 | -2.495088 | -0.90572 |
| 76 | 1 | 0 | 3.863286 | -1.864077 | 1.995959 |

| | | | | | |
|---|---|---|---|---|---|
| 77 | 1 | 0 | 3.894479 | -4.259028 | 2.612756 |
| 78 | 1 | 0 | 0.938724 | -4.925574 | -0.4467 |
| 79 | 1 | 0 | 4.321498 | 2.184835 | 1.019873 |
| 80 | 1 | 0 | 1.390495 | 2.226409 | -1.886426 |
| 81 | 1 | 0 | 4.837842 | 4.58009 | 0.65219 |
| 82 | 1 | 0 | 1.808495 | 4.604012 | -2.406989 |
| 83 | 1 | 0 | -3.885191 | 4.257897 | 2.618913 |
| 84 | 1 | 0 | -0.939202 | 4.925005 | -0.449845 |
| 85 | 1 | 0 | -1.00296 | 2.494622 | -0.90898 |
| 86 | 1 | 0 | -3.855911 | 1.863022 | 2.001619 |
| 87 | 1 | 0 | -4.327382 | -2.182515 | 1.0161 |
| 88 | 1 | 0 | -1.388663 | -2.228737 | -1.882279 |
| 89 | 1 | 0 | -4.846332 | -4.577363 | 0.646706 |
| 90 | 1 | 0 | -1.80813 | -4.605696 | -2.403545 |
| 91 | 1 | 0 | 3.723552 | 0.184022 | 2.839503 |
| 92 | 1 | 0 | 0.065642 | 1.374954 | 1.352291 |
| 93 | 1 | 0 | 3.232121 | 1.163275 | 5.068059 |
| 94 | 1 | 0 | -0.542232 | 2.49016 | 3.470708 |
| 95 | 1 | 0 | -3.723166 | -0.189081 | 2.839948 |
| 96 | 1 | 0 | -0.064049 | -1.373969 | 1.350924 |
| 97 | 1 | 0 | -3.231464 | -1.171219 | 5.066649 |
| 98 | 1 | 0 | 0.544541 | -2.492069 | 3.468161 |
| 99 | 6 | 0 | -2.434328 | 6.301573 | 1.439196 |
| 100 | 1 | 0 | -3.271021 | 6.784933 | 0.920475 |
| 101 | 1 | 0 | -2.566292 | 6.488542 | 2.508678 |
| 102 | 1 | 0 | -1.515052 | 6.794205 | 1.111753 |
| 103 | 6 | 0 | 3.683049 | 6.26626 | -1.21407 |
| 104 | 1 | 0 | 4.595359 | 6.343841 | -1.817481 |
| 105 | 1 | 0 | 2.876667 | 6.750836 | -1.77038 |
| 106 | 1 | 0 | 3.857543 | 6.831143 | -0.293545 |
| 107 | 6 | 0 | -3.690951 | -6.263689 | -1.220843 |
| 108 | 1 | 0 | -4.534303 | -6.332159 | -1.918637 |
| 109 | 1 | 0 | -2.842333 | -6.777072 | -1.68062 |
| 110 | 1 | 0 | -3.976179 | -6.806069 | -0.315097 |
| 111 | 6 | 0 | 2.439606 | -6.302455 | 1.437565 |
| 112 | 1 | 0 | 1.519175 | -6.794922 | 1.113139 |
| 113 | 1 | 0 | 3.274527 | -6.785918 | 0.916088 |
| 114 | 1 | 0 | 2.575023 | -6.489484 | 2.506602 |
| 115 | 6 | 0 | -0.974754 | -2.603359 | 5.788234 |
| 116 | 1 | 0 | -1.371976 | -2.035396 | 6.634266 |
| 117 | 1 | 0 | -1.427874 | -3.601454 | 5.827119 |
| 118 | 1 | 0 | 0.102536 | -2.725621 | 5.928147 |
| 119 | 6 | 0 | 0.97492 | 2.597696 | 5.790234 |
| 120 | 1 | 0 | 1.507022 | 2.127282 | 6.621197 |
| 121 | 1 | 0 | 1.27556 | 3.652237 | 5.764234 |
| 122 | 1 | 0 | -0.097185 | 2.570349 | 6.004847 |
| 123 | 1 | 0 | 2.932413 | -0.768123 | -4.906927 |
| 124 | 1 | 0 | -2.93397 | 0.768455 | -4.906001 |

**Table S3.** Cartesian coordinates for $Ru^{II}Ru^{II}$-6pic-$OH_2$-A.

| | | | Coordinates (Ångström) | | |
|---|---|---|---|---|---|
| **Center** | **Atomic** | **Atomic** | **X** | **Y** | **Z** |

| number | number | type | | | |
|---|---|---|---|---|---|
| 1 | 6 | 0 | -3.774873 | -0.254549 | -2.995239 |
| 2 | 6 | 0 | -3.202249 | -0.416623 | -4.278348 |
| 3 | 6 | 0 | -3.943513 | -0.426711 | -5.457108 |
| 4 | 6 | 0 | -5.321403 | -0.272732 | -5.326393 |
| 5 | 6 | 0 | -5.912367 | -0.129608 | -4.063996 |
| 6 | 6 | 0 | -5.173435 | -0.121654 | -2.875673 |
| 7 | 6 | 0 | -1.607375 | -0.451499 | -2.707577 |
| 8 | 6 | 0 | -5.94975 | -0.007117 | -1.584759 |
| 9 | 8 | 0 | -7.173867 | 0.121008 | -1.630958 |
| 10 | 8 | 0 | -5.296987 | -0.068213 | -0.4582 |
| 11 | 7 | 0 | -2.755624 | -0.277334 | -2.033328 |
| 12 | 7 | 0 | -1.84787 | -0.546808 | -4.053804 |
| 13 | 6 | 0 | 0.796158 | -1.271447 | -2.919947 |
| 14 | 6 | 0 | 1.920483 | -1.077471 | -2.116765 |
| 15 | 7 | 0 | 0.257792 | 0.002127 | -1.128003 |
| 16 | 7 | 0 | 1.570756 | -0.298526 | -1.035153 |
| 17 | 6 | 0 | -0.234825 | -0.584981 | -2.255566 |
| 18 | 6 | 0 | 5.233158 | -1.59115 | -1.164573 |
| 19 | 6 | 0 | 5.242089 | -2.387124 | -2.351083 |
| 20 | 6 | 0 | 6.437261 | -3.046285 | -2.681232 |
| 21 | 6 | 0 | 7.534041 | -2.880369 | -1.83101 |
| 22 | 6 | 0 | 7.484923 | -2.080935 | -0.666198 |
| 23 | 6 | 0 | 6.321134 | -1.40227 | -0.298967 |
| 24 | 6 | 0 | 3.29856 | -1.544718 | -2.133969 |
| 25 | 7 | 0 | 3.989017 | -1.08463 | -1.060449 |
| 26 | 7 | 0 | 3.993294 | -2.341674 | -2.956097 |
| 27 | 6 | 0 | 6.257423 | -0.521729 | 0.935674 |
| 28 | 8 | 0 | 7.237944 | -0.471484 | 1.680188 |
| 29 | 8 | 0 | 5.174839 | 0.205126 | 1.173562 |
| 30 | 44 | 0 | 3.241815 | 0.156323 | 0.289674 |
| 31 | 44 | 0 | -3.29018 | -0.108175 | 0.032649 |
| 32 | 6 | 0 | 3.373752 | -1.473913 | 2.812193 |
| 33 | 6 | 0 | 2.082059 | -2.519266 | 1.210511 |
| 34 | 6 | 0 | 3.163553 | -2.513002 | 3.708853 |
| 35 | 6 | 0 | 1.83199 | -3.591753 | 2.0594 |
| 36 | 6 | 0 | 2.374046 | -3.612507 | 3.349323 |
| 37 | 6 | 0 | 5.045288 | 2.126105 | -1.172081 |
| 38 | 6 | 0 | 2.814538 | 2.525061 | -1.598901 |
| 39 | 6 | 0 | 5.426435 | 3.178812 | -1.995904 |
| 40 | 6 | 0 | 3.12469 | 3.592549 | -2.43211 |
| 41 | 6 | 0 | 4.461052 | 3.944913 | -2.657404 |
| 42 | 6 | 0 | -4.352334 | 2.729441 | 0.070896 |
| 43 | 6 | 0 | -2.114391 | 2.657933 | -0.494365 |
| 44 | 6 | 0 | -4.373752 | 4.113812 | -0.044812 |
| 45 | 6 | 0 | -2.067176 | 4.040045 | -0.627378 |
| 46 | 6 | 0 | -3.214174 | 4.811101 | -0.402891 |
| 47 | 6 | 0 | -4.513509 | -2.861964 | 0.462081 |
| 48 | 6 | 0 | -2.320576 | -2.98773 | -0.237999 |
| 49 | 6 | 0 | -4.621416 | -4.246091 | 0.486645 |
| 50 | 6 | 0 | -2.358867 | -4.376672 | -0.237815 |
| 51 | 6 | 0 | -3.529406 | -5.048485 | 0.132974 |
| 52 | 6 | 0 | 3.52496 | 2.270136 | 2.450713 |
| 53 | 6 | 0 | 1.287234 | 1.873532 | 2.058424 |
| 54 | 6 | 0 | 3.222898 | 3.250541 | 3.387168 |

| | | | | | |
|---|---|---|---|---|---|
| 55 | 6 | 0 | 0.91386 | 2.842575 | 2.985552 |
| 56 | 6 | 0 | 1.889212 | 3.569039 | 3.676202 |
| 57 | 6 | 0 | -5.279605 | -0.017781 | 2.376209 |
| 58 | 6 | 0 | -3.125989 | 0.446919 | 3.056195 |
| 59 | 6 | 0 | -5.757377 | 0.13516 | 3.672578 |
| 60 | 6 | 0 | -3.537294 | 0.613961 | 4.372444 |
| 61 | 6 | 0 | -4.8838 | 0.449477 | 4.719047 |
| 62 | 7 | 0 | -3.375362 | -2.219668 | 0.11606 |
| 63 | 7 | 0 | -3.238265 | 1.995045 | -0.143504 |
| 64 | 7 | 0 | -3.972525 | 0.118019 | 2.053927 |
| 65 | 7 | 0 | 2.573 | 1.581648 | 1.774884 |
| 66 | 7 | 0 | 2.829211 | -1.453601 | 1.572508 |
| 67 | 7 | 0 | 3.753643 | 1.790024 | -0.965669 |
| 68 | 1 | 0 | 6.507566 | -3.666962 | -3.570469 |
| 69 | 1 | 0 | 8.466108 | -3.384737 | -2.073652 |
| 70 | 1 | 0 | 8.359271 | -1.976157 | -0.031825 |
| 71 | 1 | 0 | 0.745193 | -1.866056 | -3.820519 |
| 72 | 1 | 0 | -3.469292 | -0.550007 | -6.425909 |
| 73 | 1 | 0 | -5.947027 | -0.271545 | -6.213721 |
| 74 | 1 | 0 | -6.986792 | -0.026086 | -3.961969 |
| 75 | 1 | 0 | 1.690931 | -2.497367 | 0.20092 |
| 76 | 1 | 0 | 4.019996 | -0.637677 | 3.048677 |
| 77 | 1 | 0 | 3.635428 | -2.465094 | 4.685952 |
| 78 | 1 | 0 | 1.224011 | -4.417816 | 1.701232 |
| 79 | 1 | 0 | 5.771096 | 1.528992 | -0.636851 |
| 80 | 1 | 0 | 1.788102 | 2.222713 | -1.427674 |
| 81 | 1 | 0 | 6.48408 | 3.393608 | -2.11631 |
| 82 | 1 | 0 | 2.318901 | 4.142039 | -2.910745 |
| 83 | 1 | 0 | -5.304166 | 4.642392 | 0.139896 |
| 84 | 1 | 0 | -1.128678 | 4.509689 | -0.906781 |
| 85 | 1 | 0 | -1.235026 | 2.049639 | -0.673477 |
| 86 | 1 | 0 | -5.243063 | 2.164916 | 0.31893 |
| 87 | 1 | 0 | -5.357102 | -2.225362 | 0.69423 |
| 88 | 1 | 0 | -1.42715 | -2.458292 | -0.5487 |
| 89 | 1 | 0 | -5.567898 | -4.694138 | 0.774385 |
| 90 | 1 | 0 | -1.472704 | -4.92813 | -0.537699 |
| 91 | 1 | 0 | 4.546721 | 1.978471 | 2.224181 |
| 92 | 1 | 0 | 0.538459 | 1.300666 | 1.525303 |
| 93 | 1 | 0 | 4.03726 | 3.761889 | 3.892976 |
| 94 | 1 | 0 | -0.142705 | 3.026857 | 3.160263 |
| 95 | 1 | 0 | -5.939846 | -0.217715 | 1.539221 |
| 96 | 1 | 0 | -2.091065 | 0.58126 | 2.767471 |
| 97 | 1 | 0 | -6.821151 | 0.014243 | 3.855641 |
| 98 | 1 | 0 | -2.799186 | 0.877608 | 5.124441 |
| 99 | 8 | 0 | -1.267166 | -0.102822 | 0.856605 |
| 100 | 1 | 0 | -0.566817 | 0.00534 | 0.059623 |
| 101 | 1 | 0 | -1.057384 | -0.946139 | 1.280861 |
| 102 | 6 | 0 | -3.207324 | 6.308392 | -0.570024 |
| 103 | 1 | 0 | -3.419374 | 6.581254 | -1.611147 |
| 104 | 1 | 0 | -3.96899 | 6.784301 | 0.05374 |
| 105 | 1 | 0 | -2.233481 | 6.735139 | -0.313197 |
| 106 | 6 | 0 | -5.361614 | 0.588585 | 6.140869 |
| 107 | 1 | 0 | -5.30961 | -0.375343 | 6.66214 |
| 108 | 1 | 0 | -4.745631 | 1.296176 | 6.703051 |
| 109 | 1 | 0 | -6.400997 | 0.925923 | 6.182092 |

| | | | | | |
|---|---|---|---|---|---|
| 110 | 6 | 0 | 1.532684 | 4.643613 | 4.671371 |
| 111 | 1 | 0 | 1.779711 | 5.638047 | 4.280417 |
| 112 | 1 | 0 | 0.464949 | 4.634673 | 4.906942 |
| 113 | 1 | 0 | 2.090154 | 4.519954 | 5.605863 |
| 114 | 6 | 0 | 4.840073 | 5.069494 | -3.586287 |
| 115 | 1 | 0 | 4.98502 | 4.695808 | -4.607526 |
| 116 | 1 | 0 | 4.061205 | 5.836665 | -3.626297 |
| 117 | 1 | 0 | 5.776126 | 5.543733 | -3.277825 |
| 118 | 6 | 0 | 2.158597 | -4.77154 | 4.288005 |
| 119 | 1 | 0 | 1.297927 | -5.376612 | 3.989251 |
| 120 | 1 | 0 | 3.036408 | -5.42912 | 4.299799 |
| 121 | 1 | 0 | 1.998458 | -4.429437 | 5.315418 |
| 122 | 6 | 0 | -3.623638 | -6.551797 | 0.115752 |
| 123 | 1 | 0 | -4.061083 | -6.899795 | -0.828 |
| 124 | 1 | 0 | -2.638832 | -7.01668 | 0.211944 |
| 125 | 1 | 0 | -4.26114 | -6.921076 | 0.924411 |
| 126 | 1 | 0 | -1.117112 | -0.592793 | -4.746618 |

**Table S4.** Cartesian coordinates for $Ru^{II}Ru^{II}$-6pic-$H^{+}$-$OH_2$.

| Center number | Atomic number | Atomic type | Coordinates (Ångström) | | |
|---|---|---|---|---|---|
| | | | X | Y | Z |
| 1 | 6 | 0 | -3.721218 | -0.250419 | -3.039315 |
| 2 | 6 | 0 | -3.138047 | -0.394472 | -4.318302 |
| 3 | 6 | 0 | -3.865682 | -0.377168 | -5.507006 |
| 4 | 6 | 0 | -5.241454 | -0.215223 | -5.386288 |
| 5 | 6 | 0 | -5.845161 | -0.091068 | -4.125758 |
| 6 | 6 | 0 | -5.12071 | -0.109258 | -2.93111 |
| 7 | 6 | 0 | -1.562362 | -0.465863 | -2.73326 |
| 8 | 6 | 0 | -5.912794 | -0.016026 | -1.647568 |
| 9 | 8 | 0 | -7.134473 | 0.103956 | -1.706525 |
| 10 | 8 | 0 | -5.271503 | -0.088896 | -0.513918 |
| 11 | 7 | 0 | -2.710611 | -0.294208 | -2.065735 |
| 12 | 7 | 0 | -1.78643 | -0.541599 | -4.083461 |
| 13 | 6 | 0 | 0.825058 | -1.355039 | -2.908031 |
| 14 | 6 | 0 | 1.95448 | -1.140072 | -2.114464 |
| 15 | 7 | 0 | 0.321309 | 0.009449 | -1.172089 |
| 16 | 7 | 0 | 1.623537 | -0.304606 | -1.067635 |
| 17 | 6 | 0 | -0.187008 | -0.616701 | -2.269083 |
| 18 | 6 | 0 | 5.279862 | -1.691317 | -1.088268 |
| 19 | 6 | 0 | 5.365828 | -2.508791 | -2.234312 |
| 20 | 6 | 0 | 6.554487 | -3.175807 | -2.534661 |
| 21 | 6 | 0 | 7.615332 | -2.979565 | -1.645924 |
| 22 | 6 | 0 | 7.513033 | -2.157711 | -0.504406 |
| 23 | 6 | 0 | 6.333271 | -1.481545 | -0.191277 |
| 24 | 6 | 0 | 3.322567 | -1.605814 | -2.067473 |
| 25 | 7 | 0 | 4.019459 | -1.16864 | -1.029591 |
| 26 | 7 | 0 | 4.103525 | -2.43521 | -2.838563 |
| 27 | 6 | 0 | 6.236384 | -0.589001 | 1.044966 |
| 28 | 8 | 0 | 7.219619 | -0.531227 | 1.77784 |
| 29 | 8 | 0 | 5.136013 | 0.092052 | 1.283944 |

| | | | | | |
|---|---|---|---|---|---|
| 30 | 44 | 0 | 3.25854 | 0.112856 | 0.292897 |
| 31 | 44 | 0 | -3.280288 | -0.098058 | -0.00321 |
| 32 | 6 | 0 | 3.252997 | -1.568602 | 2.813617 |
| 33 | 6 | 0 | 1.734549 | -2.38338 | 1.283899 |
| 34 | 6 | 0 | 2.899067 | -2.545636 | 3.735821 |
| 35 | 6 | 0 | 1.331448 | -3.385957 | 2.157872 |
| 36 | 6 | 0 | 1.913758 | -3.490777 | 3.427598 |
| 37 | 6 | 0 | 5.048576 | 2.437221 | -0.501129 |
| 38 | 6 | 0 | 3.303626 | 2.198527 | -1.990266 |
| 39 | 6 | 0 | 5.539125 | 3.533362 | -1.198905 |
| 40 | 6 | 0 | 3.740475 | 3.286665 | -2.735946 |
| 41 | 6 | 0 | 4.890129 | 3.98777 | -2.353622 |
| 42 | 6 | 0 | -4.374564 | 2.725163 | 0.02765 |
| 43 | 6 | 0 | -2.161596 | 2.680855 | -0.625269 |
| 44 | 6 | 0 | -4.424138 | 4.106199 | -0.109205 |
| 45 | 6 | 0 | -2.141614 | 4.061745 | -0.782299 |
| 46 | 6 | 0 | -3.291571 | 4.817986 | -0.524118 |
| 47 | 6 | 0 | -4.517482 | -2.84454 | 0.451182 |
| 48 | 6 | 0 | -2.304422 | -2.995561 | -0.168616 |
| 49 | 6 | 0 | -4.63214 | -4.226908 | 0.50749 |
| 50 | 6 | 0 | -2.347495 | -4.384383 | -0.134991 |
| 51 | 6 | 0 | -3.532553 | -5.043422 | 0.21317 |
| 52 | 6 | 0 | 3.301816 | 1.974888 | 2.716005 |
| 53 | 6 | 0 | 1.330645 | 2.189769 | 1.538413 |
| 54 | 6 | 0 | 2.916339 | 2.96565 | 3.610625 |
| 55 | 6 | 0 | 0.883227 | 3.191792 | 2.392046 |
| 56 | 6 | 0 | 1.6794 | 3.607212 | 3.466076 |
| 57 | 6 | 0 | -5.302815 | -0.003766 | 2.316643 |
| 58 | 6 | 0 | -3.177208 | 0.567085 | 3.0024 |
| 59 | 6 | 0 | -5.801798 | 0.175202 | 3.601138 |
| 60 | 6 | 0 | -3.6106 | 0.764309 | 4.307366 |
| 61 | 6 | 0 | -4.953722 | 0.559312 | 4.645998 |
| 62 | 7 | 0 | -3.364915 | -2.213742 | 0.129057 |
| 63 | 7 | 0 | -3.256896 | 2.004252 | -0.218291 |
| 64 | 7 | 0 | -3.997592 | 0.169632 | 2.003499 |
| 65 | 7 | 0 | 2.520909 | 1.574577 | 1.68678 |
| 66 | 7 | 0 | 2.675383 | -1.464821 | 1.594471 |
| 67 | 7 | 0 | 3.940357 | 1.761527 | -0.883053 |
| 68 | 1 | 0 | 6.661417 | -3.813347 | -3.406282 |
| 69 | 1 | 0 | 8.557306 | -3.481388 | -1.844265 |
| 70 | 1 | 0 | 8.359387 | -2.031708 | 0.161943 |
| 71 | 1 | 0 | 0.735908 | -1.986546 | -3.78051 |
| 72 | 1 | 0 | -3.383226 | -0.485785 | -6.473272 |
| 73 | 1 | 0 | -5.858959 | -0.192984 | -6.278583 |
| 74 | 1 | 0 | -6.920152 | 0.016791 | -4.034592 |
| 75 | 1 | 0 | 1.30165 | -2.297654 | 0.294904 |
| 76 | 1 | 0 | 4.038038 | -0.851914 | 3.018854 |
| 77 | 1 | 0 | 3.407148 | -2.567705 | 4.695144 |
| 78 | 1 | 0 | 0.565113 | -4.087385 | 1.840599 |
| 79 | 1 | 0 | 5.539095 | 2.057844 | 0.387351 |
| 80 | 1 | 0 | 2.409889 | 1.65411 | -2.267638 |
| 81 | 1 | 0 | 6.434679 | 4.027979 | -0.835261 |
| 82 | 1 | 0 | 3.177738 | 3.584281 | -3.615773 |
| 83 | 1 | 0 | -5.356088 | 4.621415 | 0.102707 |
| 84 | 1 | 0 | -1.225712 | 4.542561 | -1.113009 |

| | | | | | |
|---|---|---|---|---|---|
| 85 | 1 | 0 | -1.281491 | 2.083591 | -0.835392 |
| 86 | 1 | 0 | -5.245928 | 2.152471 | 0.320124 |
| 87 | 1 | 0 | -5.36712 | -2.20095 | 0.634964 |
| 88 | 1 | 0 | -1.399515 | -2.475824 | -0.462675 |
| 89 | 1 | 0 | -5.590542 | -4.663184 | 0.77235 |
| 90 | 1 | 0 | -1.456331 | -4.948732 | -0.394021 |
| 91 | 1 | 0 | 4.267341 | 1.483336 | 2.784902 |
| 92 | 1 | 0 | 0.728027 | 1.858074 | 0.702531 |
| 93 | 1 | 0 | 3.591865 | 3.238421 | 4.416081 |
| 94 | 1 | 0 | -0.08598 | 3.645933 | 2.208199 |
| 95 | 1 | 0 | -5.948362 | -0.251315 | 1.481737 |
| 96 | 1 | 0 | -2.144763 | 0.736691 | 2.723798 |
| 97 | 1 | 0 | -6.862493 | 0.021328 | 3.775862 |
| 98 | 1 | 0 | -2.893428 | 1.085365 | 5.057067 |
| 99 | 8 | 0 | -1.257514 | -0.06098 | 0.865251 |
| 100 | 1 | 0 | -0.568098 | 0.039732 | 0.120404 |
| 101 | 1 | 0 | -1.047855 | -0.885603 | 1.324248 |
| 102 | 6 | 0 | -3.322316 | 6.311972 | -0.711275 |
| 103 | 1 | 0 | -3.789801 | 6.569861 | -1.669188 |
| 104 | 1 | 0 | -3.907109 | 6.799801 | 0.074074 |
| 105 | 1 | 0 | -2.316054 | 6.738864 | -0.709465 |
| 106 | 6 | 0 | -5.455817 | 0.73102 | 6.055084 |
| 107 | 1 | 0 | -5.441323 | -0.227729 | 6.587805 |
| 108 | 1 | 0 | -4.83359 | 1.4295 | 6.620907 |
| 109 | 1 | 0 | -6.486864 | 1.094801 | 6.069787 |
| 110 | 6 | 0 | 1.246801 | 4.703861 | 4.40374 |
| 111 | 1 | 0 | 1.7937 | 5.630496 | 4.192797 |
| 112 | 1 | 0 | 0.179569 | 4.918591 | 4.307088 |
| 113 | 1 | 0 | 1.453379 | 4.439732 | 5.445447 |
| 114 | 6 | 0 | 5.415102 | 5.152242 | -3.150846 |
| 115 | 1 | 0 | 6.217617 | 4.826358 | -3.823646 |
| 116 | 1 | 0 | 4.632525 | 5.603861 | -3.76604 |
| 117 | 1 | 0 | 5.833614 | 5.924898 | -2.499655 |
| 118 | 6 | 0 | 1.530321 | -4.580167 | 4.393892 |
| 119 | 1 | 0 | 0.539529 | -4.985182 | 4.171707 |
| 120 | 1 | 0 | 2.246123 | -5.409384 | 4.341184 |
| 121 | 1 | 0 | 1.532689 | -4.216954 | 5.425551 |
| 122 | 6 | 0 | -3.63541 | -6.545454 | 0.238495 |
| 123 | 1 | 0 | -4.183526 | -6.909008 | -0.638745 |
| 124 | 1 | 0 | -2.649779 | -7.017579 | 0.233266 |
| 125 | 1 | 0 | -4.179012 | -6.891157 | 1.123014 |
| 126 | 1 | 0 | -1.054677 | -0.558251 | -4.776297 |
| 127 | 1 | 0 | 3.821945 | -2.898415 | -3.687936 |

**Table S5.** Cartesian coordinates for $Ru^{II}Ru^{II}$-4pic-2$OH_2$-A.

| Center number | Atomic number | Atomic type | Coordinates (Ångström) | | |
|---|---|---|---|---|---|
| | | | X | Y | Z |
| 1 | 6 | 0 | 4.524257 | 0.40681 | 1.475317 |
| 2 | 6 | 0 | 4.724929 | 0.47996 | 2.870502 |
| 3 | 6 | 0 | 6.017675 | 0.564147 | 3.387527 |

| | | | | | |
|---|---|---|---|---|---|
| 4 | 6 | 0 | 7.060753 | 0.558185 | 2.454278 |
| 5 | 6 | 0 | 6.840588 | 0.465835 | 1.065559 |
| 6 | 6 | 0 | 5.553876 | 0.381702 | 0.5287 |
| 7 | 6 | 0 | 2.534336 | 0.340413 | 2.396784 |
| 8 | 6 | 0 | 5.333098 | 0.260513 | -0.987801 |
| 9 | 8 | 0 | 6.343098 | 0.302161 | -1.691396 |
| 10 | 8 | 0 | 4.126303 | 0.121383 | -1.470085 |
| 11 | 7 | 0 | 3.181225 | 0.336678 | 1.237217 |
| 12 | 7 | 0 | 3.443005 | 0.429719 | 3.434718 |
| 13 | 6 | 0 | 0.011695 | 0.0244 | 3.142215 |
| 14 | 6 | 0 | -1.081537 | -0.115719 | 2.278154 |
| 15 | 7 | 0 | 0.67311 | 0.141461 | 0.960808 |
| 16 | 7 | 0 | -0.650883 | -0.015442 | 0.958318 |
| 17 | 6 | 0 | 1.104611 | 0.193133 | 2.283621 |
| 18 | 6 | 0 | -4.478491 | -0.536547 | 1.448604 |
| 19 | 6 | 0 | -4.678459 | -0.649562 | 2.840935 |
| 20 | 6 | 0 | -5.96207 | -0.849266 | 3.348805 |
| 21 | 6 | 0 | -6.996603 | -0.918284 | 2.408514 |
| 22 | 6 | 0 | -6.777599 | -0.787822 | 1.022465 |
| 23 | 6 | 0 | -5.50003 | -0.587554 | 0.494917 |
| 24 | 6 | 0 | -2.503722 | -0.326899 | 2.382491 |
| 25 | 7 | 0 | -3.144719 | -0.352764 | 1.219708 |
| 26 | 7 | 0 | -3.405821 | -0.509516 | 3.412737 |
| 27 | 6 | 0 | -5.280852 | -0.411244 | -1.018105 |
| 28 | 8 | 0 | -6.288273 | -0.485599 | -1.723128 |
| 29 | 8 | 0 | -4.085608 | -0.17979 | -1.48903 |
| 30 | 44 | 0 | -2.20689 | -0.04288 | -0.492006 |
| 31 | 44 | 0 | 2.236333 | 0.113197 | -0.484895 |
| 32 | 6 | 0 | -2.284475 | -2.495572 | -2.150821 |
| 33 | 6 | 0 | -1.551354 | -3.012181 | -0.021613 |
| 34 | 6 | 0 | -2.193397 | -3.825433 | -2.538797 |
| 35 | 6 | 0 | -1.440576 | -4.359462 | -0.343581 |
| 36 | 6 | 0 | -1.768038 | -4.801834 | -1.630068 |
| 37 | 6 | 0 | -3.269662 | 2.61055 | -1.367201 |
| 38 | 6 | 0 | -2.253216 | 2.81157 | 0.695308 |
| 39 | 6 | 0 | -3.605583 | 3.957904 | -1.370129 |
| 40 | 6 | 0 | -2.56775 | 4.164371 | 0.760612 |
| 41 | 6 | 0 | -3.261859 | 4.778368 | -0.288162 |
| 42 | 6 | 0 | 2.8507 | 2.706201 | -1.883979 |
| 43 | 6 | 0 | 1.328379 | 3.038247 | -0.182931 |
| 44 | 6 | 0 | 2.787767 | 4.045403 | -2.246915 |
| 45 | 6 | 0 | 1.220025 | 4.389168 | -0.491869 |
| 46 | 6 | 0 | 1.960251 | 4.932353 | -1.547793 |
| 47 | 6 | 0 | 2.898279 | -2.61048 | -1.473704 |
| 48 | 6 | 0 | 2.198482 | -2.734425 | 0.720198 |
| 49 | 6 | 0 | 3.121939 | -3.979986 | -1.514347 |
| 50 | 6 | 0 | 2.404654 | -4.10903 | 0.7503 |
| 51 | 6 | 0 | 2.88295 | -4.772031 | -0.384772 |
| 52 | 7 | 0 | 2.44098 | -1.978332 | -0.368956 |
| 53 | 7 | 0 | 2.128836 | 2.189624 | -0.860888 |
| 54 | 7 | 0 | -1.968148 | -2.082202 | -0.902652 |
| 55 | 7 | 0 | -2.589933 | 2.026195 | -0.349718 |
| 56 | 1 | 0 | -6.160425 | -0.94413 | 4.412019 |
| 57 | 1 | 0 | -8.010829 | -1.07309 | 2.765171 |
| 58 | 1 | 0 | -7.605473 | -0.835449 | 0.323288 |

| | | | | | |
|---|---|---|---|---|---|
| 59 | 1 | 0 | 0.012734 | -0.000056 | 4.222587 |
| 60 | 1 | 0 | 6.21637 | 0.625723 | 4.45313 |
| 61 | 1 | 0 | 8.082105 | 0.620639 | 2.818567 |
| 62 | 1 | 0 | 7.674239 | 0.453788 | 0.371814 |
| 63 | 1 | 0 | -1.294054 | -2.64886 | 0.966173 |
| 64 | 1 | 0 | -2.615321 | -1.717497 | -2.82637 |
| 65 | 1 | 0 | -2.46089 | -4.093826 | -3.556457 |
| 66 | 1 | 0 | -1.093197 | -5.057944 | 0.411762 |
| 67 | 1 | 0 | -3.574827 | 1.941772 | -2.164616 |
| 68 | 1 | 0 | -1.712922 | 2.326848 | 1.499697 |
| 69 | 1 | 0 | -4.152795 | 4.358584 | -2.218433 |
| 70 | 1 | 0 | -2.270908 | 4.734029 | 1.636858 |
| 71 | 1 | 0 | 3.394433 | 4.391235 | -3.078857 |
| 72 | 1 | 0 | 0.544053 | 5.009124 | 0.089241 |
| 73 | 1 | 0 | 0.749061 | 2.59806 | 0.61798 |
| 74 | 1 | 0 | 3.506249 | 2.003647 | -2.387133 |
| 75 | 1 | 0 | 3.090079 | -1.975142 | -2.32693 |
| 76 | 1 | 0 | 1.81996 | -2.212394 | 1.590701 |
| 77 | 1 | 0 | 3.493046 | -4.422916 | -2.433905 |
| 78 | 1 | 0 | 2.190595 | -4.65651 | 1.66386 |
| 79 | 8 | 0 | -1.161606 | 0.287674 | -2.347926 |
| 80 | 6 | 0 | -1.689672 | -6.256785 | -2.01559 |
| 81 | 1 | 0 | -1.061073 | -6.822659 | -1.322383 |
| 82 | 1 | 0 | -2.686101 | -6.715369 | -2.008948 |
| 83 | 1 | 0 | -1.285183 | -6.379033 | -3.02516 |
| 84 | 6 | 0 | -3.649109 | 6.234015 | -0.245911 |
| 85 | 1 | 0 | -4.710075 | 6.345969 | 0.008686 |
| 86 | 1 | 0 | -3.070492 | 6.782699 | 0.502328 |
| 87 | 1 | 0 | -3.499463 | 6.714926 | -1.21769 |
| 88 | 6 | 0 | 1.89193 | 6.395933 | -1.900079 |
| 89 | 1 | 0 | 2.737944 | 6.940967 | -1.463415 |
| 90 | 1 | 0 | 1.934772 | 6.547374 | -2.982903 |
| 91 | 1 | 0 | 0.974071 | 6.856533 | -1.524222 |
| 92 | 6 | 0 | 3.150914 | -6.255135 | -0.389362 |
| 93 | 1 | 0 | 4.225767 | -6.456712 | -0.30541 |
| 94 | 1 | 0 | 2.654988 | -6.756022 | 0.446754 |
| 95 | 1 | 0 | 2.808708 | -6.718468 | -1.320201 |
| 96 | 1 | 0 | -1.250047 | 1.213199 | -2.606376 |
| 97 | 8 | 0 | 1.193387 | -0.241865 | -2.307283 |
| 98 | 1 | 0 | 1.653307 | 0.15558 | -3.05763 |
| 99 | 1 | 0 | 0.000298 | 0.044 | -2.330192 |
| 100 | 1 | 0 | -3.184241 | -0.554038 | 4.393737 |
| 101 | 1 | 0 | 3.219823 | 0.488268 | 4.414573 |

**Table S6.** Cartesian coordinates for $Ru^{II}Ru^{II}$-4pic-$H^{+}$-$2OH_2$.

| **Center number** | **Atomic number** | **Atomic type** | **Coordinates (Ångström)** | | |
|---|---|---|---|---|---|
| | | | **X** | **Y** | **Z** |
| 1 | 6 | 0 | -4.540591 | 0.307409 | 1.385645 |
| 2 | 6 | 0 | -4.766967 | 0.326049 | 2.777579 |
| 3 | 6 | 0 | -6.07091 | 0.369132 | 3.272062 |

| | | | | | |
|---|---|---|---|---|---|
| 4 | 6 | 0 | -7.095273 | 0.384381 | 2.319789 |
| 5 | 6 | 0 | -6.849623 | 0.356634 | 0.931714 |
| 6 | 6 | 0 | -5.552403 | 0.31873 | 0.415159 |
| 7 | 6 | 0 | -2.563165 | 0.243457 | 2.352605 |
| 8 | 6 | 0 | -5.311831 | 0.287301 | -1.099725 |
| 9 | 8 | 0 | -6.293596 | 0.233925 | -1.829058 |
| 10 | 8 | 0 | -4.077239 | 0.342214 | -1.566639 |
| 11 | 7 | 0 | -3.19121 | 0.263128 | 1.183955 |
| 12 | 7 | 0 | -3.49306 | 0.283742 | 3.365808 |
| 13 | 6 | 0 | -0.031794 | 0.017083 | 3.110256 |
| 14 | 6 | 0 | 1.068371 | -0.087692 | 2.251989 |
| 15 | 7 | 0 | -0.688657 | 0.108672 | 0.928798 |
| 16 | 7 | 0 | 0.63779 | -0.018944 | 0.931568 |
| 17 | 6 | 0 | -1.125528 | 0.144217 | 2.246939 |
| 18 | 6 | 0 | 4.475061 | -0.426586 | 1.406326 |
| 19 | 6 | 0 | 4.690306 | -0.483221 | 2.798434 |
| 20 | 6 | 0 | 5.985494 | -0.621665 | 3.299363 |
| 21 | 6 | 0 | 7.011156 | -0.692189 | 2.351502 |
| 22 | 6 | 0 | 6.775689 | -0.627451 | 0.962379 |
| 23 | 6 | 0 | 5.487874 | -0.489644 | 0.441845 |
| 24 | 6 | 0 | 2.500573 | -0.252201 | 2.360084 |
| 25 | 7 | 0 | 3.133747 | -0.291186 | 1.193909 |
| 26 | 7 | 0 | 3.417881 | -0.368966 | 3.379747 |
| 27 | 6 | 0 | 5.250746 | -0.416251 | -1.070034 |
| 28 | 8 | 0 | 6.233156 | -0.503578 | -1.798237 |
| 29 | 8 | 0 | 4.029082 | -0.267391 | -1.542739 |
| 30 | 44 | 0 | 2.209197 | -0.121244 | -0.51864 |
| 31 | 44 | 0 | -2.270628 | 0.20289 | -0.532423 |
| 32 | 6 | 0 | 3.200269 | 2.53269 | -1.544859 |
| 33 | 6 | 0 | 2.322364 | 2.768044 | 0.575465 |
| 34 | 6 | 0 | 3.590292 | 3.865314 | -1.569266 |
| 35 | 6 | 0 | 2.693621 | 4.106157 | 0.622387 |
| 36 | 6 | 0 | 3.353309 | 4.694011 | -0.464538 |
| 37 | 6 | 0 | 2.728189 | -2.849446 | -1.72972 |
| 38 | 6 | 0 | 1.408594 | -3.034667 | 0.151532 |
| 39 | 6 | 0 | 2.730224 | -4.225465 | -1.908686 |
| 40 | 6 | 0 | 1.378324 | -4.419829 | 0.037745 |
| 41 | 6 | 0 | 2.047753 | -5.058037 | -1.011927 |
| 42 | 6 | 0 | -3.190541 | -2.481728 | -1.636561 |
| 43 | 6 | 0 | -2.198956 | -2.725771 | 0.427115 |
| 44 | 6 | 0 | -3.482842 | -3.836599 | -1.698778 |
| 45 | 6 | 0 | -2.471403 | -4.088832 | 0.437908 |
| 46 | 6 | 0 | -3.129646 | -4.686343 | -0.642258 |
| 47 | 6 | 0 | -3.180632 | 3.119794 | -0.549981 |
| 48 | 6 | 0 | -0.910636 | 2.89098 | -0.907351 |
| 49 | 6 | 0 | -3.106392 | 4.493813 | -0.732616 |
| 50 | 6 | 0 | -0.768211 | 4.258198 | -1.116196 |
| 51 | 6 | 0 | -1.880649 | 5.103766 | -1.031331 |
| 52 | 7 | 0 | -2.100931 | 2.310341 | -0.637024 |
| 53 | 7 | 0 | -2.546258 | -1.911008 | -0.591153 |
| 54 | 7 | 0 | 2.567171 | 1.971644 | -0.487992 |
| 55 | 7 | 0 | 2.068988 | -2.238611 | -0.714586 |
| 56 | 1 | 0 | 6.198023 | -0.670994 | 4.362329 |
| 57 | 1 | 0 | 8.033005 | -0.799399 | 2.701724 |
| 58 | 1 | 0 | 7.600829 | -0.683155 | 0.26074 |

| | | | | | |
|---|---|---|---|---|---|
| 59 | 1 | 0 | -0.035967 | 0.001344 | 4.19045 |
| 60 | 1 | 0 | -6.291416 | 0.386358 | 4.334406 |
| 61 | 1 | 0 | -8.123541 | 0.415549 | 2.665925 |
| 62 | 1 | 0 | -7.674047 | 0.362007 | 0.227008 |
| 63 | 1 | 0 | 1.805173 | 2.306781 | 1.407771 |
| 64 | 1 | 0 | 3.425385 | 1.865769 | -2.366918 |
| 65 | 1 | 0 | 4.100391 | 4.24588 | -2.448875 |
| 66 | 1 | 0 | 2.468157 | 4.685259 | 1.512817 |
| 67 | 1 | 0 | 3.30459 | -2.195456 | -2.374117 |
| 68 | 1 | 0 | 0.889651 | -2.530501 | 0.956351 |
| 69 | 1 | 0 | 3.284315 | -4.643968 | -2.743467 |
| 70 | 1 | 0 | 0.828845 | -4.997604 | 0.774326 |
| 71 | 1 | 0 | -4.006485 | -4.219826 | -2.569199 |
| 72 | 1 | 0 | -2.173042 | -4.679621 | 1.298821 |
| 73 | 1 | 0 | -1.687636 | -2.256532 | 1.258156 |
| 74 | 1 | 0 | -3.512585 | -1.809252 | -2.420899 |
| 75 | 1 | 0 | -4.125473 | 2.63538 | -0.344863 |
| 76 | 1 | 0 | -0.05619 | 2.223888 | -0.947038 |
| 77 | 1 | 0 | -4.014712 | 5.083349 | -0.653975 |
| 78 | 1 | 0 | 0.21709 | 4.654786 | -1.339134 |
| 79 | 8 | 0 | 1.252782 | 0.101511 | -2.530281 |
| 80 | 6 | 0 | 3.814455 | 6.127175 | -0.436604 |
| 81 | 1 | 0 | 3.232464 | 6.724479 | 0.270196 |
| 82 | 1 | 0 | 4.865102 | 6.183231 | -0.127304 |
| 83 | 1 | 0 | 3.744643 | 6.590114 | -1.425029 |
| 84 | 6 | 0 | 2.060217 | -6.555992 | -1.161902 |
| 85 | 1 | 0 | 3.052076 | -6.958363 | -0.9255 |
| 86 | 1 | 0 | 1.338395 | -7.034174 | -0.495366 |
| 87 | 1 | 0 | 1.830668 | -6.852888 | -2.190121 |
| 88 | 6 | 0 | -3.464682 | -6.153935 | -0.666294 |
| 89 | 1 | 0 | -4.548266 | -6.303226 | -0.598073 |
| 90 | 1 | 0 | -3.135653 | -6.619132 | -1.601133 |
| 91 | 1 | 0 | -3.000214 | -6.68779 | 0.166231 |
| 92 | 6 | 0 | -1.771744 | 6.591785 | -1.233501 |
| 93 | 1 | 0 | -1.859597 | 7.119845 | -0.27659 |
| 94 | 1 | 0 | -0.81414 | 6.867979 | -1.681205 |
| 95 | 1 | 0 | -2.573618 | 6.962059 | -1.879439 |
| 96 | 1 | 0 | 1.874694 | -0.248453 | -3.186102 |
| 97 | 8 | 0 | -1.591549 | 0.230097 | -2.7211 |
| 98 | 1 | 0 | -2.522536 | 0.215386 | -3.024462 |
| 99 | 1 | 0 | -1.280052 | 1.126207 | -2.922187 |
| 100 | 1 | 0 | 3.211882 | -0.378856 | 4.366062 |
| 101 | 1 | 0 | 0.417027 | -0.384684 | -2.628238 |
| 102 | 1 | 0 | -3.294367 | 0.294416 | 4.353786 |

**Table S7.** Cartesian coordinates for $Ru^{II}Ru^{III}$-6pic.

| Center number | Atomic number | Atomic type | Coordinates (Ångström) X | Y | Z |
|---|---|---|---|---|---|
| 1 | 6 | 0 | 4.504691 | -0.562484 | -2.051758 |
| 2 | 6 | 0 | 4.507428 | -0.741607 | -3.468222 |

| | | | | | |
|---|---|---|---|---|---|
| 3 | 6 | 0 | 5.743715 | -0.93568 | -4.097146 |
| 4 | 6 | 0 | 6.893518 | -0.925323 | -3.29853 |
| 5 | 6 | 0 | 6.854814 | -0.7175 | -1.902114 |
| 6 | 6 | 0 | 5.644444 | -0.519756 | -1.235027 |
| 7 | 6 | 0 | 2.486324 | -0.454521 | -2.855327 |
| 8 | 6 | 0 | 5.578072 | -0.241417 | 0.246865 |
| 9 | 8 | 0 | 6.604204 | -0.297358 | 0.922287 |
| 10 | 8 | 0 | 4.415958 | 0.087856 | 0.803289 |
| 11 | 7 | 0 | 3.215509 | -0.406403 | -1.694441 |
| 12 | 7 | 0 | 3.206713 | -0.664015 | -3.955371 |
| 13 | 6 | 0 | -0.000009 | 0.000024 | -3.476843 |
| 14 | 6 | 0 | -1.077686 | 0.224524 | -2.624574 |
| 15 | 7 | 0 | 0.668337 | -0.118143 | -1.2972 |
| 16 | 7 | 0 | -0.668349 | 0.118151 | -1.297196 |
| 17 | 6 | 0 | 1.077672 | -0.224484 | -2.62458 |
| 18 | 6 | 0 | -4.504703 | 0.562491 | -2.051733 |
| 19 | 6 | 0 | -4.507448 | 0.741634 | -3.468196 |
| 20 | 6 | 0 | -5.74374 | 0.935693 | -4.097114 |
| 21 | 6 | 0 | -6.893542 | 0.925289 | -3.298497 |
| 22 | 6 | 0 | -6.854832 | 0.717428 | -1.902087 |
| 23 | 6 | 0 | -5.644456 | 0.519707 | -1.235004 |
| 24 | 6 | 0 | -2.486339 | 0.454567 | -2.85531 |
| 25 | 7 | 0 | -3.215518 | 0.40643 | -1.694422 |
| 26 | 7 | 0 | -3.206737 | 0.664049 | -3.955351 |
| 27 | 6 | 0 | -5.578087 | 0.241301 | 0.246877 |
| 28 | 8 | 0 | -6.60425 | 0.297055 | 0.922269 |
| 29 | 8 | 0 | -4.415939 | -0.087801 | 0.803328 |
| 30 | 44 | 0 | -2.462614 | -0.065713 | 0.046952 |
| 31 | 44 | 0 | 2.462623 | 0.065727 | 0.046941 |
| 32 | 6 | 0 | -3.201956 | 2.474974 | 1.551282 |
| 33 | 6 | 0 | -1.758598 | 2.908472 | -0.196089 |
| 34 | 6 | 0 | -3.29871 | 3.827701 | 1.852907 |
| 35 | 6 | 0 | -1.812364 | 4.274321 | 0.049019 |
| 36 | 6 | 0 | -2.597354 | 4.773433 | 1.094956 |
| 37 | 6 | 0 | -3.628098 | -2.854399 | 0.19377 |
| 38 | 6 | 0 | -1.998096 | -2.761131 | -1.436179 |
| 39 | 6 | 0 | -3.885979 | -4.1892 | -0.090571 |
| 40 | 6 | 0 | -2.208764 | -4.092443 | -1.774883 |
| 41 | 6 | 0 | -3.174712 | -4.847489 | -1.100292 |
| 42 | 6 | 0 | 3.202018 | -2.47496 | 1.55125 |
| 43 | 6 | 0 | 1.758632 | -2.908457 | -0.1961 |
| 44 | 6 | 0 | 3.298783 | -3.827687 | 1.852868 |
| 45 | 6 | 0 | 1.812408 | -4.274307 | 0.049002 |
| 46 | 6 | 0 | 2.597417 | -4.77342 | 1.094925 |
| 47 | 6 | 0 | 3.628078 | 2.854426 | 0.193753 |
| 48 | 6 | 0 | 1.998043 | 2.761159 | -1.436162 |
| 49 | 6 | 0 | 3.885943 | 4.189232 | -0.090582 |
| 50 | 6 | 0 | 2.208695 | 4.092476 | -1.774861 |
| 51 | 6 | 0 | 3.174651 | 4.847524 | -1.100284 |
| 52 | 6 | 0 | -2.699091 | -0.828101 | 3.03497 |
| 53 | 6 | 0 | -0.645826 | -1.434537 | 2.182856 |
| 54 | 6 | 0 | -2.413839 | -1.438024 | 4.251354 |
| 55 | 6 | 0 | -0.299024 | -2.077465 | 3.365494 |
| 56 | 6 | 0 | -1.190012 | -2.089298 | 4.445215 |
| 57 | 6 | 0 | 2.699113 | 0.828095 | 3.034963 |

| | | | | | |
|---|---|---|---|---|---|
| 58 | 6 | 0 | 0.645843 | 1.434537 | 2.182861 |
| 59 | 6 | 0 | 2.413862 | 1.43801 | 4.251355 |
| 60 | 6 | 0 | 0.299045 | 2.077455 | 3.365502 |
| 61 | 6 | 0 | 1.190038 | 2.089279 | 4.445224 |
| 62 | 7 | 0 | 2.690307 | 2.13421 | -0.461645 |
| 63 | 7 | 0 | 2.432786 | -2.002019 | 0.545172 |
| 64 | 7 | 0 | 1.82736 | 0.809266 | 2.002505 |
| 65 | 7 | 0 | -1.82734 | -0.809265 | 2.002506 |
| 66 | 7 | 0 | -2.432745 | 2.002034 | 0.545189 |
| 67 | 7 | 0 | -2.690334 | -2.134185 | -0.46164 |
| 68 | 1 | 0 | -5.809212 | 1.080548 | -5.171425 |
| 69 | 1 | 0 | -7.860247 | 1.071823 | -3.772901 |
| 70 | 1 | 0 | -7.770924 | 0.696996 | -1.321101 |
| 71 | 1 | 0 | -0.00001 | 0.000031 | -4.555608 |
| 72 | 1 | 0 | 5.809182 | -1.080523 | -5.171459 |
| 73 | 1 | 0 | 7.860218 | -1.071873 | -3.772937 |
| 74 | 1 | 0 | 7.770906 | -0.697123 | -1.321126 |
| 75 | 1 | 0 | -1.160104 | 2.50311 | -1.00091 |
| 76 | 1 | 0 | -3.777284 | 1.733647 | 2.090025 |
| 77 | 1 | 0 | -3.941334 | 4.13792 | 2.671433 |
| 78 | 1 | 0 | -1.239314 | 4.944133 | -0.584745 |
| 79 | 1 | 0 | -4.195147 | -2.311601 | 0.940362 |
| 80 | 1 | 0 | -1.262975 | -2.166728 | -1.961106 |
| 81 | 1 | 0 | -4.657529 | -4.705631 | 0.472547 |
| 82 | 1 | 0 | -1.621131 | -4.529227 | -2.576897 |
| 83 | 1 | 0 | 3.941423 | -4.137905 | 2.671381 |
| 84 | 1 | 0 | 1.239351 | -4.94412 | -0.584754 |
| 85 | 1 | 0 | 1.160123 | -2.503095 | -1.00091 |
| 86 | 1 | 0 | 3.777352 | -1.733633 | 2.089987 |
| 87 | 1 | 0 | 4.195147 | 2.311626 | 0.940327 |
| 88 | 1 | 0 | 1.262915 | 2.166757 | -1.961077 |
| 89 | 1 | 0 | 4.657499 | 4.705665 | 0.472526 |
| 90 | 1 | 0 | 1.621042 | 4.529261 | -2.576859 |
| 91 | 1 | 0 | -3.6644 | -0.374873 | 2.835227 |
| 92 | 1 | 0 | 0.040268 | -1.396013 | 1.347087 |
| 93 | 1 | 0 | -3.159868 | -1.412957 | 5.040444 |
| 94 | 1 | 0 | 0.670287 | -2.561488 | 3.435673 |
| 95 | 1 | 0 | 3.664423 | 0.374871 | 2.835214 |
| 96 | 1 | 0 | -0.040252 | 1.396019 | 1.347093 |
| 97 | 1 | 0 | 3.159893 | 1.412939 | 5.040443 |
| 98 | 1 | 0 | -0.670267 | 2.561479 | 3.435687 |
| 99 | 6 | 0 | 2.713467 | -6.250357 | 1.365842 |
| 100 | 1 | 0 | 3.557351 | -6.675898 | 0.809218 |
| 101 | 1 | 0 | 2.889044 | -6.450634 | 2.426591 |
| 102 | 1 | 0 | 1.812585 | -6.786883 | 1.055371 |
| 103 | 6 | 0 | -3.461874 | -6.279694 | -1.467747 |
| 104 | 1 | 0 | -4.24701 | -6.327478 | -2.232136 |
| 105 | 1 | 0 | -2.577081 | -6.774435 | -1.877951 |
| 106 | 1 | 0 | -3.81211 | -6.852188 | -0.60424 |
| 107 | 6 | 0 | 3.461794 | 6.279734 | -1.467732 |
| 108 | 1 | 0 | 4.246916 | 6.327531 | -2.232134 |
| 109 | 1 | 0 | 2.57699 | 6.774471 | -1.877917 |
| 110 | 1 | 0 | 3.812039 | 6.852225 | -0.604227 |
| 111 | 6 | 0 | -2.713393 | 6.25037 | 1.365876 |
| 112 | 1 | 0 | -1.812449 | 6.78687 | 1.055537 |

| | | | | | |
|---|---|---|---|---|---|
| 113 | 1 | 0 | -3.557182 | 6.675949 | 0.809137 |
| 114 | 1 | 0 | -2.88911 | 6.450641 | 2.426603 |
| 115 | 6 | 0 | 0.861805 | 2.794457 | 5.735808 |
| 116 | 1 | 0 | 1.407879 | 2.361528 | 6.57847 |
| 117 | 1 | 0 | 1.136656 | 3.854948 | 5.677948 |
| 118 | 1 | 0 | -0.20864 | 2.747314 | 5.956019 |
| 119 | 6 | 0 | -0.861796 | -2.794491 | 5.735795 |
| 120 | 1 | 0 | -1.407321 | -2.361088 | 6.578572 |
| 121 | 1 | 0 | -1.137359 | -3.854812 | 5.678189 |
| 122 | 1 | 0 | 0.208748 | -2.747998 | 5.955649 |

**Table S8.** Cartesian coordinates for $Ru^{II}Ru^{III}$-6pic-$H^+$.

| Center number | Atomic number | Atomic type | Coordinates (Ångström) | | |
|---|---|---|---|---|---|
| | | | X | Y | Z |
| 1 | 6 | 0 | -4.577135 | 0.592053 | -2.062141 |
| 2 | 6 | 0 | -4.581449 | 0.71215 | -3.474956 |
| 3 | 6 | 0 | -5.802851 | 0.918013 | -4.114942 |
| 4 | 6 | 0 | -6.955507 | 0.981084 | -3.312837 |
| 5 | 6 | 0 | -6.918637 | 0.833933 | -1.915036 |
| 6 | 6 | 0 | -5.706885 | 0.623597 | -1.243255 |
| 7 | 6 | 0 | -2.552007 | 0.386108 | -2.876515 |
| 8 | 6 | 0 | -5.634687 | 0.413757 | 0.248026 |
| 9 | 8 | 0 | -6.644419 | 0.533792 | 0.930985 |
| 10 | 8 | 0 | -4.472095 | 0.073121 | 0.806701 |
| 11 | 7 | 0 | -3.280286 | 0.406078 | -1.699524 |
| 12 | 7 | 0 | -3.270876 | 0.571007 | -3.96487 |
| 13 | 6 | 0 | -0.067105 | -0.104037 | -3.493422 |
| 14 | 6 | 0 | 1.005165 | -0.303497 | -2.622688 |
| 15 | 7 | 0 | -0.733638 | 0.06883 | -1.327284 |
| 16 | 7 | 0 | 0.60327 | -0.167009 | -1.306629 |
| 17 | 6 | 0 | -1.142854 | 0.140269 | -2.645172 |
| 18 | 6 | 0 | 4.485187 | -0.65463 | -2.134689 |
| 19 | 6 | 0 | 4.511945 | -0.871875 | -3.527994 |
| 20 | 6 | 0 | 5.722088 | -1.096674 | -4.185061 |
| 21 | 6 | 0 | 6.871778 | -1.080759 | -3.39013 |
| 22 | 6 | 0 | 6.832568 | -0.842518 | -2.00164 |
| 23 | 6 | 0 | 5.629608 | -0.613683 | -1.331603 |
| 24 | 6 | 0 | 2.413898 | -0.535971 | -2.825796 |
| 25 | 7 | 0 | 3.183662 | -0.470859 | -1.753222 |
| 26 | 7 | 0 | 3.176928 | -0.78751 | -3.940532 |
| 27 | 6 | 0 | 5.601801 | -0.317165 | 0.160023 |
| 28 | 8 | 0 | 6.657859 | -0.383089 | 0.781527 |
| 29 | 8 | 0 | 4.469131 | 0.021278 | 0.743529 |
| 30 | 44 | 0 | 2.495629 | 0.030338 | 0.050328 |
| 31 | 44 | 0 | -2.539881 | -0.004968 | 0.023861 |
| 32 | 6 | 0 | 3.23859 | -2.513205 | 1.564465 |
| 33 | 6 | 0 | 1.698017 | -2.941019 | -0.093579 |
| 34 | 6 | 0 | 3.307244 | -3.860465 | 1.896652 |
| 35 | 6 | 0 | 1.720676 | -4.3024 | 0.182627 |
| 36 | 6 | 0 | 2.540804 | -4.803684 | 1.200677 |

| | | | | | |
|---|---|---|---|---|---|
| 37 | 6 | 0 | 3.698668 | 2.8047 | 0.203099 |
| 38 | 6 | 0 | 2.098128 | 2.724112 | -1.452598 |
| 39 | 6 | 0 | 3.97774 | 4.134823 | -0.083129 |
| 40 | 6 | 0 | 2.32961 | 4.051437 | -1.794995 |
| 41 | 6 | 0 | 3.293543 | 4.799409 | -1.10816 |
| 42 | 6 | 0 | -3.179448 | 2.599441 | 1.469703 |
| 43 | 6 | 0 | -1.590587 | 2.91565 | -0.18043 |
| 44 | 6 | 0 | -3.182225 | 3.954774 | 1.772977 |
| 45 | 6 | 0 | -1.549717 | 4.280089 | 0.067759 |
| 46 | 6 | 0 | -2.359598 | 4.840131 | 1.064997 |
| 47 | 6 | 0 | -3.829147 | -2.741174 | 0.276458 |
| 48 | 6 | 0 | -2.137578 | -2.801227 | -1.296442 |
| 49 | 6 | 0 | -4.133316 | -4.07686 | 0.050771 |
| 50 | 6 | 0 | -2.394928 | -4.136823 | -1.575331 |
| 51 | 6 | 0 | -3.417604 | -4.814915 | -0.899824 |
| 52 | 6 | 0 | 2.808667 | 0.733204 | 3.032294 |
| 53 | 6 | 0 | 0.775416 | 1.432696 | 2.210876 |
| 54 | 6 | 0 | 2.555795 | 1.32966 | 4.262962 |
| 55 | 6 | 0 | 0.461508 | 2.06493 | 3.407247 |
| 56 | 6 | 0 | 1.359509 | 2.022174 | 4.481729 |
| 57 | 6 | 0 | -2.748311 | -0.687279 | 3.039744 |
| 58 | 6 | 0 | -0.703142 | -1.329014 | 2.178999 |
| 59 | 6 | 0 | -2.450704 | -1.260299 | 4.269205 |
| 60 | 6 | 0 | -0.347359 | -1.940149 | 3.375388 |
| 61 | 6 | 0 | -1.226994 | -1.915261 | 4.464472 |
| 62 | 7 | 0 | -2.839216 | -2.098373 | -0.383003 |
| 63 | 7 | 0 | -2.386984 | 2.071094 | 0.510346 |
| 64 | 7 | 0 | -1.885997 | -0.708149 | 1.999262 |
| 65 | 7 | 0 | 1.929947 | 0.764857 | 2.005666 |
| 66 | 7 | 0 | 2.436702 | -2.034626 | 0.584482 |
| 67 | 7 | 0 | 2.764388 | 2.088552 | -0.46486 |
| 68 | 1 | 0 | 5.779885 | -1.270329 | -5.254661 |
| 69 | 1 | 0 | 7.833421 | -1.250494 | -3.864185 |
| 70 | 1 | 0 | 7.747153 | -0.82435 | -1.419037 |
| 71 | 1 | 0 | -0.082945 | -0.121882 | -4.57292 |
| 72 | 1 | 0 | -5.86468 | 1.01918 | -5.193701 |
| 73 | 1 | 0 | -7.91621 | 1.140105 | -3.793219 |
| 74 | 1 | 0 | -7.83113 | 0.872144 | -1.329867 |
| 75 | 1 | 0 | 1.066155 | -2.538039 | -0.874332 |
| 76 | 1 | 0 | 3.863505 | -1.782787 | 2.060206 |
| 77 | 1 | 0 | 3.978807 | -4.169282 | 2.692118 |
| 78 | 1 | 0 | 1.095175 | -4.968863 | -0.403559 |
| 79 | 1 | 0 | 4.246001 | 2.264182 | 0.965259 |
| 80 | 1 | 0 | 1.358166 | 2.139087 | -1.98317 |
| 81 | 1 | 0 | 4.744311 | 4.644182 | 0.492955 |
| 82 | 1 | 0 | 1.759741 | 4.493122 | -2.607333 |
| 83 | 1 | 0 | -3.846302 | 4.316352 | 2.551801 |
| 84 | 1 | 0 | -0.882455 | 4.901467 | -0.520846 |
| 85 | 1 | 0 | -0.973735 | 2.461873 | -0.94444 |
| 86 | 1 | 0 | -3.845615 | 1.905678 | 1.965916 |
| 87 | 1 | 0 | -4.393689 | -2.139157 | 0.97794 |
| 88 | 1 | 0 | -1.356906 | -2.265475 | -1.819219 |
| 89 | 1 | 0 | -4.94341 | -4.531732 | 0.612123 |
| 90 | 1 | 0 | -1.800125 | -4.6412 | -2.330629 |
| 91 | 1 | 0 | 3.756824 | 0.252711 | 2.820263 |

| | | | | | |
|---|---|---|---|---|---|
| 92 | 1 | 0 | 0.086087 | 1.445939 | 1.377238 |
| 93 | 1 | 0 | 3.307959 | 1.26272 | 5.043358 |
| 94 | 1 | 0 | -0.486516 | 2.587179 | 3.494538 |
| 95 | 1 | 0 | -3.710631 | -0.228261 | 2.839529 |
| 96 | 1 | 0 | -0.023703 | -1.316747 | 1.33607 |
| 97 | 1 | 0 | -3.184596 | -1.20633 | 5.067604 |
| 98 | 1 | 0 | 0.620085 | -2.426231 | 3.450682 |
| 99 | 6 | 0 | -2.373317 | 6.319975 | 1.33608 |
| 100 | 1 | 0 | -3.150719 | 6.806269 | 0.734373 |
| 101 | 1 | 0 | -2.593491 | 6.533174 | 2.38537 |
| 102 | 1 | 0 | -1.418818 | 6.785003 | 1.07641 |
| 103 | 6 | 0 | 3.604355 | 6.226866 | -1.472445 |
| 104 | 1 | 0 | 4.46939 | 6.269565 | -2.145213 |
| 105 | 1 | 0 | 2.765776 | 6.703862 | -1.986444 |
| 106 | 1 | 0 | 3.853681 | 6.819834 | -0.587957 |
| 107 | 6 | 0 | -3.754891 | -6.24944 | -1.203697 |
| 108 | 1 | 0 | -4.565819 | -6.298418 | -1.940405 |
| 109 | 1 | 0 | -2.898892 | -6.785158 | -1.621728 |
| 110 | 1 | 0 | -4.09653 | -6.777867 | -0.309589 |
| 111 | 6 | 0 | 2.62364 | -6.2752 | 1.507956 |
| 112 | 1 | 0 | 1.729561 | -6.807387 | 1.172831 |
| 113 | 1 | 0 | 3.485204 | -6.724147 | 0.999205 |
| 114 | 1 | 0 | 2.752824 | -6.453377 | 2.579293 |
| 115 | 6 | 0 | -0.891318 | -2.583211 | 5.771167 |
| 116 | 1 | 0 | -1.297754 | -2.026198 | 6.619851 |
| 117 | 1 | 0 | -1.324558 | -3.590159 | 5.807432 |
| 118 | 1 | 0 | 0.188652 | -2.68407 | 5.90517 |
| 119 | 6 | 0 | 1.069164 | 2.714304 | 5.787502 |
| 120 | 1 | 0 | 1.594927 | 2.23762 | 6.618939 |
| 121 | 1 | 0 | 1.398096 | 3.760003 | 5.750055 |
| 122 | 1 | 0 | -0.001588 | 2.717628 | 6.01025 |
| 123 | 1 | 0 | 2.832546 | -0.896701 | -4.881543 |

**Table S9.** Cartesian coordinates for $Ru^{II}Ru^{III}$-6pic-$2H^+$.

| Center number | Atomic number | Atomic type | Coordinates (Ångström) | | |
|---|---|---|---|---|---|
| | | | X | Y | Z |
| 1 | 6 | 0 | 4.549287 | -0.59098 | -2.10197 |
| 2 | 6 | 0 | 4.610068 | -0.76399 | -3.49842 |
| 3 | 6 | 0 | 5.836074 | -0.98393 | -4.12768 |
| 4 | 6 | 0 | 6.961186 | -1.00901 | -3.29837 |
| 5 | 6 | 0 | 6.887804 | -0.815 | -1.9029 |
| 6 | 6 | 0 | 5.668292 | -0.59091 | -1.26391 |
| 7 | 6 | 0 | 2.496024 | -0.42492 | -2.84678 |
| 8 | 6 | 0 | 5.582234 | -0.34258 | 0.225717 |
| 9 | 8 | 0 | 6.585994 | -0.46196 | 0.909963 |
| 10 | 8 | 0 | 4.424188 | 0.032262 | 0.771626 |
| 11 | 7 | 0 | 3.24222 | -0.40399 | -1.74991 |
| 12 | 7 | 0 | 3.285046 | -0.64831 | -3.94392 |
| 13 | 6 | 0 | 0.012296 | 0.004629 | -3.50633 |
| 14 | 6 | 0 | -1.0655 | 0.204613 | -2.64492 |

| | | | | | |
|---|---|---|---|---|---|
| 15 | 7 | 0 | 0.679249 | -0.09505 | -1.32219 |
| 16 | 7 | 0 | -0.6556 | 0.10592 | -1.32189 |
| 17 | 6 | 0 | 1.089965 | -0.19535 | -2.64654 |
| 18 | 6 | 0 | -4.53067 | 0.585739 | -2.11855 |
| 19 | 6 | 0 | -4.582 | 0.761475 | -3.51528 |
| 20 | 6 | 0 | -5.80385 | 0.97754 | -4.154 |
| 21 | 6 | 0 | -6.93553 | 0.995794 | -3.33384 |
| 22 | 6 | 0 | -6.87198 | 0.798602 | -1.93833 |
| 23 | 6 | 0 | -5.65685 | 0.57878 | -1.28979 |
| 24 | 6 | 0 | -2.47168 | 0.429964 | -2.84904 |
| 25 | 7 | 0 | -3.22498 | 0.40307 | -1.75681 |
| 26 | 7 | 0 | -3.25394 | 0.65239 | -3.95157 |
| 27 | 6 | 0 | -5.58484 | 0.32612 | 0.200782 |
| 28 | 8 | 0 | -6.59999 | 0.431229 | 0.871369 |
| 29 | 8 | 0 | -4.43018 | -0.03409 | 0.758382 |
| 30 | 44 | 0 | -2.50549 | -0.04387 | 0.037587 |
| 31 | 44 | 0 | 2.508484 | 0.045256 | 0.038703 |
| 32 | 6 | 0 | -3.22793 | 2.527661 | 1.516154 |
| 33 | 6 | 0 | -1.70541 | 2.932009 | -0.1699 |
| 34 | 6 | 0 | -3.29564 | 3.878949 | 1.826152 |
| 35 | 6 | 0 | -1.72743 | 4.296245 | 0.085288 |
| 36 | 6 | 0 | -2.53961 | 4.812627 | 1.104119 |
| 37 | 6 | 0 | -3.70989 | -2.81769 | 0.291986 |
| 38 | 6 | 0 | -2.1082 | -2.80204 | -1.36848 |
| 39 | 6 | 0 | -3.99423 | -4.15444 | 0.052642 |
| 40 | 6 | 0 | -2.34551 | -4.13872 | -1.66282 |
| 41 | 6 | 0 | -3.3138 | -4.85814 | -0.95045 |
| 42 | 6 | 0 | 3.206241 | -2.52618 | 1.528071 |
| 43 | 6 | 0 | 1.70222 | -2.92771 | -0.17612 |
| 44 | 6 | 0 | 3.266956 | -3.87728 | 1.839451 |
| 45 | 6 | 0 | 1.717247 | -4.29165 | 0.08089 |
| 46 | 6 | 0 | 2.516591 | -4.80935 | 1.109115 |
| 47 | 6 | 0 | 3.715945 | 2.818188 | 0.300615 |
| 48 | 6 | 0 | 2.11808 | 2.807215 | -1.36421 |
| 49 | 6 | 0 | 4.000975 | 4.155644 | 0.065422 |
| 50 | 6 | 0 | 2.355907 | 4.144513 | -1.65377 |
| 51 | 6 | 0 | 3.323148 | 4.861893 | -0.93737 |
| 52 | 6 | 0 | -2.77101 | -0.68696 | 3.04511 |
| 53 | 6 | 0 | -0.74662 | -1.39471 | 2.199674 |
| 54 | 6 | 0 | -2.49641 | -1.26071 | 4.279631 |
| 55 | 6 | 0 | -0.41215 | -2.00486 | 3.402031 |
| 56 | 6 | 0 | -1.29344 | -1.94843 | 4.489513 |
| 57 | 6 | 0 | 2.753882 | 0.695279 | 3.04708 |
| 58 | 6 | 0 | 0.730244 | 1.390373 | 2.188478 |
| 59 | 6 | 0 | 2.469425 | 1.270207 | 4.278528 |
| 60 | 6 | 0 | 0.386089 | 2.001613 | 3.387757 |
| 61 | 6 | 0 | 1.261547 | 1.952359 | 4.479984 |
| 62 | 7 | 0 | 2.782578 | 2.132421 | -0.4014 |
| 63 | 7 | 0 | 2.429436 | -2.03573 | 0.533208 |
| 64 | 7 | 0 | 1.895886 | 0.738188 | 2.002031 |
| 65 | 7 | 0 | -1.90761 | -0.73617 | 2.004941 |
| 66 | 7 | 0 | -2.43769 | 2.037925 | 0.531576 |
| 67 | 7 | 0 | -2.77434 | -2.12975 | -0.40545 |
| 68 | 1 | 0 | -5.88513 | 1.120214 | -5.22625 |
| 69 | 1 | 0 | -7.90504 | 1.160644 | -3.79244 |

| | | | | | |
|---|---|---|---|---|---|
| 70 | 1 | 0 | -7.77693 | 0.807366 | -1.34061 |
| 71 | 1 | 0 | 0.011516 | 0.003747 | -4.58656 |
| 72 | 1 | 0 | 5.925582 | -1.12453 | -5.19952 |
| 73 | 1 | 0 | 7.933501 | -1.17713 | -3.74975 |
| 74 | 1 | 0 | 7.788202 | -0.82989 | -1.29845 |
| 75 | 1 | 0 | -1.08056 | 2.520962 | -0.95186 |
| 76 | 1 | 0 | -3.84295 | 1.805163 | 2.034741 |
| 77 | 1 | 0 | -3.95823 | 4.199489 | 2.624087 |
| 78 | 1 | 0 | -1.10959 | 4.954879 | -0.51682 |
| 79 | 1 | 0 | -4.25219 | -2.25166 | 1.038412 |
| 80 | 1 | 0 | -1.3632 | -2.24198 | -1.91814 |
| 81 | 1 | 0 | -4.76172 | -4.63981 | 0.647443 |
| 82 | 1 | 0 | -1.77771 | -4.61333 | -2.45735 |
| 83 | 1 | 0 | 3.919236 | -4.19915 | 2.645284 |
| 84 | 1 | 0 | 1.103669 | -4.94891 | -0.52695 |
| 85 | 1 | 0 | 1.086771 | -2.51561 | -0.96482 |
| 86 | 1 | 0 | 3.815808 | -1.80472 | 2.054504 |
| 87 | 1 | 0 | 4.255443 | 2.250768 | 1.047997 |
| 88 | 1 | 0 | 1.373645 | 2.249106 | -1.91639 |
| 89 | 1 | 0 | 4.766443 | 4.639697 | 0.663792 |
| 90 | 1 | 0 | 1.788825 | 4.621599 | -2.44731 |
| 91 | 1 | 0 | -3.72303 | -0.21047 | 2.844881 |
| 92 | 1 | 0 | -0.06825 | -1.42167 | 1.357027 |
| 93 | 1 | 0 | -3.23546 | -1.18195 | 5.071076 |
| 94 | 1 | 0 | 0.538221 | -2.52274 | 3.483857 |
| 95 | 1 | 0 | 3.70912 | 0.222421 | 2.853622 |
| 96 | 1 | 0 | 0.055547 | 1.410784 | 1.342758 |
| 97 | 1 | 0 | 3.204284 | 1.19684 | 5.07439 |
| 98 | 1 | 0 | -0.56762 | 2.514193 | 3.463045 |
| 99 | 6 | 0 | 2.593469 | -6.28337 | 1.395427 |
| 100 | 1 | 0 | 3.495727 | -6.70956 | 0.940547 |
| 101 | 1 | 0 | 2.652848 | -6.47858 | 2.469715 |
| 102 | 1 | 0 | 1.732907 | -6.81983 | 0.989075 |
| 103 | 6 | 0 | -3.6298 | -6.29589 | -1.25774 |
| 104 | 1 | 0 | -4.57237 | -6.3663 | -1.81344 |
| 105 | 1 | 0 | -2.85072 | -6.762 | -1.86521 |
| 106 | 1 | 0 | -3.75476 | -6.87951 | -0.34106 |
| 107 | 6 | 0 | 3.641108 | 6.299393 | -1.24318 |
| 108 | 1 | 0 | 4.544551 | 6.363102 | -1.86154 |
| 109 | 1 | 0 | 2.831101 | 6.786229 | -1.7913 |
| 110 | 1 | 0 | 3.837046 | 6.867141 | -0.32939 |
| 111 | 6 | 0 | -2.62399 | 6.286865 | 1.387753 |
| 112 | 1 | 0 | -1.75138 | 6.822581 | 1.00667 |
| 113 | 1 | 0 | -3.51178 | 6.713665 | 0.905741 |
| 114 | 1 | 0 | -2.71499 | 6.482772 | 2.459619 |
| 115 | 6 | 0 | 0.943112 | 2.620403 | 5.789974 |
| 116 | 1 | 0 | 1.267976 | 2.009885 | 6.63718 |
| 117 | 1 | 0 | 1.468375 | 3.57999 | 5.865825 |
| 118 | 1 | 0 | -0.12632 | 2.818858 | 5.893167 |
| 119 | 6 | 0 | -0.98544 | -2.61643 | 5.802079 |
| 120 | 1 | 0 | -1.34078 | -2.01908 | 6.646249 |
| 121 | 1 | 0 | -1.48888 | -3.58883 | 5.861803 |
| 122 | 1 | 0 | 0.086208 | -2.79095 | 5.92496 |
| 123 | 1 | 0 | -2.93276 | 0.740971 | -4.90381 |

| | | | | | |
|---|---|---|---|---|---|
| 124 | 1 | 0 | 2.970171 | -0.73254 | -4.89882 |

**Table S10.** Cartesian coordinates for $Ru^{II}Ru^{III}$-6pic-$OH_2$-A.

| Center number | Atomic number | Atomic type | Coordinates (Ångström) | | |
|---|---|---|---|---|---|
| | | | X | Y | Z |
| 1 | 6 | 0 | 3.844168 | -0.9046 | -2.79785 |
| 2 | 6 | 0 | 3.25676 | -1.38518 | -3.99992 |
| 3 | 6 | 0 | 4.04722 | -1.91683 | -5.02534 |
| 4 | 6 | 0 | 5.428155 | -1.95814 | -4.84076 |
| 5 | 6 | 0 | 6.013261 | -1.45198 | -3.67103 |
| 6 | 6 | 0 | 5.246229 | -0.89962 | -2.63619 |
| 7 | 6 | 0 | 1.670712 | -0.70444 | -2.73995 |
| 8 | 6 | 0 | 5.945806 | -0.2507 | -1.49882 |
| 9 | 8 | 0 | 7.144765 | -0.3813 | -1.28268 |
| 10 | 8 | 0 | 5.199243 | 0.569978 | -0.74886 |
| 11 | 7 | 0 | 2.81441 | -0.48298 | -1.97518 |
| 12 | 7 | 0 | 1.894496 | -1.23963 | -3.93464 |
| 13 | 6 | 0 | -0.81041 | -0.28018 | -3.21261 |
| 14 | 6 | 0 | -1.92578 | -0.13567 | -2.37353 |
| 15 | 7 | 0 | -0.19868 | -0.41203 | -1.08976 |
| 16 | 7 | 0 | -1.53901 | -0.21663 | -1.06163 |
| 17 | 6 | 0 | 0.283183 | -0.44172 | -2.36267 |
| 18 | 6 | 0 | -5.32002 | 0.315129 | -1.69989 |
| 19 | 6 | 0 | -5.38409 | 0.400357 | -3.12419 |
| 20 | 6 | 0 | -6.64296 | 0.61855 | -3.70718 |
| 21 | 6 | 0 | -7.74601 | 0.736331 | -2.85803 |
| 22 | 6 | 0 | -7.64147 | 0.641919 | -1.45136 |
| 23 | 6 | 0 | -6.41229 | 0.424092 | -0.82599 |
| 24 | 6 | 0 | -3.35354 | 0.070953 | -2.5747 |
| 25 | 7 | 0 | -4.02362 | 0.113843 | -1.39275 |
| 26 | 7 | 0 | -4.11451 | 0.242485 | -3.66185 |
| 27 | 6 | 0 | -6.29143 | 0.3043 | 0.679529 |
| 28 | 8 | 0 | -7.30165 | 0.434828 | 1.372193 |
| 29 | 8 | 0 | -5.11549 | 0.045429 | 1.236913 |
| 30 | 44 | 0 | -3.19592 | -0.14877 | 0.386362 |
| 31 | 44 | 0 | 3.327271 | 0.270143 | -0.08878 |
| 32 | 6 | 0 | -3.53559 | 2.556364 | 1.690283 |
| 33 | 6 | 0 | -2.66485 | 2.744894 | -0.43494 |
| 34 | 6 | 0 | -3.60693 | 3.936445 | 1.825031 |
| 35 | 6 | 0 | -2.72109 | 4.132992 | -0.37196 |
| 36 | 6 | 0 | -3.20151 | 4.771484 | 0.77698 |
| 37 | 6 | 0 | -4.51764 | -2.81817 | 0.946813 |
| 38 | 6 | 0 | -2.86599 | -3.04466 | -0.64578 |
| 39 | 6 | 0 | -4.82291 | -4.17123 | 0.873098 |
| 40 | 6 | 0 | -3.12127 | -4.40525 | -0.7716 |
| 41 | 6 | 0 | -4.1226 | -5.0083 | -0.00362 |
| 42 | 6 | 0 | 5.039316 | -2.04296 | 0.942017 |
| 43 | 6 | 0 | 2.904576 | -2.69014 | 0.328892 |
| 44 | 6 | 0 | 5.371036 | -3.34482 | 1.293593 |
| 45 | 6 | 0 | 3.170767 | -4.01102 | 0.66262 |

| | | | | | |
|---|---|---|---|---|---|
| 46 | 6 | 0 | 4.430761 | -4.37248 | 1.155211 |
| 47 | 6 | 0 | 3.754748 | 3.058954 | -1.25238 |
| 48 | 6 | 0 | 1.551048 | 2.735657 | -0.6493 |
| 49 | 6 | 0 | 3.474478 | 4.339412 | -1.71374 |
| 50 | 6 | 0 | 1.203478 | 4.006657 | -1.08391 |
| 51 | 6 | 0 | 2.17425 | 4.849775 | -1.63861 |
| 52 | 6 | 0 | -3.28777 | -0.54888 | 3.421705 |
| 53 | 6 | 0 | -1.14114 | -0.874 | 2.653464 |
| 54 | 6 | 0 | -2.91447 | -0.88497 | 4.716716 |
| 55 | 6 | 0 | -0.69987 | -1.22796 | 3.925405 |
| 56 | 6 | 0 | -1.58998 | -1.23981 | 5.003965 |
| 57 | 6 | 0 | 5.182431 | 1.817446 | 1.846533 |
| 58 | 6 | 0 | 3.473222 | 0.762354 | 2.986115 |
| 59 | 6 | 0 | 5.695531 | 2.336706 | 3.028811 |
| 60 | 6 | 0 | 3.934535 | 1.247397 | 4.203352 |
| 61 | 6 | 0 | 5.069261 | 2.066458 | 4.251016 |
| 62 | 7 | 0 | 2.811268 | 2.25744 | -0.71482 |
| 63 | 7 | 0 | 3.821827 | -1.71139 | 0.465269 |
| 64 | 7 | 0 | 4.074336 | 1.047749 | 1.812061 |
| 65 | 7 | 0 | -2.41812 | -0.53365 | 2.381266 |
| 66 | 7 | 0 | -3.06082 | 1.945622 | 0.580838 |
| 67 | 7 | 0 | -3.54305 | -2.24467 | 0.204994 |
| 68 | 1 | 0 | -6.75519 | 0.691789 | -4.78545 |
| 69 | 1 | 0 | -8.7276 | 0.904749 | -3.29387 |
| 70 | 1 | 0 | -8.5234 | 0.734804 | -0.82578 |
| 71 | 1 | 0 | -0.78875 | -0.28479 | -4.28998 |
| 72 | 1 | 0 | 3.581595 | -2.2803 | -5.93641 |
| 73 | 1 | 0 | 6.064794 | -2.36764 | -5.61966 |
| 74 | 1 | 0 | 7.091015 | -1.44936 | -3.54632 |
| 75 | 1 | 0 | -2.31561 | 2.241037 | -1.32628 |
| 76 | 1 | 0 | -3.89369 | 1.900143 | 2.471564 |
| 77 | 1 | 0 | -4.00533 | 4.354001 | 2.74509 |
| 78 | 1 | 0 | -2.41398 | 4.710529 | -1.23975 |
| 79 | 1 | 0 | -5.07064 | -2.13935 | 1.585195 |
| 80 | 1 | 0 | -2.10087 | -2.56596 | -1.24272 |
| 81 | 1 | 0 | -5.62134 | -4.56359 | 1.496146 |
| 82 | 1 | 0 | -2.53881 | -4.98626 | -1.4806 |
| 83 | 1 | 0 | 6.370585 | -3.55088 | 1.662936 |
| 84 | 1 | 0 | 2.39188 | -4.75514 | 0.528754 |
| 85 | 1 | 0 | 1.941741 | -2.38762 | -0.06059 |
| 86 | 1 | 0 | 5.761573 | -1.23885 | 1.014573 |
| 87 | 1 | 0 | 4.747464 | 2.629111 | -1.31799 |
| 88 | 1 | 0 | 0.798295 | 2.066708 | -0.25239 |
| 89 | 1 | 0 | 4.279253 | 4.93168 | -2.13828 |
| 90 | 1 | 0 | 0.169759 | 4.323674 | -0.99026 |
| 91 | 1 | 0 | -4.31234 | -0.29272 | 3.162573 |
| 92 | 1 | 0 | -0.44794 | -0.8647 | 1.821174 |
| 93 | 1 | 0 | -3.66891 | -0.87682 | 5.498668 |
| 94 | 1 | 0 | 0.341998 | -1.50449 | 4.062816 |
| 95 | 1 | 0 | 5.672776 | 1.970462 | 0.892838 |
| 96 | 1 | 0 | 2.60337 | 0.118801 | 2.920284 |
| 97 | 1 | 0 | 6.593222 | 2.946024 | 2.987834 |
| 98 | 1 | 0 | 3.406548 | 0.978124 | 5.113103 |
| 99 | 8 | 0 | 1.568173 | -0.03931 | 0.869648 |
| 100 | 1 | 0 | 0.375689 | -0.36813 | -0.22889 |

| | | | | | |
|---|---|---|---|---|---|
| 101 | 1 | 0 | 1.273607 | 0.768438 | 1.312837 |
| 102 | 6 | 0 | 4.767807 | -5.80228 | 1.486412 |
| 103 | 1 | 0 | 5.134814 | -6.3227 | 0.593499 |
| 104 | 1 | 0 | 5.551001 | -5.86367 | 2.246707 |
| 105 | 1 | 0 | 3.890253 | -6.34762 | 1.844644 |
| 106 | 6 | 0 | 5.581198 | 2.638132 | 5.546754 |
| 107 | 1 | 0 | 5.150996 | 3.631464 | 5.72393 |
| 108 | 1 | 0 | 5.3106 | 2.007385 | 6.397732 |
| 109 | 1 | 0 | 6.668581 | 2.75205 | 5.530742 |
| 110 | 6 | 0 | -1.15773 | -1.61505 | 6.398359 |
| 111 | 1 | 0 | -1.76209 | -2.44068 | 6.789986 |
| 112 | 1 | 0 | -0.10882 | -1.92261 | 6.424578 |
| 113 | 1 | 0 | -1.28067 | -0.77337 | 7.089986 |
| 114 | 6 | 0 | -4.45661 | -6.47147 | -0.13852 |
| 115 | 1 | 0 | -5.3016 | -6.61243 | -0.82357 |
| 116 | 1 | 0 | -3.61244 | -7.04072 | -0.53767 |
| 117 | 1 | 0 | -4.7435 | -6.90653 | 0.82364 |
| 118 | 6 | 0 | -3.32311 | 6.270619 | 0.866767 |
| 119 | 1 | 0 | -2.65167 | 6.771952 | 0.163804 |
| 120 | 1 | 0 | -4.3451 | 6.589614 | 0.628393 |
| 121 | 1 | 0 | -3.09861 | 6.631008 | 1.875465 |
| 122 | 6 | 0 | 1.825386 | 6.218626 | -2.15957 |
| 123 | 1 | 0 | 1.408639 | 6.146368 | -3.17139 |
| 124 | 1 | 0 | 1.07254 | 6.70424 | -1.53191 |
| 125 | 1 | 0 | 2.705143 | 6.865196 | -2.2106 |

**Table S11.** Cartesian coordinates for $Ru^{II}Ru^{III}$-6pic-$H^+$-$OH_2$-A.

| Center number | Atomic number | Atomic type | Coordinates (Ångström) | | |
|---|---|---|---|---|---|
| | | | X | Y | Z |
| 1 | 6 | 0 | 3.784402 | -1.38437 | -2.6601 |
| 2 | 6 | 0 | 3.189744 | -2.05261 | -3.76442 |
| 3 | 6 | 0 | 3.9773 | -2.7128 | -4.71834 |
| 4 | 6 | 0 | 5.358833 | -2.69311 | -4.5561 |
| 5 | 6 | 0 | 5.950572 | -2.00879 | -3.48091 |
| 6 | 6 | 0 | 5.188665 | -1.3295 | -2.5245 |
| 7 | 6 | 0 | 1.617416 | -1.22564 | -2.58174 |
| 8 | 6 | 0 | 5.886233 | -0.52949 | -1.48481 |
| 9 | 8 | 0 | 7.081285 | -0.63107 | -1.249 |
| 10 | 8 | 0 | 5.135097 | 0.38431 | -0.85377 |
| 11 | 7 | 0 | 2.761111 | -0.8626 | -1.88525 |
| 12 | 7 | 0 | 1.82703 | -1.92749 | -3.69223 |
| 13 | 6 | 0 | -0.8754 | -1.06606 | -3.08408 |
| 14 | 6 | 0 | -1.98158 | -0.75339 | -2.28758 |
| 15 | 7 | 0 | -0.24552 | -0.58265 | -1.01578 |
| 16 | 7 | 0 | -1.59023 | -0.46037 | -1.00604 |
| 17 | 6 | 0 | 0.22851 | -0.9426 | -2.23668 |
| 18 | 6 | 0 | -5.4369 | -0.33704 | -1.72773 |
| 19 | 6 | 0 | -5.56469 | -0.69208 | -3.08695 |
| 20 | 6 | 0 | -6.82691 | -0.77437 | -3.67735 |
| 21 | 6 | 0 | -7.91707 | -0.49402 | -2.84939 |

| | | | | | |
|---|---|---|---|---|---|
| 22 | 6 | 0 | -7.77326 | -0.14744 | -1.48991 |
| 23 | 6 | 0 | -6.51893 | -0.06021 | -0.88516 |
| 24 | 6 | 0 | -3.41212 | -0.67472 | -2.48156 |
| 25 | 7 | 0 | -4.10802 | -0.33403 | -1.40888 |
| 26 | 7 | 0 | -4.25654 | -0.90028 | -3.54035 |
| 27 | 6 | 0 | -6.37395 | 0.313235 | 0.586268 |
| 28 | 8 | 0 | -7.39887 | 0.561998 | 1.214066 |
| 29 | 8 | 0 | -5.18013 | 0.37145 | 1.140633 |
| 30 | 44 | 0 | -3.2532 | 0.007507 | 0.362106 |
| 31 | 44 | 0 | 3.29707 | 0.163845 | -0.11912 |
| 32 | 6 | 0 | -4.01349 | 2.965338 | 0.434038 |
| 33 | 6 | 0 | -2.12367 | 2.628611 | -0.83963 |
| 34 | 6 | 0 | -3.97969 | 4.331623 | 0.187491 |
| 35 | 6 | 0 | -2.03154 | 3.985157 | -1.12859 |
| 36 | 6 | 0 | -2.97486 | 4.882894 | -0.61661 |
| 37 | 6 | 0 | -4.34659 | -2.3292 | 1.934185 |
| 38 | 6 | 0 | -2.96777 | -3.07194 | 0.240673 |
| 39 | 6 | 0 | -4.60155 | -3.63136 | 2.343879 |
| 40 | 6 | 0 | -3.18331 | -4.39798 | 0.594621 |
| 41 | 6 | 0 | -4.02076 | -4.71336 | 1.671356 |
| 42 | 6 | 0 | 5.050744 | -1.97321 | 1.178413 |
| 43 | 6 | 0 | 2.893425 | -2.70591 | 0.777096 |
| 44 | 6 | 0 | 5.409574 | -3.2068 | 1.703766 |
| 45 | 6 | 0 | 3.186457 | -3.96147 | 1.291769 |
| 46 | 6 | 0 | 4.472569 | -4.2459 | 1.767736 |
| 47 | 6 | 0 | 3.177927 | 2.265569 | -2.28883 |
| 48 | 6 | 0 | 2.265164 | 3.070105 | -0.32686 |
| 49 | 6 | 0 | 2.97155 | 3.483584 | -2.92289 |
| 50 | 6 | 0 | 2.024899 | 4.312345 | -0.8998 |
| 51 | 6 | 0 | 2.381945 | 4.550336 | -2.23362 |
| 52 | 6 | 0 | -3.19726 | 0.922438 | 3.270526 |
| 53 | 6 | 0 | -1.2215 | -0.12807 | 2.716156 |
| 54 | 6 | 0 | -2.77521 | 1.106835 | 4.580766 |
| 55 | 6 | 0 | -0.73887 | 0.015342 | 4.01316 |
| 56 | 6 | 0 | -1.51591 | 0.64904 | 4.989487 |
| 57 | 6 | 0 | 5.121072 | 2.014638 | 1.513192 |
| 58 | 6 | 0 | 3.552345 | 0.972735 | 2.858516 |
| 59 | 6 | 0 | 5.68275 | 2.655384 | 2.609788 |
| 60 | 6 | 0 | 4.067851 | 1.581999 | 3.995424 |
| 61 | 6 | 0 | 5.15942 | 2.454414 | 3.893691 |
| 62 | 7 | 0 | 2.833104 | 2.049711 | -0.99992 |
| 63 | 7 | 0 | 3.80793 | -1.71738 | 0.715234 |
| 64 | 7 | 0 | 4.059945 | 1.185681 | 1.6262 |
| 65 | 7 | 0 | -2.43371 | 0.318723 | 2.330217 |
| 66 | 7 | 0 | -3.0985 | 2.10279 | -0.06724 |
| 67 | 7 | 0 | -3.53389 | -2.03388 | 0.893259 |
| 68 | 1 | 0 | -6.96599 | -1.04335 | -4.71936 |
| 69 | 1 | 0 | -8.91596 | -0.54861 | -3.27082 |
| 70 | 1 | 0 | -8.64488 | 0.059257 | -0.87858 |
| 71 | 1 | 0 | -0.8344 | -1.35707 | -4.12144 |
| 72 | 1 | 0 | 3.508187 | -3.21947 | -5.55599 |
| 73 | 1 | 0 | 5.994322 | -3.1979 | -5.27718 |
| 74 | 1 | 0 | 7.029768 | -1.97145 | -3.37664 |
| 75 | 1 | 0 | -1.40003 | 1.926478 | -1.23295 |
| 76 | 1 | 0 | -4.79642 | 2.516236 | 1.032743 |

| | | | | | |
|---|---|---|---|---|---|
| 77 | 1 | 0 | -4.75108 | 4.960343 | 0.622043 |
| 78 | 1 | 0 | -1.21933 | 4.332819 | -1.75978 |
| 79 | 1 | 0 | -4.81352 | -1.4803 | 2.417963 |
| 80 | 1 | 0 | -2.31952 | -2.81837 | -0.58852 |
| 81 | 1 | 0 | -5.2657 | -3.7946 | 3.187142 |
| 82 | 1 | 0 | -2.69532 | -5.18192 | 0.02329 |
| 83 | 1 | 0 | 6.427493 | -3.35294 | 2.050567 |
| 84 | 1 | 0 | 2.408473 | -4.7181 | 1.31228 |
| 85 | 1 | 0 | 1.907108 | -2.46508 | 0.403172 |
| 86 | 1 | 0 | 5.770878 | -1.16834 | 1.095508 |
| 87 | 1 | 0 | 3.641395 | 1.433816 | -2.80273 |
| 88 | 1 | 0 | 2.018004 | 2.885999 | 0.712789 |
| 89 | 1 | 0 | 3.282463 | 3.595048 | -3.95683 |
| 90 | 1 | 0 | 1.574134 | 5.094389 | -0.29689 |
| 91 | 1 | 0 | -4.18717 | 1.220485 | 2.94351 |
| 92 | 1 | 0 | -0.61959 | -0.61253 | 1.956671 |
| 93 | 1 | 0 | -3.44212 | 1.598173 | 5.283115 |
| 94 | 1 | 0 | 0.242029 | -0.38203 | 4.257045 |
| 95 | 1 | 0 | 5.531216 | 2.114952 | 0.51533 |
| 96 | 1 | 0 | 2.715543 | 0.284885 | 2.90706 |
| 97 | 1 | 0 | 6.540116 | 3.303261 | 2.455981 |
| 98 | 1 | 0 | 3.621687 | 1.365161 | 4.961054 |
| 99 | 8 | 0 | 1.500154 | 0.009312 | 0.830444 |
| 100 | 1 | 0 | 0.359719 | -0.36132 | -0.17791 |
| 101 | 1 | 0 | 1.222916 | 0.873532 | 1.165686 |
| 102 | 6 | 0 | 4.840694 | -5.6074 | 2.292979 |
| 103 | 1 | 0 | 5.299324 | -6.20821 | 1.498418 |
| 104 | 1 | 0 | 5.566676 | -5.53817 | 3.107701 |
| 105 | 1 | 0 | 3.963214 | -6.15083 | 2.652391 |
| 106 | 6 | 0 | 5.736143 | 3.149855 | 5.097696 |
| 107 | 1 | 0 | 5.344818 | 4.171574 | 5.17396 |
| 108 | 1 | 0 | 5.48196 | 2.628422 | 6.02377 |
| 109 | 1 | 0 | 6.824896 | 3.224743 | 5.028737 |
| 110 | 6 | 0 | -1.04197 | 0.815919 | 6.409411 |
| 111 | 1 | 0 | -1.66362 | 0.232194 | 7.097633 |
| 112 | 1 | 0 | -0.0073 | 0.48575 | 6.531012 |
| 113 | 1 | 0 | -1.10881 | 1.861697 | 6.727322 |
| 114 | 6 | 0 | -4.30779 | -6.13797 | 2.065414 |
| 115 | 1 | 0 | -5.22021 | -6.49484 | 1.572417 |
| 116 | 1 | 0 | -3.49436 | -6.80756 | 1.773648 |
| 117 | 1 | 0 | -4.46443 | -6.23042 | 3.143657 |
| 118 | 6 | 0 | -2.93209 | 6.355439 | -0.92891 |
| 119 | 1 | 0 | -1.96863 | 6.650006 | -1.35287 |
| 120 | 1 | 0 | −3.70928 | 6.617799 | -1.65636 |
| 121 | 1 | 0 | -3.11431 | 6.957393 | -0.03341 |
| 122 | 6 | 0 | 2.135554 | 5.878923 | -2.89812 |
| 123 | 1 | 0 | 1.202334 | 5.854953 | -3.47406 |
| 124 | 1 | 0 | 2.053128 | 6.685772 | -2.16557 |
| 125 | 1 | 0 | 2.938424 | 6.127066 | -3.5975 |
| 126 | 1 | 0 | -3.98062 | -1.17161 | -4.4713 |

**Table S12.** Cartesian coordinates for $Ru^{II}Ru^{III}$-6pic-2$H^{+}$-$OH_2$.

| Center number | Atomic number | Atomic type | Coordinates (Ångström) | | |
|---|---|---|---|---|---|
| | | | X | Y | Z |
| 1 | 6 | 0 | 3.823298 | -0.81736 | -2.90924 |
| 2 | 6 | 0 | 3.274127 | -1.26009 | -4.12919 |
| 3 | 6 | 0 | 4.045642 | -1.7337 | -5.19155 |
| 4 | 6 | 0 | 5.422153 | -1.75256 | -4.99833 |
| 5 | 6 | 0 | 5.993286 | -1.29686 | -3.79769 |
| 6 | 6 | 0 | 5.222434 | -0.80968 | -2.74065 |
| 7 | 6 | 0 | 1.646776 | -0.63321 | -2.72837 |
| 8 | 6 | 0 | 5.93042 | -0.26219 | -1.54157 |
| 9 | 8 | 0 | 7.118294 | -0.45717 | -1.35829 |
| 10 | 8 | 0 | 5.215595 | 0.503994 | -0.7134 |
| 11 | 7 | 0 | 2.786543 | -0.44843 | -2.04336 |
| 12 | 7 | 0 | 1.909644 | -1.11741 | -3.97977 |
| 13 | 6 | 0 | -0.82898 | -0.23727 | -3.23189 |
| 14 | 6 | 0 | -1.94843 | -0.12925 | -2.39561 |
| 15 | 7 | 0 | -0.23373 | -0.39538 | -1.0976 |
| 16 | 7 | 0 | -1.56972 | -0.22758 | -1.07803 |
| 17 | 6 | 0 | 0.257594 | -0.39945 | -2.36767 |
| 18 | 6 | 0 | -5.375 | 0.352958 | -1.75997 |
| 19 | 6 | 0 | -5.49452 | 0.431667 | -3.16266 |
| 20 | 6 | 0 | -6.74214 | 0.649327 | -3.75281 |
| 21 | 6 | 0 | -7.82253 | 0.778883 | -2.87849 |
| 22 | 6 | 0 | -7.68712 | 0.696355 | -1.47491 |
| 23 | 6 | 0 | -6.45014 | 0.476618 | -0.8724 |
| 24 | 6 | 0 | -3.36588 | 0.08179 | -2.57849 |
| 25 | 7 | 0 | -4.06027 | 0.142736 | -1.45225 |
| 26 | 7 | 0 | -4.19748 | 0.254519 | -3.65876 |
| 27 | 6 | 0 | -6.31075 | 0.377787 | 0.640433 |
| 28 | 8 | 0 | -7.31229 | 0.550241 | 1.322427 |
| 29 | 8 | 0 | -5.13804 | 0.095132 | 1.18069 |
| 30 | 44 | 0 | -3.23927 | -0.12931 | 0.342001 |
| 31 | 44 | 0 | 3.346346 | 0.232106 | -0.06703 |
| 32 | 6 | 0 | -3.68187 | 2.587344 | 1.628285 |
| 33 | 6 | 0 | -2.44502 | 2.792462 | -0.30302 |
| 34 | 6 | 0 | -3.71124 | 3.964483 | 1.799589 |
| 35 | 6 | 0 | -2.44463 | 4.178989 | -0.20046 |
| 36 | 6 | 0 | -3.09228 | 4.809301 | 0.869532 |
| 37 | 6 | 0 | -4.62963 | -2.77138 | 0.942302 |
| 38 | 6 | 0 | -2.90833 | -3.09018 | -0.55589 |
| 39 | 6 | 0 | -4.94475 | -4.12303 | 0.919997 |
| 40 | 6 | 0 | -3.1702 | -4.45297 | -0.6279 |
| 41 | 6 | 0 | -4.21513 | -5.0107 | 0.118397 |
| 42 | 6 | 0 | 5.067091 | -2.1018 | 0.888622 |
| 43 | 6 | 0 | 2.914158 | -2.76092 | 0.346091 |
| 44 | 6 | 0 | 5.416299 | -3.40131 | 1.225193 |
| 45 | 6 | 0 | 3.199402 | -4.08032 | 0.66864 |
| 46 | 6 | 0 | 4.478523 | -4.43789 | 1.117226 |
| 47 | 6 | 0 | 3.810644 | 2.996084 | -1.32785 |
| 48 | 6 | 0 | 1.794558 | 2.910841 | -0.20636 |
| 49 | 6 | 0 | 3.604264 | 4.325273 | -1.66798 |
| 50 | 6 | 0 | 1.527919 | 4.239718 | -0.50774 |
| 51 | 6 | 0 | 2.441708 | 4.991749 | -1.25677 |
| 52 | 6 | 0 | -3.32451 | -0.46338 | 3.388352 |

| | | | | | |
|---|---|---|---|---|---|
| 53 | 6 | 0 | -1.22458 | -0.96263 | 2.587527 |
| 54 | 6 | 0 | -2.95164 | -0.8011 | 4.68298 |
| 55 | 6 | 0 | -0.78461 | -1.3238 | 3.856205 |
| 56 | 6 | 0 | -1.65162 | -1.24714 | 4.953043 |
| 57 | 6 | 0 | 5.051584 | 1.824518 | 1.881164 |
| 58 | 6 | 0 | 3.592493 | 0.405088 | 2.980883 |
| 59 | 6 | 0 | 5.569178 | 2.278182 | 3.086029 |
| 60 | 6 | 0 | 4.066301 | 0.816772 | 4.218822 |
| 61 | 6 | 0 | 5.078432 | 1.783191 | 4.302159 |
| 62 | 7 | 0 | 2.922622 | 2.282598 | -0.59697 |
| 63 | 7 | 0 | 3.830193 | -1.77554 | 0.447166 |
| 64 | 7 | 0 | 4.063592 | 0.904317 | 1.816884 |
| 65 | 7 | 0 | -2.47602 | -0.52925 | 2.335159 |
| 66 | 7 | 0 | -3.05408 | 1.985099 | 0.590933 |
| 67 | 7 | 0 | -3.62 | -2.23978 | 0.213228 |
| 68 | 1 | 0 | -6.87656 | 0.715197 | -4.82726 |
| 69 | 1 | 0 | -8.80964 | 0.949066 | -3.29618 |
| 70 | 1 | 0 | -8.55425 | 0.802299 | -0.83217 |
| 71 | 1 | 0 | -0.80877 | -0.16969 | -4.30953 |
| 72 | 1 | 0 | 3.595748 | -2.06986 | -6.11987 |
| 73 | 1 | 0 | 6.068486 | -2.11307 | -5.79138 |
| 74 | 1 | 0 | 7.069947 | -1.29762 | -3.66755 |
| 75 | 1 | 0 | -1.95456 | 2.301404 | -1.13443 |
| 76 | 1 | 0 | -4.20365 | 1.931049 | 2.310387 |
| 77 | 1 | 0 | -4.24228 | 4.373596 | 2.653507 |
| 78 | 1 | 0 | -1.95623 | 4.764599 | -0.97383 |
| 79 | 1 | 0 | -5.19523 | -2.06682 | 1.539017 |
| 80 | 1 | 0 | -2.09905 | -2.65334 | -1.12758 |
| 81 | 1 | 0 | -5.77062 | -4.47707 | 1.529342 |
| 82 | 1 | 0 | -2.55726 | -5.07651 | -1.27164 |
| 83 | 1 | 0 | 6.427294 | -3.59935 | 1.566412 |
| 84 | 1 | 0 | 2.420059 | -4.82895 | 0.568255 |
| 85 | 1 | 0 | 1.929513 | -2.46224 | 0.01022 |
| 86 | 1 | 0 | 5.78636 | -1.29433 | 0.950368 |
| 87 | 1 | 0 | 4.710251 | 2.469205 | -1.61943 |
| 88 | 1 | 0 | 1.08656 | 2.320761 | 0.361095 |
| 89 | 1 | 0 | 4.362023 | 4.83967 | -2.25073 |
| 90 | 1 | 0 | 0.603288 | 4.680599 | -0.14976 |
| 91 | 1 | 0 | -4.33687 | -0.15334 | 3.150379 |
| 92 | 1 | 0 | -0.55472 | -1.03035 | 1.738579 |
| 93 | 1 | 0 | -3.68831 | -0.72516 | 5.477132 |
| 94 | 1 | 0 | 0.233514 | -1.68065 | 3.981917 |
| 95 | 1 | 0 | 5.446954 | 2.16546 | 0.933632 |
| 96 | 1 | 0 | 2.824358 | -0.35517 | 2.897427 |
| 97 | 1 | 0 | 6.367683 | 3.013073 | 3.069017 |
| 98 | 1 | 0 | 3.650598 | 0.372672 | 5.117595 |
| 99 | 8 | 0 | 1.526737 | -0.06441 | 0.76054 |
| 100 | 1 | 0 | 0.366084 | -0.31628 | -0.2238 |
| 101 | 1 | 0 | 1.336451 | 0.55168 | 1.480846 |
| 102 | 6 | 0 | 4.838471 | -5.86012 | 1.446376 |
| 103 | 1 | 0 | 5.423335 | -6.30055 | 0.630073 |
| 104 | 1 | 0 | 5.454596 | -5.91487 | 2.348306 |
| 105 | 1 | 0 | 3.950566 | -6.47933 | 1.591942 |
| 106 | 6 | 0 | 5.606403 | 2.271403 | 5.622636 |
| 107 | 1 | 0 | 5.145876 | 3.231964 | 5.88337 |

| | | | | | |
|---|---|---|---|---|---|
| 108 | 1 | 0 | 5.387424 | 1.568713 | 6.429701 |
| 109 | 1 | 0 | 6.687226 | 2.431717 | 5.582838 |
| 110 | 6 | 0 | -1.22393 | -1.63004 | 6.344493 |
| 111 | 1 | 0 | -1.86769 | -2.4193 | 6.746712 |
| 112 | 1 | 0 | -0.19243 | -1.98948 | 6.36685 |
| 113 | 1 | 0 | -1.30343 | -0.77612 | 7.026122 |
| 114 | 6 | 0 | -4.55287 | -6.47567 | 0.054182 |
| 115 | 1 | 0 | -5.49158 | -6.62889 | -0.49068 |
| 116 | 1 | 0 | -3.77396 | -7.04881 | -0.45402 |
| 117 | 1 | 0 | -4.6927 | -6.89317 | 1.05597 |
| 118 | 6 | 0 | -3.15331 | 6.307184 | 1.001945 |
| 119 | 1 | 0 | -2.46588 | 6.803889 | 0.312905 |
| 120 | 1 | 0 | -4.16423 | 6.669139 | 0.78195 |
| 121 | 1 | 0 | -2.91466 | 6.625996 | 2.021236 |
| 122 | 6 | 0 | 2.191194 | 6.430691 | -1.61552 |
| 123 | 1 | 0 | 1.933847 | 6.521827 | -2.67736 |
| 124 | 1 | 0 | 1.371245 | 6.855877 | -1.03269 |
| 125 | 1 | 0 | 3.085101 | 7.039177 | -1.44992 |
| 126 | 1 | 0 | 1.205341 | -1.42352 | -4.63396 |
| 127 | 1 | 0 | -3.93006 | 0.252566 | -4.63086 |

**Table S13.** Cartesian coordinates for $Ru^{II}Ru^{III}$-4pic-OH-$OH_2$-A.

| Center number | Atomic number | Atomic type | Coordinates (Ångström) | | |
|---|---|---|---|---|---|
| | | | X | Y | Z |
| 1 | 6 | 0 | -4.44808 | 0.681825 | 1.420689 |
| 2 | 6 | 0 | -4.59633 | 0.662895 | 2.838 |
| 3 | 6 | 0 | -5.89338 | 0.796009 | 3.356015 |
| 4 | 6 | 0 | -6.95372 | 0.912575 | 2.451125 |
| 5 | 6 | 0 | -6.76876 | 0.892894 | 1.051785 |
| 6 | 6 | 0 | -5.49315 | 0.771416 | 0.495153 |
| 7 | 6 | 0 | -2.52527 | 0.415777 | 2.392528 |
| 8 | 6 | 0 | -5.25832 | 0.701917 | -0.9909 |
| 9 | 8 | 0 | -6.20207 | 0.732398 | -1.77176 |
| 10 | 8 | 0 | -4.00265 | 0.623348 | -1.44464 |
| 11 | 7 | 0 | -3.12644 | 0.556327 | 1.175151 |
| 12 | 7 | 0 | -3.35395 | 0.483595 | 3.435419 |
| 13 | 6 | 0 | -0.02364 | -0.07271 | 3.108391 |
| 14 | 6 | 0 | 1.065835 | -0.26276 | 2.245861 |
| 15 | 7 | 0 | -0.66839 | 0.067388 | 0.944653 |
| 16 | 7 | 0 | 0.653967 | -0.14933 | 0.92891 |
| 17 | 6 | 0 | -1.10304 | 0.1451 | 2.253269 |
| 18 | 6 | 0 | 4.478436 | -0.70162 | 1.480855 |
| 19 | 6 | 0 | 4.64604 | -0.81595 | 2.877404 |
| 20 | 6 | 0 | 5.918484 | -1.00744 | 3.41523 |
| 21 | 6 | 0 | 6.976988 | -1.06612 | 2.501527 |
| 22 | 6 | 0 | 6.791168 | -0.93522 | 1.111014 |
| 23 | 6 | 0 | 5.524875 | -0.74271 | 0.55362 |
| 24 | 6 | 0 | 2.482284 | -0.50154 | 2.371692 |
| 25 | 7 | 0 | 3.149057 | -0.52417 | 1.224899 |
| 26 | 7 | 0 | 3.360084 | -0.68317 | 3.419036 |

| | | | | | |
|---|---|---|---|---|---|
| 27 | 6 | 0 | 5.340349 | -0.5847 | -0.96069 |
| 28 | 8 | 0 | 6.349659 | -0.69357 | -1.65607 |
| 29 | 8 | 0 | 4.152064 | -0.34581 | -1.45944 |
| 30 | 44 | 0 | 2.26563 | -0.19898 | -0.5027 |
| 31 | 44 | 0 | -2.21315 | 0.207452 | -0.57444 |
| 32 | 6 | 0 | 3.273496 | 2.477262 | -1.35427 |
| 33 | 6 | 0 | 2.292444 | 2.633038 | 0.72802 |
| 34 | 6 | 0 | 3.589686 | 3.828938 | -1.34438 |
| 35 | 6 | 0 | 2.583244 | 3.990293 | 0.805346 |
| 36 | 6 | 0 | 3.252458 | 4.627604 | -0.24473 |
| 37 | 6 | 0 | 2.715645 | -2.85061 | -1.88521 |
| 38 | 6 | 0 | 1.099667 | -3.03908 | -0.24883 |
| 39 | 6 | 0 | 2.558051 | -4.18433 | -2.24161 |
| 40 | 6 | 0 | 0.893853 | -4.37916 | -0.55341 |
| 41 | 6 | 0 | 1.63129 | -4.99285 | -1.57276 |
| 42 | 6 | 0 | -3.60817 | -2.31906 | -1.42204 |
| 43 | 6 | 0 | -2.61168 | -2.60488 | 0.643474 |
| 44 | 6 | 0 | -4.11943 | -3.61019 | -1.39404 |
| 45 | 6 | 0 | -3.09763 | -3.90334 | 0.738325 |
| 46 | 6 | 0 | -3.87603 | -4.44089 | -0.29358 |
| 47 | 6 | 0 | -1.75886 | 2.681749 | -2.17671 |
| 48 | 6 | 0 | -1.52939 | 3.121132 | 0.082025 |
| 49 | 6 | 0 | -1.55783 | 4.019581 | -2.49256 |
| 50 | 6 | 0 | -1.32525 | 4.472399 | -0.16391 |
| 51 | 6 | 0 | -1.33878 | 4.957241 | -1.47763 |
| 52 | 7 | 0 | -1.74098 | 2.231592 | -0.90677 |
| 53 | 7 | 0 | -2.85499 | -1.81359 | -0.41997 |
| 54 | 7 | 0 | 2.628742 | 1.869696 | -0.332 |
| 55 | 7 | 0 | 1.995089 | -2.26484 | -0.89924 |
| 56 | 1 | 0 | 6.090964 | -1.10248 | 4.482772 |
| 57 | 1 | 0 | 7.98314 | -1.21314 | 2.882892 |
| 58 | 1 | 0 | 7.636804 | -0.97801 | 0.433202 |
| 59 | 1 | 0 | -0.04341 | -0.08021 | 4.187804 |
| 60 | 1 | 0 | -6.07018 | 0.798223 | 4.427791 |
| 61 | 1 | 0 | -7.96414 | 1.011017 | 2.838669 |
| 62 | 1 | 0 | -7.61895 | 0.963658 | 0.38121 |
| 63 | 1 | 0 | 1.766321 | 2.131912 | 1.53103 |
| 64 | 1 | 0 | 3.55167 | 1.833104 | -2.17789 |
| 65 | 1 | 0 | 4.111823 | 4.251154 | -2.19789 |
| 66 | 1 | 0 | 2.283833 | 4.544154 | 1.690315 |
| 67 | 1 | 0 | 3.44886 | -2.21023 | -2.36436 |
| 68 | 1 | 0 | 0.528977 | -2.54618 | 0.527193 |
| 69 | 1 | 0 | 3.170164 | -4.58835 | -3.04263 |
| 70 | 1 | 0 | 0.148208 | -4.93651 | 0.005854 |
| 71 | 1 | 0 | -4.72317 | -3.95452 | -2.22801 |
| 72 | 1 | 0 | -2.87581 | -4.48585 | 1.62746 |
| 73 | 1 | 0 | -2.01933 | -2.16911 | 1.438555 |
| 74 | 1 | 0 | -3.82507 | -1.63684 | -2.23671 |
| 75 | 1 | 0 | -1.9368 | 1.930306 | -2.93601 |
| 76 | 1 | 0 | -1.5385 | 2.724734 | 1.089556 |
| 77 | 1 | 0 | -1.58081 | 4.324001 | -3.5343 |
| 78 | 1 | 0 | -1.16295 | 5.143109 | 0.674278 |
| 79 | 8 | 0 | 1.325061 | 0.285682 | -2.40914 |
| 80 | 6 | 0 | 3.617239 | 6.088558 | -0.18798 |
| 81 | 1 | 0 | 3.021484 | 6.623069 | 0.557027 |

| | | | | | |
|---|---|---|---|---|---|
| 82 | 1 | 0 | 4.672989 | 6.213998 | 0.08168 |
| 83 | 1 | 0 | 3.473264 | 6.573738 | -1.15834 |
| 84 | 6 | 0 | 1.459201 | -6.45037 | -1.91322 |
| 85 | 1 | 0 | 2.187648 | -7.06352 | -1.36839 |
| 86 | 1 | 0 | 0.461896 | -6.80983 | -1.64451 |
| 87 | 1 | 0 | 1.616076 | -6.6323 | -2.9803 |
| 88 | 6 | 0 | -4.45703 | -5.82807 | -0.20938 |
| 89 | 1 | 0 | -5.48706 | -5.78754 | 0.164841 |
| 90 | 1 | 0 | -4.48701 | -6.30889 | -1.19143 |
| 91 | 1 | 0 | -3.88469 | -6.46338 | 0.471877 |
| 92 | 6 | 0 | -1.1592 | 6.422552 | -1.77896 |
| 93 | 1 | 0 | -2.11917 | 6.948905 | -1.71258 |
| 94 | 1 | 0 | -0.47794 | 6.896526 | -1.06632 |
| 95 | 1 | 0 | -0.76877 | 6.578502 | -2.78823 |
| 96 | 1 | 0 | 1.725423 | -0.20995 | -3.13493 |
| 97 | 8 | 0 | -1.1404 | -0.26546 | -2.24742 |
| 98 | 1 | 0 | -1.31607 | -1.17542 | -2.52145 |
| 99 | 1 | 0 | 0.322206 | 0.059818 | -2.38087 |
| 100 | 1 | 0 | 3.113804 | -0.72067 | 4.394914 |

**Table S14.** Cartesian coordinates for $Ru^{II}Ru^{III}$-4pic-2$OH_2$-A.

| Center number | Atomic number | Atomic type | Coordinates (Ångström) | | |
|---|---|---|---|---|---|
| | | | X | Y | Z |
| 1 | 6 | 0 | 4.459001 | 0.628465 | 1.471584 |
| 2 | 6 | 0 | 4.643841 | 0.757374 | 2.863291 |
| 3 | 6 | 0 | 5.927654 | 0.927759 | 3.384829 |
| 4 | 6 | 0 | 6.973069 | 0.947944 | 2.45728 |
| 5 | 6 | 0 | 6.769162 | 0.79946 | 1.068914 |
| 6 | 6 | 0 | 5.493954 | 0.628675 | 0.529722 |
| 7 | 6 | 0 | 2.469864 | 0.48723 | 2.387071 |
| 8 | 6 | 0 | 5.287316 | 0.446502 | -0.97767 |
| 9 | 8 | 0 | 6.28274 | 0.522124 | -1.6903 |
| 10 | 8 | 0 | 4.08452 | 0.216355 | -1.45907 |
| 11 | 7 | 0 | 3.122599 | 0.479532 | 1.23239 |
| 12 | 7 | 0 | 3.36208 | 0.659448 | 3.422866 |
| 13 | 6 | 0 | -0.04034 | 0.082127 | 3.126566 |
| 14 | 6 | 0 | -1.12383 | -0.11915 | 2.266911 |
| 15 | 7 | 0 | 0.627257 | 0.162475 | 0.943266 |
| 16 | 7 | 0 | -0.69327 | -0.03549 | 0.955041 |
| 17 | 6 | 0 | 1.046265 | 0.269016 | 2.264118 |
| 18 | 6 | 0 | -4.49543 | -0.71418 | 1.44192 |
| 19 | 6 | 0 | -4.69099 | -0.78886 | 2.834676 |
| 20 | 6 | 0 | -5.96685 | -1.01368 | 3.352536 |
| 21 | 6 | 0 | -7.0028 | -1.13919 | 2.421018 |
| 22 | 6 | 0 | -6.79453 | -1.03683 | 1.030703 |
| 23 | 6 | 0 | -5.5221 | -0.81724 | 0.501736 |
| 24 | 6 | 0 | -2.53291 | -0.40305 | 2.367265 |
| 25 | 7 | 0 | -3.16749 | -0.4983 | 1.205214 |
| 26 | 7 | 0 | -3.42159 | -0.58328 | 3.395924 |
| 27 | 6 | 0 | -5.28466 | -0.66137 | -0.98614 |

| | | | | | |
|---|---|---|---|---|---|
| 28 | 8 | 0 | -6.23509 | -0.70516 | -1.75068 |
| 29 | 8 | 0 | -4.04228 | -0.48805 | -1.42507 |
| 30 | 44 | 0 | -2.23853 | -0.11763 | -0.56561 |
| 31 | 44 | 0 | 2.230727 | 0.1493 | -0.48291 |
| 32 | 6 | 0 | -2.03742 | -2.55084 | -2.26224 |
| 33 | 6 | 0 | -1.34461 | -3.03804 | -0.11014 |
| 34 | 6 | 0 | -1.83677 | -3.86489 | -2.66036 |
| 35 | 6 | 0 | -1.12571 | -4.36874 | -0.44168 |
| 36 | 6 | 0 | -1.3754 | -4.8183 | -1.74423 |
| 37 | 6 | 0 | -3.63647 | 2.443165 | -1.33849 |
| 38 | 6 | 0 | -2.45821 | 2.746139 | 0.626246 |
| 39 | 6 | 0 | -4.0925 | 3.754168 | -1.30758 |
| 40 | 6 | 0 | -2.88461 | 4.064792 | 0.722046 |
| 41 | 6 | 0 | -3.72743 | 4.606188 | -0.25698 |
| 42 | 6 | 0 | 2.748848 | 2.73752 | -1.96005 |
| 43 | 6 | 0 | 1.277361 | 3.075143 | -0.21643 |
| 44 | 6 | 0 | 2.682623 | 4.076906 | -2.3199 |
| 45 | 6 | 0 | 1.170307 | 4.427768 | -0.51794 |
| 46 | 6 | 0 | 1.882692 | 4.969193 | -1.59446 |
| 47 | 6 | 0 | 3.169768 | -2.5653 | -1.31564 |
| 48 | 6 | 0 | 2.317852 | -2.66385 | 0.824195 |
| 49 | 6 | 0 | 3.517871 | -3.90876 | -1.27534 |
| 50 | 6 | 0 | 2.645236 | -4.0098 | 0.935686 |
| 51 | 6 | 0 | 3.26939 | -4.67218 | -0.1282 |
| 52 | 7 | 0 | 2.573392 | -1.93234 | -0.27969 |
| 53 | 7 | 0 | 2.051108 | 2.221746 | -0.91907 |
| 54 | 7 | 0 | -1.79512 | -2.13459 | -1.00052 |
| 55 | 7 | 0 | -2.82466 | 1.931492 | -0.38374 |
| 56 | 1 | 0 | -6.15897 | -1.08361 | 4.418059 |
| 57 | 1 | 0 | -8.00973 | -1.31354 | 2.786255 |
| 58 | 1 | 0 | -7.62712 | -1.12135 | 0.340799 |
| 59 | 1 | 0 | -0.03894 | 0.080194 | 4.206742 |
| 60 | 1 | 0 | 6.116651 | 1.034531 | 4.448013 |
| 61 | 1 | 0 | 7.986892 | 1.077081 | 2.823316 |
| 62 | 1 | 0 | 7.609859 | 0.811068 | 0.383852 |
| 63 | 1 | 0 | -1.15455 | -2.6706 | 0.890687 |
| 64 | 1 | 0 | -2.40388 | -1.79679 | -2.94671 |
| 65 | 1 | 0 | -2.04917 | -4.13998 | -3.6886 |
| 66 | 1 | 0 | -0.75865 | -5.04988 | 0.319077 |
| 67 | 1 | 0 | -3.93809 | 1.755974 | -2.12115 |
| 68 | 1 | 0 | -1.80237 | 2.317001 | 1.374183 |
| 69 | 1 | 0 | -4.74406 | 4.100764 | -2.10357 |
| 70 | 1 | 0 | -2.55843 | 4.665744 | 1.565388 |
| 71 | 1 | 0 | 3.271853 | 4.421167 | -3.1645 |
| 72 | 1 | 0 | 0.528713 | 5.0559 | 0.092623 |
| 73 | 1 | 0 | 0.726467 | 2.641095 | 0.608279 |
| 74 | 1 | 0 | 3.399284 | 2.039369 | -2.47553 |
| 75 | 1 | 0 | 3.389718 | -1.95028 | -2.17757 |
| 76 | 1 | 0 | 1.834631 | -2.14502 | 1.643358 |
| 77 | 1 | 0 | 4.000859 | -4.35024 | -2.14169 |
| 78 | 1 | 0 | 2.41861 | -4.53511 | 1.858907 |
| 79 | 8 | 0 | -1.19438 | 0.354036 | -2.2023 |
| 80 | 6 | 0 | -1.18315 | -6.25818 | -2.13937 |
| 81 | 1 | 0 | -0.55128 | -6.79153 | -1.42505 |
| 82 | 1 | 0 | -2.14926 | -6.77509 | -2.1798 |

| | | | | | |
|---|---|---|---|---|---|
| 83 | 1 | 0 | -0.73152 | -6.33973 | -3.13227 |
| 84 | 6 | 0 | -4.23845 | 6.019344 | -0.17618 |
| 85 | 1 | 0 | -5.27463 | 6.028818 | 0.182198 |
| 86 | 1 | 0 | -3.64386 | 6.625449 | 0.511457 |
| 87 | 1 | 0 | -4.23317 | 6.500118 | -1.15864 |
| 88 | 6 | 0 | 1.818144 | 6.432636 | -1.94293 |
| 89 | 1 | 0 | 2.746127 | 6.938682 | -1.65199 |
| 90 | 1 | 0 | 1.697347 | 6.578793 | -3.02072 |
| 91 | 1 | 0 | 0.991953 | 6.933242 | -1.43192 |
| 92 | 6 | 0 | 3.682555 | -6.11734 | -0.03575 |
| 93 | 1 | 0 | 4.747241 | -6.19548 | 0.215068 |
| 94 | 1 | 0 | 3.122907 | -6.64896 | 0.738425 |
| 95 | 1 | 0 | 3.537891 | -6.63594 | -0.98797 |
| 96 | 1 | 0 | -1.32034 | 1.284127 | -2.43746 |
| 97 | 8 | 0 | 1.281507 | -0.34675 | -2.38652 |
| 98 | 1 | 0 | 1.717503 | 0.07824 | -3.13693 |
| 99 | 1 | 0 | 0.309088 | -0.07488 | -2.38453 |
| 100 | 1 | 0 | -3.19802 | -0.57485 | 4.379029 |
| 101 | 1 | 0 | 3.133837 | 0.730196 | 4.401618 |

**Table S15.** Cartesian coordinates for $Ru^{II}Ru^{III}$-4pic-$H^{+}$-$2OH_2$.

| Center number | Atomic number | Atomic type | Coordinates (Ångström) | | |
|---|---|---|---|---|---|
| | | | X | Y | Z |
| 1 | 6 | 0 | -4.48417 | -0.06863 | 1.414334 |
| 2 | 6 | 0 | -4.68261 | -0.08364 | 2.810172 |
| 3 | 6 | 0 | -5.97745 | -0.121 | 3.332834 |
| 4 | 6 | 0 | -7.01714 | -0.14316 | 2.400804 |
| 5 | 6 | 0 | -6.79946 | -0.12911 | 1.004711 |
| 6 | 6 | 0 | -5.51504 | -0.09051 | 0.464982 |
| 7 | 6 | 0 | -2.4916 | -0.02464 | 2.345327 |
| 8 | 6 | 0 | -5.29637 | -0.074 | -1.04541 |
| 9 | 8 | 0 | -6.27799 | -0.10835 | -1.77078 |
| 10 | 8 | 0 | -4.06387 | -0.01742 | -1.5365 |
| 11 | 7 | 0 | -3.13984 | -0.03176 | 1.183995 |
| 12 | 7 | 0 | -3.39941 | -0.05561 | 3.374926 |
| 13 | 6 | 0 | 0.050255 | -0.02374 | 3.081716 |
| 14 | 6 | 0 | 1.151909 | -0.01825 | 2.23027 |
| 15 | 7 | 0 | -0.62702 | 0.015096 | 0.897405 |
| 16 | 7 | 0 | 0.711318 | 0.00324 | 0.901719 |
| 17 | 6 | 0 | -1.05375 | -0.00465 | 2.215593 |
| 18 | 6 | 0 | 4.567425 | 0.000789 | 1.369056 |
| 19 | 6 | 0 | 4.797448 | -0.02416 | 2.757364 |
| 20 | 6 | 0 | 6.106512 | -0.03054 | 3.242323 |
| 21 | 6 | 0 | 7.122917 | -0.0122 | 2.281317 |
| 22 | 6 | 0 | 6.87074 | 0.010002 | 0.892702 |
| 23 | 6 | 0 | 5.567652 | 0.018157 | 0.392417 |
| 24 | 6 | 0 | 2.590547 | -0.02108 | 2.342856 |
| 25 | 7 | 0 | 3.22137 | 0.00319 | 1.170556 |
| 26 | 7 | 0 | 3.520376 | -0.03739 | 3.347372 |
| 27 | 6 | 0 | 5.281392 | 0.029699 | -1.09474 |

| | | | | | |
|---|---|---|---|---|---|
| 28 | 8 | 0 | 6.20468 | -0.00169 | -1.88472 |
| 29 | 8 | 0 | 4.008932 | 0.072889 | -1.54393 |
| 30 | 44 | 0 | 2.268524 | 0.020231 | -0.53513 |
| 31 | 44 | 0 | -2.24978 | 0.016485 | -0.54324 |
| 32 | 6 | 0 | 2.735213 | 2.734594 | -1.74652 |
| 33 | 6 | 0 | 2.026234 | 2.949519 | 0.446218 |
| 34 | 6 | 0 | 2.891253 | 4.106733 | -1.86665 |
| 35 | 6 | 0 | 2.166176 | 4.32868 | 0.394409 |
| 36 | 6 | 0 | 2.615173 | 4.949573 | -0.7801 |
| 37 | 6 | 0 | 3.065841 | -2.68689 | -1.66163 |
| 38 | 6 | 0 | 1.875911 | -2.9432 | 0.304933 |
| 39 | 6 | 0 | 3.235109 | -4.05696 | -1.78012 |
| 40 | 6 | 0 | 2.015069 | -4.32331 | 0.249113 |
| 41 | 6 | 0 | 2.709662 | -4.92336 | -0.8093 |
| 42 | 6 | 0 | -3.01626 | -2.70989 | -1.66236 |
| 43 | 6 | 0 | -1.81626 | -2.93644 | 0.290507 |
| 44 | 6 | 0 | -3.19543 | -4.08244 | -1.75595 |
| 45 | 6 | 0 | -1.96901 | -4.31722 | 0.267572 |
| 46 | 6 | 0 | -2.67446 | -4.93466 | -0.77264 |
| 47 | 6 | 0 | -3.1919 | 2.73655 | -1.53417 |
| 48 | 6 | 0 | -1.78193 | 2.976706 | 0.276287 |
| 49 | 6 | 0 | -3.38842 | 4.108529 | -1.60932 |
| 50 | 6 | 0 | -1.94695 | 4.355942 | 0.266653 |
| 51 | 6 | 0 | -2.76817 | 4.966109 | -0.69053 |
| 52 | 7 | 0 | -2.3893 | 2.154254 | -0.60673 |
| 53 | 7 | 0 | -2.32557 | -2.1198 | -0.65606 |
| 54 | 7 | 0 | 2.304786 | 2.149198 | -0.60455 |
| 55 | 7 | 0 | 2.388491 | -2.11965 | -0.63321 |
| 56 | 1 | 0 | 6.338176 | -0.04901 | 4.301785 |
| 57 | 1 | 0 | 8.153527 | -0.01666 | 2.620341 |
| 58 | 1 | 0 | 7.694847 | 0.019905 | 0.187612 |
| 59 | 1 | 0 | 0.04475 | -0.04054 | 4.161729 |
| 60 | 1 | 0 | -6.17737 | -0.13301 | 4.398907 |
| 61 | 1 | 0 | -8.03866 | -0.17282 | 2.765764 |
| 62 | 1 | 0 | -7.63972 | -0.14841 | 0.319219 |
| 63 | 1 | 0 | 1.684223 | 2.45927 | 1.348823 |
| 64 | 1 | 0 | 2.983391 | 2.067199 | -2.56047 |
| 65 | 1 | 0 | 3.247739 | 4.513176 | -2.80771 |
| 66 | 1 | 0 | 1.935233 | 4.916695 | 1.276902 |
| 67 | 1 | 0 | 3.516922 | -2.00238 | -2.37014 |
| 68 | 1 | 0 | 1.340843 | -2.47014 | 1.117653 |
| 69 | 1 | 0 | 3.795387 | -4.44397 | -2.62538 |
| 70 | 1 | 0 | 1.584144 | -4.92865 | 1.039888 |
| 71 | 1 | 0 | -3.76649 | -4.47984 | -2.5893 |
| 72 | 1 | 0 | -1.54608 | -4.90884 | 1.073736 |
| 73 | 1 | 0 | -1.27831 | -2.4529 | 1.095754 |
| 74 | 1 | 0 | -3.483 | -2.03978 | -2.37435 |
| 75 | 1 | 0 | -3.72122 | 2.054549 | -2.19075 |
| 76 | 1 | 0 | -1.1477 | 2.499223 | 1.012082 |
| 77 | 1 | 0 | -4.04814 | 4.501023 | -2.37703 |
| 78 | 1 | 0 | -1.43575 | 4.953546 | 1.014972 |
| 79 | 8 | 0 | 1.309006 | 0.075734 | -2.50691 |
| 80 | 6 | 0 | 2.821444 | 6.435576 | -0.86715 |
| 81 | 1 | 0 | 2.307029 | 6.96516 | -0.06235 |
| 82 | 1 | 0 | 3.88969 | 6.671665 | -0.79145 |

| | | | | | |
|---|---|---|---|---|---|
| 83 | 1 | 0 | 2.474202 | 6.829275 | -1.82664 |
| 84 | 6 | 0 | 2.907778 | -6.41038 | -0.89632 |
| 85 | 1 | 0 | 3.957867 | -6.66468 | -0.71112 |
| 86 | 1 | 0 | 2.298831 | -6.94517 | -0.16463 |
| 87 | 1 | 0 | 2.661365 | -6.78207 | -1.89568 |
| 88 | 6 | 0 | -2.89344 | -6.42194 | -0.82222 |
| 89 | 1 | 0 | -3.93442 | -6.66181 | -0.57633 |
| 90 | 1 | 0 | -2.70265 | -6.81741 | -1.82441 |
| 91 | 1 | 0 | -2.25383 | -6.95016 | -0.11147 |
| 92 | 6 | 0 | -3.0014 | 6.451628 | -0.71673 |
| 93 | 1 | 0 | -3.9883 | 6.687833 | -0.30153 |
| 94 | 1 | 0 | -2.25618 | 6.989781 | -0.1265 |
| 95 | 1 | 0 | -2.98373 | 6.838764 | -1.7396 |
| 96 | 1 | 0 | 1.823717 | -0.3409 | -3.21219 |
| 97 | 8 | 0 | -1.44345 | 0.146227 | -2.68713 |
| 98 | 1 | 0 | -2.16533 | -0.27745 | -3.18429 |
| 99 | 1 | 0 | -1.51081 | 1.089995 | -2.90665 |
| 100 | 1 | 0 | 3.325723 | -0.05936 | 4.337702 |
| 101 | 1 | 0 | 0.364741 | -0.15513 | -2.61949 |
| 102 | 1 | 0 | -3.18687 | -0.05276 | 4.36098 |

**Table S16.** Cartesian coordinates for Ru$^{III}$Ru$^{III}$-6pic.

| Center number | Atomic number | Atomic type | Coordinates (Ångström) | | |
|---|---|---|---|---|---|
| | | | X | Y | Z |
| 1 | 6 | 0 | -4.52699 | 0.58052 | -2.05589 |
| 2 | 6 | 0 | -4.51401 | 0.78162 | -3.45916 |
| 3 | 6 | 0 | -5.72908 | 0.999479 | -4.10564 |
| 4 | 6 | 0 | -6.89493 | 0.994788 | -3.319 |
| 5 | 6 | 0 | -6.87521 | 0.770792 | -1.93157 |
| 6 | 6 | 0 | -5.66876 | 0.546401 | -1.25398 |
| 7 | 6 | 0 | -2.48489 | 0.463323 | -2.85178 |
| 8 | 6 | 0 | -5.61351 | 0.256561 | 0.224001 |
| 9 | 8 | 0 | -6.63467 | 0.315255 | 0.897759 |
| 10 | 8 | 0 | -4.45097 | -0.0877 | 0.780335 |
| 11 | 7 | 0 | -3.23124 | 0.398707 | -1.68593 |
| 12 | 7 | 0 | -3.19362 | 0.696933 | -3.93635 |
| 13 | 6 | 0 | -3E-06 | -7.6E-05 | -3.47564 |
| 14 | 6 | 0 | 1.072693 | -0.23208 | -2.6187 |
| 15 | 7 | 0 | -0.66934 | 0.128405 | -1.30072 |
| 16 | 7 | 0 | 0.669352 | -0.12845 | -1.30072 |
| 17 | 6 | 0 | -1.07269 | 0.231976 | -2.6187 |
| 18 | 6 | 0 | 4.526985 | -0.58065 | -2.05589 |
| 19 | 6 | 0 | 4.513994 | -0.78184 | -3.45914 |
| 20 | 6 | 0 | 5.729062 | -0.99978 | -4.10561 |
| 21 | 6 | 0 | 6.89491 | -0.99509 | -3.31897 |
| 22 | 6 | 0 | 6.8752 | -0.77102 | -1.93155 |
| 23 | 6 | 0 | 5.668762 | -0.54653 | -1.25398 |
| 24 | 6 | 0 | 2.484886 | -0.46345 | -2.85178 |
| 25 | 7 | 0 | 3.231245 | -0.39878 | -1.68594 |
| 26 | 7 | 0 | 3.193608 | -0.69715 | -3.93633 |

| | | | | | |
|---|---|---|---|---|---|
| 27 | 6 | 0 | 5.613501 | -0.25663 | 0.22399 |
| 28 | 8 | 0 | 6.634641 | -0.31544 | 0.897778 |
| 29 | 8 | 0 | 4.450993 | 0.087797 | 0.780276 |
| 30 | 44 | 0 | 2.509109 | 0.06745 | 0.027306 |
| 31 | 44 | 0 | -2.50911 | -0.06742 | 0.027338 |
| 32 | 6 | 0 | 3.246449 | -2.45453 | 1.57979 |
| 33 | 6 | 0 | 1.75768 | -2.91175 | -0.12838 |
| 34 | 6 | 0 | 3.340178 | -3.80199 | 1.904594 |
| 35 | 6 | 0 | 1.811406 | -4.27205 | 0.138371 |
| 36 | 6 | 0 | 2.619808 | -4.75678 | 1.176029 |
| 37 | 6 | 0 | 3.694105 | 2.856718 | 0.147227 |
| 38 | 6 | 0 | 2.005283 | 2.775887 | -1.42867 |
| 39 | 6 | 0 | 3.955991 | 4.187176 | -0.15143 |
| 40 | 6 | 0 | 2.22095 | 4.101434 | -1.78072 |
| 41 | 6 | 0 | 3.220147 | 4.848135 | -1.14237 |
| 42 | 6 | 0 | -3.24636 | 2.454621 | 1.579748 |
| 43 | 6 | 0 | -1.7577 | 2.911766 | -0.12854 |
| 44 | 6 | 0 | -3.34007 | 3.802093 | 1.904494 |
| 45 | 6 | 0 | -1.81142 | 4.272081 | 0.138147 |
| 46 | 6 | 0 | -2.61976 | 4.756856 | 1.175833 |
| 47 | 6 | 0 | -3.69412 | -2.85669 | 0.147415 |
| 48 | 6 | 0 | -2.00535 | -2.77592 | -1.42853 |
| 49 | 6 | 0 | -3.95604 | -4.18715 | -0.15121 |
| 50 | 6 | 0 | -2.22106 | -4.10147 | -1.78055 |
| 51 | 6 | 0 | -3.22025 | -4.84814 | -1.14217 |
| 52 | 6 | 0 | 2.77051 | 0.857299 | 3.020924 |
| 53 | 6 | 0 | 0.693626 | 1.426931 | 2.191961 |
| 54 | 6 | 0 | 2.48933 | 1.461639 | 4.239281 |
| 55 | 6 | 0 | 0.350479 | 2.065374 | 3.377927 |
| 56 | 6 | 0 | 1.255412 | 2.09336 | 4.446208 |
| 57 | 6 | 0 | -2.7705 | -0.85718 | 3.020991 |
| 58 | 6 | 0 | -0.69359 | -1.42675 | 2.192052 |
| 59 | 6 | 0 | -2.4893 | -1.46147 | 4.239368 |
| 60 | 6 | 0 | -0.35042 | -2.06514 | 3.37804 |
| 61 | 6 | 0 | -1.25536 | -2.09313 | 4.446318 |
| 62 | 7 | 0 | -2.72648 | -2.14755 | -0.47571 |
| 63 | 7 | 0 | -2.45751 | 1.99988 | 0.581458 |
| 64 | 7 | 0 | -1.88343 | -0.82425 | 2.001292 |
| 65 | 7 | 0 | 1.883447 | 0.824371 | 2.001222 |
| 66 | 7 | 0 | 2.457539 | -1.99983 | 0.581533 |
| 67 | 7 | 0 | 2.72645 | 2.147554 | -0.47586 |
| 68 | 1 | 0 | 5.776805 | -1.16033 | -5.1778 |
| 69 | 1 | 0 | 7.851501 | -1.16176 | -3.80484 |
| 70 | 1 | 0 | 7.796485 | -0.7588 | -1.35923 |
| 71 | 1 | 0 | -9E-06 | -9.9E-05 | -4.55462 |
| 72 | 1 | 0 | -5.77683 | 1.159949 | -5.17783 |
| 73 | 1 | 0 | -7.85153 | 1.161395 | -3.80488 |
| 74 | 1 | 0 | -7.79649 | 0.758566 | -1.35924 |
| 75 | 1 | 0 | 1.144449 | -2.51499 | -0.92683 |
| 76 | 1 | 0 | 3.83892 | -1.70802 | 2.091751 |
| 77 | 1 | 0 | 3.998308 | -4.10178 | 2.714066 |
| 78 | 1 | 0 | 1.224818 | -4.95147 | -0.47182 |
| 79 | 1 | 0 | 4.278784 | 2.312043 | 0.87846 |
| 80 | 1 | 0 | 1.247033 | 2.186107 | -1.9254 |
| 81 | 1 | 0 | 4.750744 | 4.697633 | 0.38343 |

| | | | | | |
|---|---|---|---|---|---|
| 82 | 1 | 0 | 1.614874 | 4.5422 | -2.56627 |
| 83 | 1 | 0 | -3.99815 | 4.101923 | 2.713997 |
| 84 | 1 | 0 | -1.22488 | 4.951481 | -0.47212 |
| 85 | 1 | 0 | -1.14452 | 2.514975 | -0.92701 |
| 86 | 1 | 0 | -3.83879 | 1.708135 | 2.091782 |
| 87 | 1 | 0 | -4.27877 | -2.312 | 0.878664 |
| 88 | 1 | 0 | -1.2471 | -2.18617 | -1.9253 |
| 89 | 1 | 0 | -4.75079 | -4.69758 | 0.38369 |
| 90 | 1 | 0 | -1.61501 | -4.54226 | -2.56611 |
| 91 | 1 | 0 | 3.740489 | 0.419818 | 2.809312 |
| 92 | 1 | 0 | 0.001928 | 1.382992 | 1.360897 |
| 93 | 1 | 0 | 3.244881 | 1.451419 | 5.019065 |
| 94 | 1 | 0 | -0.62396 | 2.536041 | 3.461909 |
| 95 | 1 | 0 | -3.7405 | -0.41974 | 2.809362 |
| 96 | 1 | 0 | -0.0019 | -1.38282 | 1.360985 |
| 97 | 1 | 0 | -3.24486 | -1.45126 | 5.019146 |
| 98 | 1 | 0 | 0.624032 | -2.53577 | 3.462041 |
| 99 | 6 | 0 | -2.73901 | 6.22842 | 1.464805 |
| 100 | 1 | 0 | -1.81797 | 6.762707 | 1.217201 |
| 101 | 1 | 0 | -3.54384 | 6.666147 | 0.861923 |
| 102 | 1 | 0 | -2.98055 | 6.413425 | 2.514567 |
| 103 | 6 | 0 | 3.514027 | 6.272324 | -1.52849 |
| 104 | 1 | 0 | 4.288959 | 6.299719 | -2.30407 |
| 105 | 1 | 0 | 2.629559 | 6.771 | -1.93316 |
| 106 | 1 | 0 | 3.884632 | 6.849936 | -0.67758 |
| 107 | 6 | 0 | 0.936909 | 2.791114 | 5.741501 |
| 108 | 1 | 0 | 1.299752 | 2.219345 | 6.600507 |
| 109 | 1 | 0 | 1.426564 | 3.771629 | 5.777596 |
| 110 | 1 | 0 | -0.13743 | 2.952082 | 5.859467 |
| 111 | 6 | 0 | -0.93683 | -2.79081 | 5.741644 |
| 112 | 1 | 0 | -1.29935 | -2.21882 | 6.600638 |
| 113 | 1 | 0 | -1.42677 | -3.77118 | 5.777963 |
| 114 | 1 | 0 | 0.137487 | -2.95208 | 5.859446 |
| 115 | 6 | 0 | 2.739055 | -6.22833 | 1.465054 |
| 116 | 1 | 0 | 3.543371 | -6.66624 | 0.861613 |
| 117 | 1 | 0 | 2.981335 | -6.41328 | 2.514655 |
| 118 | 1 | 0 | 1.81776 | -6.76251 | 1.218168 |
| 119 | 6 | 0 | -3.51418 | -6.27233 | -1.52824 |
| 120 | 1 | 0 | -4.2892 | -6.29972 | -2.30374 |
| 121 | 1 | 0 | -2.62975 | -6.77101 | -1.93302 |
| 122 | 1 | 0 | -3.88469 | -6.84994 | -0.6773 |

**Table S17.** Cartesian coordinates for $Ru^{III}Ru^{III}$-6pic-$H^{+}$.

| Center number | Atomic number | Atomic type | Coordinates (Ångström) | | |
|---|---|---|---|---|---|
| | | | X | Y | Z |
| 1 | 6 | 0 | -4.40124 | 0.604288 | -2.18126 |
| 2 | 6 | 0 | -4.32163 | 0.806515 | -3.58229 |
| 3 | 6 | 0 | -5.50404 | 1.025461 | -4.29267 |
| 4 | 6 | 0 | -6.70518 | 1.018632 | -3.56609 |
| 5 | 6 | 0 | -6.75227 | 0.791231 | -2.17815 |

| | | | | | |
|---|---|---|---|---|---|
| 6 | 6 | 0 | -5.5814 | 0.566518 | -1.44034 |
| 7 | 6 | 0 | -2.32927 | 0.490762 | -2.86849 |
| 8 | 6 | 0 | -5.60239 | 0.274222 | 0.035902 |
| 9 | 8 | 0 | -6.6513 | 0.323644 | 0.659421 |
| 10 | 8 | 0 | -4.46675 | -0.06675 | 0.65821 |
| 11 | 7 | 0 | -3.12339 | 0.424097 | -1.74298 |
| 12 | 7 | 0 | -2.98461 | 0.721289 | -3.99149 |
| 13 | 6 | 0 | 0.150172 | 0.053252 | -3.47376 |
| 14 | 6 | 0 | 1.22247 | -0.17328 | -2.62463 |
| 15 | 7 | 0 | -0.52536 | 0.162325 | -1.2942 |
| 16 | 7 | 0 | 0.814057 | -0.07464 | -1.29106 |
| 17 | 6 | 0 | -0.92551 | 0.266412 | -2.60604 |
| 18 | 6 | 0 | 4.668231 | -0.52986 | -2.01339 |
| 19 | 6 | 0 | 4.770591 | -0.69275 | -3.40758 |
| 20 | 6 | 0 | 6.021903 | -0.88374 | -3.99556 |
| 21 | 6 | 0 | 7.121312 | -0.89164 | -3.13003 |
| 22 | 6 | 0 | 7.002906 | -0.70883 | -1.73644 |
| 23 | 6 | 0 | 5.756645 | -0.51118 | -1.13886 |
| 24 | 6 | 0 | 2.632459 | -0.39567 | -2.81293 |
| 25 | 7 | 0 | 3.351999 | -0.37273 | -1.69991 |
| 26 | 7 | 0 | 3.453579 | -0.5993 | -3.88689 |
| 27 | 6 | 0 | 5.592745 | -0.28212 | 0.339284 |
| 28 | 8 | 0 | 6.529632 | -0.44162 | 1.098768 |
| 29 | 8 | 0 | 4.403997 | 0.13985 | 0.82634 |
| 30 | 44 | 0 | 2.540283 | 0.069933 | 0.061629 |
| 31 | 44 | 0 | -2.51189 | -0.05899 | 0.014546 |
| 32 | 6 | 0 | 3.267952 | -2.516 | 1.527297 |
| 33 | 6 | 0 | 1.750834 | -2.90522 | -0.17383 |
| 34 | 6 | 0 | 3.329839 | -3.86891 | 1.827857 |
| 35 | 6 | 0 | 1.764926 | -4.26967 | 0.077477 |
| 36 | 6 | 0 | 2.572204 | -4.7948 | 1.096417 |
| 37 | 6 | 0 | 3.613088 | 2.898217 | 0.384532 |
| 38 | 6 | 0 | 2.083402 | 2.823514 | -1.34674 |
| 39 | 6 | 0 | 3.846736 | 4.247536 | 0.165404 |
| 40 | 6 | 0 | 2.271954 | 4.171994 | -1.61825 |
| 41 | 6 | 0 | 3.177548 | 4.927858 | -0.86178 |
| 42 | 6 | 0 | -3.31499 | 2.447172 | 1.561759 |
| 43 | 6 | 0 | -1.7849 | 2.937669 | -0.09876 |
| 44 | 6 | 0 | -3.44762 | 3.791302 | 1.884117 |
| 45 | 6 | 0 | -1.87729 | 4.296115 | 0.165253 |
| 46 | 6 | 0 | -2.72867 | 4.764162 | 1.177066 |
| 47 | 6 | 0 | -3.73886 | -2.83357 | 0.110128 |
| 48 | 6 | 0 | -2.05864 | -2.766 | -1.47341 |
| 49 | 6 | 0 | -4.03565 | -4.15094 | -0.21186 |
| 50 | 6 | 0 | -2.30864 | -4.07803 | -1.84984 |
| 51 | 6 | 0 | -3.32432 | -4.81257 | -1.22061 |
| 52 | 6 | 0 | 2.648931 | 0.666902 | 3.091584 |
| 53 | 6 | 0 | 0.654248 | 1.35157 | 2.155928 |
| 54 | 6 | 0 | 2.302244 | 1.213772 | 4.319402 |
| 55 | 6 | 0 | 0.249978 | 1.935122 | 3.349996 |
| 56 | 6 | 0 | 1.077486 | 1.877305 | 4.478871 |
| 57 | 6 | 0 | -2.93205 | -0.8471 | 2.9932 |
| 58 | 6 | 0 | -0.83984 | -1.47789 | 2.253133 |
| 59 | 6 | 0 | -2.71889 | -1.45389 | 4.223705 |
| 60 | 6 | 0 | -0.56352 | -2.11885 | 3.45405 |

| | | | | | |
|---|---|---|---|---|---|
| 61 | 6 | 0 | -1.51205 | -2.11757 | 4.485871 |
| 62 | 7 | 0 | -2.75707 | -2.13517 | -0.50515 |
| 63 | 7 | 0 | -2.48625 | 2.007808 | 0.587224 |
| 64 | 7 | 0 | -2.0029 | -0.84064 | 2.009989 |
| 65 | 7 | 0 | 1.837418 | 0.721114 | 2.010878 |
| 66 | 7 | 0 | 2.485914 | -2.02179 | 0.538676 |
| 67 | 7 | 0 | 2.741698 | 2.176568 | -0.35981 |
| 68 | 1 | 0 | 6.151059 | -1.01544 | -5.06446 |
| 69 | 1 | 0 | 8.109984 | -1.03741 | -3.55233 |
| 70 | 1 | 0 | 7.886503 | -0.7121 | -1.10747 |
| 71 | 1 | 0 | 0.120107 | 0.063326 | -4.55309 |
| 72 | 1 | 0 | -5.49716 | 1.188127 | -5.36531 |
| 73 | 1 | 0 | -7.63727 | 1.185427 | -4.09639 |
| 74 | 1 | 0 | -7.70168 | 0.777323 | -1.65363 |
| 75 | 1 | 0 | 1.131528 | -2.48597 | -0.95531 |
| 76 | 1 | 0 | 3.878693 | -1.79949 | 2.059896 |
| 77 | 1 | 0 | 3.986956 | -4.19677 | 2.627129 |
| 78 | 1 | 0 | 1.14453 | -4.92149 | -0.52905 |
| 79 | 1 | 0 | 4.145355 | 2.353682 | 1.153541 |
| 80 | 1 | 0 | 1.387083 | 2.238648 | -1.93182 |
| 81 | 1 | 0 | 4.564659 | 4.762152 | 0.796209 |
| 82 | 1 | 0 | 1.714271 | 4.62639 | -2.43121 |
| 83 | 1 | 0 | -4.13695 | 4.074871 | 2.673125 |
| 84 | 1 | 0 | -1.29153 | 4.989795 | -0.42943 |
| 85 | 1 | 0 | -1.13984 | 2.560379 | -0.88158 |
| 86 | 1 | 0 | -3.90898 | 1.691667 | 2.057075 |
| 87 | 1 | 0 | -4.30915 | -2.2916 | 0.85361 |
| 88 | 1 | 0 | -1.28864 | -2.18668 | -1.9654 |
| 89 | 1 | 0 | -4.84047 | -4.65023 | 0.318254 |
| 90 | 1 | 0 | -1.72076 | -4.5197 | -2.64863 |
| 91 | 1 | 0 | 3.617503 | 0.208108 | 2.93657 |
| 92 | 1 | 0 | 0.017269 | 1.377608 | 1.28175 |
| 93 | 1 | 0 | 3.00146 | 1.133455 | 5.145962 |
| 94 | 1 | 0 | -0.71412 | 2.43152 | 3.392212 |
| 95 | 1 | 0 | -3.88184 | -0.38764 | 2.74288 |
| 96 | 1 | 0 | -0.11491 | -1.46822 | 1.44877 |
| 97 | 1 | 0 | -3.5073 | -1.42187 | 4.969471 |
| 98 | 1 | 0 | 0.391258 | -2.61949 | 3.580418 |
| 99 | 6 | 0 | -2.89412 | 6.231067 | 1.460967 |
| 100 | 1 | 0 | -2.00751 | 6.801202 | 1.172938 |
| 101 | 1 | 0 | -3.74137 | 6.629255 | 0.889478 |
| 102 | 1 | 0 | -3.10267 | 6.414292 | 2.518122 |
| 103 | 6 | 0 | 3.441255 | 6.379413 | -1.14886 |
| 104 | 1 | 0 | 4.376681 | 6.48777 | -1.71092 |
| 105 | 1 | 0 | 2.643269 | 6.826323 | -1.7459 |
| 106 | 1 | 0 | 3.553792 | 6.953753 | -0.22509 |
| 107 | 6 | 0 | 0.692303 | 2.517938 | 5.784077 |
| 108 | 1 | 0 | 1.0368 | 1.925785 | 6.636142 |
| 109 | 1 | 0 | 1.155426 | 3.50818 | 5.871859 |
| 110 | 1 | 0 | -0.3891 | 2.651188 | 5.864945 |
| 111 | 6 | 0 | -1.26825 | -2.81487 | 5.796278 |
| 112 | 1 | 0 | -1.58136 | -2.19254 | 6.639803 |
| 113 | 1 | 0 | -1.85226 | -3.74119 | 5.849263 |
| 114 | 1 | 0 | -0.2156 | -3.07543 | 5.927766 |
| 115 | 6 | 0 | 2.649452 | -6.27043 | 1.370312 |

| | | | | | |
|---|---|---|---|---|---|
| 116 | 1 | 0 | 3.511959 | -6.70346 | 0.849129 |
| 117 | 1 | 0 | 2.782111 | -6.47326 | 2.436212 |
| 118 | 1 | 0 | 1.756538 | -6.79369 | 1.020534 |
| 119 | 6 | 0 | -3.65927 | -6.21922 | -1.6324 |
| 120 | 1 | 0 | -4.46039 | -6.20936 | -2.38145 |
| 121 | 1 | 0 | -2.80028 | -6.72587 | -2.0792 |
| 122 | 1 | 0 | -4.01582 | -6.81057 | -0.78515 |
| 123 | 1 | 0 | 3.162409 | -0.67424 | -4.85091 |

**Table S18.** Cartesian coordinates for $Ru^{III}Ru^{III}$-6pic-2$H^+$.

| Center number | Atomic number | Atomic type | Coordinates (Ångström) | | |
|---|---|---|---|---|---|
| | | | X | Y | Z |
| 1 | 6 | 0 | -4.56066 | 0.426196 | -2.11407 |
| 2 | 6 | 0 | -4.61957 | 0.635131 | -3.5037 |
| 3 | 6 | 0 | -5.8524 | 0.83314 | -4.13155 |
| 4 | 6 | 0 | -6.97985 | 0.805673 | -3.30651 |
| 5 | 6 | 0 | -6.90779 | 0.583566 | -1.91331 |
| 6 | 6 | 0 | -5.68174 | 0.375829 | -1.28131 |
| 7 | 6 | 0 | -2.49968 | 0.35535 | -2.84642 |
| 8 | 6 | 0 | -5.56003 | 0.112059 | 0.188137 |
| 9 | 8 | 0 | -6.48517 | 0.285839 | 0.951948 |
| 10 | 8 | 0 | -4.39029 | -0.39144 | 0.674887 |
| 11 | 7 | 0 | -3.25199 | 0.276913 | -1.75788 |
| 12 | 7 | 0 | -3.28739 | 0.579775 | -3.93951 |
| 13 | 6 | 0 | -1.3E-05 | -0.0001 | -3.49718 |
| 14 | 6 | 0 | 1.08124 | -0.18047 | -2.63655 |
| 15 | 7 | 0 | -0.67255 | 0.093775 | -1.31731 |
| 16 | 7 | 0 | 0.672568 | -0.09388 | -1.31732 |
| 17 | 6 | 0 | -1.08125 | 0.180327 | -2.63653 |
| 18 | 6 | 0 | 4.560659 | -0.42627 | -2.11412 |
| 19 | 6 | 0 | 4.619556 | -0.63526 | -3.50375 |
| 20 | 6 | 0 | 5.852384 | -0.83326 | -4.13161 |
| 21 | 6 | 0 | 6.979845 | -0.80574 | -3.30658 |
| 22 | 6 | 0 | 6.907798 | -0.58358 | -1.9134 |
| 23 | 6 | 0 | 5.681753 | -0.37585 | -1.28138 |
| 24 | 6 | 0 | 2.499672 | -0.35549 | -2.84645 |
| 25 | 7 | 0 | 3.251997 | -0.277 | -1.75793 |
| 26 | 7 | 0 | 3.287375 | -0.57994 | -3.93954 |
| 27 | 6 | 0 | 5.560065 | -0.11201 | 0.188057 |
| 28 | 8 | 0 | 6.485244 | -0.2857 | 0.951844 |
| 29 | 8 | 0 | 4.390309 | 0.391428 | 0.674831 |
| 30 | 44 | 0 | 2.523765 | 0.184522 | 0.049502 |
| 31 | 44 | 0 | -2.52375 | -0.18455 | 0.049565 |
| 32 | 6 | 0 | 3.442122 | -2.32761 | 1.541977 |
| 33 | 6 | 0 | 1.956596 | -2.84609 | -0.15254 |
| 34 | 6 | 0 | 3.625733 | -3.66953 | 1.839446 |
| 35 | 6 | 0 | 2.090848 | -4.20378 | 0.097315 |
| 36 | 6 | 0 | 2.949463 | -4.65914 | 1.10971 |
| 37 | 6 | 0 | 3.442618 | 3.07608 | 0.314716 |
| 38 | 6 | 0 | 1.937263 | 2.888224 | -1.42729 |

| | | | | | |
|---|---|---|---|---|---|
| 39 | 6 | 0 | 3.627384 | 4.425699 | 0.055079 |
| 40 | 6 | 0 | 2.07748 | 4.232736 | -1.74156 |
| 41 | 6 | 0 | 2.94637 | 5.048093 | -1.0013 |
| 42 | 6 | 0 | -3.44216 | 2.327667 | 1.541851 |
| 43 | 6 | 0 | -1.95648 | 2.846034 | -0.15256 |
| 44 | 6 | 0 | -3.62576 | 3.669614 | 1.839246 |
| 45 | 6 | 0 | -2.09071 | 4.203738 | 0.097223 |
| 46 | 6 | 0 | -2.94939 | 4.659164 | 1.109526 |
| 47 | 6 | 0 | -3.44276 | -3.07605 | 0.314833 |
| 48 | 6 | 0 | -1.93722 | -2.88836 | -1.42703 |
| 49 | 6 | 0 | -3.62758 | -4.42567 | 0.055241 |
| 50 | 6 | 0 | -2.07748 | -4.23288 | -1.74125 |
| 51 | 6 | 0 | -2.94649 | -5.04815 | -1.00103 |
| 52 | 6 | 0 | 2.764598 | 0.827149 | 3.064699 |
| 53 | 6 | 0 | 0.712715 | 1.451223 | 2.212961 |
| 54 | 6 | 0 | 2.459066 | 1.37696 | 4.301294 |
| 55 | 6 | 0 | 0.344868 | 2.030964 | 3.419825 |
| 56 | 6 | 0 | 1.2234 | 2.007067 | 4.512435 |
| 57 | 6 | 0 | -2.76457 | -0.82694 | 3.064809 |
| 58 | 6 | 0 | -0.71273 | -1.45119 | 2.213082 |
| 59 | 6 | 0 | -2.45904 | -1.37667 | 4.301443 |
| 60 | 6 | 0 | -0.34489 | -2.03085 | 3.419987 |
| 61 | 6 | 0 | -1.2234 | -2.00682 | 4.512612 |
| 62 | 7 | 0 | -2.60607 | -2.29688 | -0.41239 |
| 63 | 7 | 0 | -2.61528 | 1.900323 | 0.556756 |
| 64 | 7 | 0 | -1.90398 | -0.84732 | 2.019902 |
| 65 | 7 | 0 | 1.90399 | 0.847396 | 2.019806 |
| 66 | 7 | 0 | 2.615307 | -1.90033 | 0.556793 |
| 67 | 7 | 0 | 2.60605 | 2.296831 | -0.41256 |
| 68 | 1 | 0 | 5.945168 | -0.9977 | -5.1997 |
| 69 | 1 | 0 | 7.955015 | -0.95583 | -3.75761 |
| 70 | 1 | 0 | 7.814381 | -0.56387 | -1.31798 |
| 71 | 1 | 0 | -2.5E-05 | -0.00012 | -4.57746 |
| 72 | 1 | 0 | -5.94519 | 0.997542 | -5.19965 |
| 73 | 1 | 0 | -7.95502 | 0.955773 | -3.75753 |
| 74 | 1 | 0 | -7.81437 | 0.563902 | -1.31789 |
| 75 | 1 | 0 | 1.301444 | -2.4854 | -0.93467 |
| 76 | 1 | 0 | 3.991859 | -1.5655 | 2.076486 |
| 77 | 1 | 0 | 4.315589 | -3.93887 | 2.632964 |
| 78 | 1 | 0 | 1.530022 | -4.90814 | -0.50854 |
| 79 | 1 | 0 | 3.994506 | 2.58019 | 1.101757 |
| 80 | 1 | 0 | 1.27473 | 2.257102 | -2.00407 |
| 81 | 1 | 0 | 4.320326 | 4.986167 | 0.674658 |
| 82 | 1 | 0 | 1.517318 | 4.640143 | -2.57741 |
| 83 | 1 | 0 | -4.31567 | 3.939004 | 2.632695 |
| 84 | 1 | 0 | -1.52981 | 4.90805 | -0.50861 |
| 85 | 1 | 0 | -1.30127 | 2.485281 | -0.93462 |
| 86 | 1 | 0 | -3.99197 | 1.565601 | 2.076346 |
| 87 | 1 | 0 | -3.99471 | -2.58008 | 1.101784 |
| 88 | 1 | 0 | -1.2746 | -2.25731 | -2.00378 |
| 89 | 1 | 0 | -4.32062 | -4.98606 | 0.674773 |
| 90 | 1 | 0 | -1.51727 | -4.64036 | -2.57703 |
| 91 | 1 | 0 | 3.74012 | 0.398819 | 2.872673 |
| 92 | 1 | 0 | 0.041945 | 1.468629 | 1.363246 |
| 93 | 1 | 0 | 3.199196 | 1.328139 | 5.093982 |

| | | | | | |
|---|---|---|---|---|---|
| 94 | 1 | 0 | -0.62723 | 2.5063 | 3.502285 |
| 95 | 1 | 0 | -3.74007 | -0.39858 | 2.872768 |
| 96 | 1 | 0 | -0.04199 | -1.46871 | 1.363353 |
| 97 | 1 | 0 | -3.19915 | -1.32775 | 5.094142 |
| 98 | 1 | 0 | 0.627188 | -2.50623 | 3.502467 |
| 99 | 6 | 0 | -3.163 | 6.120933 | 1.377783 |
| 100 | 1 | 0 | -2.33518 | 6.727932 | 1.004864 |
| 101 | 1 | 0 | -4.07659 | 6.462221 | 0.87536 |
| 102 | 1 | 0 | -3.29321 | 6.317048 | 2.4453 |
| 103 | 6 | 0 | 3.162378 | 6.496465 | -1.33482 |
| 104 | 1 | 0 | 4.110475 | 6.619655 | -1.87235 |
| 105 | 1 | 0 | 2.367631 | 6.891194 | -1.97136 |
| 106 | 1 | 0 | 3.227461 | 7.107736 | -0.43037 |
| 107 | 6 | 0 | 0.882624 | 2.64781 | 5.828396 |
| 108 | 1 | 0 | 1.203267 | 2.025098 | 6.668325 |
| 109 | 1 | 0 | 1.403205 | 3.607934 | 5.927312 |
| 110 | 1 | 0 | -0.1885 | 2.839188 | 5.922554 |
| 111 | 6 | 0 | -0.88263 | -2.64747 | 5.828622 |
| 112 | 1 | 0 | -1.20321 | -2.02466 | 6.668503 |
| 113 | 1 | 0 | -1.40327 | -3.60755 | 5.927639 |
| 114 | 1 | 0 | 0.188483 | -2.8389 | 5.922768 |
| 115 | 6 | 0 | 3.163089 | -6.12089 | 1.378049 |
| 116 | 1 | 0 | 4.076739 | -6.46217 | 0.87572 |
| 117 | 1 | 0 | 3.29322 | -6.31695 | 2.445586 |
| 118 | 1 | 0 | 2.335325 | -6.72793 | 1.005083 |
| 119 | 6 | 0 | -3.16256 | -6.49653 | -1.33451 |
| 120 | 1 | 0 | -4.1106 | -6.61967 | -1.87214 |
| 121 | 1 | 0 | -2.36777 | -6.89134 | -1.97095 |
| 122 | 1 | 0 | -3.22778 | -7.10775 | -0.43005 |
| 123 | 1 | 0 | 2.970317 | -0.70712 | -4.89043 |
| 124 | 1 | 0 | -2.97034 | 0.706925 | -4.8904 |

**Table S19.** Cartesian coordinates for $Ru^{III}Ru^{III}$-6pic-OH-A.

| Center number | Atomic number | Atomic type | Coordinates (Ångström) | | |
|---|---|---|---|---|---|
| | | | X | Y | Z |
| 1 | 6 | 0 | 3.602311 | -1.61034 | -2.51862 |
| 2 | 6 | 0 | 2.974207 | -2.06905 | -3.71316 |
| 3 | 6 | 0 | 3.685546 | -2.81088 | -4.66545 |
| 4 | 6 | 0 | 5.022835 | -3.10083 | -4.41072 |
| 5 | 6 | 0 | 5.644239 | -2.65307 | -3.23362 |
| 6 | 6 | 0 | 4.965892 | -1.89141 | -2.27463 |
| 7 | 6 | 0 | 1.498553 | -1.04926 | -2.55468 |
| 8 | 6 | 0 | 5.727958 | -1.40496 | -1.08972 |
| 9 | 8 | 0 | 6.818915 | -1.87346 | -0.7747 |
| 10 | 8 | 0 | 5.20323 | -0.38453 | -0.41938 |
| 11 | 7 | 0 | 2.641863 | -0.94896 | -1.77598 |
| 12 | 7 | 0 | 1.657183 | -1.69422 | -3.71102 |
| 13 | 6 | 0 | -0.84445 | -0.22855 | -3.14778 |
| 14 | 6 | 0 | -1.97231 | 0.02393 | -2.37854 |
| 15 | 7 | 0 | -0.35816 | -0.57004 | -0.95353 |

| | | | | | |
|---|---|---|---|---|---|
| 16 | 7 | 0 | -1.64428 | -0.18386 | -1.04089 |
| 17 | 6 | 0 | 0.147822 | -0.59044 | -2.21741 |
| 18 | 6 | 0 | -5.30279 | 0.835193 | -1.68738 |
| 19 | 6 | 0 | -5.33269 | 1.052043 | -3.09507 |
| 20 | 6 | 0 | -6.53628 | 1.484797 | -3.65692 |
| 21 | 6 | 0 | -7.62959 | 1.68 | -2.79771 |
| 22 | 6 | 0 | -7.5601 | 1.459358 | -1.40844 |
| 23 | 6 | 0 | -6.37458 | 1.022127 | -0.80585 |
| 24 | 6 | 0 | -3.35156 | 0.415558 | -2.59691 |
| 25 | 7 | 0 | -4.04912 | 0.431879 | -1.40065 |
| 26 | 7 | 0 | -4.076 | 0.778836 | -3.64497 |
| 27 | 6 | 0 | -6.26502 | 0.772585 | 0.679147 |
| 28 | 8 | 0 | -7.22298 | 1.015165 | 1.408492 |
| 29 | 8 | 0 | -5.13482 | 0.265766 | 1.177939 |
| 30 | 44 | 0 | -3.23647 | -0.00487 | 0.302624 |
| 31 | 44 | 0 | 3.255875 | 0.116456 | -0.0587 |
| 32 | 6 | 0 | -3.48493 | 2.694253 | 1.646578 |
| 33 | 6 | 0 | -1.94637 | 2.766427 | -0.08157 |
| 34 | 6 | 0 | -3.29111 | 4.041111 | 1.922527 |
| 35 | 6 | 0 | -1.70906 | 4.116368 | 0.14203 |
| 36 | 6 | 0 | -2.38864 | 4.793462 | 1.161368 |
| 37 | 6 | 0 | -4.86457 | -2.58108 | 0.288345 |
| 38 | 6 | 0 | -2.70365 | -2.94328 | -0.45582 |
| 39 | 6 | 0 | -5.1956 | -3.91412 | 0.084063 |
| 40 | 6 | 0 | -2.97083 | -4.28843 | -0.67752 |
| 41 | 6 | 0 | -4.24385 | -4.80892 | -0.41852 |
| 42 | 6 | 0 | 4.10998 | -2.23631 | 1.683215 |
| 43 | 6 | 0 | 1.906069 | -2.39866 | 0.995461 |
| 44 | 6 | 0 | 4.0309 | -3.45074 | 2.351158 |
| 45 | 6 | 0 | 1.760846 | -3.61821 | 1.647156 |
| 46 | 6 | 0 | 2.835488 | -4.18 | 2.344779 |
| 47 | 6 | 0 | 4.512975 | 2.023216 | -2.08628 |
| 48 | 6 | 0 | 2.591206 | 2.891293 | -1.13265 |
| 49 | 6 | 0 | 4.666777 | 3.148951 | -2.88551 |
| 50 | 6 | 0 | 2.691333 | 4.044091 | -1.90121 |
| 51 | 6 | 0 | 3.742244 | 4.197096 | -2.81305 |
| 52 | 6 | 0 | -3.27975 | -0.7845 | 3.265381 |
| 53 | 6 | 0 | -1.1022 | -0.45495 | 2.55869 |
| 54 | 6 | 0 | -2.85437 | -1.09896 | 4.549556 |
| 55 | 6 | 0 | -0.60963 | -0.75253 | 3.825647 |
| 56 | 6 | 0 | -1.48767 | -1.0945 | 4.859553 |
| 57 | 6 | 0 | 5.367913 | 1.323734 | 1.860837 |
| 58 | 6 | 0 | 3.208506 | 1.829293 | 2.515907 |
| 59 | 6 | 0 | 5.896307 | 2.046559 | 2.923596 |
| 60 | 6 | 0 | 3.673374 | 2.567562 | 3.598895 |
| 61 | 6 | 0 | 5.047724 | 2.696709 | 3.827857 |
| 62 | 7 | 0 | 3.489182 | 1.88953 | -1.21865 |
| 63 | 7 | 0 | 3.063572 | -1.70805 | 1.015 |
| 64 | 7 | 0 | 4.038463 | 1.211069 | 1.651774 |
| 65 | 7 | 0 | -2.41885 | -0.47313 | 2.271552 |
| 66 | 7 | 0 | -2.8228 | 2.055809 | 0.657471 |
| 67 | 7 | 0 | -3.63603 | -2.08874 | 0.018161 |
| 68 | 1 | 0 | -6.62447 | 1.666432 | -4.72386 |
| 69 | 1 | 0 | -8.57132 | 2.01747 | -3.22203 |
| 70 | 1 | 0 | -8.42697 | 1.62517 | -0.77748 |

| | | | | | |
|---|---|---|---|---|---|
| 71 | 1 | 0 | -0.7411 | -0.18638 | -4.22008 |
| 72 | 1 | 0 | 3.189112 | -3.1491 | -5.5704 |
| 73 | 1 | 0 | 5.598108 | -3.67825 | -5.12921 |
| 74 | 1 | 0 | 6.686889 | -2.8802 | -3.0372 |
| 75 | 1 | 0 | -1.43252 | 2.222846 | -0.86378 |
| 76 | 1 | 0 | -4.19872 | 2.091799 | 2.195855 |
| 77 | 1 | 0 | -3.85789 | 4.499638 | 2.726807 |
| 78 | 1 | 0 | -0.99438 | 4.635453 | -0.48899 |
| 79 | 1 | 0 | -5.57646 | -1.86631 | 0.681632 |
| 80 | 1 | 0 | -1.72647 | -2.52223 | -0.65656 |
| 81 | 1 | 0 | -6.20299 | -4.24578 | 0.31608 |
| 82 | 1 | 0 | -2.17917 | -4.92202 | -1.0656 |
| 83 | 1 | 0 | 4.911278 | -3.82819 | 2.862037 |
| 84 | 1 | 0 | 0.804795 | -4.13044 | 1.59593 |
| 85 | 1 | 0 | 1.093018 | -1.9545 | 0.432789 |
| 86 | 1 | 0 | 5.03279 | -1.67081 | 1.641314 |
| 87 | 1 | 0 | 5.214355 | 1.19921 | -2.11355 |
| 88 | 1 | 0 | 1.783028 | 2.713081 | -0.431 |
| 89 | 1 | 0 | 5.510432 | 3.198056 | -3.56699 |
| 90 | 1 | 0 | 1.940454 | 4.821003 | -1.79056 |
| 91 | 1 | 0 | -4.33115 | -0.74291 | 2.997415 |
| 92 | 1 | 0 | -0.42963 | -0.18464 | 1.752828 |
| 93 | 1 | 0 | -3.59485 | -1.3391 | 5.306921 |
| 94 | 1 | 0 | 0.462527 | -0.71677 | 3.992747 |
| 95 | 1 | 0 | 5.991238 | 0.799131 | 1.143611 |
| 96 | 1 | 0 | 2.153244 | 1.707935 | 2.294461 |
| 97 | 1 | 0 | 6.975043 | 2.096848 | 3.039948 |
| 98 | 1 | 0 | 2.955536 | 3.041684 | 4.262274 |
| 99 | 8 | 0 | 1.490684 | 0.756704 | 0.535861 |
| 100 | 1 | 0 | 0.775315 | 0.238368 | 0.101673 |
| 101 | 6 | 0 | 2.728121 | -5.52035 | 3.0242 |
| 102 | 1 | 0 | 3.139663 | -6.3084 | 2.382054 |
| 103 | 1 | 0 | 3.291971 | -5.53951 | 3.961662 |
| 104 | 1 | 0 | 1.688012 | -5.77965 | 3.239299 |
| 105 | 6 | 0 | 5.591154 | 3.511985 | 4.97278 |
| 106 | 1 | 0 | 5.913165 | 4.500959 | 4.62447 |
| 107 | 1 | 0 | 4.837809 | 3.665272 | 5.750157 |
| 108 | 1 | 0 | 6.462676 | 3.030433 | 5.42653 |
| 109 | 6 | 0 | -0.99197 | -1.45707 | 6.235489 |
| 110 | 1 | 0 | -0.95861 | -2.54646 | 6.359019 |
| 111 | 1 | 0 | 0.016656 | -1.07424 | 6.411964 |
| 112 | 1 | 0 | -1.65322 | -1.06394 | 7.013485 |
| 113 | 6 | 0 | -4.58061 | -6.2506 | -0.69419 |
| 114 | 1 | 0 | -4.9434 | -6.36657 | -1.72273 |
| 115 | 1 | 0 | -3.70432 | -6.89517 | -0.5829 |
| 116 | 1 | 0 | -5.36736 | -6.61298 | -0.0268 |
| 117 | 6 | 0 | -2.18471 | 6.265469 | 1.405283 |
| 118 | 1 | 0 | -1.16912 | 6.577816 | 1.146539 |
| 119 | 1 | 0 | -2.87583 | 6.852842 | 0.788557 |
| 120 | 1 | 0 | -2.37414 | 6.528521 | 2.449491 |
| 121 | 6 | 0 | 3.856896 | 5.413387 | -3.6945 |
| 122 | 1 | 0 | 3.383575 | 5.227727 | -4.66633 |
| 123 | 1 | 0 | 3.362071 | 6.280516 | -3.24842 |
| 124 | 1 | 0 | 4.902444 | 5.671204 | -3.8859 |

| Table S20. Cartesian coordinates for $Ru^{III}Ru^{III}$-6pic-$OH_2$-A. | | | | | |
|---|---|---|---|---|---|
| | | | Coordinates (Ångström) | | |
| Center number | Atomic number | Atomic type | X | Y | Z |
| 1 | 6 | 0 | 3.638704 | -1.49839 | -2.64862 |
| 2 | 6 | 0 | 3.03715 | -1.93067 | -3.84959 |
| 3 | 6 | 0 | 3.722243 | -2.63348 | -4.83968 |
| 4 | 6 | 0 | 5.062018 | -2.91547 | -4.58973 |
| 5 | 6 | 0 | 5.679553 | -2.49768 | -3.40096 |
| 6 | 6 | 0 | 5.000376 | -1.77456 | -2.41667 |
| 7 | 6 | 0 | 1.529155 | -0.90369 | -2.55217 |
| 8 | 6 | 0 | 5.773303 | -1.32674 | -1.20834 |
| 9 | 8 | 0 | 6.85646 | -1.82974 | -0.942 |
| 10 | 8 | 0 | 5.262116 | -0.33583 | -0.50108 |
| 11 | 7 | 0 | 2.676061 | -0.85309 | -1.86026 |
| 12 | 7 | 0 | 1.72087 | -1.52535 | -3.75536 |
| 13 | 6 | 0 | -0.77525 | 0.021311 | -3.13885 |
| 14 | 6 | 0 | -1.91629 | 0.201466 | -2.36235 |
| 15 | 7 | 0 | -0.35007 | -0.56192 | -0.97835 |
| 16 | 7 | 0 | -1.62534 | -0.15318 | -1.06139 |
| 17 | 6 | 0 | 0.184336 | -0.45153 | -2.22499 |
| 18 | 6 | 0 | -5.25589 | 1.058471 | -1.7167 |
| 19 | 6 | 0 | -5.21816 | 1.405675 | -3.0918 |
| 20 | 6 | 0 | -6.37333 | 1.935331 | -3.66602 |
| 21 | 6 | 0 | -7.49774 | 2.094731 | -2.83785 |
| 22 | 6 | 0 | -7.49675 | 1.746333 | -1.4751 |
| 23 | 6 | 0 | -6.35448 | 1.208367 | -0.86748 |
| 24 | 6 | 0 | -3.27882 | 0.653574 | -2.58165 |
| 25 | 7 | 0 | -4.02383 | 0.578549 | -1.41333 |
| 26 | 7 | 0 | -3.93967 | 1.137126 | -3.61363 |
| 27 | 6 | 0 | -6.31737 | 0.824979 | 0.594752 |
| 28 | 8 | 0 | -7.29781 | 1.03849 | 1.298405 |
| 29 | 8 | 0 | -5.22888 | 0.234909 | 1.088963 |
| 30 | 44 | 0 | -3.31068 | -0.03243 | 0.263198 |
| 31 | 44 | 0 | 3.31428 | 0.125768 | -0.05492 |
| 32 | 6 | 0 | -3.54307 | 2.500827 | 1.897011 |
| 33 | 6 | 0 | -1.95786 | 2.739292 | 0.226668 |
| 34 | 6 | 0 | -3.33439 | 3.803397 | 2.328648 |
| 35 | 6 | 0 | -1.70877 | 4.051919 | 0.603456 |
| 36 | 6 | 0 | -2.4038 | 4.622486 | 1.677231 |
| 37 | 6 | 0 | -5.03536 | -2.53072 | -0.00303 |
| 38 | 6 | 0 | -2.84193 | -2.95256 | -0.60716 |
| 39 | 6 | 0 | -5.39918 | -3.84238 | -0.2766 |
| 40 | 6 | 0 | -3.13963 | -4.27873 | -0.8912 |
| 41 | 6 | 0 | -4.44703 | -4.75834 | -0.73963 |
| 42 | 6 | 0 | 4.244842 | -2.27081 | 1.583221 |
| 43 | 6 | 0 | 2.020499 | -2.44913 | 0.966746 |
| 44 | 6 | 0 | 4.198833 | -3.4923 | 2.240256 |
| 45 | 6 | 0 | 1.908192 | -3.67602 | 1.609687 |
| 46 | 6 | 0 | 3.011101 | -4.23471 | 2.267001 |
| 47 | 6 | 0 | 4.625029 | 2.246507 | -1.84408 |

| | | | | | |
|---|---|---|---|---|---|
| 48 | 6 | 0 | 2.585449 | 2.941444 | -0.99908 |
| 49 | 6 | 0 | 4.789997 | 3.443673 | -2.52779 |
| 50 | 6 | 0 | 2.692467 | 4.159905 | -1.65708 |
| 51 | 6 | 0 | 3.809705 | 4.441122 | -2.45296 |
| 52 | 6 | 0 | -3.49478 | -1.02888 | 3.158018 |
| 53 | 6 | 0 | -1.28043 | -0.79926 | 2.525025 |
| 54 | 6 | 0 | -3.12526 | -1.47173 | 4.420731 |
| 55 | 6 | 0 | -0.84192 | -1.22856 | 3.773042 |
| 56 | 6 | 0 | -1.76941 | -1.58555 | 4.759101 |
| 57 | 6 | 0 | 5.41599 | 1.30821 | 1.841565 |
| 58 | 6 | 0 | 3.264546 | 1.545504 | 2.661447 |
| 59 | 6 | 0 | 5.945993 | 1.959091 | 2.947922 |
| 60 | 6 | 0 | 3.733278 | 2.201636 | 3.793094 |
| 61 | 6 | 0 | 5.104123 | 2.430335 | 3.963127 |
| 62 | 7 | 0 | 3.535467 | 1.987657 | -1.09001 |
| 63 | 7 | 0 | 3.171984 | -1.74718 | 0.950806 |
| 64 | 7 | 0 | 4.089442 | 1.100035 | 1.690956 |
| 65 | 7 | 0 | -2.58695 | -0.70111 | 2.211491 |
| 66 | 7 | 0 | -2.86281 | 1.964254 | 0.859045 |
| 67 | 7 | 0 | -3.7731 | -2.07775 | -0.17072 |
| 68 | 1 | 0 | -6.40485 | 2.217683 | -4.71352 |
| 69 | 1 | 0 | -8.40598 | 2.507054 | -3.26724 |
| 70 | 1 | 0 | -8.38313 | 1.889639 | -0.86647 |
| 71 | 1 | 0 | -0.66131 | 0.239172 | -4.19069 |
| 72 | 1 | 0 | 3.233232 | -2.95064 | -5.75514 |
| 73 | 1 | 0 | 5.639031 | -3.46567 | -5.32583 |
| 74 | 1 | 0 | 6.72329 | -2.72385 | -3.21297 |
| 75 | 1 | 0 | -1.43124 | 2.281096 | -0.60053 |
| 76 | 1 | 0 | -4.28575 | 1.857957 | 2.353881 |
| 77 | 1 | 0 | -3.91501 | 4.178582 | 3.165444 |
| 78 | 1 | 0 | -0.97469 | 4.629438 | 0.050513 |
| 79 | 1 | 0 | -5.74544 | -1.80597 | 0.374192 |
| 80 | 1 | 0 | -1.83893 | -2.56133 | -0.7243 |
| 81 | 1 | 0 | -6.43106 | -4.14268 | -0.1241 |
| 82 | 1 | 0 | -2.34693 | -4.9348 | -1.23759 |
| 83 | 1 | 0 | 5.097721 | -3.86236 | 2.723095 |
| 84 | 1 | 0 | 0.954728 | -4.19475 | 1.590446 |
| 85 | 1 | 0 | 1.180499 | -2.00593 | 0.444208 |
| 86 | 1 | 0 | 5.158699 | -1.69208 | 1.529919 |
| 87 | 1 | 0 | 5.372744 | 1.463877 | -1.87123 |
| 88 | 1 | 0 | 1.739004 | 2.681393 | -0.37318 |
| 89 | 1 | 0 | 5.690109 | 3.592767 | -3.11595 |
| 90 | 1 | 0 | 1.898146 | 4.891643 | -1.54513 |
| 91 | 1 | 0 | -4.53367 | -0.89736 | 2.870322 |
| 92 | 1 | 0 | -0.57677 | -0.51307 | 1.751572 |
| 93 | 1 | 0 | -3.89951 | -1.71944 | 5.140593 |
| 94 | 1 | 0 | 0.224579 | -1.28355 | 3.968189 |
| 95 | 1 | 0 | 6.042363 | 0.914213 | 1.049628 |
| 96 | 1 | 0 | 2.211037 | 1.362881 | 2.484101 |
| 97 | 1 | 0 | 7.021655 | 2.091897 | 3.012245 |
| 98 | 1 | 0 | 3.02062 | 2.534257 | 4.541875 |
| 99 | 8 | 0 | 1.552183 | 0.698318 | 0.581156 |
| 100 | 1 | 0 | 0.828333 | 0.196405 | 0.14976 |
| 101 | 6 | 0 | 2.938559 | -5.5769 | 2.944769 |
| 102 | 1 | 0 | 3.399249 | -6.34735 | 2.315129 |

| | | | | | |
|---|---|---|---|---|---|
| 103 | 1 | 0 | 3.480164 | -5.57214 | 3.895225 |
| 104 | 1 | 0 | 1.905285 | -5.87745 | 3.1348 |
| 105 | 6 | 0 | 5.646195 | 3.163484 | 5.161247 |
| 106 | 1 | 0 | 5.80299 | 4.222512 | 4.923044 |
| 107 | 1 | 0 | 4.957046 | 3.11491 | 6.008153 |
| 108 | 1 | 0 | 6.61143 | 2.756121 | 5.474668 |
| 109 | 6 | 0 | -1.34075 | -2.07498 | 6.117154 |
| 110 | 1 | 0 | -1.56243 | -3.14256 | 6.230477 |
| 111 | 1 | 0 | -0.26893 | -1.93533 | 6.276751 |
| 112 | 1 | 0 | -1.8794 | -1.55046 | 6.912591 |
| 113 | 6 | 0 | -4.81627 | -6.17738 | -1.07871 |
| 114 | 1 | 0 | -5.18667 | -6.23686 | -2.10932 |
| 115 | 1 | 0 | -3.9563 | -6.84736 | -0.99904 |
| 116 | 1 | 0 | -5.61068 | -6.54977 | -0.42639 |
| 117 | 6 | 0 | -2.18935 | 6.053777 | 2.089826 |
| 118 | 1 | 0 | -1.19891 | 6.412891 | 1.798452 |
| 119 | 1 | 0 | -2.93096 | 6.70188 | 1.607396 |
| 120 | 1 | 0 | -2.30286 | 6.179345 | 3.169994 |
| 121 | 6 | 0 | 3.943095 | 5.737464 | -3.20637 |
| 122 | 1 | 0 | 3.577882 | 5.621014 | -4.23394 |
| 123 | 1 | 0 | 3.360615 | 6.53548 | -2.73901 |
| 124 | 1 | 0 | 4.986608 | 6.058236 | -3.26692 |
| 125 | 1 | 0 | 0.970336 | -1.74706 | -4.39203 |

**Table S21.** Cartesian coordinates for $Ru^{III}Ru^{III}$-6pic-$H^+$-$OH_2$-A.

| Center number | Atomic number | Atomic type | Coordinates (Ångström) | | |
|---|---|---|---|---|---|
| | | | X | Y | Z |
| 1 | 6 | 0 | 3.672233 | -1.15754 | -2.81268 |
| 2 | 6 | 0 | 3.039575 | -1.66383 | -3.98924 |
| 3 | 6 | 0 | 3.796099 | -2.21602 | -5.04263 |
| 4 | 6 | 0 | 5.172449 | -2.25358 | -4.90015 |
| 5 | 6 | 0 | 5.800187 | -1.73601 | -3.74454 |
| 6 | 6 | 0 | 5.07974 | -1.16734 | -2.69393 |
| 7 | 6 | 0 | 1.522584 | -0.96136 | -2.66603 |
| 8 | 6 | 0 | 5.818929 | -0.56357 | -1.55379 |
| 9 | 8 | 0 | 7.002154 | -0.76603 | -1.34446 |
| 10 | 8 | 0 | 5.120802 | 0.291342 | -0.78699 |
| 11 | 7 | 0 | 2.684853 | -0.71712 | -1.95719 |
| 12 | 7 | 0 | 1.694151 | -1.51804 | -3.87532 |
| 13 | 6 | 0 | -0.96312 | -0.86612 | -3.12131 |
| 14 | 6 | 0 | -2.06184 | -0.61149 | -2.32291 |
| 15 | 7 | 0 | -0.30968 | -0.41979 | -1.03303 |
| 16 | 7 | 0 | -1.66333 | -0.34704 | -1.01529 |
| 17 | 6 | 0 | 0.152812 | -0.73462 | -2.26752 |
| 18 | 6 | 0 | -5.4988 | -0.27885 | -1.66673 |
| 19 | 6 | 0 | -5.66837 | -0.59392 | -3.02894 |
| 20 | 6 | 0 | -6.95542 | -0.68303 | -3.56459 |
| 21 | 6 | 0 | -8.01275 | -0.45479 | -2.67876 |
| 22 | 6 | 0 | -7.82277 | -0.15336 | -1.31223 |
| 23 | 6 | 0 | -6.54309 | -0.05617 | -0.76525 |

| | | | | | |
|---|---|---|---|---|---|
| 24 | 6 | 0 | -3.49514 | -0.55456 | -2.50325 |
| 25 | 7 | 0 | -4.166 | -0.25971 | -1.40036 |
| 26 | 7 | 0 | -4.37026 | -0.76407 | -3.53341 |
| 27 | 6 | 0 | -6.31018 | 0.254224 | 0.694561 |
| 28 | 8 | 0 | -7.26017 | 0.382649 | 1.4441 |
| 29 | 8 | 0 | -5.05578 | 0.41792 | 1.168303 |
| 30 | 44 | 0 | -3.24607 | 0.051812 | 0.336954 |
| 31 | 44 | 0 | 3.275916 | 0.109195 | -0.09194 |
| 32 | 6 | 0 | -4.04592 | 3.026629 | 0.34015 |
| 33 | 6 | 0 | -2.02257 | 2.706785 | -0.72451 |
| 34 | 6 | 0 | -3.99818 | 4.391686 | 0.096576 |
| 35 | 6 | 0 | -1.91302 | 4.063593 | -0.99838 |
| 36 | 6 | 0 | -2.91742 | 4.952863 | -0.59588 |
| 37 | 6 | 0 | -4.22659 | -2.31465 | 1.947303 |
| 38 | 6 | 0 | -3.05227 | -3.02888 | 0.085008 |
| 39 | 6 | 0 | -4.49228 | -3.62288 | 2.322484 |
| 40 | 6 | 0 | -3.28108 | -4.35909 | 0.407038 |
| 41 | 6 | 0 | -4.02435 | -4.69364 | 1.547523 |
| 42 | 6 | 0 | 4.916135 | -2.20987 | 1.009338 |
| 43 | 6 | 0 | 2.746172 | -2.83266 | 0.501009 |
| 44 | 6 | 0 | 5.23177 | -3.50246 | 1.402559 |
| 45 | 6 | 0 | 2.995912 | -4.14385 | 0.880935 |
| 46 | 6 | 0 | 4.266247 | -4.5162 | 1.34158 |
| 47 | 6 | 0 | 3.350539 | 2.438889 | -2.0348 |
| 48 | 6 | 0 | 2.440514 | 3.092265 | -0.01669 |
| 49 | 6 | 0 | 3.265374 | 3.731574 | -2.53142 |
| 50 | 6 | 0 | 2.319373 | 4.406859 | -0.45122 |
| 51 | 6 | 0 | 2.743748 | 4.763205 | -1.73823 |
| 52 | 6 | 0 | -3.03391 | 1.086066 | 3.210098 |
| 53 | 6 | 0 | -1.23062 | -0.25959 | 2.690687 |
| 54 | 6 | 0 | -2.5919 | 1.238817 | 4.516756 |
| 55 | 6 | 0 | -0.73624 | -0.15625 | 3.985902 |
| 56 | 6 | 0 | -1.41712 | 0.605179 | 4.944534 |
| 57 | 6 | 0 | 5.135356 | 1.732176 | 1.708269 |
| 58 | 6 | 0 | 3.589344 | 0.536673 | 2.947893 |
| 59 | 6 | 0 | 5.730223 | 2.221988 | 2.862705 |
| 60 | 6 | 0 | 4.138178 | 0.991034 | 4.14002 |
| 61 | 6 | 0 | 5.237833 | 1.86026 | 4.124448 |
| 62 | 7 | 0 | 2.94807 | 2.109392 | -0.78613 |
| 63 | 7 | 0 | 3.687194 | -1.86866 | 0.559571 |
| 64 | 7 | 0 | 4.067815 | 0.90236 | 1.739686 |
| 65 | 7 | 0 | -2.36713 | 0.348981 | 2.292696 |
| 66 | 7 | 0 | -3.07536 | 2.174239 | -0.06526 |
| 67 | 7 | 0 | -3.51509 | -2.00615 | 0.837079 |
| 68 | 1 | 0 | -7.13961 | -0.91986 | -4.60693 |
| 69 | 1 | 0 | -9.02706 | -0.51727 | -3.05883 |
| 70 | 1 | 0 | -8.67757 | 0.00531 | -0.66378 |
| 71 | 1 | 0 | -0.91035 | -1.11732 | -4.16865 |
| 72 | 1 | 0 | 3.3005 | -2.59617 | -5.92982 |
| 73 | 1 | 0 | 5.787577 | -2.67424 | -5.68884 |
| 74 | 1 | 0 | 6.881352 | -1.75556 | -3.655 |
| 75 | 1 | 0 | -1.24888 | 2.018214 | -1.03532 |
| 76 | 1 | 0 | -4.8755 | 2.580419 | 0.872498 |
| 77 | 1 | 0 | -4.81538 | 5.01307 | 0.449175 |
| 78 | 1 | 0 | -1.03698 | 4.419673 | -1.53097 |

| | | | | | |
|---|---|---|---|---|---|
| 79 | 1 | 0 | -4.60421 | -1.4761 | 2.518418 |
| 80 | 1 | 0 | -2.47894 | -2.76272 | -0.79376 |
| 81 | 1 | 0 | -5.07637 | -3.80053 | 3.219778 |
| 82 | 1 | 0 | -2.88023 | -5.13289 | -0.23986 |
| 83 | 1 | 0 | 6.239517 | -3.71389 | 1.745223 |
| 84 | 1 | 0 | 2.198941 | -4.877 | 0.807218 |
| 85 | 1 | 0 | 1.774306 | -2.52482 | 0.137473 |
| 86 | 1 | 0 | 5.660015 | -1.42273 | 1.025007 |
| 87 | 1 | 0 | 3.765322 | 1.636857 | -2.6305 |
| 88 | 1 | 0 | 2.14941 | 2.817185 | 0.99129 |
| 89 | 1 | 0 | 3.621355 | 3.930825 | -3.53734 |
| 90 | 1 | 0 | 1.917276 | 5.153216 | 0.226697 |
| 91 | 1 | 0 | -3.96497 | 1.527928 | 2.878097 |
| 92 | 1 | 0 | -0.70006 | -0.84021 | 1.945269 |
| 93 | 1 | 0 | -3.17859 | 1.841889 | 5.202745 |
| 94 | 1 | 0 | 0.175076 | -0.686 | 4.245088 |
| 95 | 1 | 0 | 5.525201 | 1.95943 | 0.723969 |
| 96 | 1 | 0 | 2.752027 | -0.15258 | 2.934628 |
| 97 | 1 | 0 | 6.591423 | 2.876374 | 2.770953 |
| 98 | 1 | 0 | 3.716286 | 0.652299 | 5.081011 |
| 99 | 8 | 0 | 1.419098 | -0.01318 | 0.78051 |
| 100 | 1 | 0 | 0.337916 | -0.23216 | -0.16786 |
| 101 | 1 | 0 | 1.231581 | 0.785271 | 1.292649 |
| 102 | 6 | 0 | 4.589655 | -5.93475 | 1.72243 |
| 103 | 1 | 0 | 5.093364 | -6.44277 | 0.891299 |
| 104 | 1 | 0 | 5.265824 | -5.97292 | 2.580813 |
| 105 | 1 | 0 | 3.688628 | -6.50468 | 1.961312 |
| 106 | 6 | 0 | 5.858394 | 2.383547 | 5.390863 |
| 107 | 1 | 0 | 5.553918 | 3.422376 | 5.565648 |
| 108 | 1 | 0 | 5.556914 | 1.796446 | 6.261253 |
| 109 | 1 | 0 | 6.950229 | 2.37522 | 5.32767 |
| 110 | 6 | 0 | -0.93426 | 0.724799 | 6.364372 |
| 111 | 1 | 0 | -1.61857 | 0.207317 | 7.046201 |
| 112 | 1 | 0 | 0.059133 | 0.289352 | 6.493048 |
| 113 | 1 | 0 | -0.89918 | 1.771689 | 6.681875 |
| 114 | 6 | 0 | -4.32941 | -6.1206 | 1.907618 |
| 115 | 1 | 0 | -5.32537 | -6.39589 | 1.539826 |
| 116 | 1 | 0 | -3.61077 | -6.81228 | 1.462119 |
| 117 | 1 | 0 | -4.33386 | -6.26666 | 2.991023 |
| 118 | 6 | 0 | -2.85719 | 6.422995 | -0.90559 |
| 119 | 1 | 0 | -1.84246 | 6.743394 | -1.15251 |
| 120 | 1 | 0 | -3.49718 | 6.654963 | -1.76529 |
| 121 | 1 | 0 | -3.21886 | 7.02164 | -0.06499 |
| 122 | 6 | 0 | 2.653922 | 6.175897 | -2.24911 |
| 123 | 1 | 0 | 1.922523 | 6.248927 | -3.06234 |
| 124 | 1 | 0 | 2.360161 | 6.873936 | -1.46214 |
| 125 | 1 | 0 | 3.614861 | 6.504201 | -2.65715 |
| 126 | 1 | 0 | -4.12395 | -0.99601 | -4.48498 |

**Table S22.** Cartesian coordinates for $Ru^{III}Ru^{III}$-6pic-$2H^+$-$OH_2$.

| | | | Coordinates (Ångström) | | |
|---|---|---|---|---|---|
| **Center** | **Atomic** | **Atomic** | **X** | **Y** | **Z** |

| number | number | type | | | |
|---|---|---|---|---|---|
| 1 | 6 | 0 | 3.69709 | -1.17941 | -2.81642 |
| 2 | 6 | 0 | 3.095334 | -1.74419 | -3.95654 |
| 3 | 6 | 0 | 3.820376 | -2.38002 | -4.96741 |
| 4 | 6 | 0 | 5.199565 | -2.43528 | -4.80171 |
| 5 | 6 | 0 | 5.823425 | -1.86301 | -3.67812 |
| 6 | 6 | 0 | 5.098341 | -1.21656 | -2.67566 |
| 7 | 6 | 0 | 1.536053 | -0.87272 | -2.60644 |
| 8 | 6 | 0 | 5.840655 | -0.57283 | -1.55673 |
| 9 | 8 | 0 | 7.011116 | -0.79177 | -1.32891 |
| 10 | 8 | 0 | 5.166273 | 0.344162 | -0.82738 |
| 11 | 7 | 0 | 2.703733 | -0.65415 | -1.98104 |
| 12 | 7 | 0 | 1.742067 | -1.52038 | -3.79072 |
| 13 | 6 | 0 | -0.88136 | -0.27423 | -3.15071 |
| 14 | 6 | 0 | -1.9963 | -0.08451 | -2.34049 |
| 15 | 7 | 0 | -0.3083 | -0.49116 | -0.96984 |
| 16 | 7 | 0 | -1.6274 | -0.22369 | -1.01263 |
| 17 | 6 | 0 | 0.163043 | -0.52472 | -2.24228 |
| 18 | 6 | 0 | -5.40691 | 0.540821 | -1.72466 |
| 19 | 6 | 0 | -5.51422 | 0.652931 | -3.12317 |
| 20 | 6 | 0 | -6.75222 | 0.930601 | -3.7085 |
| 21 | 6 | 0 | -7.83019 | 1.081925 | -2.83198 |
| 22 | 6 | 0 | -7.70539 | 0.96581 | -1.42955 |
| 23 | 6 | 0 | -6.47522 | 0.684635 | -0.83461 |
| 24 | 6 | 0 | -3.39868 | 0.206225 | -2.54021 |
| 25 | 7 | 0 | -4.10656 | 0.275603 | -1.41932 |
| 26 | 7 | 0 | -4.21554 | 0.433915 | -3.61173 |
| 27 | 6 | 0 | -6.31461 | 0.558253 | 0.657039 |
| 28 | 8 | 0 | -7.24261 | 0.798186 | 1.401599 |
| 29 | 8 | 0 | -5.13163 | 0.152855 | 1.187368 |
| 30 | 44 | 0 | -3.31491 | -0.0699 | 0.374438 |
| 31 | 44 | 0 | 3.360281 | 0.214973 | -0.11485 |
| 32 | 6 | 0 | -3.65365 | 2.674707 | 1.655529 |
| 33 | 6 | 0 | -2.27481 | 2.801897 | -0.19691 |
| 34 | 6 | 0 | -3.59056 | 4.046664 | 1.842028 |
| 35 | 6 | 0 | -2.16838 | 4.179154 | -0.06541 |
| 36 | 6 | 0 | -2.84193 | 4.848158 | 0.967566 |
| 37 | 6 | 0 | -4.62263 | -2.71759 | 1.077984 |
| 38 | 6 | 0 | -3.21924 | -2.97339 | -0.7439 |
| 39 | 6 | 0 | -4.9862 | -4.0539 | 1.028216 |
| 40 | 6 | 0 | -3.54082 | -4.31874 | -0.84704 |
| 41 | 6 | 0 | -4.45081 | -4.90176 | 0.046956 |
| 42 | 6 | 0 | 4.943546 | -2.07961 | 1.108146 |
| 43 | 6 | 0 | 2.797081 | -2.73026 | 0.527847 |
| 44 | 6 | 0 | 5.246121 | -3.35622 | 1.55629 |
| 45 | 6 | 0 | 3.034498 | -4.02518 | 0.964857 |
| 46 | 6 | 0 | 4.286456 | -4.37755 | 1.492037 |
| 47 | 6 | 0 | 3.428595 | 2.552523 | -2.07316 |
| 48 | 6 | 0 | 2.610959 | 3.218429 | -0.01828 |
| 49 | 6 | 0 | 3.372535 | 3.851571 | -2.55576 |
| 50 | 6 | 0 | 2.522533 | 4.53832 | -0.43737 |
| 51 | 6 | 0 | 2.918872 | 4.895094 | -1.73514 |
| 52 | 6 | 0 | -3.34542 | -0.34279 | 3.448578 |
| 53 | 6 | 0 | -1.35525 | -1.09137 | 2.547367 |
| 54 | 6 | 0 | -2.94362 | -0.70128 | 4.726945 |

| | | | | | |
|---|---|---|---|---|---|
| 55 | 6 | 0 | -0.89211 | -1.47925 | 3.798501 |
| 56 | 6 | 0 | -1.68661 | -1.28784 | 4.937534 |
| 57 | 6 | 0 | 5.238393 | 1.841525 | 1.639219 |
| 58 | 6 | 0 | 3.677964 | 0.677311 | 2.898359 |
| 59 | 6 | 0 | 5.831658 | 2.347686 | 2.785783 |
| 60 | 6 | 0 | 4.224155 | 1.154469 | 4.081923 |
| 61 | 6 | 0 | 5.332319 | 2.014427 | 4.054599 |
| 62 | 7 | 0 | 3.057295 | 2.222756 | -0.81453 |
| 63 | 7 | 0 | 3.732344 | -1.75737 | 0.592707 |
| 64 | 7 | 0 | 4.164323 | 1.017102 | 1.683257 |
| 65 | 7 | 0 | -2.56362 | -0.52192 | 2.357644 |
| 66 | 7 | 0 | -3.00586 | 2.041148 | 0.648441 |
| 67 | 7 | 0 | -3.74486 | -2.16602 | 0.204596 |
| 68 | 1 | 0 | -6.88547 | 1.025122 | -4.78076 |
| 69 | 1 | 0 | -8.80846 | 1.297803 | -3.24837 |
| 70 | 1 | 0 | -8.5743 | 1.094008 | -0.79301 |
| 71 | 1 | 0 | -0.82435 | -0.20182 | -4.22715 |
| 72 | 1 | 0 | 3.335758 | -2.80868 | -5.83829 |
| 73 | 1 | 0 | 5.809077 | -2.91834 | -5.55776 |
| 74 | 1 | 0 | 6.902511 | -1.90034 | -3.57424 |
| 75 | 1 | 0 | -1.76965 | 2.280286 | -0.99958 |
| 76 | 1 | 0 | -4.26335 | 2.054086 | 2.297292 |
| 77 | 1 | 0 | -4.1462 | 4.488802 | 2.662892 |
| 78 | 1 | 0 | -1.57231 | 4.732067 | -0.78437 |
| 79 | 1 | 0 | -5.05353 | -2.04655 | 1.809114 |
| 80 | 1 | 0 | -2.51958 | -2.51818 | -1.43201 |
| 81 | 1 | 0 | -5.70071 | -4.42826 | 1.754445 |
| 82 | 1 | 0 | -3.08391 | -4.91132 | -1.63308 |
| 83 | 1 | 0 | 6.239066 | -3.55088 | 1.948711 |
| 84 | 1 | 0 | 2.242798 | -4.76332 | 0.886008 |
| 85 | 1 | 0 | 1.837631 | -2.44815 | 0.110863 |
| 86 | 1 | 0 | 5.684245 | -1.28991 | 1.135241 |
| 87 | 1 | 0 | 3.799968 | 1.745876 | -2.69133 |
| 88 | 1 | 0 | 2.346598 | 2.947714 | 0.998298 |
| 89 | 1 | 0 | 3.699222 | 4.048666 | -3.57189 |
| 90 | 1 | 0 | 2.166493 | 5.290823 | 0.259028 |
| 91 | 1 | 0 | -4.33092 | 0.068406 | 3.261819 |
| 92 | 1 | 0 | -0.75504 | -1.24934 | 1.659006 |
| 93 | 1 | 0 | -3.62308 | -0.53317 | 5.556851 |
| 94 | 1 | 0 | 0.084677 | -1.94592 | 3.879768 |
| 95 | 1 | 0 | 5.630742 | 2.058806 | 0.654037 |
| 96 | 1 | 0 | 2.837808 | -0.00828 | 2.904266 |
| 97 | 1 | 0 | 6.696156 | 2.996193 | 2.683887 |
| 98 | 1 | 0 | 3.792102 | 0.842006 | 5.027037 |
| 99 | 8 | 0 | 1.4324 | 0.164511 | 0.783598 |
| 100 | 1 | 0 | 0.659845 | -0.14056 | 0.14633 |
| 101 | 1 | 0 | 1.155682 | 0.991668 | 1.201483 |
| 102 | 6 | 0 | 4.597366 | -5.77621 | 1.943203 |
| 103 | 1 | 0 | 5.234565 | -6.27688 | 1.20436 |
| 104 | 1 | 0 | 5.147195 | -5.77454 | 2.888763 |
| 105 | 1 | 0 | 3.692673 | -6.37539 | 2.064961 |
| 106 | 6 | 0 | 5.962683 | 2.546995 | 5.310083 |
| 107 | 1 | 0 | 5.891297 | 3.639803 | 5.343789 |
| 108 | 1 | 0 | 5.487115 | 2.145339 | 6.206719 |
| 109 | 1 | 0 | 7.028394 | 2.297552 | 5.343485 |

| | | | | | |
|---|---|---|---|---|---|
| 110 | 6 | 0 | -1.23403 | -1.69668 | 6.31136 |
| 111 | 1 | 0 | -1.93559 | -2.41264 | 6.752424 |
| 112 | 1 | 0 | -0.2437 | -2.15622 | 6.294365 |
| 113 | 1 | 0 | -1.20177 | -0.83139 | 6.982323 |
| 114 | 6 | 0 | -4.85458 | -6.34485 | -0.05197 |
| 115 | 1 | 0 | -5.84882 | -6.42595 | -0.50786 |
| 116 | 1 | 0 | -4.15917 | -6.92085 | -0.66594 |
| 117 | 1 | 0 | -4.91832 | -6.80748 | 0.936983 |
| 118 | 6 | 0 | -2.79713 | 6.342095 | 1.116371 |
| 119 | 1 | 0 | -1.99443 | 6.787056 | 0.524611 |
| 120 | 1 | 0 | -3.7433 | 6.781041 | 0.777618 |
| 121 | 1 | 0 | -2.66855 | 6.633745 | 2.162754 |
| 122 | 6 | 0 | 2.894641 | 6.319656 | -2.2122 |
| 123 | 1 | 0 | 2.593699 | 6.385203 | -3.2612 |
| 124 | 1 | 0 | 2.223118 | 6.937514 | -1.61205 |
| 125 | 1 | 0 | 3.898289 | 6.755864 | -2.13829 |
| 126 | 1 | 0 | -3.94036 | 0.44408 | -4.58352 |
| 127 | 1 | 0 | 1.00558 | -1.8626 | -4.39062 |

**Table S23.** Cartesian coordinates for Ru$^{III}$Ru$^{III}$-5pic-OH-A.

| Center number | Atomic number | Atomic type | Coordinates (Ångström) | | |
|---|---|---|---|---|---|
| | | | X | Y | Z |
| 1 | 6 | 0 | -4.39626 | 0.915432 | -1.77971 |
| 2 | 6 | 0 | -4.42703 | 1.207747 | -3.17264 |
| 3 | 6 | 0 | -5.67192 | 1.456939 | -3.75587 |
| 4 | 6 | 0 | -6.80732 | 1.388004 | -2.93306 |
| 5 | 6 | 0 | -6.7404 | 1.070558 | -1.5628 |
| 6 | 6 | 0 | -5.51213 | 0.815752 | -0.94111 |
| 7 | 6 | 0 | -2.38658 | 0.86241 | -2.64014 |
| 8 | 6 | 0 | -5.40189 | 0.424887 | 0.511893 |
| 9 | 8 | 0 | -6.41374 | 0.353086 | 1.201518 |
| 10 | 8 | 0 | -4.20133 | 0.154458 | 1.02929 |
| 11 | 7 | 0 | -3.09549 | 0.727521 | -1.46426 |
| 12 | 7 | 0 | -3.13131 | 1.157863 | -3.69505 |
| 13 | 6 | 0 | 0.123118 | 0.506899 | -3.28657 |
| 14 | 6 | 0 | 1.183162 | 0.173875 | -2.44366 |
| 15 | 7 | 0 | -0.60316 | 0.280052 | -1.13959 |
| 16 | 7 | 0 | 0.731324 | 0.056564 | -1.14284 |
| 17 | 6 | 0 | -0.98043 | 0.585286 | -2.44224 |
| 18 | 6 | 0 | 4.584596 | -0.18449 | -1.75805 |
| 19 | 6 | 0 | 4.663863 | -0.15416 | -3.1823 |
| 20 | 6 | 0 | 5.939138 | -0.24445 | -3.76291 |
| 21 | 6 | 0 | 7.042649 | -0.3453 | -2.91128 |
| 22 | 6 | 0 | 6.924368 | -0.34862 | -1.50302 |
| 23 | 6 | 0 | 5.675658 | -0.26101 | -0.88547 |
| 24 | 6 | 0 | 2.613234 | 0.01818 | -2.63132 |
| 25 | 7 | 0 | 3.276602 | -0.08622 | -1.44742 |
| 26 | 7 | 0 | 3.391216 | -0.02539 | -3.71661 |
| 27 | 6 | 0 | 5.506947 | -0.21687 | 0.608109 |
| 28 | 8 | 0 | 6.475556 | -0.34434 | 1.349336 |

| | | | | | |
|---|---|---|---|---|---|
| 29 | 8 | 0 | 4.289872 | -0.01148 | 1.121066 |
| 30 | 44 | 0 | 2.398864 | 0.028608 | 0.356508 |
| 31 | 44 | 0 | -2.30737 | 0.085387 | 0.187602 |
| 32 | 6 | 0 | 3.118874 | -2.6412 | 1.538471 |
| 33 | 6 | 0 | 1.879005 | -2.9006 | -0.39596 |
| 34 | 6 | 0 | 3.239634 | -4.01811 | 1.685542 |
| 35 | 6 | 0 | 1.964726 | -4.28454 | -0.30924 |
| 36 | 6 | 0 | 2.659125 | -4.88277 | 0.749738 |
| 37 | 6 | 0 | 3.122803 | 2.708264 | 1.43047 |
| 38 | 6 | 0 | 2.223462 | 2.943113 | -0.68621 |
| 39 | 6 | 0 | 3.362287 | 4.074553 | 1.516854 |
| 40 | 6 | 0 | 2.434706 | 4.316245 | -0.66444 |
| 41 | 6 | 0 | 3.02507 | 4.918883 | 0.452365 |
| 42 | 6 | 0 | -2.85592 | 3.018396 | 0.703923 |
| 43 | 6 | 0 | -0.83017 | 2.332476 | 1.605581 |
| 44 | 6 | 0 | -2.68001 | 4.306379 | 1.188907 |
| 45 | 6 | 0 | -0.60202 | 3.605332 | 2.118365 |
| 46 | 6 | 0 | -1.53215 | 4.631146 | 1.922278 |
| 47 | 6 | 0 | -3.62368 | -2.64788 | 0.21042 |
| 48 | 6 | 0 | -2.10475 | -2.5001 | -1.52815 |
| 49 | 6 | 0 | -3.98151 | -3.93534 | -0.16611 |
| 50 | 6 | 0 | -2.41801 | -3.78318 | -1.95829 |
| 51 | 6 | 0 | -3.38146 | -4.53809 | -1.27882 |
| 52 | 6 | 0 | -2.32195 | -0.3557 | 3.214943 |
| 53 | 6 | 0 | -0.79816 | -1.7663 | 2.210741 |
| 54 | 6 | 0 | -2.08565 | -0.92761 | 4.459302 |
| 55 | 6 | 0 | -0.52867 | -2.40113 | 3.417989 |
| 56 | 6 | 0 | -1.17385 | -1.98193 | 4.588588 |
| 57 | 7 | 0 | -2.69346 | -1.92949 | -0.45695 |
| 58 | 7 | 0 | -1.94853 | 2.038889 | 0.907244 |
| 59 | 7 | 0 | -1.68403 | -0.75911 | 2.096372 |
| 60 | 7 | 0 | 2.437425 | -2.0781 | 0.516197 |
| 61 | 7 | 0 | 2.558709 | 2.1413 | 0.34333 |
| 62 | 1 | 0 | 6.064279 | -0.22901 | -4.84177 |
| 63 | 1 | 0 | 8.03579 | -0.41391 | -3.3469 |
| 64 | 1 | 0 | 7.807307 | -0.41117 | -0.87527 |
| 65 | 1 | 0 | 0.15679 | 0.681996 | -4.35042 |
| 66 | 1 | 0 | -5.76036 | 1.687943 | -4.81306 |
| 67 | 1 | 0 | -7.78206 | 1.576748 | -3.37423 |
| 68 | 1 | 0 | -7.64256 | 1.006955 | -0.96366 |
| 69 | 1 | 0 | 1.356688 | -2.41432 | -1.21057 |
| 70 | 1 | 0 | 3.604927 | -1.94677 | 2.214799 |
| 71 | 1 | 0 | 3.807163 | -4.40856 | 2.524897 |
| 72 | 1 | 0 | 1.495249 | -4.89123 | -1.07766 |
| 73 | 1 | 0 | 3.389792 | 2.026302 | 2.227441 |
| 74 | 1 | 0 | 1.775675 | 2.462579 | -1.54648 |
| 75 | 1 | 0 | 3.828801 | 4.4709 | 2.413815 |
| 76 | 1 | 0 | 2.142968 | 4.907009 | -1.52729 |
| 77 | 1 | 0 | -3.44532 | 5.050305 | 0.990153 |
| 78 | 1 | 0 | 0.318002 | 3.78332 | 2.665621 |
| 79 | 1 | 0 | -0.10815 | 1.531435 | 1.757499 |
| 80 | 1 | 0 | -3.73742 | 2.751142 | 0.136526 |
| 81 | 1 | 0 | -4.09469 | -2.14346 | 1.046098 |
| 82 | 1 | 0 | -1.37165 | -1.89856 | -2.05 |
| 83 | 1 | 0 | -4.74052 | -4.45808 | 0.407584 |

| | | | | | |
|---|---|---|---|---|---|
| 84 | 1 | 0 | -1.91264 | -4.18275 | -2.83206 |
| 85 | 1 | 0 | -3.06143 | 0.424291 | 3.079294 |
| 86 | 1 | 0 | -0.29317 | -2.06249 | 1.300186 |
| 87 | 1 | 0 | -2.62698 | -0.55271 | 5.322546 |
| 88 | 1 | 0 | 0.192291 | -3.21279 | 3.439168 |
| 89 | 8 | 0 | 1.466844 | 0.260101 | 2.108293 |
| 90 | 1 | 0 | 1.442469 | -0.55213 | 2.631651 |
| 91 | 6 | 0 | 3.315825 | 6.396825 | 0.491727 |
| 92 | 1 | 0 | 4.315203 | 6.600612 | 0.088396 |
| 93 | 1 | 0 | 2.599582 | 6.9628 | -0.11053 |
| 94 | 1 | 0 | 3.293298 | 6.783279 | 1.514697 |
| 95 | 6 | 0 | -1.32564 | 6.011499 | 2.489112 |
| 96 | 1 | 0 | -1.68256 | 6.782808 | 1.800157 |
| 97 | 1 | 0 | -1.88348 | 6.129958 | 3.425972 |
| 98 | 1 | 0 | -0.27123 | 6.200668 | 2.706211 |
| 99 | 6 | 0 | -0.92287 | -2.6484 | 5.915984 |
| 100 | 1 | 0 | -1.70902 | -3.38179 | 6.133017 |
| 101 | 1 | 0 | 0.034315 | -3.17612 | 5.926096 |
| 102 | 1 | 0 | -0.92493 | -1.92019 | 6.732142 |
| 103 | 6 | 0 | -3.77987 | -5.9145 | -1.74083 |
| 104 | 1 | 0 | -4.03665 | -6.56079 | -0.89681 |
| 105 | 1 | 0 | -4.66369 | -5.85576 | -2.38752 |
| 106 | 1 | 0 | -2.98279 | -6.39186 | -2.31675 |
| 107 | 6 | 0 | 2.810181 | -6.37795 | 0.853811 |
| 108 | 1 | 0 | 1.97483 | -6.90012 | 0.379092 |
| 109 | 1 | 0 | 3.729224 | -6.7046 | 0.352335 |
| 110 | 1 | 0 | 2.87541 | -6.70252 | 1.896157 |

**Table S24.** Cartesian coordinates for Ru$^{III}$Ru$^{III}$-5pic-OH$_2$-A.

| Center number | Atomic number | Atomic type | Coordinates (Ångström) | | |
|---|---|---|---|---|---|
| | | | X | Y | Z |
| 1 | 6 | 0 | 4.310276 | -1.00178 | -1.89355 |
| 2 | 6 | 0 | 4.290286 | -1.27236 | -3.28506 |
| 3 | 6 | 0 | 5.498808 | -1.55962 | -3.91868 |
| 4 | 6 | 0 | 6.6636 | -1.54925 | -3.13226 |
| 5 | 6 | 0 | 6.650747 | -1.25406 | -1.75768 |
| 6 | 6 | 0 | 5.452301 | -0.96176 | -1.09191 |
| 7 | 6 | 0 | 2.275587 | -0.85905 | -2.69357 |
| 8 | 6 | 0 | 5.406434 | -0.60124 | 0.375142 |
| 9 | 8 | 0 | 6.444511 | -0.58223 | 1.023947 |
| 10 | 8 | 0 | 4.236676 | -0.30929 | 0.940597 |
| 11 | 7 | 0 | 3.019559 | -0.76313 | -1.53146 |
| 12 | 7 | 0 | 2.974034 | -1.16451 | -3.76744 |
| 13 | 6 | 0 | -0.22992 | -0.48471 | -3.31851 |
| 14 | 6 | 0 | -1.27832 | -0.14407 | -2.46552 |
| 15 | 7 | 0 | 0.511891 | -0.251 | -1.17847 |
| 16 | 7 | 0 | -0.81639 | -0.02349 | -1.16839 |
| 17 | 6 | 0 | 0.87555 | -0.55791 | -2.47322 |
| 18 | 6 | 0 | -4.70763 | 0.219957 | -1.74978 |
| 19 | 6 | 0 | -4.87084 | 0.15301 | -3.14727 |

| | | | | | |
|---|---|---|---|---|---|
| 20 | 6 | 0 | -6.1478 | 0.210315 | -3.70732 |
| 21 | 6 | 0 | -7.21535 | 0.32404 | -2.81093 |
| 22 | 6 | 0 | -7.03624 | 0.370615 | -1.4133 |
| 23 | 6 | 0 | -5.76425 | 0.313871 | -0.84328 |
| 24 | 6 | 0 | -2.7045 | 0.014409 | -2.60122 |
| 25 | 7 | 0 | -3.37308 | 0.14548 | -1.46737 |
| 26 | 7 | 0 | -3.57341 | 0.021348 | -3.66169 |
| 27 | 6 | 0 | -5.54833 | 0.329742 | 0.654724 |
| 28 | 8 | 0 | -6.51125 | 0.459959 | 1.395371 |
| 29 | 8 | 0 | -4.31789 | 0.192041 | 1.133113 |
| 30 | 44 | 0 | -2.43807 | 0.067606 | 0.346468 |
| 31 | 44 | 0 | 2.325913 | -0.1405 | 0.150113 |
| 32 | 6 | 0 | -3.05712 | 2.777271 | 1.512982 |
| 33 | 6 | 0 | -1.70984 | 2.990819 | -0.35376 |
| 34 | 6 | 0 | -3.0935 | 4.155999 | 1.684731 |
| 35 | 6 | 0 | -1.70889 | 4.375015 | -0.24135 |
| 36 | 6 | 0 | -2.41579 | 4.999357 | 0.795777 |
| 37 | 6 | 0 | -3.24536 | -2.55813 | 1.504799 |
| 38 | 6 | 0 | -2.29381 | -2.90128 | -0.57405 |
| 39 | 6 | 0 | -3.49772 | -3.9169 | 1.649365 |
| 40 | 6 | 0 | -2.51798 | -4.26941 | -0.49406 |
| 41 | 6 | 0 | -3.14369 | -4.81463 | 0.634663 |
| 42 | 6 | 0 | 2.826311 | -3.0743 | 0.715586 |
| 43 | 6 | 0 | 0.801252 | -2.35614 | 1.590247 |
| 44 | 6 | 0 | 2.645988 | -4.34538 | 1.240485 |
| 45 | 6 | 0 | 0.571286 | -3.60774 | 2.150584 |
| 46 | 6 | 0 | 1.500062 | -4.64222 | 1.990033 |
| 47 | 6 | 0 | 3.786623 | 2.515679 | 0.254836 |
| 48 | 6 | 0 | 2.260432 | 2.503324 | -1.48366 |
| 49 | 6 | 0 | 4.218837 | 3.789323 | -0.08965 |
| 50 | 6 | 0 | 2.650142 | 3.77389 | -1.88439 |
| 51 | 6 | 0 | 3.657931 | 4.454209 | -1.18749 |
| 52 | 6 | 0 | 2.448635 | 0.257832 | 3.185676 |
| 53 | 6 | 0 | 0.950257 | 1.744248 | 2.249517 |
| 54 | 6 | 0 | 2.260138 | 0.808579 | 4.447626 |
| 55 | 6 | 0 | 0.728933 | 2.357282 | 3.476487 |
| 56 | 6 | 0 | 1.386239 | 1.888332 | 4.623411 |
| 57 | 7 | 0 | 2.81384 | 1.870838 | -0.42804 |
| 58 | 7 | 0 | 1.92058 | -2.08545 | 0.884266 |
| 59 | 7 | 0 | 1.79474 | 0.706122 | 2.092854 |
| 60 | 7 | 0 | -2.37114 | 2.188433 | 0.506923 |
| 61 | 7 | 0 | -2.65398 | -2.04604 | 0.403872 |
| 62 | 1 | 0 | -6.31645 | 0.164568 | -4.77811 |
| 63 | 1 | 0 | -8.2238 | 0.369916 | -3.20935 |
| 64 | 1 | 0 | -7.89098 | 0.445239 | -0.74985 |
| 65 | 1 | 0 | -0.24617 | -0.66829 | -4.3822 |
| 66 | 1 | 0 | 5.541544 | -1.77639 | -4.98117 |
| 67 | 1 | 0 | 7.614861 | -1.7688 | -3.60748 |
| 68 | 1 | 0 | 7.571959 | -1.23841 | -1.18519 |
| 69 | 1 | 0 | -1.16729 | 2.488925 | -1.14562 |
| 70 | 1 | 0 | -3.60902 | 2.106273 | 2.161495 |
| 71 | 1 | 0 | -3.66765 | 4.565725 | 2.509978 |
| 72 | 1 | 0 | -1.15715 | 4.96359 | -0.9678 |
| 73 | 1 | 0 | -3.52325 | -1.84151 | 2.266907 |
| 74 | 1 | 0 | -1.80506 | -2.46935 | -1.43853 |

| | | | | | |
|---|---|---|---|---|---|
| 75 | 1 | 0 | -3.98477 | -4.26625 | 2.554502 |
| 76 | 1 | 0 | -2.20299 | -4.90481 | -1.31592 |
| 77 | 1 | 0 | 3.409731 | -5.09703 | 1.067434 |
| 78 | 1 | 0 | -0.34422 | -3.76386 | 2.71202 |
| 79 | 1 | 0 | 0.084172 | -1.54876 | 1.714223 |
| 80 | 1 | 0 | 3.711705 | -2.82817 | 0.145158 |
| 81 | 1 | 0 | 4.230138 | 1.962565 | 1.074198 |
| 82 | 1 | 0 | 1.492401 | 1.959573 | -2.01984 |
| 83 | 1 | 0 | 5.007871 | 4.251136 | 0.495387 |
| 84 | 1 | 0 | 2.173742 | 4.224277 | -2.7497 |
| 85 | 1 | 0 | 3.160958 | -0.54032 | 3.014222 |
| 86 | 1 | 0 | 0.44434 | 2.085346 | 1.354126 |
| 87 | 1 | 0 | 2.810817 | 0.398497 | 5.288366 |
| 88 | 1 | 0 | 0.043381 | 3.197283 | 3.534869 |
| 89 | 8 | 0 | -1.48856 | -0.15996 | 2.049462 |
| 90 | 1 | 0 | -1.31538 | 0.674491 | 2.507309 |
| 91 | 6 | 0 | -3.44566 | -6.2859 | 0.738895 |
| 92 | 1 | 0 | -4.44498 | -6.49765 | 0.339804 |
| 93 | 1 | 0 | -2.73192 | -6.88457 | 0.166821 |
| 94 | 1 | 0 | -3.43353 | -6.62508 | 1.778163 |
| 95 | 6 | 0 | 1.296874 | -5.99915 | 2.609368 |
| 96 | 1 | 0 | 1.626903 | -6.79715 | 1.937938 |
| 97 | 1 | 0 | 1.883857 | -6.08927 | 3.531192 |
| 98 | 1 | 0 | 0.248819 | -6.17057 | 2.866894 |
| 99 | 6 | 0 | 1.18974 | 2.531518 | 5.969943 |
| 100 | 1 | 0 | 2.00873 | 3.229246 | 6.182284 |
| 101 | 1 | 0 | 0.254343 | 3.094641 | 6.015469 |
| 102 | 1 | 0 | 1.185585 | 1.785276 | 6.769208 |
| 103 | 6 | 0 | 4.140493 | 5.813095 | -1.61681 |
| 104 | 1 | 0 | 4.424609 | 6.4262 | -0.75729 |
| 105 | 1 | 0 | 5.02838 | 5.713686 | -2.25278 |
| 106 | 1 | 0 | 3.381501 | 6.347712 | -2.1934 |
| 107 | 6 | 0 | -2.47181 | 6.497656 | 0.92778 |
| 108 | 1 | 0 | -1.59027 | 6.973689 | 0.490658 |
| 109 | 1 | 0 | -3.35178 | 6.891693 | 0.405196 |
| 110 | 1 | 0 | -2.55163 | 6.804701 | 1.973957 |
| 111 | 1 | 0 | -3.31806 | -0.0497 | -4.63475 |

**Table S25.** Cartesian coordinates for $Ru^{III}Ru^{III}$-5pic-OH-$OH_2$-A.

| Center number | Atomic number | Atomic type | Coordinates (Ångström) | | |
|---|---|---|---|---|---|
| | | | X | Y | Z |
| 1 | 6 | 0 | 3.71206 | -0.44385 | -2.67025 |
| 2 | 6 | 0 | 3.291112 | -0.99041 | -3.91476 |
| 3 | 6 | 0 | 4.218194 | -1.26667 | -4.92662 |
| 4 | 6 | 0 | 5.560808 | -0.97847 | -4.68749 |
| 5 | 6 | 0 | 5.9705 | -0.39624 | -3.47793 |
| 6 | 6 | 0 | 5.063363 | -0.0992 | -2.45304 |
| 7 | 6 | 0 | 1.55458 | -0.73823 | -2.70338 |
| 8 | 6 | 0 | 5.541504 | 0.659597 | -1.26437 |
| 9 | 8 | 0 | 6.730212 | 0.833891 | -1.02192 |

| | | | | | |
|---|---|---|---|---|---|
| 10 | 8 | 0 | 4.591194 | 1.215402 | -0.50727 |
| 11 | 7 | 0 | 2.59077 | -0.31026 | -1.87281 |
| 12 | 7 | 0 | 1.927579 | -1.15673 | -3.90534 |
| 13 | 6 | 0 | -1.01029 | -0.86478 | -3.12888 |
| 14 | 6 | 0 | -2.08628 | -0.7665 | -2.22934 |
| 15 | 7 | 0 | -0.26092 | -0.56449 | -1.06364 |
| 16 | 7 | 0 | -1.59249 | -0.58319 | -0.9719 |
| 17 | 6 | 0 | 0.143358 | -0.73102 | -2.34329 |
| 18 | 6 | 0 | -5.38923 | -0.62017 | -1.10346 |
| 19 | 6 | 0 | -5.66487 | -0.77345 | -2.49436 |
| 20 | 6 | 0 | -7.01149 | -0.77264 | -2.89174 |
| 21 | 6 | 0 | -7.98712 | -0.60732 | -1.90463 |
| 22 | 6 | 0 | -7.67113 | -0.43608 | -0.53805 |
| 23 | 6 | 0 | -6.34605 | -0.43425 | -0.09817 |
| 24 | 6 | 0 | -3.54762 | -0.79464 | -2.24185 |
| 25 | 7 | 0 | -4.04806 | -0.65247 | -0.97736 |
| 26 | 7 | 0 | -4.47283 | -0.87932 | -3.19881 |
| 27 | 6 | 0 | -5.97316 | -0.22796 | 1.348726 |
| 28 | 8 | 0 | -6.84628 | -0.09528 | 2.198934 |
| 29 | 8 | 0 | -4.6822 | -0.19907 | 1.692314 |
| 30 | 44 | 0 | -2.92149 | -0.31801 | 0.664097 |
| 31 | 44 | 0 | 2.887536 | 0.34766 | 0.119897 |
| 32 | 6 | 0 | -3.2165 | 2.511951 | 1.509971 |
| 33 | 6 | 0 | -3.28922 | 2.37403 | -0.79688 |
| 34 | 6 | 0 | -3.52607 | 3.866298 | 1.474443 |
| 35 | 6 | 0 | -3.60242 | 3.72431 | -0.90387 |
| 36 | 6 | 0 | -3.74534 | 4.506953 | 0.248135 |
| 37 | 6 | 0 | -3.48333 | -2.78077 | 2.298713 |
| 38 | 6 | 0 | -2.32231 | -3.33172 | 0.376799 |
| 39 | 6 | 0 | -3.52593 | -4.11175 | 2.694499 |
| 40 | 6 | 0 | -2.33374 | -4.67966 | 0.712359 |
| 41 | 6 | 0 | -2.94796 | -5.10541 | 1.895773 |
| 42 | 6 | 0 | 5.182139 | -1.51653 | 0.931118 |
| 43 | 6 | 0 | 3.267611 | -2.64674 | 0.292939 |
| 44 | 6 | 0 | 5.852383 | -2.71027 | 1.164489 |
| 45 | 6 | 0 | 3.880706 | -3.87463 | 0.503354 |
| 46 | 6 | 0 | 5.2079 | -3.93328 | 0.944624 |
| 47 | 6 | 0 | 2.276648 | 2.899275 | -1.36173 |
| 48 | 6 | 0 | 0.848642 | 2.637376 | 0.437449 |
| 49 | 6 | 0 | 1.672392 | 4.100827 | -1.70972 |
| 50 | 6 | 0 | 0.202473 | 3.830713 | 0.145703 |
| 51 | 6 | 0 | 0.606151 | 4.598629 | -0.95398 |
| 52 | 6 | 0 | 4.199747 | 2.185695 | 2.192211 |
| 53 | 6 | 0 | 2.972364 | 0.505643 | 3.205914 |
| 54 | 6 | 0 | 4.568006 | 2.705841 | 3.426884 |
| 55 | 6 | 0 | 3.305358 | 0.974878 | 4.470047 |
| 56 | 6 | 0 | 4.117223 | 2.107357 | 4.609644 |
| 57 | 7 | 0 | 1.878392 | 2.169014 | -0.29812 |
| 58 | 7 | 0 | 3.905316 | -1.47684 | 0.497194 |
| 59 | 7 | 0 | 3.402937 | 1.102718 | 2.074975 |
| 60 | 7 | 0 | -3.10582 | 1.765937 | 0.390953 |
| 61 | 7 | 0 | -2.88605 | -2.38376 | 1.152932 |
| 62 | 1 | 0 | -7.2863 | -0.8904 | -3.93604 |
| 63 | 1 | 0 | -9.03343 | -0.6024 | -2.19811 |
| 64 | 1 | 0 | -8.45796 | -0.29707 | 0.196256 |

| | | | | | |
|---|---|---|---|---|---|
| 65 | 1 | 0 | -1.04658 | -1.00576 | -4.19679 |
| 66 | 1 | 0 | 3.884188 | -1.6863 | -5.87076 |
| 67 | 1 | 0 | 6.300842 | -1.18299 | -5.45582 |
| 68 | 1 | 0 | 7.009669 | -0.13054 | -3.31441 |
| 69 | 1 | 0 | -3.2007 | 1.748891 | -1.67663 |
| 70 | 1 | 0 | -3.0554 | 1.982272 | 2.440898 |
| 71 | 1 | 0 | -3.61842 | 4.410582 | 2.409565 |
| 72 | 1 | 0 | -3.75635 | 4.151722 | -1.89011 |
| 73 | 1 | 0 | -3.95883 | -1.99311 | 2.872658 |
| 74 | 1 | 0 | -1.85613 | -2.98488 | -0.53662 |
| 75 | 1 | 0 | -4.02377 | -4.3669 | 3.625087 |
| 76 | 1 | 0 | -1.86641 | -5.39361 | 0.040991 |
| 77 | 1 | 0 | 6.882235 | -2.67652 | 1.505542 |
| 78 | 1 | 0 | 3.317669 | -4.7838 | 0.316636 |
| 79 | 1 | 0 | 2.239492 | -2.57316 | -0.03564 |
| 80 | 1 | 0 | 5.674856 | -0.56125 | 1.066713 |
| 81 | 1 | 0 | 3.110649 | 2.498264 | -1.92236 |
| 82 | 1 | 0 | 0.523632 | 2.031424 | 1.274659 |
| 83 | 1 | 0 | 2.040869 | 4.64225 | -2.57535 |
| 84 | 1 | 0 | -0.62497 | 4.144175 | 0.77373 |
| 85 | 1 | 0 | 4.563343 | 2.599154 | 1.258885 |
| 86 | 1 | 0 | 2.346965 | -0.36793 | 3.062183 |
| 87 | 1 | 0 | 5.21554 | 3.576737 | 3.457591 |
| 88 | 1 | 0 | 2.930148 | 0.449778 | 5.343107 |
| 89 | 8 | 0 | 1.305808 | -0.56133 | 0.996118 |
| 90 | 1 | 0 | 0.344177 | -0.59156 | -0.19951 |
| 91 | 1 | 0 | 0.667359 | -0.01028 | 1.486246 |
| 92 | 6 | 0 | 5.917294 | -5.24698 | 1.142145 |
| 93 | 1 | 0 | 6.419834 | -5.55075 | 0.215823 |
| 94 | 1 | 0 | 6.681775 | -5.17653 | 1.92072 |
| 95 | 1 | 0 | 5.217977 | -6.04318 | 1.411719 |
| 96 | 6 | 0 | 4.472401 | 2.663361 | 5.963538 |
| 97 | 1 | 0 | 3.735742 | 3.41412 | 6.274768 |
| 98 | 1 | 0 | 4.48369 | 1.880703 | 6.726934 |
| 99 | 1 | 0 | 5.450982 | 3.150914 | 5.951165 |
| 100 | 6 | 0 | -3.01492 | -6.56202 | 2.274167 |
| 101 | 1 | 0 | -3.95094 | -7.00768 | 1.91605 |
| 102 | 1 | 0 | -2.19154 | -7.12986 | 1.832303 |
| 103 | 1 | 0 | -2.98574 | -6.6947 | 3.359438 |
| 104 | 6 | 0 | -4.15484 | 5.955445 | 0.176243 |
| 105 | 1 | 0 | -3.85116 | 6.413852 | -0.76904 |
| 106 | 1 | 0 | -5.24543 | 6.046068 | 0.247701 |
| 107 | 1 | 0 | -3.72648 | 6.535895 | 0.998614 |
| 108 | 6 | 0 | -0.09472 | 5.880453 | -1.3191 |
| 109 | 1 | 0 | -1.03545 | 5.663475 | -1.83891 |
| 110 | 1 | 0 | -0.34326 | 6.465877 | -0.42885 |
| 111 | 1 | 0 | 0.516918 | 6.498473 | -1.98118 |
| 112 | 8 | 0 | -1.47541 | 0.073734 | 2.029687 |
| 113 | 1 | 0 | -1.26977 | -0.74327 | 2.506853 |

**Table S26.** Cartesian coordinates for $Ru^{III}Ru^{III}$-5pic-2OH$_2$-A.

| | | | Coordinates (Ångström) | | |
|---|---|---|---|---|---|

| Center number | Atomic number | Atomic type | X | Y | Z |
|---|---|---|---|---|---|
| 1 | 6 | 0 | 3.621089 | -0.51026 | -2.73441 |
| 2 | 6 | 0 | 3.114614 | -0.98513 | -3.97619 |
| 3 | 6 | 0 | 3.976958 | -1.28021 | -5.04165 |
| 4 | 6 | 0 | 5.342042 | -1.09096 | -4.85227 |
| 5 | 6 | 0 | 5.842054 | -0.59531 | -3.63578 |
| 6 | 6 | 0 | 5.003791 | -0.28098 | -2.56131 |
| 7 | 6 | 0 | 1.459989 | -0.68647 | -2.66903 |
| 8 | 6 | 0 | 5.59265 | 0.345744 | -1.34895 |
| 9 | 8 | 0 | 6.795079 | 0.385041 | -1.13393 |
| 10 | 8 | 0 | 4.726873 | 0.942155 | -0.51512 |
| 11 | 7 | 0 | 2.547449 | -0.3347 | -1.88042 |
| 12 | 7 | 0 | 1.748312 | -1.07865 | -3.90547 |
| 13 | 6 | 0 | -1.0829 | -0.7857 | -3.07101 |
| 14 | 6 | 0 | -2.15736 | -0.67755 | -2.17918 |
| 15 | 7 | 0 | -0.35168 | -0.47943 | -0.99281 |
| 16 | 7 | 0 | -1.68269 | -0.49132 | -0.9097 |
| 17 | 6 | 0 | 0.061817 | -0.65045 | -2.26953 |
| 18 | 6 | 0 | -5.52773 | -0.46361 | -1.1706 |
| 19 | 6 | 0 | -5.82037 | -0.69453 | -2.52927 |
| 20 | 6 | 0 | -7.14753 | -0.7422 | -2.9605 |
| 21 | 6 | 0 | -8.12486 | -0.54629 | -1.98052 |
| 22 | 6 | 0 | -7.81334 | -0.30923 | -0.62488 |
| 23 | 6 | 0 | -6.49224 | -0.26033 | -0.18125 |
| 24 | 6 | 0 | -3.60676 | -0.67522 | -2.19859 |
| 25 | 7 | 0 | -4.17185 | -0.46433 | -1.01724 |
| 26 | 7 | 0 | -4.57339 | -0.82228 | -3.15777 |
| 27 | 6 | 0 | -6.13887 | -0.00161 | 1.272807 |
| 28 | 8 | 0 | -7.04293 | 0.156588 | 2.079155 |
| 29 | 8 | 0 | -4.86363 | 0.03884 | 1.634141 |
| 30 | 44 | 0 | -3.06646 | -0.17906 | 0.682144 |
| 31 | 44 | 0 | 2.968847 | 0.256082 | 0.119487 |
| 32 | 6 | 0 | -3.3595 | 2.651654 | 1.536546 |
| 33 | 6 | 0 | -2.90798 | 2.576314 | -0.72944 |
| 34 | 6 | 0 | -3.48413 | 4.034655 | 1.499528 |
| 35 | 6 | 0 | -3.02051 | 3.956296 | -0.83736 |
| 36 | 6 | 0 | -3.32866 | 4.726172 | 0.291946 |
| 37 | 6 | 0 | -3.81555 | -2.60597 | 2.312872 |
| 38 | 6 | 0 | -2.51786 | -3.22667 | 0.502667 |
| 39 | 6 | 0 | -3.90203 | -3.92204 | 2.744911 |
| 40 | 6 | 0 | -2.5699 | -4.56349 | 0.877466 |
| 41 | 6 | 0 | -3.27637 | -4.94757 | 2.023344 |
| 42 | 6 | 0 | 5.064738 | -1.85523 | 0.820764 |
| 43 | 6 | 0 | 3.029935 | -2.77727 | 0.219838 |
| 44 | 6 | 0 | 5.621741 | -3.11415 | 1.001048 |
| 45 | 6 | 0 | 3.525287 | -4.06347 | 0.381108 |
| 46 | 6 | 0 | 4.854925 | -4.26338 | 0.775007 |
| 47 | 6 | 0 | 2.563556 | 2.874534 | -1.33625 |
| 48 | 6 | 0 | 1.36904 | 2.841571 | 0.641036 |
| 49 | 6 | 0 | 2.139266 | 4.166648 | -1.61074 |
| 50 | 6 | 0 | 0.903777 | 4.133574 | 0.426565 |
| 51 | 6 | 0 | 1.291028 | 4.837375 | -0.71941 |
| 52 | 6 | 0 | 4.496871 | 1.869316 | 2.219765 |
| 53 | 6 | 0 | 3.156016 | 0.255489 | 3.198111 |

| | | | | | |
|---|---|---|---|---|---|
| 54 | 6 | 0 | 4.957467 | 2.285036 | 3.462971 |
| 55 | 6 | 0 | 3.576612 | 0.622953 | 4.468889 |
| 56 | 6 | 0 | 4.497586 | 1.667211 | 4.632275 |
| 57 | 7 | 0 | 2.191226 | 2.20985 | -0.21899 |
| 58 | 7 | 0 | 3.784184 | -1.67953 | 0.430249 |
| 59 | 7 | 0 | 3.597223 | 0.87217 | 2.080125 |
| 60 | 7 | 0 | -3.08202 | 1.922087 | 0.43518 |
| 61 | 7 | 0 | -3.13274 | -2.24907 | 1.199633 |
| 62 | 1 | 0 | -7.41792 | -0.91872 | -3.99623 |
| 63 | 1 | 0 | -9.16842 | -0.57552 | -2.27725 |
| 64 | 1 | 0 | -8.60285 | -0.15778 | 0.103164 |
| 65 | 1 | 0 | -1.0866 | -0.93671 | -4.13872 |
| 66 | 1 | 0 | 3.576505 | -1.6424 | -5.98341 |
| 67 | 1 | 0 | 6.034457 | -1.31184 | -5.65871 |
| 68 | 1 | 0 | 6.905036 | -0.42128 | -3.50626 |
| 69 | 1 | 0 | -2.67047 | 1.968037 | -1.59388 |
| 70 | 1 | 0 | -3.49161 | 2.091113 | 2.453143 |
| 71 | 1 | 0 | -3.71906 | 4.565008 | 2.417076 |
| 72 | 1 | 0 | -2.87567 | 4.424191 | -1.80616 |
| 73 | 1 | 0 | -4.31319 | -1.79795 | 2.837053 |
| 74 | 1 | 0 | -1.96698 | -2.91504 | -0.37602 |
| 75 | 1 | 0 | -4.46789 | -4.14111 | 3.645112 |
| 76 | 1 | 0 | -2.05646 | -5.30191 | 0.269702 |
| 77 | 1 | 0 | 6.659909 | -3.18904 | 1.308002 |
| 78 | 1 | 0 | 2.870779 | -4.90878 | 0.192981 |
| 79 | 1 | 0 | 2.005081 | -2.59724 | -0.07645 |
| 80 | 1 | 0 | 5.652911 | -0.95747 | 0.966865 |
| 81 | 1 | 0 | 3.228322 | 2.342916 | -2.0042 |
| 82 | 1 | 0 | 1.079734 | 2.295917 | 1.531228 |
| 83 | 1 | 0 | 2.479114 | 4.647376 | -2.52278 |
| 84 | 1 | 0 | 0.241942 | 4.582658 | 1.160038 |
| 85 | 1 | 0 | 4.866323 | 2.299555 | 1.296506 |
| 86 | 1 | 0 | 2.452821 | -0.55492 | 3.042436 |
| 87 | 1 | 0 | 5.686278 | 3.088027 | 3.510277 |
| 88 | 1 | 0 | 3.189423 | 0.085506 | 5.328989 |
| 89 | 8 | 0 | 1.241612 | -0.44341 | 0.958567 |
| 90 | 1 | 0 | 0.288544 | -0.46288 | -0.10147 |
| 91 | 1 | 0 | 0.790188 | 0.164751 | 1.564563 |
| 92 | 6 | 0 | 5.438367 | -5.64326 | 0.918448 |
| 93 | 1 | 0 | 5.855155 | -5.9792 | -0.03876 |
| 94 | 1 | 0 | 6.24854 | -5.6612 | 1.651887 |
| 95 | 1 | 0 | 4.679543 | -6.37088 | 1.218533 |
| 96 | 6 | 0 | 4.956427 | 2.108363 | 5.996239 |
| 97 | 1 | 0 | 4.254059 | 2.837578 | 6.418142 |
| 98 | 1 | 0 | 5.00796 | 1.266266 | 6.691732 |
| 99 | 1 | 0 | 5.938821 | 2.584988 | 5.953565 |
| 100 | 6 | 0 | -3.37654 | -6.38595 | 2.455426 |
| 101 | 1 | 0 | -4.39731 | -6.75889 | 2.313084 |
| 102 | 1 | 0 | -2.70202 | -7.02725 | 1.883588 |
| 103 | 1 | 0 | -3.14025 | -6.49548 | 3.518533 |
| 104 | 6 | 0 | -3.51682 | 6.217863 | 0.206111 |
| 105 | 1 | 0 | -2.95465 | 6.648208 | -0.6271 |
| 106 | 1 | 0 | -4.5746 | 6.45823 | 0.045125 |
| 107 | 1 | 0 | -3.21013 | 6.715121 | 1.130479 |
| 108 | 6 | 0 | 0.844045 | 6.251528 | -0.97857 |

| | | | | | |
|---|---|---|---|---|---|
| 109 | 1 | 0 | 0.563579 | 6.39332 | -2.02687 |
| 110 | 1 | 0 | -0.00481 | 6.527703 | -0.34875 |
| 111 | 1 | 0 | 1.65814 | 6.955046 | -0.76769 |
| 112 | 8 | 0 | -1.74675 | 0.134455 | 2.1139 |
| 113 | 1 | 0 | -1.57051 | -0.68774 | 2.595544 |
| 114 | 1 | 0 | -4.41056 | -1.00915 | -4.1356 |

**Table S27.** Cartesian coordinates for $Ru^{III}Ru^{III}$-4pic-OH-$OH_2$-A.

| Center number | Atomic number | Atomic type | Coordinates (Ångström) | | |
|---|---|---|---|---|---|
| | | | X | Y | Z |
| 1 | 6 | 0 | -4.48204 | -0.7231 | 1.432058 |
| 2 | 6 | 0 | -4.59936 | -0.80437 | 2.843311 |
| 3 | 6 | 0 | -5.87507 | -0.97638 | 3.382493 |
| 4 | 6 | 0 | -6.95626 | -1.04564 | 2.488342 |
| 5 | 6 | 0 | -6.80079 | -0.94045 | 1.094052 |
| 6 | 6 | 0 | -5.53532 | -0.76751 | 0.516956 |
| 7 | 6 | 0 | -2.51884 | -0.51618 | 2.406271 |
| 8 | 6 | 0 | -5.34201 | -0.62586 | -0.98072 |
| 9 | 8 | 0 | -6.31945 | -0.72794 | -1.71663 |
| 10 | 8 | 0 | -4.1281 | -0.39832 | -1.47227 |
| 11 | 7 | 0 | -3.15943 | -0.55735 | 1.17626 |
| 12 | 7 | 0 | -3.33166 | -0.66502 | 3.435511 |
| 13 | 6 | 0 | -0.00557 | -0.05506 | 3.104257 |
| 14 | 6 | 0 | 1.07409 | 0.167832 | 2.245944 |
| 15 | 7 | 0 | -0.67772 | -0.16402 | 0.938974 |
| 16 | 7 | 0 | 0.64396 | 0.074724 | 0.939037 |
| 17 | 6 | 0 | -1.09327 | -0.26844 | 2.251567 |
| 18 | 6 | 0 | 4.41809 | 0.709224 | 1.393712 |
| 19 | 6 | 0 | 4.572109 | 0.698027 | 2.80993 |
| 20 | 6 | 0 | 5.870768 | 0.834188 | 3.321691 |
| 21 | 6 | 0 | 6.927635 | 0.945711 | 2.411362 |
| 22 | 6 | 0 | 6.737013 | 0.919377 | 1.013376 |
| 23 | 6 | 0 | 5.458473 | 0.79524 | 0.462622 |
| 24 | 6 | 0 | 2.49635 | 0.449713 | 2.379035 |
| 25 | 7 | 0 | 3.094674 | 0.585312 | 1.156569 |
| 26 | 7 | 0 | 3.331928 | 0.521461 | 3.414094 |
| 27 | 6 | 0 | 5.216366 | 0.723228 | -1.02133 |
| 28 | 8 | 0 | 6.154346 | 0.735967 | -1.80886 |
| 29 | 8 | 0 | 3.954696 | 0.668735 | -1.46766 |
| 30 | 44 | 0 | 2.183122 | 0.225837 | -0.57951 |
| 31 | 44 | 0 | -2.2804 | -0.23688 | -0.48621 |
| 32 | 6 | 0 | 1.707707 | 2.700743 | -2.1793 |
| 33 | 6 | 0 | 1.494817 | 3.136392 | 0.082095 |
| 34 | 6 | 0 | 1.518891 | 4.040269 | -2.49141 |
| 35 | 6 | 0 | 1.305009 | 4.491436 | -0.1604 |
| 36 | 6 | 0 | 1.317726 | 4.979104 | -1.47218 |
| 37 | 6 | 0 | 3.615931 | -2.289 | -1.41497 |
| 38 | 6 | 0 | 2.652161 | -2.56147 | 0.668181 |
| 39 | 6 | 0 | 4.158069 | -3.56706 | -1.37123 |
| 40 | 6 | 0 | 3.169103 | -3.84682 | 0.778924 |

| | | | | | |
|---|---|---|---|---|---|
| 41 | 6 | 0 | 3.947971 | -4.38382 | -0.25338 |
| 42 | 6 | 0 | -2.76026 | -2.95734 | -1.74779 |
| 43 | 6 | 0 | -0.95346 | -3.01724 | -0.30231 |
| 44 | 6 | 0 | -2.59361 | -4.29931 | -2.06412 |
| 45 | 6 | 0 | -0.73387 | -4.36126 | -0.57448 |
| 46 | 6 | 0 | -1.56559 | -5.04265 | -1.47155 |
| 47 | 6 | 0 | -3.35798 | 2.438174 | -1.30241 |
| 48 | 6 | 0 | -2.26932 | 2.606862 | 0.732656 |
| 49 | 6 | 0 | -3.68937 | 3.786336 | -1.27537 |
| 50 | 6 | 0 | -2.57591 | 3.957668 | 0.825474 |
| 51 | 6 | 0 | -3.30716 | 4.584418 | -0.19099 |
| 52 | 7 | 0 | -2.65318 | 1.848903 | -0.31367 |
| 53 | 7 | 0 | -1.95182 | -2.31507 | -0.87533 |
| 54 | 7 | 0 | 1.691519 | 2.247384 | -0.90948 |
| 55 | 7 | 0 | 2.862989 | -1.78393 | -0.41279 |
| 56 | 1 | 0 | 6.051702 | 0.842924 | 4.392611 |
| 57 | 1 | 0 | 7.939543 | 1.045902 | 2.794258 |
| 58 | 1 | 0 | 7.584542 | 0.987315 | 0.339165 |
| 59 | 1 | 0 | -0.00061 | -0.05648 | 4.183086 |
| 60 | 1 | 0 | -6.02876 | -1.04723 | 4.45487 |
| 61 | 1 | 0 | -7.95679 | -1.1781 | 2.890407 |
| 62 | 1 | 0 | -7.66132 | -0.98621 | 0.435079 |
| 63 | 1 | 0 | 1.506943 | 2.738534 | 1.089043 |
| 64 | 1 | 0 | 1.87915 | 1.949999 | -2.94081 |
| 65 | 1 | 0 | 1.542011 | 4.346907 | -3.53261 |
| 66 | 1 | 0 | 1.15919 | 5.162648 | 0.680354 |
| 67 | 1 | 0 | 3.809314 | -1.61628 | -2.24346 |
| 68 | 1 | 0 | 2.061975 | -2.12541 | 1.464884 |
| 69 | 1 | 0 | 4.761146 | -3.911 | -2.2058 |
| 70 | 1 | 0 | 2.973168 | -4.41725 | 1.681814 |
| 71 | 1 | 0 | -3.27631 | -4.76027 | -2.77125 |
| 72 | 1 | 0 | 0.091532 | -4.86827 | -0.08469 |
| 73 | 1 | 0 | -0.32164 | -2.46694 | 0.381389 |
| 74 | 1 | 0 | -3.55536 | -2.35435 | -2.17336 |
| 75 | 1 | 0 | -3.67677 | 1.785438 | -2.10521 |
| 76 | 1 | 0 | -1.70671 | 2.105645 | 1.509758 |
| 77 | 1 | 0 | -4.26167 | 4.203306 | -2.09812 |
| 78 | 1 | 0 | -2.24515 | 4.513689 | 1.697132 |
| 79 | 8 | 0 | 1.096111 | -0.27962 | -2.25739 |
| 80 | 6 | 0 | 1.158847 | 6.4466 | -1.77414 |
| 81 | 1 | 0 | 0.658807 | 6.973113 | -0.95685 |
| 82 | 1 | 0 | 2.139328 | 6.916338 | -1.91778 |
| 83 | 1 | 0 | 0.585529 | 6.60532 | -2.69227 |
| 84 | 6 | 0 | 4.564744 | -5.75406 | -0.151 |
| 85 | 1 | 0 | 5.591605 | -5.68093 | 0.226922 |
| 86 | 1 | 0 | 4.007392 | -6.39606 | 0.536312 |
| 87 | 1 | 0 | 4.612032 | -6.24534 | -1.12708 |
| 88 | 6 | 0 | -1.38238 | -6.50806 | -1.76463 |
| 89 | 1 | 0 | -2.02549 | -7.10967 | -1.11087 |
| 90 | 1 | 0 | -1.65422 | -6.74587 | -2.79675 |
| 91 | 1 | 0 | -0.35041 | -6.8258 | -1.5937 |
| 92 | 6 | 0 | -3.6945 | 6.037099 | -0.10498 |
| 93 | 1 | 0 | -4.67165 | 6.141673 | 0.382012 |
| 94 | 1 | 0 | -2.97344 | 6.610887 | 0.483563 |
| 95 | 1 | 0 | -3.7743 | 6.489357 | -1.0973 |

| | | | | | |
|---|---|---|---|---|---|
| 96 | 1 | 0 | 1.319931 | -1.16414 | -2.57429 |
| 97 | 8 | 0 | -1.3195 | 0.22557 | -2.34312 |
| 98 | 1 | 0 | -1.77131 | -0.13421 | -3.11701 |
| 99 | 1 | 0 | -0.28615 | -0.00477 | -2.36233 |

**Table S28.** Cartesian coordinates for $Ru^{III}Ru^{III}$-4pic-$2OH_2$-A.

| Center number | Atomic number | Atomic type | Coordinates (Ångström) | | |
|---|---|---|---|---|---|
| | | | X | Y | Z |
| 1 | 6 | 0 | 4.454974 | 0.632538 | 1.450883 |
| 2 | 6 | 0 | 4.568782 | 0.72107 | 2.860278 |
| 3 | 6 | 0 | 5.845884 | 0.866423 | 3.405502 |
| 4 | 6 | 0 | 6.929582 | 0.902623 | 2.513435 |
| 5 | 6 | 0 | 6.776439 | 0.789897 | 1.118304 |
| 6 | 6 | 0 | 5.509921 | 0.643164 | 0.53787 |
| 7 | 6 | 0 | 2.48535 | 0.481657 | 2.413621 |
| 8 | 6 | 0 | 5.314608 | 0.491401 | -0.95836 |
| 9 | 8 | 0 | 6.288495 | 0.566847 | -1.69575 |
| 10 | 8 | 0 | 4.093872 | 0.276381 | -1.45122 |
| 11 | 7 | 0 | 3.129406 | 0.497636 | 1.187535 |
| 12 | 7 | 0 | 3.293373 | 0.615746 | 3.446443 |
| 13 | 6 | 0 | -0.02919 | 0.061465 | 3.116586 |
| 14 | 6 | 0 | -1.11358 | -0.13698 | 2.255274 |
| 15 | 7 | 0 | 0.630191 | 0.173753 | 0.946106 |
| 16 | 7 | 0 | -0.68873 | -0.04058 | 0.94471 |
| 17 | 6 | 0 | 1.054357 | 0.263606 | 2.258317 |
| 18 | 6 | 0 | -4.48562 | -0.73031 | 1.422656 |
| 19 | 6 | 0 | -4.6833 | -0.80258 | 2.815039 |
| 20 | 6 | 0 | -5.96167 | -1.02084 | 3.330019 |
| 21 | 6 | 0 | -6.99619 | -1.14124 | 2.39632 |
| 22 | 6 | 0 | -6.78503 | -1.04144 | 1.006047 |
| 23 | 6 | 0 | -5.51005 | -0.83016 | 0.479747 |
| 24 | 6 | 0 | -2.52343 | -0.42459 | 2.352631 |
| 25 | 7 | 0 | -3.15648 | -0.51904 | 1.189144 |
| 26 | 7 | 0 | -3.41377 | -0.6015 | 3.378111 |
| 27 | 6 | 0 | -5.26496 | -0.68065 | -1.00681 |
| 28 | 8 | 0 | -6.21119 | -0.69594 | -1.77659 |
| 29 | 8 | 0 | -4.01303 | -0.55465 | -1.44193 |
| 30 | 44 | 0 | -2.22633 | -0.14233 | -0.5744 |
| 31 | 44 | 0 | 2.259678 | 0.190971 | -0.47749 |
| 32 | 6 | 0 | -1.98198 | -2.58873 | -2.25099 |
| 33 | 6 | 0 | -1.33005 | -3.05643 | -0.08167 |
| 34 | 6 | 0 | -1.78455 | -3.90877 | -2.63095 |
| 35 | 6 | 0 | -1.11667 | -4.39263 | -0.39425 |
| 36 | 6 | 0 | -1.34845 | -4.8557 | -1.69568 |
| 37 | 6 | 0 | -3.65456 | 2.397808 | -1.36474 |
| 38 | 6 | 0 | -2.52037 | 2.708962 | 0.625199 |
| 39 | 6 | 0 | -4.13906 | 3.698428 | -1.33217 |
| 40 | 6 | 0 | -2.97629 | 4.017589 | 0.722978 |
| 41 | 6 | 0 | -3.81202 | 4.549164 | -0.2678 |
| 42 | 6 | 0 | 2.795739 | 2.825222 | -1.87243 |

| | | | | | |
|---|---|---|---|---|---|
| 43 | 6 | 0 | 1.2169 | 3.089419 | -0.20108 |
| 44 | 6 | 0 | 2.730095 | 4.174474 | -2.19278 |
| 45 | 6 | 0 | 1.109633 | 4.447721 | -0.46581 |
| 46 | 6 | 0 | 1.878795 | 5.030264 | -1.48212 |
| 47 | 6 | 0 | 3.220533 | -2.51872 | -1.32632 |
| 48 | 6 | 0 | 2.351645 | -2.63226 | 0.813438 |
| 49 | 6 | 0 | 3.584901 | -3.85751 | -1.2861 |
| 50 | 6 | 0 | 2.700434 | -3.97098 | 0.924357 |
| 51 | 6 | 0 | 3.340558 | -4.62195 | -0.13874 |
| 52 | 7 | 0 | 2.605171 | -1.90442 | -0.29284 |
| 53 | 7 | 0 | 2.045277 | 2.277646 | -0.88889 |
| 54 | 7 | 0 | -1.75774 | -2.15962 | -0.99034 |
| 55 | 7 | 0 | -2.84958 | 1.895777 | -0.39922 |
| 56 | 1 | 0 | -6.15651 | -1.08918 | 4.395091 |
| 57 | 1 | 0 | -8.00475 | -1.30974 | 2.759678 |
| 58 | 1 | 0 | -7.61735 | -1.1222 | 0.315347 |
| 59 | 1 | 0 | -0.0095 | 0.053069 | 4.195957 |
| 60 | 1 | 0 | 5.998724 | 0.942173 | 4.477235 |
| 61 | 1 | 0 | 7.931528 | 1.014155 | 2.916677 |
| 62 | 1 | 0 | 7.641585 | 0.809448 | 0.46423 |
| 63 | 1 | 0 | -1.15777 | -2.67981 | 0.919048 |
| 64 | 1 | 0 | -2.33383 | -1.84063 | -2.94958 |
| 65 | 1 | 0 | -1.98291 | -4.1945 | -3.65909 |
| 66 | 1 | 0 | -0.77329 | -5.06895 | 0.381837 |
| 67 | 1 | 0 | -3.92936 | 1.710772 | -2.1575 |
| 68 | 1 | 0 | -1.87269 | 2.287011 | 1.384477 |
| 69 | 1 | 0 | -4.78425 | 4.037331 | -2.13659 |
| 70 | 1 | 0 | -2.68087 | 4.617169 | 1.578545 |
| 71 | 1 | 0 | 3.359815 | 4.554878 | -2.99088 |
| 72 | 1 | 0 | 0.427334 | 5.049396 | 0.12612 |
| 73 | 1 | 0 | 0.632685 | 2.618091 | 0.578098 |
| 74 | 1 | 0 | 3.477576 | 2.145916 | -2.37269 |
| 75 | 1 | 0 | 3.446985 | -1.89484 | -2.18051 |
| 76 | 1 | 0 | 1.866379 | -2.11553 | 1.631747 |
| 77 | 1 | 0 | 4.082501 | -4.29155 | -2.14748 |
| 78 | 1 | 0 | 2.483421 | -4.4983 | 1.848122 |
| 79 | 8 | 0 | -1.15975 | 0.348435 | -2.20858 |
| 80 | 6 | 0 | -1.16751 | -6.30286 | -2.06847 |
| 81 | 1 | 0 | -0.5319 | -6.82799 | -1.35132 |
| 82 | 1 | 0 | -2.13712 | -6.81446 | -2.08881 |
| 83 | 1 | 0 | -0.72777 | -6.40478 | -3.06468 |
| 84 | 6 | 0 | -4.35633 | 5.949498 | -0.18408 |
| 85 | 1 | 0 | -5.39545 | 5.93203 | 0.165406 |
| 86 | 1 | 0 | -3.78277 | 6.565268 | 0.512702 |
| 87 | 1 | 0 | -4.35427 | 6.43583 | -1.16376 |
| 88 | 6 | 0 | 1.821224 | 6.504778 | -1.7757 |
| 89 | 1 | 0 | 2.654166 | 7.020567 | -1.28301 |
| 90 | 1 | 0 | 1.9081 | 6.701334 | -2.84784 |
| 91 | 1 | 0 | 0.893551 | 6.953277 | -1.41172 |
| 92 | 6 | 0 | 3.780458 | -6.0576 | -0.03931 |
| 93 | 1 | 0 | 4.826876 | -6.10937 | 0.284699 |
| 94 | 1 | 0 | 3.184396 | -6.61323 | 0.6892 |
| 95 | 1 | 0 | 3.715634 | -6.56418 | -1.0061 |
| 96 | 1 | 0 | -1.34052 | 1.256999 | -2.48655 |
| 97 | 8 | 0 | 1.288711 | -0.26137 | -2.34578 |

| | | | | | |
|---|---|---|---|---|---|
| 98 | 1 | 0 | 1.736101 | 0.063389 | -3.13811 |
| 99 | 1 | 0 | 0.292276 | -0.0138 | -2.36598 |
| 100 | 1 | 0 | -3.19055 | -0.58626 | 4.36173 |

**Table S29.** Cartesian coordinates for $Ru^{III}Ru^{III}$-4pic-$H^{+}$-2$OH_2$-A.

| Center number | Atomic number | Atomic type | Coordinates (Ångström) | | |
|---|---|---|---|---|---|
| | | | X | Y | Z |
| 1 | 6 | 0 | -4.53173 | -0.38127 | 1.461847 |
| 2 | 6 | 0 | -4.7241 | -0.51796 | 2.848349 |
| 3 | 6 | 0 | -6.01699 | -0.64345 | 3.361194 |
| 4 | 6 | 0 | -7.05793 | -0.6227 | 2.427552 |
| 5 | 6 | 0 | -6.84516 | -0.4804 | 1.039008 |
| 6 | 6 | 0 | -5.55875 | -0.34703 | 0.515695 |
| 7 | 6 | 0 | -2.5374 | -0.33675 | 2.385165 |
| 8 | 6 | 0 | -5.29544 | -0.19357 | -0.96428 |
| 9 | 8 | 0 | -6.19107 | -0.36848 | -1.76649 |
| 10 | 8 | 0 | -4.06695 | 0.175318 | -1.38322 |
| 11 | 7 | 0 | -3.19259 | -0.28118 | 1.22891 |
| 12 | 7 | 0 | -3.43434 | -0.48443 | 3.40675 |
| 13 | 6 | 0 | -0.00682 | -0.10748 | 3.118954 |
| 14 | 6 | 0 | 1.083992 | 0.036605 | 2.255057 |
| 15 | 7 | 0 | -0.67547 | -0.13205 | 0.94226 |
| 16 | 7 | 0 | 0.654135 | 0.002815 | 0.945211 |
| 17 | 6 | 0 | -1.1027 | -0.2177 | 2.260238 |
| 18 | 6 | 0 | 4.503881 | 0.359998 | 1.467652 |
| 19 | 6 | 0 | 4.694664 | 0.368256 | 2.862153 |
| 20 | 6 | 0 | 5.984751 | 0.451648 | 3.392308 |
| 21 | 6 | 0 | 7.030715 | 0.504766 | 2.46784 |
| 22 | 6 | 0 | 6.823921 | 0.469273 | 1.070911 |
| 23 | 6 | 0 | 5.540831 | 0.397016 | 0.532116 |
| 24 | 6 | 0 | 2.516147 | 0.202254 | 2.375249 |
| 25 | 7 | 0 | 3.163791 | 0.274021 | 1.219035 |
| 26 | 7 | 0 | 3.408257 | 0.264061 | 3.411412 |
| 27 | 6 | 0 | 5.289938 | 0.318811 | -0.95615 |
| 28 | 8 | 0 | 6.222821 | 0.211116 | -1.72841 |
| 29 | 8 | 0 | 4.028334 | 0.408759 | -1.39907 |
| 30 | 44 | 0 | 2.239981 | 0.054469 | -0.56361 |
| 31 | 44 | 0 | -2.27646 | -0.03472 | -0.50414 |
| 32 | 6 | 0 | 2.151115 | 2.57511 | -2.15109 |
| 33 | 6 | 0 | 1.67008 | 3.035687 | 0.063722 |
| 34 | 6 | 0 | 2.122771 | 3.924507 | -2.47313 |
| 35 | 6 | 0 | 1.631645 | 4.400169 | -0.18875 |
| 36 | 6 | 0 | 1.870859 | 4.883406 | -1.48285 |
| 37 | 6 | 0 | 3.537135 | -2.5231 | -1.47668 |
| 38 | 6 | 0 | 2.333238 | -2.88053 | 0.464097 |
| 39 | 6 | 0 | 3.938605 | -3.85101 | -1.51309 |
| 40 | 6 | 0 | 2.703719 | -4.21859 | 0.49167 |
| 41 | 6 | 0 | 3.532596 | -4.74265 | -0.51007 |
| 42 | 6 | 0 | -3.09854 | -2.59467 | -1.90708 |
| 43 | 6 | 0 | -1.71661 | -3.049 | -0.10559 |

| | | | | | |
|---|---|---|---|---|---|
| 44 | 6 | 0 | -3.20161 | -3.94275 | -2.2121 |
| 45 | 6 | 0 | -1.77844 | -4.41266 | -0.35779 |
| 46 | 6 | 0 | -2.53693 | -4.90029 | -1.43088 |
| 47 | 6 | 0 | -2.72442 | 2.772648 | -1.45931 |
| 48 | 6 | 0 | -2.10174 | 2.804092 | 0.767754 |
| 49 | 6 | 0 | -2.91668 | 4.146007 | -1.45952 |
| 50 | 6 | 0 | -2.27985 | 4.178163 | 0.837676 |
| 51 | 6 | 0 | -2.70772 | 4.891578 | -0.29108 |
| 52 | 7 | 0 | -2.32198 | 2.096809 | -0.35956 |
| 53 | 7 | 0 | -2.36351 | -2.13833 | -0.86335 |
| 54 | 7 | 0 | 1.927314 | 2.126418 | -0.89645 |
| 55 | 7 | 0 | 2.740294 | -2.02838 | -0.49895 |
| 56 | 1 | 0 | 6.177868 | 0.468585 | 4.459496 |
| 57 | 1 | 0 | 8.04785 | 0.567328 | 2.840323 |
| 58 | 1 | 0 | 7.668937 | 0.493345 | 0.391252 |
| 59 | 1 | 0 | -0.00334 | -0.12112 | 4.198829 |
| 60 | 1 | 0 | -6.21822 | -0.75038 | 4.421616 |
| 61 | 1 | 0 | -8.07665 | -0.71848 | 2.788291 |
| 62 | 1 | 0 | -7.68729 | -0.47056 | 0.355457 |
| 63 | 1 | 0 | 1.499934 | 2.648748 | 1.061324 |
| 64 | 1 | 0 | 2.366536 | 1.818129 | -2.89396 |
| 65 | 1 | 0 | 2.31585 | 4.223157 | -3.49851 |
| 66 | 1 | 0 | 1.42951 | 5.084539 | 0.628968 |
| 67 | 1 | 0 | 3.87596 | -1.81236 | -2.22236 |
| 68 | 1 | 0 | 1.693385 | -2.46459 | 1.233562 |
| 69 | 1 | 0 | 4.582957 | -4.18048 | -2.32202 |
| 70 | 1 | 0 | 2.350577 | -4.84935 | 1.301582 |
| 71 | 1 | 0 | -3.81359 | -4.24244 | -3.05689 |
| 72 | 1 | 0 | -1.23608 | -5.09431 | 0.289321 |
| 73 | 1 | 0 | -1.13301 | -2.65849 | 0.718623 |
| 74 | 1 | 0 | -3.63494 | -1.84642 | -2.47918 |
| 75 | 1 | 0 | -2.90803 | 2.179536 | -2.34403 |
| 76 | 1 | 0 | -1.77586 | 2.245929 | 1.636295 |
| 77 | 1 | 0 | -3.24691 | 4.62798 | -2.37409 |
| 78 | 1 | 0 | -2.09448 | 4.688269 | 1.777615 |
| 79 | 8 | 0 | 1.136079 | -0.31719 | -2.21446 |
| 80 | 6 | 0 | 1.890627 | 6.355667 | -1.79073 |
| 81 | 1 | 0 | 1.360294 | 6.935934 | -1.0319 |
| 82 | 1 | 0 | 2.924284 | 6.720251 | -1.82222 |
| 83 | 1 | 0 | 1.446734 | 6.564993 | -2.76792 |
| 84 | 6 | 0 | 3.987962 | -6.17555 | -0.50131 |
| 85 | 1 | 0 | 5.029175 | -6.23856 | -0.16356 |
| 86 | 1 | 0 | 3.383204 | -6.78993 | 0.16935 |
| 87 | 1 | 0 | 3.950337 | -6.60985 | -1.50445 |
| 88 | 6 | 0 | -2.65711 | -6.36957 | -1.72217 |
| 89 | 1 | 0 | -3.6486 | -6.7332 | -1.42734 |
| 90 | 1 | 0 | -2.54854 | -6.56991 | -2.79213 |
| 91 | 1 | 0 | -1.91137 | -6.95374 | -1.17902 |
| 92 | 6 | 0 | -2.96032 | 6.372785 | -0.24672 |
| 93 | 1 | 0 | -4.03313 | 6.567343 | -0.1287 |
| 94 | 1 | 0 | -2.44537 | 6.84756 | 0.591626 |
| 95 | 1 | 0 | -2.64694 | 6.858727 | -1.17476 |
| 96 | 1 | 0 | 1.368751 | -1.15741 | -2.63347 |
| 97 | 8 | 0 | -1.26632 | 0.242401 | -2.35005 |
| 98 | 1 | 0 | -1.68028 | -0.17049 | -3.12012 |

| | | | | | |
|---|---|---|---|---|---|
| 99 | 1 | 0 | -0.23538 | 0.009456 | -2.33636 |
| 100 | 1 | 0 | 3.183312 | 0.236097 | 4.394948 |
| 101 | 1 | 0 | -3.21037 | -0.57533 | 4.38704 |

**Table S30.** Cartesian coordinates for $Ru^{III}Ru^{III}$-4pic-$2H^{+}$-$2OH_2$.

| Center number | Atomic number | Atomic type | Coordinates (Ångström) | | |
|---|---|---|---|---|---|
| | | | X | Y | Z |
| 1 | 6 | 0 | -4.54557 | 0.46854 | 1.415416 |
| 2 | 6 | 0 | -4.73982 | 0.507199 | 2.808599 |
| 3 | 6 | 0 | -6.03395 | 0.616432 | 3.326545 |
| 4 | 6 | 0 | -7.07294 | 0.669003 | 2.393201 |
| 5 | 6 | 0 | -6.86081 | 0.612936 | 0.996672 |
| 6 | 6 | 0 | -5.57332 | 0.516525 | 0.466917 |
| 7 | 6 | 0 | -2.55621 | 0.314983 | 2.343015 |
| 8 | 6 | 0 | -5.30916 | 0.425837 | -1.01102 |
| 9 | 8 | 0 | -6.20341 | 0.255855 | -1.80725 |
| 10 | 8 | 0 | -4.02648 | 0.566292 | -1.47167 |
| 11 | 7 | 0 | -3.20801 | 0.357603 | 1.18442 |
| 12 | 7 | 0 | -3.45506 | 0.405805 | 3.366283 |
| 13 | 6 | 0 | -0.03961 | -0.00168 | 3.071997 |
| 14 | 6 | 0 | 1.057368 | -0.13501 | 2.217355 |
| 15 | 7 | 0 | -0.69823 | 0.092987 | 0.891555 |
| 16 | 7 | 0 | 0.638956 | -0.0543 | 0.896015 |
| 17 | 6 | 0 | -1.12583 | 0.146242 | 2.208232 |
| 18 | 6 | 0 | 4.468644 | -0.55577 | 1.434218 |
| 19 | 6 | 0 | 4.657034 | -0.62726 | 2.827473 |
| 20 | 6 | 0 | 5.943889 | -0.7993 | 3.346424 |
| 21 | 6 | 0 | 6.979288 | -0.88275 | 2.412159 |
| 22 | 6 | 0 | 6.771345 | -0.7972 | 1.015557 |
| 23 | 6 | 0 | 5.493282 | -0.62801 | 0.483798 |
| 24 | 6 | 0 | 2.483199 | -0.34109 | 2.36067 |
| 25 | 7 | 0 | 3.138744 | -0.38993 | 1.203555 |
| 26 | 7 | 0 | 3.375326 | -0.48584 | 3.385572 |
| 27 | 6 | 0 | 5.253452 | -0.51896 | -1.00161 |
| 28 | 8 | 0 | 6.175791 | -0.57343 | -1.78391 |
| 29 | 8 | 0 | 3.986071 | -0.36085 | -1.48388 |
| 30 | 44 | 0 | 2.261469 | -0.16028 | -0.52815 |
| 31 | 44 | 0 | -2.31645 | 0.222638 | -0.55538 |
| 32 | 6 | 0 | 3.336228 | 2.504148 | -1.47848 |
| 33 | 6 | 0 | 2.427547 | 2.719888 | 0.641014 |
| 34 | 6 | 0 | 3.765708 | 3.822116 | -1.46579 |
| 35 | 6 | 0 | 2.83158 | 4.043759 | 0.718713 |
| 36 | 6 | 0 | 3.534256 | 4.634858 | -0.34421 |
| 37 | 6 | 0 | 2.601004 | -2.87418 | -1.85384 |
| 38 | 6 | 0 | 1.46442 | -3.08989 | 0.149958 |
| 39 | 6 | 0 | 2.599307 | -4.24656 | -2.04392 |
| 40 | 6 | 0 | 1.433729 | -4.47087 | 0.023278 |
| 41 | 6 | 0 | 2.015142 | -5.0959 | -1.09069 |
| 42 | 6 | 0 | -3.55052 | -2.37813 | -1.56642 |
| 43 | 6 | 0 | -2.37218 | -2.73028 | 0.395053 |

| | | | | | |
|---|---|---|---|---|---|
| 44 | 6 | 0 | -3.94905 | -3.70393 | -1.60836 |
| 45 | 6 | 0 | -2.74116 | -4.0679 | 0.414605 |
| 46 | 6 | 0 | -3.55569 | -4.59714 | -0.59766 |
| 47 | 6 | 0 | -2.96626 | 3.209103 | -0.42573 |
| 48 | 6 | 0 | -0.81735 | 2.828337 | -1.19886 |
| 49 | 6 | 0 | -2.84266 | 4.57184 | -0.64877 |
| 50 | 6 | 0 | -0.63377 | 4.179859 | -1.45441 |
| 51 | 6 | 0 | -1.66095 | 5.099572 | -1.19085 |
| 52 | 7 | 0 | -1.97293 | 2.331224 | -0.70097 |
| 53 | 7 | 0 | -2.7631 | -1.87721 | -0.57824 |
| 54 | 7 | 0 | 2.671996 | 1.94172 | -0.43861 |
| 55 | 7 | 0 | 2.036493 | -2.28258 | -0.77107 |
| 56 | 1 | 0 | 6.143617 | -0.86333 | 4.41054 |
| 57 | 1 | 0 | 7.992921 | -1.01538 | 2.775485 |
| 58 | 1 | 0 | 7.614635 | -0.86038 | 0.335966 |
| 59 | 1 | 0 | -0.04563 | -0.01089 | 4.152118 |
| 60 | 1 | 0 | -6.2369 | 0.654593 | 4.391375 |
| 61 | 1 | 0 | -8.09135 | 0.750569 | 2.758087 |
| 62 | 1 | 0 | -7.7064 | 0.643204 | 0.31753 |
| 63 | 1 | 0 | 1.889464 | 2.259075 | 1.459429 |
| 64 | 1 | 0 | 3.556556 | 1.855596 | -2.31611 |
| 65 | 1 | 0 | 4.305097 | 4.204667 | -2.32651 |
| 66 | 1 | 0 | 2.608325 | 4.611412 | 1.616396 |
| 67 | 1 | 0 | 3.111672 | -2.21859 | -2.54786 |
| 68 | 1 | 0 | 1.028835 | -2.60296 | 1.012884 |
| 69 | 1 | 0 | 3.07972 | -4.65244 | -2.9286 |
| 70 | 1 | 0 | 0.970538 | -5.06224 | 0.806519 |
| 71 | 1 | 0 | -4.58903 | -4.032 | -2.42146 |
| 72 | 1 | 0 | -2.40311 | -4.6955 | 1.232954 |
| 73 | 1 | 0 | -1.74922 | -2.31556 | 1.177579 |
| 74 | 1 | 0 | -3.90775 | -1.67129 | -2.30731 |
| 75 | 1 | 0 | -3.88585 | 2.796827 | -0.03297 |
| 76 | 1 | 0 | -0.02324 | 2.116196 | -1.39059 |
| 77 | 1 | 0 | -3.68065 | 5.219308 | -0.41106 |
| 78 | 1 | 0 | 0.314084 | 4.51192 | -1.8651 |
| 79 | 8 | 0 | 1.392077 | 0.192135 | -2.5194 |
| 80 | 6 | 0 | 4.042649 | 6.045475 | -0.27473 |
| 81 | 1 | 0 | 3.470139 | 6.650521 | 0.431847 |
| 82 | 1 | 0 | 5.086498 | 6.045877 | 0.063642 |
| 83 | 1 | 0 | 4.025161 | 6.528283 | -1.2552 |
| 84 | 6 | 0 | 2.04792 | -6.58873 | -1.24398 |
| 85 | 1 | 0 | 3.053446 | -6.96511 | -1.01939 |
| 86 | 1 | 0 | 1.349305 | -7.08449 | -0.56705 |
| 87 | 1 | 0 | 1.819699 | -6.88803 | -2.271 |
| 88 | 6 | 0 | -4.0107 | -6.02729 | -0.59572 |
| 89 | 1 | 0 | -5.08536 | -6.07967 | -0.38407 |
| 90 | 1 | 0 | -3.85925 | -6.49023 | -1.57576 |
| 91 | 1 | 0 | -3.49077 | -6.621 | 0.158346 |
| 92 | 6 | 0 | -1.51991 | 6.563359 | -1.4931 |
| 93 | 1 | 0 | -1.99545 | 7.177152 | -0.72344 |
| 94 | 1 | 0 | -0.47328 | 6.860877 | -1.58548 |
| 95 | 1 | 0 | -2.0162 | 6.798212 | -2.44286 |
| 96 | 1 | 0 | 1.981007 | -0.06868 | -3.24442 |
| 97 | 8 | 0 | -1.47027 | 0.084407 | -2.67453 |
| 98 | 1 | 0 | -1.96194 | -0.60472 | -3.15119 |

| | | | | | |
|---|---|---|---|---|---|
| 99 | 1 | 0 | -1.74276 | 0.916114 | -3.10187 |
| 100 | 1 | 0 | 3.157889 | -0.4952 | 4.372675 |
| 101 | 1 | 0 | 0.485341 | -0.10041 | -2.72339 |
| 102 | 1 | 0 | -3.23794 | 0.409039 | 4.353721 |

**Table S31.** Cartesian coordinates for $Ru^{III}Ru^{IV}$-6pic.

| Center number | Atomic number | Atomic type | Coordinates (Ångström) | | |
|---|---|---|---|---|---|
| | | | X | Y | Z |
| 1 | 6 | 0 | -4.49664 | 0.668222 | -2.08832 |
| 2 | 6 | 0 | -4.47415 | 0.815654 | -3.50905 |
| 3 | 6 | 0 | -5.69201 | 1.042195 | -4.17537 |
| 4 | 6 | 0 | -6.84862 | 1.093157 | -3.39537 |
| 5 | 6 | 0 | -6.8332 | 0.91595 | -1.99099 |
| 6 | 6 | 0 | -5.63675 | 0.68632 | -1.29525 |
| 7 | 6 | 0 | -2.46553 | 0.462558 | -2.86046 |
| 8 | 6 | 0 | -5.59145 | 0.444286 | 0.191446 |
| 9 | 8 | 0 | -6.61058 | 0.541675 | 0.855597 |
| 10 | 8 | 0 | -4.43426 | 0.098964 | 0.766424 |
| 11 | 7 | 0 | -3.20755 | 0.468593 | -1.70566 |
| 12 | 7 | 0 | -3.18092 | 0.677073 | -3.97152 |
| 13 | 6 | 0 | -1.8E-05 | -5.6E-05 | -3.49669 |
| 14 | 6 | 0 | 1.075582 | -0.20557 | -2.64362 |
| 15 | 7 | 0 | -0.66096 | 0.099912 | -1.2988 |
| 16 | 7 | 0 | 0.660939 | -0.09995 | -1.2988 |
| 17 | 6 | 0 | -1.07561 | 0.205477 | -2.64361 |
| 18 | 6 | 0 | 4.49661 | -0.66836 | -2.08833 |
| 19 | 6 | 0 | 4.474107 | -0.81581 | -3.50905 |
| 20 | 6 | 0 | 5.691961 | -1.04237 | -4.17538 |
| 21 | 6 | 0 | 6.848575 | -1.09333 | -3.39538 |
| 22 | 6 | 0 | 6.833166 | -0.91611 | -1.99101 |
| 23 | 6 | 0 | 5.636717 | -0.68647 | -1.29526 |
| 24 | 6 | 0 | 2.465496 | -0.46267 | -2.86047 |
| 25 | 7 | 0 | 3.20752 | -0.46868 | -1.70567 |
| 26 | 7 | 0 | 3.180875 | -0.67723 | -3.97152 |
| 27 | 6 | 0 | 5.591414 | -0.44447 | 0.191438 |
| 28 | 8 | 0 | 6.610615 | -0.54152 | 0.855531 |
| 29 | 8 | 0 | 4.434256 | -0.09897 | 0.766381 |
| 30 | 44 | 0 | 2.508366 | -0.01368 | 0.030885 |
| 31 | 44 | 0 | -2.50837 | 0.013685 | 0.030913 |
| 32 | 6 | 0 | 3.124375 | -2.57549 | 1.568222 |
| 33 | 6 | 0 | 1.656903 | -2.97245 | -0.17448 |
| 34 | 6 | 0 | 3.175848 | -3.92785 | 1.877308 |
| 35 | 6 | 0 | 1.67135 | -4.33615 | 0.074618 |
| 36 | 6 | 0 | 2.447676 | -4.85663 | 1.121281 |
| 37 | 6 | 0 | 3.834871 | 2.712325 | 0.235485 |
| 38 | 6 | 0 | 2.187966 | 2.751889 | -1.38608 |
| 39 | 6 | 0 | 4.18195 | 4.027856 | -0.03775 |
| 40 | 6 | 0 | 2.490669 | 4.065186 | -1.71416 |
| 41 | 6 | 0 | 3.516011 | 4.744946 | -1.04056 |
| 42 | 6 | 0 | -3.12437 | 2.57554 | 1.56818 |

| | | | | | |
|---|---|---|---|---|---|
| 43 | 6 | 0 | -1.657 | 2.972471 | -0.17462 |
| 44 | 6 | 0 | -3.17586 | 3.927907 | 1.877216 |
| 45 | 6 | 0 | -1.67147 | 4.336177 | 0.074424 |
| 46 | 6 | 0 | -2.44776 | 4.856683 | 1.121108 |
| 47 | 6 | 0 | -3.83486 | -2.71232 | 0.235619 |
| 48 | 6 | 0 | -2.18791 | -2.75196 | -1.3859 |
| 49 | 6 | 0 | -4.18192 | -4.02787 | -0.03755 |
| 50 | 6 | 0 | -2.49059 | -4.06528 | -1.71391 |
| 51 | 6 | 0 | -3.51594 | -4.74501 | -1.0403 |
| 52 | 6 | 0 | 2.818927 | 0.710216 | 3.029172 |
| 53 | 6 | 0 | 0.770506 | 1.396885 | 2.213678 |
| 54 | 6 | 0 | 2.570776 | 1.308607 | 4.256565 |
| 55 | 6 | 0 | 0.463274 | 2.032256 | 3.40961 |
| 56 | 6 | 0 | 1.37054 | 1.999483 | 4.477589 |
| 57 | 6 | 0 | -2.81883 | -0.70996 | 3.029272 |
| 58 | 6 | 0 | -0.77051 | -1.39686 | 2.213713 |
| 59 | 6 | 0 | -2.57066 | -1.30828 | 4.256694 |
| 60 | 6 | 0 | -0.46326 | -2.03216 | 3.409676 |
| 61 | 6 | 0 | -1.37046 | -1.99924 | 4.477702 |
| 62 | 7 | 0 | -2.84538 | -2.06603 | -0.42463 |
| 63 | 7 | 0 | -2.3685 | 2.085897 | 0.558391 |
| 64 | 7 | 0 | -1.92912 | -0.73708 | 2.00949 |
| 65 | 7 | 0 | 1.929156 | 0.737186 | 2.009439 |
| 66 | 7 | 0 | 2.368459 | -2.08587 | 0.558459 |
| 67 | 7 | 0 | 2.845403 | 2.06601 | -0.42476 |
| 68 | 1 | 0 | 5.728781 | -1.16519 | -5.25245 |
| 69 | 1 | 0 | 7.803009 | -1.26591 | -3.88177 |
| 70 | 1 | 0 | 7.758439 | -0.94789 | -1.42508 |
| 71 | 1 | 0 | -2.3E-05 | -7.5E-05 | -4.57595 |
| 72 | 1 | 0 | -5.72883 | 1.165001 | -5.25244 |
| 73 | 1 | 0 | -7.80306 | 1.265711 | -3.88175 |
| 74 | 1 | 0 | -7.75847 | 0.947709 | -1.42507 |
| 75 | 1 | 0 | 1.068083 | -2.55797 | -0.98194 |
| 76 | 1 | 0 | 3.725072 | -1.85514 | 2.106105 |
| 77 | 1 | 0 | 3.808575 | -4.25374 | 2.69678 |
| 78 | 1 | 0 | 1.081505 | -4.99306 | -0.55643 |
| 79 | 1 | 0 | 4.366696 | 2.131877 | 0.978369 |
| 80 | 1 | 0 | 1.40596 | 2.221221 | -1.91217 |
| 81 | 1 | 0 | 4.98891 | 4.483391 | 0.527296 |
| 82 | 1 | 0 | 1.932619 | 4.551083 | -2.5085 |
| 83 | 1 | 0 | -3.80855 | 4.253813 | 2.696713 |
| 84 | 1 | 0 | -1.08168 | 4.993079 | -0.55668 |
| 85 | 1 | 0 | -1.06822 | 2.557971 | -0.9821 |
| 86 | 1 | 0 | -3.72501 | 1.855195 | 2.106129 |
| 87 | 1 | 0 | -4.36671 | -2.13183 | 0.978452 |
| 88 | 1 | 0 | -1.40589 | -2.22132 | -1.91199 |
| 89 | 1 | 0 | -4.98889 | -4.48338 | 0.527493 |
| 90 | 1 | 0 | -1.93251 | -4.55122 | -2.50821 |
| 91 | 1 | 0 | 3.766253 | 0.228851 | 2.814234 |
| 92 | 1 | 0 | 0.075443 | 1.407303 | 1.384281 |
| 93 | 1 | 0 | 3.326992 | 1.248463 | 5.033171 |
| 94 | 1 | 0 | -0.48461 | 2.552431 | 3.503342 |
| 95 | 1 | 0 | -3.76612 | -0.22852 | 2.814352 |
| 96 | 1 | 0 | -0.07549 | -1.40739 | 1.384276 |
| 97 | 1 | 0 | -3.32682 | -1.24802 | 5.033339 |

| | | | | | |
|---|---|---|---|---|---|
| 98 | 1 | 0 | 0.484592 | -2.55241 | 3.503393 |
| 99 | 6 | 0 | -2.52609 | 6.332704 | 1.391862 |
| 100 | 1 | 0 | -1.61278 | 6.848388 | 1.085102 |
| 101 | 1 | 0 | -3.35654 | 6.772187 | 0.825752 |
| 102 | 1 | 0 | -2.70994 | 6.538309 | 2.449213 |
| 103 | 6 | 0 | 3.904557 | 6.151902 | -1.39831 |
| 104 | 1 | 0 | 4.714537 | 6.139199 | -2.1379 |
| 105 | 1 | 0 | 3.069689 | 6.702718 | -1.83823 |
| 106 | 1 | 0 | 4.27019 | 6.700796 | -0.52686 |
| 107 | 6 | 0 | 1.092508 | 2.693518 | 5.782333 |
| 108 | 1 | 0 | 1.421799 | 2.088743 | 6.631961 |
| 109 | 1 | 0 | 1.642055 | 3.641179 | 5.831955 |
| 110 | 1 | 0 | 0.030453 | 2.91846 | 5.903811 |
| 111 | 6 | 0 | -1.09241 | -2.69319 | 5.782488 |
| 112 | 1 | 0 | -1.4214 | -2.08821 | 6.632091 |
| 113 | 1 | 0 | -1.64222 | -3.64069 | 5.832322 |
| 114 | 1 | 0 | -0.0304 | -2.91841 | 5.90383 |
| 115 | 6 | 0 | 2.525981 | -6.33265 | 1.392079 |
| 116 | 1 | 0 | 3.356087 | -6.77226 | 0.825558 |
| 117 | 1 | 0 | 2.710341 | -6.53822 | 2.449345 |
| 118 | 1 | 0 | 1.612451 | -6.84824 | 1.085806 |
| 119 | 6 | 0 | -3.90446 | -6.15199 | -1.39799 |
| 120 | 1 | 0 | -4.71445 | -6.13934 | -2.13757 |
| 121 | 1 | 0 | -3.06959 | -6.70281 | -1.83788 |
| 122 | 1 | 0 | -4.27009 | -6.70085 | -0.5265 |

**Table S32.** Cartesian coordinates for Ru$^{III}$Ru$^{IV}$-6pic-H$^{+}$.

| Center number | Atomic number | Atomic type | Coordinates (Ångström) | | |
|---|---|---|---|---|---|
| | | | X | Y | Z |
| 1 | 6 | 0 | -4.57922 | 0.617744 | -2.03281 |
| 2 | 6 | 0 | -4.59842 | 0.739505 | -3.44853 |
| 3 | 6 | 0 | -5.81669 | 0.942938 | -4.07056 |
| 4 | 6 | 0 | -6.97227 | 0.998936 | -3.23932 |
| 5 | 6 | 0 | -6.92901 | 0.841361 | -1.84211 |
| 6 | 6 | 0 | -5.70445 | 0.638655 | -1.19434 |
| 7 | 6 | 0 | -2.56663 | 0.391334 | -2.86526 |
| 8 | 6 | 0 | -5.58011 | 0.409583 | 0.281482 |
| 9 | 8 | 0 | -6.55373 | 0.385677 | 1.00406 |
| 10 | 8 | 0 | -4.34809 | 0.229474 | 0.839901 |
| 11 | 7 | 0 | -3.30314 | 0.418452 | -1.67545 |
| 12 | 7 | 0 | -3.28233 | 0.584185 | -3.94253 |
| 13 | 6 | 0 | -0.09819 | -0.1125 | -3.48209 |
| 14 | 6 | 0 | 0.98149 | -0.3061 | -2.62051 |
| 15 | 7 | 0 | -0.75166 | 0.057391 | -1.30575 |
| 16 | 7 | 0 | 0.594779 | -0.16772 | -1.29949 |
| 17 | 6 | 0 | -1.16378 | 0.126348 | -2.62644 |
| 18 | 6 | 0 | 4.458713 | -0.71052 | -2.18082 |
| 19 | 6 | 0 | 4.465367 | -0.95093 | -3.56718 |
| 20 | 6 | 0 | 5.667063 | -1.22568 | -4.22597 |
| 21 | 6 | 0 | 6.818466 | -1.24155 | -3.4346 |

| | | | | | |
|---|---|---|---|---|---|
| 22 | 6 | 0 | 6.799443 | -0.98969 | -2.04452 |
| 23 | 6 | 0 | 5.605423 | -0.70529 | -1.38201 |
| 24 | 6 | 0 | 2.383964 | -0.54994 | -2.8558 |
| 25 | 7 | 0 | 3.170665 | -0.48553 | -1.79102 |
| 26 | 7 | 0 | 3.126007 | -0.83772 | -3.96535 |
| 27 | 6 | 0 | 5.54189 | -0.40888 | 0.083974 |
| 28 | 8 | 0 | 6.481376 | -0.60626 | 0.823626 |
| 29 | 8 | 0 | 4.410392 | 0.154721 | 0.598138 |
| 30 | 44 | 0 | 2.52257 | 0.04564 | 0.026856 |
| 31 | 44 | 0 | -2.54345 | -0.0076 | 0.058563 |
| 32 | 6 | 0 | 3.293276 | -2.48415 | 1.57155 |
| 33 | 6 | 0 | 1.86809 | -2.96572 | -0.18467 |
| 34 | 6 | 0 | 3.437844 | -3.83075 | 1.87023 |
| 35 | 6 | 0 | 1.96558 | -4.32673 | 0.062832 |
| 36 | 6 | 0 | 2.773869 | -4.80344 | 1.106967 |
| 37 | 6 | 0 | 3.58857 | 2.890519 | 0.225284 |
| 38 | 6 | 0 | 2.095636 | 2.729348 | -1.53028 |
| 39 | 6 | 0 | 3.861623 | 4.214472 | -0.08311 |
| 40 | 6 | 0 | 2.325033 | 4.04876 | -1.8943 |
| 41 | 6 | 0 | 3.235426 | 4.836107 | -1.17356 |
| 42 | 6 | 0 | -2.92743 | 2.61958 | 1.599704 |
| 43 | 6 | 0 | -1.62604 | 2.944044 | -0.2837 |
| 44 | 6 | 0 | -2.92404 | 3.978298 | 1.879012 |
| 45 | 6 | 0 | -1.58531 | 4.312208 | -0.06198 |
| 46 | 6 | 0 | -2.25123 | 4.876384 | 1.037086 |
| 47 | 6 | 0 | -3.87317 | -2.70808 | 0.488202 |
| 48 | 6 | 0 | -2.51901 | -2.76645 | -1.39052 |
| 49 | 6 | 0 | -4.29166 | -4.01091 | 0.268657 |
| 50 | 6 | 0 | -2.8971 | -4.07246 | -1.66307 |
| 51 | 6 | 0 | -3.8144 | -4.7364 | -0.83381 |
| 52 | 6 | 0 | 2.927743 | 0.734949 | 3.016388 |
| 53 | 6 | 0 | 0.857354 | 1.413173 | 2.255498 |
| 54 | 6 | 0 | 2.701306 | 1.310063 | 4.258215 |
| 55 | 6 | 0 | 0.568869 | 2.020114 | 3.471248 |
| 56 | 6 | 0 | 1.497746 | 1.981808 | 4.520493 |
| 57 | 6 | 0 | -2.67934 | -0.5914 | 3.108309 |
| 58 | 6 | 0 | -0.74312 | -1.42324 | 2.164558 |
| 59 | 6 | 0 | -2.37377 | -1.16712 | 4.332943 |
| 60 | 6 | 0 | -0.38172 | -2.03718 | 3.356002 |
| 61 | 6 | 0 | -1.20086 | -1.9217 | 4.488906 |
| 62 | 7 | 0 | -2.99565 | -2.07549 | -0.32925 |
| 63 | 7 | 0 | -2.28526 | 2.089063 | 0.532277 |
| 64 | 7 | 0 | -1.87513 | -0.70127 | 2.024257 |
| 65 | 7 | 0 | 2.017797 | 0.768563 | 2.013512 |
| 66 | 7 | 0 | 2.516832 | -2.03644 | 0.555713 |
| 67 | 7 | 0 | 2.713078 | 2.137355 | -0.48334 |
| 68 | 1 | 0 | 5.718716 | -1.41584 | -5.29256 |
| 69 | 1 | 0 | 7.770707 | -1.45151 | -3.91005 |
| 70 | 1 | 0 | 7.723062 | -1.00702 | -1.47579 |
| 71 | 1 | 0 | -0.12217 | -0.12819 | -4.56182 |
| 72 | 1 | 0 | -5.90456 | 1.045751 | -5.14703 |
| 73 | 1 | 0 | -7.93616 | 1.157515 | -3.71228 |
| 74 | 1 | 0 | -7.84238 | 0.867943 | -1.25799 |
| 75 | 1 | 0 | 1.255085 | -2.5884 | -0.9929 |
| 76 | 1 | 0 | 3.832767 | -1.7347 | 2.133116 |

| | | | | | |
|---|---|---|---|---|---|
| 77 | 1 | 0 | 4.089199 | -4.11768 | 2.68969 |
| 78 | 1 | 0 | 1.420293 | -5.01827 | -0.57146 |
| 79 | 1 | 0 | 4.100479 | 2.392457 | 1.037295 |
| 80 | 1 | 0 | 1.402289 | 2.119988 | -2.09411 |
| 81 | 1 | 0 | 4.58195 | 4.753814 | 0.523861 |
| 82 | 1 | 0 | 1.803183 | 4.455751 | -2.75487 |
| 83 | 1 | 0 | -3.47045 | 4.33455 | 2.746674 |
| 84 | 1 | 0 | -1.04098 | 4.939691 | -0.76007 |
| 85 | 1 | 0 | -1.12162 | 2.505723 | -1.13372 |
| 86 | 1 | 0 | -3.48503 | 1.931063 | 2.218004 |
| 87 | 1 | 0 | -4.25661 | -2.1339 | 1.321336 |
| 88 | 1 | 0 | -1.82105 | -2.24972 | -2.03581 |
| 89 | 1 | 0 | -5.00477 | -4.45313 | 0.957021 |
| 90 | 1 | 0 | -2.48377 | -4.56728 | -2.53612 |
| 91 | 1 | 0 | 3.879717 | 0.274966 | 2.782247 |
| 92 | 1 | 0 | 0.14655 | 1.442416 | 1.439486 |
| 93 | 1 | 0 | 3.478266 | 1.248113 | 5.013945 |
| 94 | 1 | 0 | -0.38049 | 2.531242 | 3.593189 |
| 95 | 1 | 0 | -3.61094 | -0.05836 | 2.96108 |
| 96 | 1 | 0 | -0.11601 | -1.50903 | 1.285829 |
| 97 | 1 | 0 | -3.06481 | -1.03904 | 5.16036 |
| 98 | 1 | 0 | 0.539332 | -2.60949 | 3.396066 |
| 99 | 6 | 0 | -2.27677 | 6.357702 | 1.279193 |
| 100 | 1 | 0 | -1.41474 | 6.857012 | 0.831202 |
| 101 | 1 | 0 | -3.17832 | 6.791973 | 0.829244 |
| 102 | 1 | 0 | -2.30646 | 6.590277 | 2.346591 |
| 103 | 6 | 0 | 3.550664 | 6.251963 | -1.56245 |
| 104 | 1 | 0 | 4.500272 | 6.286271 | -2.11043 |
| 105 | 1 | 0 | 2.781163 | 6.678155 | -2.20968 |
| 106 | 1 | 0 | 3.667613 | 6.890719 | -0.68266 |
| 107 | 6 | 0 | 1.242192 | 2.647529 | 5.843401 |
| 108 | 1 | 0 | 1.531514 | 1.998805 | 6.675395 |
| 109 | 1 | 0 | 1.842544 | 3.56088 | 5.93026 |
| 110 | 1 | 0 | 0.192803 | 2.924495 | 5.964423 |
| 111 | 6 | 0 | -0.86656 | -2.59325 | 5.790673 |
| 112 | 1 | 0 | -1.07597 | -1.93799 | 6.640913 |
| 113 | 1 | 0 | -1.48356 | -3.49043 | 5.921549 |
| 114 | 1 | 0 | 0.180555 | -2.90027 | 5.833593 |
| 115 | 6 | 0 | 2.949861 | -6.27059 | 1.372344 |
| 116 | 1 | 0 | 3.861997 | -6.63034 | 0.88005 |
| 117 | 1 | 0 | 3.060984 | -6.4746 | 2.440392 |
| 118 | 1 | 0 | 2.113975 | -6.85683 | 0.984274 |
| 119 | 6 | 0 | -4.28523 | -6.13167 | -1.12461 |
| 120 | 1 | 0 | -5.23938 | -6.09697 | -1.66526 |
| 121 | 1 | 0 | -3.57465 | -6.6785 | -1.74816 |
| 122 | 1 | 0 | -4.4583 | -6.69591 | -0.20459 |
| 123 | 1 | 0 | 2.77342 | -0.96159 | -4.90454 |

**Table S33.** Cartesian coordinates for $Ru^{III}Ru^{IV}$-6pic-2$H^{+}$.

| Center number | Atomic number | Atomic type | Coordinates (Ångström) | | |
|---|---|---|---|---|---|
| | | | X | Y | Z |

| | | | | | |
|---|---|---|---|---|---|
| 1 | 6 | 0 | -4.67799 | 0.657951 | -2.02141 |
| 2 | 6 | 0 | -4.76122 | 0.790431 | -3.41768 |
| 3 | 6 | 0 | -5.99212 | 1.030195 | -4.0306 |
| 4 | 6 | 0 | -7.10169 | 1.113795 | -3.17711 |
| 5 | 6 | 0 | -7.00899 | 0.956116 | -1.77831 |
| 6 | 6 | 0 | -5.77608 | 0.715876 | -1.16186 |
| 7 | 6 | 0 | -2.641 | 0.392305 | -2.81415 |
| 8 | 6 | 0 | -5.63593 | 0.504666 | 0.31215 |
| 9 | 8 | 0 | -6.57742 | 0.60258 | 1.066216 |
| 10 | 8 | 0 | -4.41824 | 0.173339 | 0.850572 |
| 11 | 7 | 0 | -3.37188 | 0.435 | -1.69131 |
| 12 | 7 | 0 | -3.44226 | 0.612795 | -3.88226 |
| 13 | 6 | 0 | -0.1683 | -0.09504 | -3.46956 |
| 14 | 6 | 0 | 0.917657 | -0.28995 | -2.60331 |
| 15 | 7 | 0 | -0.82042 | 0.035489 | -1.28687 |
| 16 | 7 | 0 | 0.52465 | -0.17393 | -1.28739 |
| 17 | 6 | 0 | -1.23662 | 0.124442 | -2.61428 |
| 18 | 6 | 0 | 4.410176 | -0.71367 | -2.26428 |
| 19 | 6 | 0 | 4.363385 | -0.93957 | -3.65566 |
| 20 | 6 | 0 | 5.530516 | -1.21538 | -4.369 |
| 21 | 6 | 0 | 6.715876 | -1.24593 | -3.62525 |
| 22 | 6 | 0 | 6.757255 | -1.00589 | -2.23008 |
| 23 | 6 | 0 | 5.595763 | -0.72497 | -1.51495 |
| 24 | 6 | 0 | 2.311791 | -0.53278 | -2.86502 |
| 25 | 7 | 0 | 3.144813 | -0.48404 | -1.81918 |
| 26 | 7 | 0 | 3.011132 | -0.81192 | -4.00008 |
| 27 | 6 | 0 | 5.615688 | -0.43959 | -0.04758 |
| 28 | 8 | 0 | 6.614271 | -0.56694 | 0.621398 |
| 29 | 8 | 0 | 4.485808 | 0.023062 | 0.580841 |
| 30 | 44 | 0 | 2.607369 | 0.028852 | 0.039941 |
| 31 | 44 | 0 | -2.60145 | 0.005851 | 0.070817 |
| 32 | 6 | 0 | 3.269304 | -2.49584 | 1.63237 |
| 33 | 6 | 0 | 1.908728 | -2.97984 | -0.17719 |
| 34 | 6 | 0 | 3.397826 | -3.84083 | 1.94046 |
| 35 | 6 | 0 | 1.993846 | -4.33919 | 0.077928 |
| 36 | 6 | 0 | 2.763159 | -4.81666 | 1.153409 |
| 37 | 6 | 0 | 3.79486 | 2.824657 | 0.233665 |
| 38 | 6 | 0 | 2.238736 | 2.734538 | -1.47709 |
| 39 | 6 | 0 | 4.107853 | 4.138615 | -0.07469 |
| 40 | 6 | 0 | 2.505267 | 4.046965 | -1.83651 |
| 41 | 6 | 0 | 3.470922 | 4.794339 | -1.14086 |
| 42 | 6 | 0 | -3.07389 | 2.613177 | 1.58731 |
| 43 | 6 | 0 | -1.61057 | 2.930219 | -0.18443 |
| 44 | 6 | 0 | -3.06296 | 3.966262 | 1.882067 |
| 45 | 6 | 0 | -1.56355 | 4.293183 | 0.05571 |
| 46 | 6 | 0 | -2.30814 | 4.861655 | 1.104945 |
| 47 | 6 | 0 | -4.0188 | -2.67031 | 0.414721 |
| 48 | 6 | 0 | -2.40962 | -2.81381 | -1.25018 |
| 49 | 6 | 0 | -4.40325 | -3.98366 | 0.201769 |
| 50 | 6 | 0 | -2.75153 | -4.13046 | -1.51229 |
| 51 | 6 | 0 | -3.78019 | -4.76041 | -0.78959 |
| 52 | 6 | 0 | 3.125891 | 0.724945 | 3.013099 |
| 53 | 6 | 0 | 1.018447 | 1.368511 | 2.317815 |
| 54 | 6 | 0 | 2.929847 | 1.299993 | 4.259443 |
| 55 | 6 | 0 | 0.760305 | 1.974178 | 3.539734 |

| | | | | | |
|---|---|---|---|---|---|
| 56 | 6 | 0 | 1.724057 | 1.95447 | 4.560422 |
| 57 | 6 | 0 | -2.77743 | -0.61652 | 3.078319 |
| 58 | 6 | 0 | -0.79717 | -1.37845 | 2.14635 |
| 59 | 6 | 0 | -2.46959 | -1.2 | 4.296267 |
| 60 | 6 | 0 | -0.43728 | -1.99858 | 3.33267 |
| 61 | 6 | 0 | -1.27686 | -1.92624 | 4.457422 |
| 62 | 7 | 0 | -3.03333 | -2.07031 | -0.30348 |
| 63 | 7 | 0 | -2.3581 | 2.078988 | 0.562781 |
| 64 | 7 | 0 | -1.9529 | -0.68867 | 2.001503 |
| 65 | 7 | 0 | 2.182175 | 0.74085 | 2.038927 |
| 66 | 7 | 0 | 2.533571 | -2.04799 | 0.583543 |
| 67 | 7 | 0 | 2.869343 | 2.109248 | -0.45512 |
| 68 | 1 | 0 | 5.537891 | -1.39599 | -5.43858 |
| 69 | 1 | 0 | 7.646586 | -1.45721 | -4.14168 |
| 70 | 1 | 0 | 7.706253 | -1.03166 | -1.70444 |
| 71 | 1 | 0 | -0.16802 | -0.10317 | -4.55037 |
| 72 | 1 | 0 | -6.10419 | 1.143404 | -5.10372 |
| 73 | 1 | 0 | -8.07686 | 1.299415 | -3.61521 |
| 74 | 1 | 0 | -7.90175 | 1.014092 | -1.16406 |
| 75 | 1 | 0 | 1.328617 | -2.6065 | -1.01108 |
| 76 | 1 | 0 | 3.788682 | -1.74751 | 2.213219 |
| 77 | 1 | 0 | 4.016599 | -4.12702 | 2.785253 |
| 78 | 1 | 0 | 1.473739 | -5.03252 | -0.57519 |
| 79 | 1 | 0 | 4.309734 | 2.305149 | 1.030281 |
| 80 | 1 | 0 | 1.503186 | 2.155461 | -2.02053 |
| 81 | 1 | 0 | 4.867316 | 4.645097 | 0.512779 |
| 82 | 1 | 0 | 1.971698 | 4.483673 | -2.67498 |
| 83 | 1 | 0 | -3.66796 | 4.324442 | 2.709299 |
| 84 | 1 | 0 | -0.95103 | 4.918359 | -0.58571 |
| 85 | 1 | 0 | -1.04166 | 2.48888 | -0.99156 |
| 86 | 1 | 0 | -3.69056 | 1.929874 | 2.153528 |
| 87 | 1 | 0 | -4.50838 | -2.06089 | 1.162573 |
| 88 | 1 | 0 | -1.61761 | -2.32962 | -1.80605 |
| 89 | 1 | 0 | -5.20373 | -4.39671 | 0.807559 |
| 90 | 1 | 0 | -2.21956 | -4.66749 | -2.29122 |
| 91 | 1 | 0 | 4.075215 | 0.272233 | 2.754204 |
| 92 | 1 | 0 | 0.282164 | 1.389054 | 1.523152 |
| 93 | 1 | 0 | 3.731332 | 1.251306 | 4.990261 |
| 94 | 1 | 0 | -0.19242 | 2.47076 | 3.69278 |
| 95 | 1 | 0 | -3.72118 | -0.10797 | 2.927966 |
| 96 | 1 | 0 | -0.15782 | -1.43366 | 1.274083 |
| 97 | 1 | 0 | -3.17459 | -1.10385 | 5.116276 |
| 98 | 1 | 0 | 0.498609 | -2.54549 | 3.378679 |
| 99 | 6 | 0 | -2.32627 | 6.337444 | 1.36318 |
| 100 | 1 | 0 | -1.45807 | 6.83972 | 0.931785 |
| 101 | 1 | 0 | -3.22294 | 6.779955 | 0.909497 |
| 102 | 1 | 0 | -2.37286 | 6.557204 | 2.433098 |
| 103 | 6 | 0 | 3.827681 | 6.198756 | -1.52748 |
| 104 | 1 | 0 | 4.781383 | 6.204191 | -2.07025 |
| 105 | 1 | 0 | 3.074828 | 6.649881 | -2.17679 |
| 106 | 1 | 0 | 3.963676 | 6.831903 | -0.64605 |
| 107 | 6 | 0 | 1.501734 | 2.615893 | 5.889472 |
| 108 | 1 | 0 | 1.782057 | 1.951088 | 6.712334 |
| 109 | 1 | 0 | 2.133397 | 3.507884 | 5.978491 |
| 110 | 1 | 0 | 0.463364 | 2.924114 | 6.025888 |

| | | | | | |
|---|---|---|---|---|---|
| 111 | 6 | 0 | -0.94486 | -2.60709 | 5.751767 |
| 112 | 1 | 0 | -1.14245 | -1.95199 | 6.605583 |
| 113 | 1 | 0 | -1.5796 | -3.49234 | 5.883606 |
| 114 | 1 | 0 | 0.096247 | -2.93315 | 5.789664 |
| 115 | 6 | 0 | 2.929895 | -6.28127 | 1.427375 |
| 116 | 1 | 0 | 3.877166 | -6.63193 | 0.99755 |
| 117 | 1 | 0 | 2.974131 | -6.48654 | 2.500216 |
| 118 | 1 | 0 | 2.128027 | -6.87445 | 0.982973 |
| 119 | 6 | 0 | -4.20828 | -6.16863 | -1.07183 |
| 120 | 1 | 0 | -5.12489 | -6.16328 | -1.67589 |
| 121 | 1 | 0 | -3.4501 | -6.72431 | -1.62707 |
| 122 | 1 | 0 | -4.44175 | -6.70693 | -0.14905 |
| 123 | 1 | 0 | 2.628655 | -0.92921 | -4.92944 |
| 124 | 1 | 0 | -3.15181 | 0.654043 | -4.85225 |

**Table S34.** Cartesian coordinates for Ru$^{III}$Ru$^{IV}$-6pic-OH-A.

| Center number | Atomic number | Atomic type | Coordinates (Ångström) | | |
|---|---|---|---|---|---|
| | | | X | Y | Z |
| 1 | 6 | 0 | -3.69339 | 1.143193 | -2.77929 |
| 2 | 6 | 0 | -3.07065 | 1.417258 | -4.01414 |
| 3 | 6 | 0 | -3.7507 | 1.931651 | -5.1165 |
| 4 | 6 | 0 | -5.11272 | 2.174752 | -4.94756 |
| 5 | 6 | 0 | -5.75099 | 1.896176 | -3.73059 |
| 6 | 6 | 0 | -5.07412 | 1.368412 | -2.62741 |
| 7 | 6 | 0 | -1.56252 | 0.627677 | -2.54888 |
| 8 | 6 | 0 | -5.87624 | 1.064484 | -1.38692 |
| 9 | 8 | 0 | -7.0508 | 1.405579 | -1.31297 |
| 10 | 8 | 0 | -5.26692 | 0.389054 | -0.43272 |
| 11 | 7 | 0 | -2.7261 | 0.655381 | -1.89071 |
| 12 | 7 | 0 | -1.74277 | 1.067719 | -3.82541 |
| 13 | 6 | 0 | 0.593798 | -0.66781 | -2.91612 |
| 14 | 6 | 0 | 1.762621 | -0.75908 | -2.1587 |
| 15 | 7 | 0 | 0.410673 | 0.625889 | -1.03972 |
| 16 | 7 | 0 | 1.619968 | 0.051888 | -1.04958 |
| 17 | 6 | 0 | -0.23252 | 0.173556 | -2.15085 |
| 18 | 6 | 0 | 5.023332 | -1.80895 | -1.40103 |
| 19 | 6 | 0 | 4.848044 | -2.53373 | -2.61162 |
| 20 | 6 | 0 | 5.904124 | -3.33048 | -3.05608 |
| 21 | 6 | 0 | 7.070874 | -3.36272 | -2.27569 |
| 22 | 6 | 0 | 7.208426 | -2.63335 | -1.07911 |
| 23 | 6 | 0 | 6.170152 | -1.82725 | -0.60044 |
| 24 | 6 | 0 | 3.03649 | -1.4458 | -2.26724 |
| 25 | 7 | 0 | 3.872851 | -1.12481 | -1.20098 |
| 26 | 7 | 0 | 3.57042 | -2.27954 | -3.13672 |
| 27 | 6 | 0 | 6.274021 | -1.03493 | 0.68547 |
| 28 | 8 | 0 | 7.308457 | -1.09041 | 1.344415 |
| 29 | 8 | 0 | 5.227756 | -0.31947 | 1.092727 |
| 30 | 44 | 0 | 3.338126 | 0.036347 | 0.232588 |
| 31 | 44 | 0 | -3.30111 | -0.08204 | 0.00568 |
| 32 | 6 | 0 | 3.388838 | -2.32415 | 2.142653 |

| | | | | | |
|---|---|---|---|---|---|
| 33 | 6 | 0 | 1.247213 | -1.92067 | 1.351639 |
| 34 | 6 | 0 | 2.95331 | -3.40587 | 2.89425 |
| 35 | 6 | 0 | 0.751007 | -2.98878 | 2.090426 |
| 36 | 6 | 0 | 1.601814 | -3.77353 | 2.877087 |
| 37 | 6 | 0 | 5.427992 | 2.143009 | -0.54946 |
| 38 | 6 | 0 | 3.368773 | 2.557122 | -1.51473 |
| 39 | 6 | 0 | 5.980787 | 3.260628 | -1.15836 |
| 40 | 6 | 0 | 3.859081 | 3.690579 | -2.15025 |
| 41 | 6 | 0 | 5.198618 | 4.067789 | -1.99455 |
| 42 | 6 | 0 | -4.12842 | 2.58606 | 1.209078 |
| 43 | 6 | 0 | -1.88475 | 2.519023 | 0.612624 |
| 44 | 6 | 0 | -4.01335 | 3.904889 | 1.626357 |
| 45 | 6 | 0 | -1.70813 | 3.840654 | 1.004126 |
| 46 | 6 | 0 | -2.78382 | 4.572014 | 1.525331 |
| 47 | 6 | 0 | -4.84605 | -2.47313 | -1.14047 |
| 48 | 6 | 0 | -2.54837 | -2.80342 | -1.14169 |
| 49 | 6 | 0 | -5.06187 | -3.71641 | -1.71722 |
| 50 | 6 | 0 | -2.69944 | -4.05538 | -1.7222 |
| 51 | 6 | 0 | -3.97547 | -4.54499 | -2.02983 |
| 52 | 6 | 0 | 3.739041 | 1.458693 | 2.922901 |
| 53 | 6 | 0 | 1.588717 | 1.783402 | 2.129093 |
| 54 | 6 | 0 | 3.46358 | 2.204156 | 4.063024 |
| 55 | 6 | 0 | 1.243773 | 2.534593 | 3.246895 |
| 56 | 6 | 0 | 2.191653 | 2.764466 | 4.25309 |
| 57 | 6 | 0 | -5.37002 | -0.79376 | 2.185089 |
| 58 | 6 | 0 | -3.18463 | -1.08481 | 2.895771 |
| 59 | 6 | 0 | -5.86134 | -1.21857 | 3.41171 |
| 60 | 6 | 0 | -3.61268 | -1.51192 | 4.146175 |
| 61 | 6 | 0 | -4.98209 | -1.59335 | 4.434492 |
| 62 | 7 | 0 | -3.60865 | -2.01773 | -0.8529 |
| 63 | 7 | 0 | -3.0795 | 1.90258 | 0.712393 |
| 64 | 7 | 0 | -4.04893 | -0.72754 | 1.922657 |
| 65 | 7 | 0 | 2.81288 | 1.25202 | 1.960742 |
| 66 | 7 | 0 | 2.556679 | -1.59242 | 1.364768 |
| 67 | 7 | 0 | 4.14105 | 1.778755 | -0.72586 |
| 68 | 1 | 0 | 5.829357 | -3.90526 | -3.97387 |
| 69 | 1 | 0 | 7.90276 | -3.97625 | -2.60924 |
| 70 | 1 | 0 | 8.125902 | -2.68464 | -0.50209 |
| 71 | 1 | 0 | 0.373956 | -1.18523 | -3.83833 |
| 72 | 1 | 0 | -3.25067 | 2.133571 | -6.05761 |
| 73 | 1 | 0 | -5.68853 | 2.577731 | -5.77435 |
| 74 | 1 | 0 | -6.81348 | 2.078972 | -3.60925 |
| 75 | 1 | 0 | 0.575396 | -1.3079 | 0.764655 |
| 76 | 1 | 0 | 4.421259 | -2.00468 | 2.153497 |
| 77 | 1 | 0 | 3.673301 | -3.95473 | 3.493008 |
| 78 | 1 | 0 | -0.31288 | -3.19881 | 2.042274 |
| 79 | 1 | 0 | 6.016006 | 1.508089 | 0.100864 |
| 80 | 1 | 0 | 2.332346 | 2.263335 | -1.61628 |
| 81 | 1 | 0 | 7.026189 | 3.492135 | -0.98095 |
| 82 | 1 | 0 | 3.190338 | 4.277679 | -2.77196 |
| 83 | 1 | 0 | -4.8898 | 4.412142 | 2.016471 |
| 84 | 1 | 0 | -0.72964 | 4.299708 | 0.893476 |
| 85 | 1 | 0 | -1.08046 | 1.92336 | 0.188706 |
| 86 | 1 | 0 | -5.07113 | 2.053559 | 1.236136 |
| 87 | 1 | 0 | -5.6627 | -1.7983 | -0.9132 |

| | | | | | |
|---|---|---|---|---|---|
| 88 | 1 | 0 | -1.57425 | -2.4049 | -0.88792 |
| 89 | 1 | 0 | -6.08 | -4.03038 | -1.92571 |
| 90 | 1 | 0 | -1.81278 | -4.64433 | -1.93407 |
| 91 | 1 | 0 | 4.709387 | 1.005628 | 2.742646 |
| 92 | 1 | 0 | 0.879932 | 1.570504 | 1.332946 |
| 93 | 1 | 0 | 4.246446 | 2.345336 | 4.802246 |
| 94 | 1 | 0 | 0.236725 | 2.934304 | 3.328485 |
| 95 | 1 | 0 | -6.0228 | -0.48854 | 1.377221 |
| 96 | 1 | 0 | -2.13549 | -1.00905 | 2.636078 |
| 97 | 1 | 0 | -6.93576 | -1.25268 | 3.563978 |
| 98 | 1 | 0 | -2.87292 | -1.77801 | 4.895023 |
| 99 | 8 | 0 | -1.67663 | -0.55675 | 0.536708 |
| 100 | 6 | 0 | -2.64321 | 6.014658 | 1.931914 |
| 101 | 1 | 0 | -3.03908 | 6.672398 | 1.149056 |
| 102 | 1 | 0 | -3.20757 | 6.224395 | 2.844973 |
| 103 | 1 | 0 | -1.59857 | 6.29017 | 2.095861 |
| 104 | 6 | 0 | -5.48801 | -2.08232 | 5.764676 |
| 105 | 1 | 0 | -5.67768 | -3.16186 | 5.724553 |
| 106 | 1 | 0 | -4.76086 | -1.90571 | 6.561301 |
| 107 | 1 | 0 | -6.42866 | -1.59627 | 6.035908 |
| 108 | 6 | 0 | -4.17174 | -5.88349 | -2.68878 |
| 109 | 1 | 0 | -4.19551 | -5.77081 | -3.77939 |
| 110 | 1 | 0 | -3.35712 | -6.57141 | -2.44935 |
| 111 | 1 | 0 | -5.11752 | -6.34223 | -2.38842 |
| 112 | 6 | 0 | 1.099019 | -4.96214 | 3.654193 |
| 113 | 1 | 0 | 0.016465 | -4.91697 | 3.80429 |
| 114 | 1 | 0 | 1.320703 | -5.89292 | 3.119062 |
| 115 | 1 | 0 | 1.583336 | -5.03122 | 4.632726 |
| 116 | 6 | 0 | 1.862652 | 3.554808 | 5.492163 |
| 117 | 1 | 0 | 1.766524 | 2.892003 | 6.360004 |
| 118 | 1 | 0 | 2.655337 | 4.27284 | 5.723965 |
| 119 | 1 | 0 | 0.922762 | 4.101361 | 5.383563 |
| 120 | 6 | 0 | 5.776513 | 5.262091 | -2.70631 |
| 121 | 1 | 0 | 6.28436 | 4.952897 | -3.62779 |
| 122 | 1 | 0 | 4.997296 | 5.9769 | -2.98193 |
| 123 | 1 | 0 | 6.518642 | 5.774854 | -2.08785 |
| 124 | 1 | 0 | -0.98451 | 1.170627 | -4.48246 |

**Table S35.** Cartesian coordinates for $Ru^{III}Ru^{IV}$-6pic-$OH_2$-A.

| Center number | Atomic number | Atomic type | Coordinates (Ångström) | | |
|---|---|---|---|---|---|
| | | | X | Y | Z |
| 1 | 6 | 0 | -3.57099 | 1.805124 | -2.44181 |
| 2 | 6 | 0 | -2.88203 | 2.409068 | -3.51336 |
| 3 | 6 | 0 | -3.49143 | 3.282427 | -4.4161 |
| 4 | 6 | 0 | -4.83994 | 3.547684 | -4.20709 |
| 5 | 6 | 0 | -5.54333 | 2.955022 | -3.14365 |
| 6 | 6 | 0 | -4.94146 | 2.070539 | -2.24858 |
| 7 | 6 | 0 | -1.48379 | 1.132434 | -2.32081 |
| 8 | 6 | 0 | -5.78606 | 1.452036 | -1.16758 |
| 9 | 8 | 0 | -6.91457 | 1.863128 | -0.94932 |

| | | | | | |
|---|---|---|---|---|---|
| 10 | 8 | 0 | -5.25576 | 0.433111 | -0.51643 |
| 11 | 7 | 0 | -2.6697 | 1.011488 | -1.72117 |
| 12 | 7 | 0 | -1.58134 | 1.950673 | -3.40785 |
| 13 | 6 | 0 | 0.529304 | -0.36709 | -2.82561 |
| 14 | 6 | 0 | 1.709655 | -0.58413 | -2.12494 |
| 15 | 7 | 0 | 0.518471 | 0.841002 | -0.88909 |
| 16 | 7 | 0 | 1.662301 | 0.158899 | -0.95123 |
| 17 | 6 | 0 | -0.18862 | 0.518418 | -1.9987 |
| 18 | 6 | 0 | 4.947628 | -1.82762 | -1.55328 |
| 19 | 6 | 0 | 4.778865 | -2.50255 | -2.77633 |
| 20 | 6 | 0 | 5.802403 | -3.31383 | -3.26785 |
| 21 | 6 | 0 | 6.95758 | -3.40639 | -2.48241 |
| 22 | 6 | 0 | 7.107828 | -2.72804 | -1.25548 |
| 23 | 6 | 0 | 6.094867 | -1.90418 | -0.75993 |
| 24 | 6 | 0 | 2.94899 | -1.30005 | -2.28962 |
| 25 | 7 | 0 | 3.815266 | -1.11483 | -1.30011 |
| 26 | 7 | 0 | 3.493648 | -2.14531 | -3.21811 |
| 27 | 6 | 0 | 6.202421 | -1.16854 | 0.551611 |
| 28 | 8 | 0 | 7.09103 | -1.43153 | 1.339908 |
| 29 | 8 | 0 | 5.316322 | -0.18725 | 0.820118 |
| 30 | 44 | 0 | 3.384379 | 0.058148 | 0.265856 |
| 31 | 44 | 0 | -3.32558 | -0.162 | -0.06771 |
| 32 | 6 | 0 | 3.54016 | -2.22278 | 2.281545 |
| 33 | 6 | 0 | 1.493373 | -2.19051 | 1.193794 |
| 34 | 6 | 0 | 3.169043 | -3.33352 | 3.022883 |
| 35 | 6 | 0 | 1.063137 | -3.30081 | 1.910802 |
| 36 | 6 | 0 | 1.904603 | -3.91422 | 2.845904 |
| 37 | 6 | 0 | 5.26453 | 1.978867 | -1.231 |
| 38 | 6 | 0 | 3.189334 | 2.910155 | -0.81145 |
| 39 | 6 | 0 | 5.707884 | 3.148655 | -1.83122 |
| 40 | 6 | 0 | 3.572977 | 4.110664 | -1.39398 |
| 41 | 6 | 0 | 4.86208 | 4.262448 | -1.92189 |
| 42 | 6 | 0 | -4.26898 | 2.071708 | 1.790754 |
| 43 | 6 | 0 | -2.01363 | 2.237855 | 1.278929 |
| 44 | 6 | 0 | -4.22084 | 3.232254 | 2.54918 |
| 45 | 6 | 0 | -1.90278 | 3.408337 | 2.020103 |
| 46 | 6 | 0 | -3.01887 | 3.941477 | 2.678462 |
| 47 | 6 | 0 | -4.75729 | -2.07755 | -2.00476 |
| 48 | 6 | 0 | -2.63024 | -2.80919 | -1.46155 |
| 49 | 6 | 0 | -4.97408 | -3.19209 | -2.80311 |
| 50 | 6 | 0 | -2.78558 | -3.94731 | -2.24164 |
| 51 | 6 | 0 | -3.98027 | -4.17051 | -2.93919 |
| 52 | 6 | 0 | 4.004976 | 1.734487 | 2.729971 |
| 53 | 6 | 0 | 1.720332 | 1.48542 | 2.427874 |
| 54 | 6 | 0 | 3.806282 | 2.451752 | 3.900943 |
| 55 | 6 | 0 | 1.454655 | 2.189639 | 3.596013 |
| 56 | 6 | 0 | 2.507433 | 2.694539 | 4.37077 |
| 57 | 6 | 0 | -5.52617 | -1.42877 | 1.670906 |
| 58 | 6 | 0 | -3.3999 | -1.83659 | 2.496931 |
| 59 | 6 | 0 | -6.10203 | -2.14838 | 2.708742 |
| 60 | 6 | 0 | -3.9161 | -2.56434 | 3.560612 |
| 61 | 6 | 0 | -5.29958 | -2.74348 | 3.691628 |
| 62 | 7 | 0 | -3.59958 | -1.87967 | -1.33728 |
| 63 | 7 | 0 | -3.18189 | 1.575302 | 1.160577 |
| 64 | 7 | 0 | -4.18801 | -1.27197 | 1.558613 |

| | | | | | |
|---|---|---|---|---|---|
| 65 | 7 | 0 | 2.976605 | 1.254109 | 1.995259 |
| 66 | 7 | 0 | 2.723603 | -1.65746 | 1.35932 |
| 67 | 7 | 0 | 4.020568 | 1.850568 | -0.71973 |
| 68 | 1 | 0 | 5.721533 | -3.85191 | -4.20631 |
| 69 | 1 | 0 | 7.772293 | -4.03069 | -2.83395 |
| 70 | 1 | 0 | 8.019135 | -2.83818 | -0.67765 |
| 71 | 1 | 0 | 0.220375 | -0.78361 | -3.77339 |
| 72 | 1 | 0 | -2.9415 | 3.734011 | -5.23523 |
| 73 | 1 | 0 | -5.36121 | 4.2225 | -4.8779 |
| 74 | 1 | 0 | -6.59548 | 3.170037 | -2.99198 |
| 75 | 1 | 0 | 0.834597 | -1.69674 | 0.489545 |
| 76 | 1 | 0 | 4.511678 | -1.76351 | 2.414245 |
| 77 | 1 | 0 | 3.875421 | -3.74364 | 3.737745 |
| 78 | 1 | 0 | 0.063068 | -3.68211 | 1.731092 |
| 79 | 1 | 0 | 5.913555 | 1.119379 | -1.13465 |
| 80 | 1 | 0 | 2.191018 | 2.771837 | -0.41405 |
| 81 | 1 | 0 | 6.719871 | 3.186631 | -2.22171 |
| 82 | 1 | 0 | 2.860278 | 4.928392 | -1.43272 |
| 83 | 1 | 0 | -5.12959 | 3.585062 | 3.026263 |
| 84 | 1 | 0 | -0.94093 | 3.908894 | 2.072147 |
| 85 | 1 | 0 | -1.16741 | 1.809817 | 0.752062 |
| 86 | 1 | 0 | -5.19434 | 1.52767 | 1.649579 |
| 87 | 1 | 0 | -5.51216 | -1.31297 | -1.87197 |
| 88 | 1 | 0 | -1.72078 | -2.60595 | -0.90836 |
| 89 | 1 | 0 | -5.92597 | -3.29249 | -3.31496 |
| 90 | 1 | 0 | -1.97099 | -4.66256 | -2.30073 |
| 91 | 1 | 0 | 4.997602 | 1.507537 | 2.35685 |
| 92 | 1 | 0 | 0.92028 | 1.091563 | 1.809182 |
| 93 | 1 | 0 | 4.670916 | 2.813444 | 4.448613 |
| 94 | 1 | 0 | 0.422914 | 2.33913 | 3.897599 |
| 95 | 1 | 0 | -6.12164 | -0.93753 | 0.909706 |
| 96 | 1 | 0 | -2.33519 | -1.6827 | 2.363347 |
| 97 | 1 | 0 | -7.18332 | -2.23627 | 2.746694 |
| 98 | 1 | 0 | -3.23374 | -2.98906 | 4.290186 |
| 99 | 8 | 0 | -1.69332 | -0.7306 | 0.39746 |
| 100 | 6 | 0 | -2.94658 | 5.220089 | 3.468154 |
| 101 | 1 | 0 | -3.51752 | 6.010793 | 2.968414 |
| 102 | 1 | 0 | -3.38222 | 5.093126 | 4.464241 |
| 103 | 1 | 0 | -1.91782 | 5.569311 | 3.581484 |
| 104 | 6 | 0 | -5.89797 | -3.54172 | 4.817564 |
| 105 | 1 | 0 | -6.25487 | -4.51126 | 4.450714 |
| 106 | 1 | 0 | -5.17094 | -3.7308 | 5.610455 |
| 107 | 1 | 0 | -6.75974 | -3.0266 | 5.252088 |
| 108 | 6 | 0 | 2.267472 | 3.446806 | 5.651445 |
| 109 | 1 | 0 | 2.831807 | 4.384395 | 5.668537 |
| 110 | 1 | 0 | 1.209399 | 3.6767 | 5.794542 |
| 111 | 1 | 0 | 2.603233 | 2.855829 | 6.51118 |
| 112 | 6 | 0 | 5.325754 | 5.555427 | -2.53424 |
| 113 | 1 | 0 | 5.938568 | 5.377368 | -3.42233 |
| 114 | 1 | 0 | 4.485355 | 6.19534 | -2.81246 |
| 115 | 1 | 0 | 5.945701 | 6.111455 | -1.82083 |
| 116 | 6 | 0 | 1.487256 | -5.13162 | 3.624184 |
| 117 | 1 | 0 | 0.44542 | -5.39752 | 3.432752 |
| 118 | 1 | 0 | 2.111432 | -5.99132 | 3.355959 |
| 119 | 1 | 0 | 1.61229 | -4.97064 | 4.699863 |

| | | | | | |
|---|---|---|---|---|---|
| 120 | 6 | 0 | -4.19928 | -5.40457 | -3.77117 |
| 121 | 1 | 0 | -4.73037 | -5.16984 | -4.69793 |
| 122 | 1 | 0 | -3.25608 | -5.89434 | -4.0241 |
| 123 | 1 | 0 | -4.81322 | -6.12861 | -3.22235 |
| 124 | 1 | 0 | -0.79651 | 2.219076 | -3.98283 |
| 125 | 1 | 0 | 3.051141 | -2.45134 | -4.07174 |

**Table S36.** Cartesian coordinates for $Ru^{III}Ru^{IV}$-6pic-$H^+$-$OH_2$-A.

| Center number | Atomic number | Atomic type | Coordinates (Ångström) | | |
|---|---|---|---|---|---|
| | | | X | Y | Z |
| 1 | 6 | 0 | 3.740524 | -1.67014 | -2.50202 |
| 2 | 6 | 0 | 3.15125 | -2.52906 | -3.4462 |
| 3 | 6 | 0 | 3.894025 | -3.36328 | -4.28524 |
| 4 | 6 | 0 | 5.278245 | -3.30677 | -4.15813 |
| 5 | 6 | 0 | 5.890588 | -2.42856 | -3.24711 |
| 6 | 6 | 0 | 5.144841 | -1.58913 | -2.41631 |
| 7 | 6 | 0 | 1.561129 | -1.40814 | -2.33173 |
| 8 | 6 | 0 | 5.850649 | -0.60389 | -1.55757 |
| 9 | 8 | 0 | 7.037026 | -0.61589 | -1.32785 |
| 10 | 8 | 0 | 5.052718 | 0.388803 | -1.08172 |
| 11 | 7 | 0 | 2.728102 | -0.99961 | -1.80111 |
| 12 | 7 | 0 | 1.789809 | -2.32574 | -3.3124 |
| 13 | 6 | 0 | -0.86133 | -1.00216 | -3.00349 |
| 14 | 6 | 0 | -1.981 | -0.65341 | -2.25663 |
| 15 | 7 | 0 | -0.29272 | -0.69749 | -0.82709 |
| 16 | 7 | 0 | -1.60913 | -0.47073 | -0.93663 |
| 17 | 6 | 0 | 0.181202 | -1.01553 | -2.05851 |
| 18 | 6 | 0 | -5.41943 | -0.10123 | -1.74956 |
| 19 | 6 | 0 | -5.52743 | -0.24499 | -3.14417 |
| 20 | 6 | 0 | -6.77601 | -0.12806 | -3.76098 |
| 21 | 6 | 0 | -7.86182 | 0.137229 | -2.92078 |
| 22 | 6 | 0 | -7.7367 | 0.279611 | -1.52103 |
| 23 | 6 | 0 | -6.49646 | 0.151053 | -0.89534 |
| 24 | 6 | 0 | -3.39607 | -0.48232 | -2.49804 |
| 25 | 7 | 0 | -4.1086 | -0.24542 | -1.40449 |
| 26 | 7 | 0 | -4.21814 | -0.48774 | -3.59036 |
| 27 | 6 | 0 | -6.30324 | 0.276628 | 0.590187 |
| 28 | 8 | 0 | -7.15799 | 0.747529 | 1.308681 |
| 29 | 8 | 0 | -5.15798 | -0.22141 | 1.130686 |
| 30 | 44 | 0 | -3.29502 | -0.18615 | 0.428237 |
| 31 | 44 | 0 | 3.313985 | 0.32943 | -0.20707 |
| 32 | 6 | 0 | -4.00472 | 2.614056 | 1.327409 |
| 33 | 6 | 0 | -2.82851 | 2.660486 | -0.6676 |
| 34 | 6 | 0 | -4.17633 | 3.989055 | 1.298981 |
| 35 | 6 | 0 | -2.9628 | 4.039086 | -0.75372 |
| 36 | 6 | 0 | -3.65957 | 4.746732 | 0.236936 |
| 37 | 6 | 0 | -4.46745 | -3.02611 | 0.464878 |
| 38 | 6 | 0 | -2.208 | -3.01275 | 0.953242 |
| 39 | 6 | 0 | -4.53431 | -4.40049 | 0.640381 |
| 40 | 6 | 0 | -2.2072 | -4.38703 | 1.148089 |

| | | | | | |
|---|---|---|---|---|---|
| 41 | 6 | 0 | -3.38959 | -5.12609 | 0.999133 |
| 42 | 6 | 0 | 5.269868 | -1.35094 | 1.447907 |
| 43 | 6 | 0 | 3.207896 | -2.38841 | 1.209169 |
| 44 | 6 | 0 | 5.753837 | -2.41078 | 2.198868 |
| 45 | 6 | 0 | 3.632888 | -3.47646 | 1.955657 |
| 46 | 6 | 0 | 4.937487 | -3.51838 | 2.472647 |
| 47 | 6 | 0 | 3.29077 | 2.226363 | -2.61116 |
| 48 | 6 | 0 | 1.973624 | 2.99745 | -0.86333 |
| 49 | 6 | 0 | 3.03712 | 3.370112 | -3.35241 |
| 50 | 6 | 0 | 1.683829 | 4.164771 | -1.55479 |
| 51 | 6 | 0 | 2.224022 | 4.38788 | -2.83056 |
| 52 | 6 | 0 | -3.3648 | -0.52326 | 3.453784 |
| 53 | 6 | 0 | -1.31674 | 0.314288 | 2.774565 |
| 54 | 6 | 0 | -2.97807 | -0.48586 | 4.78547 |
| 55 | 6 | 0 | -0.86993 | 0.379041 | 4.088203 |
| 56 | 6 | 0 | -1.70299 | -0.02311 | 5.141648 |
| 57 | 6 | 0 | 5.115498 | 2.442992 | 1.044299 |
| 58 | 6 | 0 | 3.50288 | 1.666618 | 2.529514 |
| 59 | 6 | 0 | 5.641574 | 3.288457 | 2.007862 |
| 60 | 6 | 0 | 3.987665 | 2.49233 | 3.533728 |
| 61 | 6 | 0 | 5.085421 | 3.333245 | 3.29669 |
| 62 | 7 | 0 | 2.771752 | 2.036317 | -1.37702 |
| 63 | 7 | 0 | 4.010628 | -1.33279 | 0.950557 |
| 64 | 7 | 0 | 4.05573 | 1.63821 | 1.297373 |
| 65 | 7 | 0 | -2.54848 | -0.12881 | 2.447931 |
| 66 | 7 | 0 | -3.34186 | 1.942142 | 0.354444 |
| 67 | 7 | 0 | -3.32074 | -2.32592 | 0.618764 |
| 68 | 1 | 0 | -6.91191 | -0.23318 | -4.83195 |
| 69 | 1 | 0 | -8.8464 | 0.236898 | -3.36534 |
| 70 | 1 | 0 | -8.61197 | 0.486051 | -0.91427 |
| 71 | 1 | 0 | -0.80337 | -1.18635 | -4.06649 |
| 72 | 1 | 0 | 3.418217 | -4.02078 | -5.00525 |
| 73 | 1 | 0 | 5.898301 | -3.93629 | -4.78703 |
| 74 | 1 | 0 | 6.972175 | -2.3691 | -3.18715 |
| 75 | 1 | 0 | -2.30217 | 2.100331 | -1.43122 |
| 76 | 1 | 0 | -4.4271 | 2.014674 | 2.124043 |
| 77 | 1 | 0 | -4.73138 | 4.464078 | 2.101548 |
| 78 | 1 | 0 | -2.53801 | 4.558851 | -1.60653 |
| 79 | 1 | 0 | -5.35005 | -2.45423 | 0.213554 |
| 80 | 1 | 0 | -1.30123 | -2.42992 | 1.06155 |
| 81 | 1 | 0 | -5.48789 | -4.90043 | 0.503424 |
| 82 | 1 | 0 | -1.28095 | -4.88162 | 1.422621 |
| 83 | 1 | 0 | 6.77488 | -2.36898 | 2.563947 |
| 84 | 1 | 0 | 2.943768 | -4.29545 | 2.134157 |
| 85 | 1 | 0 | 2.203021 | -2.33552 | 0.809512 |
| 86 | 1 | 0 | 5.89935 | -0.50125 | 1.216533 |
| 87 | 1 | 0 | 3.940468 | 1.446681 | -2.9863 |
| 88 | 1 | 0 | 1.571743 | 2.806895 | 0.124399 |
| 89 | 1 | 0 | 3.488751 | 3.469413 | -4.3343 |
| 90 | 1 | 0 | 1.045087 | 4.907302 | -1.08765 |
| 91 | 1 | 0 | -4.35463 | -0.8523 | 3.159847 |
| 92 | 1 | 0 | -0.67612 | 0.621721 | 1.95483 |
| 93 | 1 | 0 | -3.68117 | -0.81399 | 5.544643 |
| 94 | 1 | 0 | 0.130122 | 0.749769 | 4.288972 |
| 95 | 1 | 0 | 5.546485 | 2.373093 | 0.053157 |

| | | | | | |
|---|---|---|---|---|---|
| 96 | 1 | 0 | 2.654441 | 1.010687 | 2.683118 |
| 97 | 1 | 0 | 6.494307 | 3.908254 | 1.749537 |
| 98 | 1 | 0 | 3.509252 | 2.474112 | 4.507471 |
| 99 | 8 | 0 | 1.646114 | 0.273702 | 0.65669 |
| 100 | 1 | 0 | 0.830687 | -0.09984 | 0.172022 |
| 101 | 6 | 0 | 5.445046 | -4.69404 | 3.256893 |
| 102 | 1 | 0 | 6.032055 | -5.35271 | 2.604899 |
| 103 | 1 | 0 | 6.104507 | -4.37634 | 4.068767 |
| 104 | 1 | 0 | 4.628379 | -5.28528 | 3.676469 |
| 105 | 6 | 0 | 5.65346 | 4.225251 | 4.363072 |
| 106 | 1 | 0 | 5.782435 | 5.246725 | 3.991869 |
| 107 | 1 | 0 | 5.019066 | 4.254263 | 5.250823 |
| 108 | 1 | 0 | 6.645206 | 3.869667 | 4.666083 |
| 109 | 6 | 0 | -1.27033 | 0.050129 | 6.579515 |
| 110 | 1 | 0 | -1.49127 | -0.88388 | 7.104899 |
| 111 | 1 | 0 | -0.2022 | 0.257215 | 6.673564 |
| 112 | 1 | 0 | -1.81553 | 0.845121 | 7.101368 |
| 113 | 6 | 0 | -3.44032 | -6.60981 | 1.232766 |
| 114 | 1 | 0 | -4.05905 | -7.10901 | 0.481815 |
| 115 | 1 | 0 | -2.44437 | -7.05769 | 1.219979 |
| 116 | 1 | 0 | -3.88949 | -6.82263 | 2.210329 |
| 117 | 6 | 0 | -3.87006 | 6.23238 | 0.15975 |
| 118 | 1 | 0 | -3.23918 | 6.694733 | -0.60221 |
| 119 | 1 | 0 | -4.91441 | 6.451847 | -0.09194 |
| 120 | 1 | 0 | -3.6678 | 6.71148 | 1.122296 |
| 121 | 6 | 0 | 1.974054 | 5.65906 | -3.59107 |
| 122 | 1 | 0 | 1.823855 | 5.463514 | -4.6564 |
| 123 | 1 | 0 | 1.107421 | 6.200124 | -3.20554 |
| 124 | 1 | 0 | 2.843248 | 6.322725 | -3.50704 |
| 125 | 1 | 0 | 1.06039 | -2.8342 | -3.79192 |
| 126 | 1 | 0 | -3.93705 | -0.63338 | -4.54941 |

**Table S37.** Cartesian coordinates for $Ru^{III}Ru^{IV}$-6pic-2$H^{+}$-$OH_2$-A.

| Center number | Atomic number | Atomic type | Coordinates (Ångström) | | |
|---|---|---|---|---|---|
| | | | X | Y | Z |
| 1 | 6 | 0 | 3.56324 | -1.36606 | -2.78213 |
| 2 | 6 | 0 | 2.896873 | -1.92821 | -3.89392 |
| 3 | 6 | 0 | 3.550581 | -2.63471 | -4.90834 |
| 4 | 6 | 0 | 4.926417 | -2.76457 | -4.77931 |
| 5 | 6 | 0 | 5.61862 | -2.19558 | -3.68644 |
| 6 | 6 | 0 | 4.96755 | -1.48101 | -2.6844 |
| 7 | 6 | 0 | 1.430018 | -0.93662 | -2.52356 |
| 8 | 6 | 0 | 5.787515 | -0.84826 | -1.61414 |
| 9 | 8 | 0 | 6.94277 | -1.14079 | -1.40743 |
| 10 | 8 | 0 | 5.200501 | 0.147113 | -0.90775 |
| 11 | 7 | 0 | 2.63037 | -0.7675 | -1.9345 |
| 12 | 7 | 0 | 1.568385 | -1.62202 | -3.70106 |
| 13 | 6 | 0 | -0.96312 | -0.29192 | -3.07858 |
| 14 | 6 | 0 | -2.07735 | -0.06225 | -2.29349 |
| 15 | 7 | 0 | -0.39741 | -0.42032 | -0.88002 |

| | | | | | |
|---|---|---|---|---|---|
| 16 | 7 | 0 | -1.71792 | -0.15696 | -0.9494 |
| 17 | 6 | 0 | 0.080252 | -0.52257 | -2.14685 |
| 18 | 6 | 0 | -5.50293 | 0.579204 | -1.72005 |
| 19 | 6 | 0 | -5.59066 | 0.660985 | -3.11995 |
| 20 | 6 | 0 | -6.81947 | 0.904117 | -3.7335 |
| 21 | 6 | 0 | -7.92111 | 1.045132 | -2.87577 |
| 22 | 6 | 0 | -7.82257 | 0.94279 | -1.47182 |
| 23 | 6 | 0 | -6.59044 | 0.704597 | -0.85389 |
| 24 | 6 | 0 | -3.47379 | 0.2321 | -2.51587 |
| 25 | 7 | 0 | -4.20447 | 0.326437 | -1.39 |
| 26 | 7 | 0 | -4.27617 | 0.436415 | -3.58399 |
| 27 | 6 | 0 | -6.43832 | 0.566996 | 0.629202 |
| 28 | 8 | 0 | -7.38514 | 0.628236 | 1.378908 |
| 29 | 8 | 0 | -5.19379 | 0.373056 | 1.17887 |
| 30 | 44 | 0 | -3.40278 | 0.033629 | 0.382638 |
| 31 | 44 | 0 | 3.426712 | 0.156443 | -0.13153 |
| 32 | 6 | 0 | -3.80547 | 2.84801 | 1.512785 |
| 33 | 6 | 0 | -2.06363 | 2.811953 | -0.01936 |
| 34 | 6 | 0 | -3.65997 | 4.212275 | 1.696973 |
| 35 | 6 | 0 | -1.87617 | 4.177823 | 0.115895 |
| 36 | 6 | 0 | -2.68497 | 4.930541 | 0.985104 |
| 37 | 6 | 0 | -4.86999 | -2.52609 | 1.111215 |
| 38 | 6 | 0 | -3.31169 | -2.91734 | -0.56838 |
| 39 | 6 | 0 | -5.26904 | -3.8511 | 1.093417 |
| 40 | 6 | 0 | -3.67243 | -4.25269 | -0.63434 |
| 41 | 6 | 0 | -4.68045 | -4.76483 | 0.202059 |
| 42 | 6 | 0 | 4.956495 | -2.11839 | 1.189671 |
| 43 | 6 | 0 | 2.792303 | -2.75104 | 0.657155 |
| 44 | 6 | 0 | 5.238383 | -3.37972 | 1.690061 |
| 45 | 6 | 0 | 3.008258 | -4.02976 | 1.148288 |
| 46 | 6 | 0 | 4.25911 | -4.38541 | 1.679429 |
| 47 | 6 | 0 | 3.553331 | 2.394995 | -2.20653 |
| 48 | 6 | 0 | 2.909597 | 3.212945 | -0.14351 |
| 49 | 6 | 0 | 3.606585 | 3.674783 | -2.73723 |
| 50 | 6 | 0 | 2.935335 | 4.51923 | -0.61069 |
| 51 | 6 | 0 | 3.306123 | 4.789425 | -1.93802 |
| 52 | 6 | 0 | -3.38597 | -0.11642 | 3.437431 |
| 53 | 6 | 0 | -1.45326 | -1.0147 | 2.514586 |
| 54 | 6 | 0 | -2.97216 | -0.45039 | 4.715839 |
| 55 | 6 | 0 | -0.98678 | -1.38175 | 3.76669 |
| 56 | 6 | 0 | -1.74546 | -1.10664 | 4.918497 |
| 57 | 6 | 0 | 5.466345 | 1.759355 | 1.452421 |
| 58 | 6 | 0 | 3.902921 | 0.742313 | 2.832719 |
| 59 | 6 | 0 | 6.138783 | 2.279843 | 2.547567 |
| 60 | 6 | 0 | 4.528378 | 1.239182 | 3.967047 |
| 61 | 6 | 0 | 5.68324 | 2.030304 | 3.852387 |
| 62 | 7 | 0 | 3.214101 | 2.147431 | -0.91887 |
| 63 | 7 | 0 | 3.74568 | -1.79262 | 0.670202 |
| 64 | 7 | 0 | 4.350933 | 0.999243 | 1.581158 |
| 65 | 7 | 0 | -2.63873 | -0.38454 | 2.335705 |
| 66 | 7 | 0 | -3.02196 | 2.13402 | 0.66128 |
| 67 | 7 | 0 | -3.90147 | -2.04432 | 0.286174 |
| 68 | 1 | 0 | -6.93812 | 0.977134 | -4.80937 |
| 69 | 1 | 0 | -8.89529 | 1.233929 | -3.31475 |
| 70 | 1 | 0 | -8.70989 | 1.046818 | -0.85582 |

| | | | | | |
|---|---|---|---|---|---|
| 71 | 1 | 0 | -0.8937 | -0.25795 | -4.15648 |
| 72 | 1 | 0 | 3.016206 | -3.05745 | -5.7525 |
| 73 | 1 | 0 | 5.486917 | -3.30287 | -5.53628 |
| 74 | 1 | 0 | 6.69692 | -2.29522 | -3.61645 |
| 75 | 1 | 0 | -1.44437 | 2.232907 | -0.69132 |
| 76 | 1 | 0 | -4.5762 | 2.297377 | 2.034045 |
| 77 | 1 | 0 | -4.32525 | 4.717158 | 2.390569 |
| 78 | 1 | 0 | -1.10047 | 4.659852 | -0.47035 |
| 79 | 1 | 0 | -5.3291 | -1.81494 | 1.785201 |
| 80 | 1 | 0 | -2.53248 | -2.52163 | -1.20594 |
| 81 | 1 | 0 | -6.05193 | -4.16757 | 1.775588 |
| 82 | 1 | 0 | -3.16732 | -4.89917 | -1.34464 |
| 83 | 1 | 0 | 6.232019 | -3.57671 | 2.079873 |
| 84 | 1 | 0 | 2.203438 | -4.7567 | 1.106587 |
| 85 | 1 | 0 | 1.83541 | -2.47014 | 0.232264 |
| 86 | 1 | 0 | 5.714733 | -1.34567 | 1.175937 |
| 87 | 1 | 0 | 3.815309 | 1.535233 | -2.80953 |
| 88 | 1 | 0 | 2.678628 | 3.007775 | 0.896455 |
| 89 | 1 | 0 | 3.905052 | 3.802031 | -3.77307 |
| 90 | 1 | 0 | 2.699057 | 5.329601 | 0.071747 |
| 91 | 1 | 0 | -4.3478 | 0.349519 | 3.263625 |
| 92 | 1 | 0 | -0.88541 | -1.23233 | 1.617615 |
| 93 | 1 | 0 | -3.61729 | -0.21032 | 5.555307 |
| 94 | 1 | 0 | -0.03483 | -1.8966 | 3.846171 |
| 95 | 1 | 0 | 5.826087 | 1.91435 | 0.443528 |
| 96 | 1 | 0 | 3.029964 | 0.103214 | 2.909846 |
| 97 | 1 | 0 | 7.030956 | 2.874458 | 2.377662 |
| 98 | 1 | 0 | 4.123669 | 0.994927 | 4.943882 |
| 99 | 8 | 0 | 1.509777 | 0.275452 | 0.844023 |
| 100 | 1 | 0 | 0.716679 | -0.00815 | 0.287394 |
| 101 | 1 | 0 | 1.307926 | 1.135158 | 1.238555 |
| 102 | 6 | 0 | 4.548853 | -5.76699 | 2.189497 |
| 103 | 1 | 0 | 5.226133 | -6.28766 | 1.501899 |
| 104 | 1 | 0 | 5.051927 | -5.73125 | 3.160553 |
| 105 | 1 | 0 | 3.641745 | -6.36653 | 2.2864 |
| 106 | 6 | 0 | 6.411561 | 2.560612 | 5.052937 |
| 107 | 1 | 0 | 6.650329 | 3.621602 | 4.931711 |
| 108 | 1 | 0 | 5.834561 | 2.433511 | 5.970667 |
| 109 | 1 | 0 | 7.365446 | 2.034149 | 5.177748 |
| 110 | 6 | 0 | -1.29334 | -1.50757 | 6.290543 |
| 111 | 1 | 0 | -1.95179 | -2.28723 | 6.692507 |
| 112 | 1 | 0 | -0.27268 | -1.89399 | 6.291004 |
| 113 | 1 | 0 | -1.35189 | -0.66244 | 6.984239 |
| 114 | 6 | 0 | -5.12008 | -6.19568 | 0.139016 |
| 115 | 1 | 0 | -6.09086 | -6.26435 | -0.36871 |
| 116 | 1 | 0 | -4.41162 | -6.81904 | -0.40943 |
| 117 | 1 | 0 | -5.25958 | -6.61159 | 1.141212 |
| 118 | 6 | 0 | -2.5406 | 6.414745 | 1.134065 |
| 119 | 1 | 0 | -1.62801 | 6.788334 | 0.666418 |
| 120 | 1 | 0 | -3.39204 | 6.922125 | 0.663351 |
| 121 | 1 | 0 | -2.54578 | 6.707917 | 2.188469 |
| 122 | 6 | 0 | 3.416419 | 6.190128 | -2.46639 |
| 123 | 1 | 0 | 3.063953 | 6.25813 | -3.4992 |
| 124 | 1 | 0 | 2.86148 | 6.903419 | -1.85348 |
| 125 | 1 | 0 | 4.467568 | 6.504652 | -2.46739 |

| | | | | | |
|---|---|---|---|---|---|
| 126 | 1 | 0 | -3.98981 | 0.435595 | -4.55606 |
| 127 | 1 | 0 | 0.804537 | -1.95473 | -4.27219 |

**Table S38.** Cartesian coordinates for $Ru^{III}Ru^{IV}$-5pic-OH-A.

| Center number | Atomic number | Atomic type | Coordinates (Ångström) | | |
|---|---|---|---|---|---|
| | | | X | Y | Z |
| 1 | 6 | 0 | 4.445498 | -1.05515 | -1.79573 |
| 2 | 6 | 0 | 4.549401 | -1.33611 | -3.17261 |
| 3 | 6 | 0 | 5.79235 | -1.64243 | -3.72628 |
| 4 | 6 | 0 | 6.883644 | -1.63713 | -2.84891 |
| 5 | 6 | 0 | 6.763139 | -1.33562 | -1.47739 |
| 6 | 6 | 0 | 5.523981 | -1.03252 | -0.90849 |
| 7 | 6 | 0 | 2.426391 | -0.87431 | -2.62461 |
| 8 | 6 | 0 | 5.369715 | -0.6752 | 0.55573 |
| 9 | 8 | 0 | 6.366497 | -0.5978 | 1.255225 |
| 10 | 8 | 0 | 4.152818 | -0.46267 | 1.069658 |
| 11 | 7 | 0 | 3.138864 | -0.79834 | -1.5112 |
| 12 | 7 | 0 | 3.243884 | -1.2093 | -3.673 |
| 13 | 6 | 0 | -0.07453 | -0.51928 | -3.29605 |
| 14 | 6 | 0 | -1.13407 | -0.19273 | -2.43917 |
| 15 | 7 | 0 | 0.663195 | -0.24673 | -1.14996 |
| 16 | 7 | 0 | -0.6708 | -0.06041 | -1.14646 |
| 17 | 6 | 0 | 1.032246 | -0.56301 | -2.46436 |
| 18 | 6 | 0 | -4.52713 | 0.269483 | -1.82173 |
| 19 | 6 | 0 | -4.59536 | 0.091705 | -3.23781 |
| 20 | 6 | 0 | -5.86903 | 0.11119 | -3.83714 |
| 21 | 6 | 0 | -6.97154 | 0.2858 | -3.00297 |
| 22 | 6 | 0 | -6.86143 | 0.431714 | -1.59546 |
| 23 | 6 | 0 | -5.62014 | 0.421283 | -0.96221 |
| 24 | 6 | 0 | -2.5519 | -0.01532 | -2.64718 |
| 25 | 7 | 0 | -3.22443 | 0.217597 | -1.48641 |
| 26 | 7 | 0 | -3.31965 | -0.09183 | -3.74137 |
| 27 | 6 | 0 | -5.46584 | 0.532484 | 0.535057 |
| 28 | 8 | 0 | -6.45258 | 0.699763 | 1.237047 |
| 29 | 8 | 0 | -4.25419 | 0.442026 | 1.10086 |
| 30 | 44 | 0 | -2.36926 | 0.168169 | 0.361176 |
| 31 | 44 | 0 | 2.328876 | -0.18395 | 0.205916 |
| 32 | 6 | 0 | -2.69703 | 2.91502 | 1.501781 |
| 33 | 6 | 0 | -1.88966 | 3.053435 | -0.66392 |
| 34 | 6 | 0 | -2.78623 | 4.29791 | 1.592048 |
| 35 | 6 | 0 | -1.95728 | 4.44072 | -0.64057 |
| 36 | 6 | 0 | -2.41979 | 5.102071 | 0.504661 |
| 37 | 6 | 0 | -3.40211 | -2.36162 | 1.585531 |
| 38 | 6 | 0 | -2.64796 | -2.8077 | -0.55551 |
| 39 | 6 | 0 | -3.83801 | -3.67143 | 1.74076 |
| 40 | 6 | 0 | -3.06209 | -4.13051 | -0.46723 |
| 41 | 6 | 0 | -3.68376 | -4.59521 | 0.698731 |
| 42 | 6 | 0 | 2.642544 | -3.18773 | 0.617751 |
| 43 | 6 | 0 | 0.659302 | -2.37218 | 1.501761 |
| 44 | 6 | 0 | 2.355499 | -4.47279 | 1.053577 |

| | | | | | |
|---|---|---|---|---|---|
| 45 | 6 | 0 | 0.315389 | -3.63843 | 1.960393 |
| 46 | 6 | 0 | 1.166641 | -4.72946 | 1.74962 |
| 47 | 6 | 0 | 3.799055 | 2.454666 | 0.472745 |
| 48 | 6 | 0 | 2.418576 | 2.481218 | -1.38401 |
| 49 | 6 | 0 | 4.240093 | 3.742487 | 0.205092 |
| 50 | 6 | 0 | 2.816859 | 3.771992 | -1.706 |
| 51 | 6 | 0 | 3.755455 | 4.440324 | -0.90953 |
| 52 | 6 | 0 | 2.253802 | 0.069025 | 3.237356 |
| 53 | 6 | 0 | 0.847687 | 1.613195 | 2.251735 |
| 54 | 6 | 0 | 1.995301 | 0.584871 | 4.499185 |
| 55 | 6 | 0 | 0.56318 | 2.193609 | 3.480872 |
| 56 | 6 | 0 | 1.129605 | 1.676445 | 4.650637 |
| 57 | 7 | 0 | 2.898898 | 1.81747 | -0.31159 |
| 58 | 7 | 0 | 1.812903 | -2.14456 | 0.835357 |
| 59 | 7 | 0 | 1.688142 | 0.569535 | 2.116434 |
| 60 | 7 | 0 | -2.24737 | 2.295919 | 0.389915 |
| 61 | 7 | 0 | -2.81152 | -1.92998 | 0.451427 |
| 62 | 1 | 0 | -5.98903 | -0.01282 | -4.90874 |
| 63 | 1 | 0 | -7.96394 | 0.30165 | -3.44353 |
| 64 | 1 | 0 | -7.75197 | 0.546229 | -0.98625 |
| 65 | 1 | 0 | -0.14035 | -0.70544 | -4.35743 |
| 66 | 1 | 0 | 5.922067 | -1.86867 | -4.77935 |
| 67 | 1 | 0 | 7.866624 | -1.8684 | -3.24624 |
| 68 | 1 | 0 | 7.63838 | -1.32779 | -0.83697 |
| 69 | 1 | 0 | -1.55753 | 2.519963 | -1.54621 |
| 70 | 1 | 0 | -3.00531 | 2.263652 | 2.310238 |
| 71 | 1 | 0 | -3.16414 | 4.742301 | 2.507545 |
| 72 | 1 | 0 | -1.66451 | 5.000204 | -1.52351 |
| 73 | 1 | 0 | -3.53441 | -1.6176 | 2.361144 |
| 74 | 1 | 0 | -2.18089 | -2.42959 | -1.45604 |
| 75 | 1 | 0 | -4.31674 | -3.95909 | 2.671589 |
| 76 | 1 | 0 | -2.90898 | -4.78956 | -1.31601 |
| 77 | 1 | 0 | 3.065192 | -5.2686 | 0.850813 |
| 78 | 1 | 0 | -0.62689 | -3.76119 | 2.484556 |
| 79 | 1 | 0 | 0.006099 | -1.51832 | 1.667152 |
| 80 | 1 | 0 | 3.560924 | -2.97343 | 0.08677 |
| 81 | 1 | 0 | 4.172181 | 1.890964 | 1.31914 |
| 82 | 1 | 0 | 1.689166 | 1.955768 | -1.98791 |
| 83 | 1 | 0 | 4.96842 | 4.195254 | 0.870341 |
| 84 | 1 | 0 | 2.391772 | 4.251609 | -2.58201 |
| 85 | 1 | 0 | 2.955354 | -0.74317 | 3.093227 |
| 86 | 1 | 0 | 0.395501 | 1.987807 | 1.34299 |
| 87 | 1 | 0 | 2.48339 | 0.139442 | 5.360293 |
| 88 | 1 | 0 | -0.11855 | 3.036796 | 3.518034 |
| 89 | 8 | 0 | -1.46168 | 0.014014 | 1.915224 |
| 90 | 6 | 0 | -2.55655 | 6.600306 | 0.553048 |
| 91 | 1 | 0 | -1.8922 | 7.089612 | -0.16371 |
| 92 | 1 | 0 | -3.58352 | 6.893717 | 0.304611 |
| 93 | 1 | 0 | -2.34111 | 6.989544 | 1.551949 |
| 94 | 6 | 0 | -4.19668 | -6.00608 | 0.812152 |
| 95 | 1 | 0 | -5.23959 | -6.05606 | 0.476819 |
| 96 | 1 | 0 | -3.6204 | -6.69569 | 0.189603 |
| 97 | 1 | 0 | -4.17124 | -6.36111 | 1.845903 |
| 98 | 6 | 0 | 0.83809 | -6.10379 | 2.267374 |
| 99 | 1 | 0 | 1.242197 | -6.88442 | 1.617302 |

| | | | | | |
|---|---|---|---|---|---|
| 100 | 1 | 0 | 1.275363 | -6.24925 | 3.262622 |
| 101 | 1 | 0 | -0.2412 | -6.24896 | 2.359567 |
| 102 | 6 | 0 | 0.806312 | 2.244967 | 6.006215 |
| 103 | 1 | 0 | 0.533703 | 3.301717 | 5.94404 |
| 104 | 1 | 0 | -0.0446 | 1.71141 | 6.446746 |
| 105 | 1 | 0 | 1.648849 | 2.142349 | 6.695292 |
| 106 | 6 | 0 | 4.238858 | 5.825346 | -1.24313 |
| 107 | 1 | 0 | 5.206562 | 5.775561 | -1.75664 |
| 108 | 1 | 0 | 3.541391 | 6.348242 | -1.90175 |
| 109 | 1 | 0 | 4.383223 | 6.424705 | -0.33993 |
| 110 | 1 | 0 | 2.952805 | -1.34653 | -4.62917 |

**Table S39.** Cartesian coordinates for $Ru^{III}Ru^{IV}$-5pic-$OH_2$-A.

| Center number | Atomic number | Atomic type | Coordinates (Ångström) | | |
|---|---|---|---|---|---|
| | | | X | Y | Z |
| 1 | 6 | 0 | 4.395291 | -0.94855 | -1.92409 |
| 2 | 6 | 0 | 4.477428 | -1.15302 | -3.31364 |
| 3 | 6 | 0 | 5.714967 | -1.39563 | -3.91288 |
| 4 | 6 | 0 | 6.827072 | -1.4024 | -3.06476 |
| 5 | 6 | 0 | 6.732852 | -1.17334 | -1.67609 |
| 6 | 6 | 0 | 5.498323 | -0.94353 | -1.06694 |
| 7 | 6 | 0 | 2.35874 | -0.76812 | -2.7025 |
| 8 | 6 | 0 | 5.353167 | -0.66866 | 0.403801 |
| 9 | 8 | 0 | 6.322568 | -0.46482 | 1.105418 |
| 10 | 8 | 0 | 4.115333 | -0.70538 | 0.950795 |
| 11 | 7 | 0 | 3.08756 | -0.72999 | -1.59735 |
| 12 | 7 | 0 | 3.159829 | -1.02981 | -3.7798 |
| 13 | 6 | 0 | -0.15488 | -0.40318 | -3.35299 |
| 14 | 6 | 0 | -1.20492 | -0.11685 | -2.47838 |
| 15 | 7 | 0 | 0.589936 | -0.2462 | -1.20602 |
| 16 | 7 | 0 | -0.73733 | -0.04883 | -1.18223 |
| 17 | 6 | 0 | 0.956996 | -0.4883 | -2.51795 |
| 18 | 6 | 0 | -4.62086 | 0.432374 | -1.78633 |
| 19 | 6 | 0 | -4.78723 | 0.343012 | -3.18123 |
| 20 | 6 | 0 | -6.06039 | 0.46005 | -3.74378 |
| 21 | 6 | 0 | -7.11293 | 0.655648 | -2.84539 |
| 22 | 6 | 0 | -6.92896 | 0.726928 | -1.44643 |
| 23 | 6 | 0 | -5.6629 | 0.611875 | -0.87421 |
| 24 | 6 | 0 | -2.63102 | 0.10036 | -2.62693 |
| 25 | 7 | 0 | -3.2955 | 0.291569 | -1.50059 |
| 26 | 7 | 0 | -3.49458 | 0.128545 | -3.68886 |
| 27 | 6 | 0 | -5.44928 | 0.660358 | 0.626975 |
| 28 | 8 | 0 | -6.41085 | 0.845798 | 1.351774 |
| 29 | 8 | 0 | -4.22938 | 0.495848 | 1.143917 |
| 30 | 44 | 0 | -2.37311 | 0.188699 | 0.358198 |
| 31 | 44 | 0 | 2.32996 | -0.24376 | 0.209651 |
| 32 | 6 | 0 | -2.67488 | 2.928492 | 1.54151 |
| 33 | 6 | 0 | -1.71814 | 3.094138 | -0.56039 |
| 34 | 6 | 0 | -2.69749 | 4.310051 | 1.675077 |
| 35 | 6 | 0 | -1.71578 | 4.480884 | -0.49234 |

| | | | | | |
|---|---|---|---|---|---|
| 36 | 6 | 0 | -2.22032 | 5.13055 | 0.64339 |
| 37 | 6 | 0 | -3.54 | -2.32069 | 1.524624 |
| 38 | 6 | 0 | -2.6288 | -2.80202 | -0.54708 |
| 39 | 6 | 0 | -3.98954 | -3.6269 | 1.666111 |
| 40 | 6 | 0 | -3.05459 | -4.12115 | -0.4717 |
| 41 | 6 | 0 | -3.76557 | -4.56762 | 0.651407 |
| 42 | 6 | 0 | 2.58709 | -3.27838 | 0.413167 |
| 43 | 6 | 0 | 0.635604 | -2.49647 | 1.389398 |
| 44 | 6 | 0 | 2.299333 | -4.58196 | 0.787906 |
| 45 | 6 | 0 | 0.293681 | -3.78084 | 1.795595 |
| 46 | 6 | 0 | 1.129953 | -4.86673 | 1.507486 |
| 47 | 6 | 0 | 3.844498 | 2.341255 | 0.681364 |
| 48 | 6 | 0 | 2.552755 | 2.491189 | -1.23337 |
| 49 | 6 | 0 | 4.345839 | 3.61888 | 0.484139 |
| 50 | 6 | 0 | 3.01156 | 3.776786 | -1.48538 |
| 51 | 6 | 0 | 3.939252 | 4.377909 | -0.62309 |
| 52 | 6 | 0 | 2.3082 | -0.28244 | 3.25308 |
| 53 | 6 | 0 | 0.979952 | 1.42401 | 2.430759 |
| 54 | 6 | 0 | 2.087078 | 0.127991 | 4.558962 |
| 55 | 6 | 0 | 0.728453 | 1.89442 | 3.711924 |
| 56 | 6 | 0 | 1.278538 | 1.243304 | 4.823052 |
| 57 | 7 | 0 | 2.956714 | 1.768606 | -0.16707 |
| 58 | 7 | 0 | 1.773103 | -2.24021 | 0.707501 |
| 59 | 7 | 0 | 1.761187 | 0.351405 | 2.190323 |
| 60 | 7 | 0 | -2.18968 | 2.31986 | 0.436493 |
| 61 | 7 | 0 | -2.86907 | -1.90414 | 0.427978 |
| 62 | 1 | 0 | -6.23787 | 0.400947 | -4.8122 |
| 63 | 1 | 0 | -8.11879 | 0.750861 | -3.24111 |
| 64 | 1 | 0 | -7.77953 | 0.869096 | -0.78859 |
| 65 | 1 | 0 | -0.1961 | -0.53662 | -4.42401 |
| 66 | 1 | 0 | 5.824024 | -1.56433 | -4.97884 |
| 67 | 1 | 0 | 7.805708 | -1.58421 | -3.49627 |
| 68 | 1 | 0 | 7.625701 | -1.17014 | -1.06018 |
| 69 | 1 | 0 | -1.3388 | 2.576057 | -1.43323 |
| 70 | 1 | 0 | -3.07026 | 2.273637 | 2.308129 |
| 71 | 1 | 0 | -3.1074 | 4.74241 | 2.582357 |
| 72 | 1 | 0 | -1.33059 | 5.052574 | -1.33075 |
| 73 | 1 | 0 | -3.72908 | -1.56898 | 2.280883 |
| 74 | 1 | 0 | -2.07589 | -2.44571 | -1.40769 |
| 75 | 1 | 0 | -4.5307 | -3.89974 | 2.566534 |
| 76 | 1 | 0 | -2.835 | -4.79643 | -1.29261 |
| 77 | 1 | 0 | 2.99699 | -5.37087 | 0.525283 |
| 78 | 1 | 0 | -0.63056 | -3.92336 | 2.346229 |
| 79 | 1 | 0 | -0.00195 | -1.64677 | 1.614788 |
| 80 | 1 | 0 | 3.497319 | -3.0442 | -0.12319 |
| 81 | 1 | 0 | 4.163733 | 1.737928 | 1.522379 |
| 82 | 1 | 0 | 1.838658 | 2.014451 | -1.89378 |
| 83 | 1 | 0 | 5.062666 | 4.014915 | 1.196338 |
| 84 | 1 | 0 | 2.648668 | 4.305306 | -2.36112 |
| 85 | 1 | 0 | 2.962982 | -1.11481 | 3.02774 |
| 86 | 1 | 0 | 0.548693 | 1.912905 | 1.56626 |
| 87 | 1 | 0 | 2.56075 | -0.41794 | 5.36851 |
| 88 | 1 | 0 | 0.095183 | 2.766384 | 3.837532 |
| 89 | 8 | 0 | -1.41815 | -0.00911 | 1.858203 |
| 90 | 6 | 0 | -2.2796 | 6.630167 | 0.739436 |

| | | | | | |
|---|---|---|---|---|---|
| 91 | 1 | 0 | -1.57367 | 7.108323 | 0.056332 |
| 92 | 1 | 0 | -3.28423 | 6.984033 | 0.478454 |
| 93 | 1 | 0 | -2.0719 | 6.973622 | 1.756436 |
| 94 | 6 | 0 | -4.28823 | -5.97452 | 0.750606 |
| 95 | 1 | 0 | -5.32332 | -6.0165 | 0.390579 |
| 96 | 1 | 0 | -3.70187 | -6.66798 | 0.142708 |
| 97 | 1 | 0 | -4.29272 | -6.32875 | 1.784736 |
| 98 | 6 | 0 | 0.810806 | -6.26265 | 1.967051 |
| 99 | 1 | 0 | 1.091908 | -7.00504 | 1.214965 |
| 100 | 1 | 0 | 1.372869 | -6.49834 | 2.878714 |
| 101 | 1 | 0 | -0.25108 | -6.37873 | 2.195662 |
| 102 | 6 | 0 | 1.003436 | 1.701449 | 6.228543 |
| 103 | 1 | 0 | 0.735559 | 2.760149 | 6.261965 |
| 104 | 1 | 0 | 0.165252 | 1.134883 | 6.652056 |
| 105 | 1 | 0 | 1.867335 | 1.538168 | 6.878071 |
| 106 | 6 | 0 | 4.49088 | 5.752546 | -0.87842 |
| 107 | 1 | 0 | 5.513345 | 5.682253 | -1.26831 |
| 108 | 1 | 0 | 3.892508 | 6.300781 | -1.60923 |
| 109 | 1 | 0 | 4.540072 | 6.337346 | 0.044578 |
| 110 | 1 | 0 | 2.856719 | -1.12314 | -4.73814 |
| 111 | 1 | 0 | -3.25106 | 0.019832 | -4.66239 |

**Table S40.** Cartesian coordinates for $Ru^{III}Ru^{IV}$-5pic-OH-$OH_2$-A.

| Center number | Atomic number | Atomic type | Coordinates (Ångström) | | |
|---|---|---|---|---|---|
| | | | X | Y | Z |
| 1 | 6 | 0 | -3.62204 | -0.16158 | -2.77504 |
| 2 | 6 | 0 | -3.1085 | -0.51507 | -4.05439 |
| 3 | 6 | 0 | -3.96135 | -0.67353 | -5.15611 |
| 4 | 6 | 0 | -5.32421 | -0.47293 | -4.96413 |
| 5 | 6 | 0 | -5.83101 | -0.1018 | -3.70666 |
| 6 | 6 | 0 | -5.00274 | 0.075206 | -2.59372 |
| 7 | 6 | 0 | -1.46752 | -0.3992 | -2.70179 |
| 8 | 6 | 0 | -5.60326 | 0.563431 | -1.32415 |
| 9 | 8 | 0 | -6.80941 | 0.609587 | -1.13287 |
| 10 | 8 | 0 | -4.7475 | 1.023694 | -0.39834 |
| 11 | 7 | 0 | -2.55756 | -0.10467 | -1.89481 |
| 12 | 7 | 0 | -1.7466 | -0.65143 | -3.9768 |
| 13 | 6 | 0 | 1.072446 | -0.57273 | -3.08823 |
| 14 | 6 | 0 | 2.143187 | -0.55064 | -2.18666 |
| 15 | 7 | 0 | 0.336682 | -0.33663 | -1.00254 |
| 16 | 7 | 0 | 1.665326 | -0.40338 | -0.91098 |
| 17 | 6 | 0 | -0.07368 | -0.4348 | -2.2876 |
| 18 | 6 | 0 | 5.523925 | -0.56896 | -1.18141 |
| 19 | 6 | 0 | 5.806152 | -0.74206 | -2.55021 |
| 20 | 6 | 0 | 7.130043 | -0.83682 | -2.9826 |
| 21 | 6 | 0 | 8.110466 | -0.74433 | -1.98936 |
| 22 | 6 | 0 | 7.806853 | -0.5609 | -0.62323 |
| 23 | 6 | 0 | 6.488415 | -0.46474 | -0.17691 |
| 24 | 6 | 0 | 3.594356 | -0.62132 | -2.21231 |
| 25 | 7 | 0 | 4.172905 | -0.50915 | -1.02579 |

| | | | | | |
|---|---|---|---|---|---|
| 26 | 7 | 0 | 4.5497 | -0.76995 | -3.18062 |
| 27 | 6 | 0 | 6.147584 | -0.24514 | 1.291794 |
| 28 | 8 | 0 | 7.070321 | -0.17905 | 2.090691 |
| 29 | 8 | 0 | 4.88501 | -0.11968 | 1.680894 |
| 30 | 44 | 0 | 3.059585 | -0.18831 | 0.707136 |
| 31 | 44 | 0 | -2.99868 | 0.253902 | 0.157081 |
| 32 | 6 | 0 | 3.492309 | -2.63917 | 2.376061 |
| 33 | 6 | 0 | 2.631467 | -3.23936 | 0.312712 |
| 34 | 6 | 0 | 3.528755 | -3.96559 | 2.78309 |
| 35 | 6 | 0 | 2.646508 | -4.58466 | 0.655628 |
| 36 | 6 | 0 | 3.105682 | -4.98262 | 1.917704 |
| 37 | 6 | 0 | 3.881436 | 2.62065 | 1.413226 |
| 38 | 6 | 0 | 2.851313 | 2.61164 | -0.65861 |
| 39 | 6 | 0 | 4.088537 | 3.991768 | 1.340994 |
| 40 | 6 | 0 | 3.031668 | 3.982043 | -0.79651 |
| 41 | 6 | 0 | 3.671792 | 4.711264 | 0.213766 |
| 42 | 6 | 0 | -2.57715 | 3.037425 | -0.95145 |
| 43 | 6 | 0 | -1.34732 | 2.727704 | 0.979743 |
| 44 | 6 | 0 | -2.13742 | 4.350408 | -1.06052 |
| 45 | 6 | 0 | -0.86658 | 4.029222 | 0.929507 |
| 46 | 6 | 0 | -1.26549 | 4.885744 | -0.10533 |
| 47 | 6 | 0 | -5.1223 | -1.90099 | 0.575719 |
| 48 | 6 | 0 | -3.08601 | -2.76607 | -0.10372 |
| 49 | 6 | 0 | -5.69296 | -3.16553 | 0.595444 |
| 50 | 6 | 0 | -3.59589 | -4.05756 | -0.10399 |
| 51 | 6 | 0 | -4.93241 | -4.2882 | 0.243799 |
| 52 | 6 | 0 | -4.49887 | 1.642262 | 2.429422 |
| 53 | 6 | 0 | -3.22154 | -0.12392 | 3.209185 |
| 54 | 6 | 0 | -4.95877 | 1.916398 | 3.711503 |
| 55 | 6 | 0 | -3.64388 | 0.096905 | 4.512819 |
| 56 | 6 | 0 | -4.53227 | 1.143179 | 4.798073 |
| 57 | 7 | 0 | -3.83349 | -1.69447 | 0.226804 |
| 58 | 7 | 0 | -2.19703 | 2.226841 | 0.059828 |
| 59 | 7 | 0 | -3.63066 | 0.640558 | 2.173093 |
| 60 | 7 | 0 | 3.048951 | -2.27504 | 1.154332 |
| 61 | 7 | 0 | 3.274597 | 1.931488 | 0.423474 |
| 62 | 1 | 0 | 7.397647 | -0.97228 | -4.02517 |
| 63 | 1 | 0 | 9.152588 | -0.8131 | -2.28471 |
| 64 | 1 | 0 | 8.601646 | -0.48738 | 0.111103 |
| 65 | 1 | 0 | 1.077896 | -0.67353 | -4.16194 |
| 66 | 1 | 0 | -3.55525 | -0.94315 | -6.12608 |
| 67 | 1 | 0 | -6.0097 | -0.58946 | -5.79785 |
| 68 | 1 | 0 | -6.89229 | 0.077584 | -3.5709 |
| 69 | 1 | 0 | 2.27235 | -2.9122 | -0.65534 |
| 70 | 1 | 0 | 3.835023 | -1.83431 | 3.014409 |
| 71 | 1 | 0 | 3.895229 | -4.19928 | 3.777601 |
| 72 | 1 | 0 | 2.298447 | -5.31798 | -0.06498 |
| 73 | 1 | 0 | 4.218414 | 2.03444 | 2.259645 |
| 74 | 1 | 0 | 2.350037 | 2.035805 | -1.4269 |
| 75 | 1 | 0 | 4.587012 | 4.490916 | 2.165944 |
| 76 | 1 | 0 | 2.674417 | 4.474026 | -1.69571 |
| 77 | 1 | 0 | -2.48564 | 4.951706 | -1.89438 |
| 78 | 1 | 0 | -0.18583 | 4.368859 | 1.703673 |
| 79 | 1 | 0 | -1.04618 | 2.061879 | 1.779296 |
| 80 | 1 | 0 | -3.2608 | 2.60848 | -1.67208 |

| | | | | | |
|---|---|---|---|---|---|
| 81 | 1 | 0 | -5.70304 | -1.01995 | 0.820673 |
| 82 | 1 | 0 | -2.05572 | -2.56208 | -0.36299 |
| 83 | 1 | 0 | -6.73654 | -3.26552 | 0.876133 |
| 84 | 1 | 0 | -2.94677 | -4.88141 | -0.38316 |
| 85 | 1 | 0 | -4.84537 | 2.196194 | 1.565174 |
| 86 | 1 | 0 | -2.54399 | -0.93334 | 2.96146 |
| 87 | 1 | 0 | -5.66152 | 2.73137 | 3.853383 |
| 88 | 1 | 0 | -3.28273 | -0.55519 | 5.302006 |
| 89 | 8 | 0 | 1.881406 | 0.116359 | 2.027674 |
| 90 | 6 | 0 | 3.167807 | -6.43239 | 2.317141 |
| 91 | 1 | 0 | 2.481791 | -7.04295 | 1.724862 |
| 92 | 1 | 0 | 4.17967 | -6.82572 | 2.162168 |
| 93 | 1 | 0 | 2.927688 | -6.56417 | 3.375742 |
| 94 | 6 | 0 | 3.92607 | 6.189549 | 0.0849 |
| 95 | 1 | 0 | 4.941934 | 6.368619 | -0.28734 |
| 96 | 1 | 0 | 3.23248 | 6.658935 | -0.61758 |
| 97 | 1 | 0 | 3.843302 | 6.695271 | 1.050956 |
| 98 | 6 | 0 | -0.80373 | 6.31727 | -0.16951 |
| 99 | 1 | 0 | -0.78355 | 6.68626 | -1.19831 |
| 100 | 1 | 0 | -1.48623 | 6.963653 | 0.395388 |
| 101 | 1 | 0 | 0.192128 | 6.436707 | 0.265993 |
| 102 | 6 | 0 | -4.99205 | 1.427086 | 6.202877 |
| 103 | 1 | 0 | -5.08854 | 0.506607 | 6.785071 |
| 104 | 1 | 0 | -4.26612 | 2.065728 | 6.720724 |
| 105 | 1 | 0 | -5.95327 | 1.946773 | 6.212223 |
| 106 | 6 | 0 | -5.534 | -5.66749 | 0.21557 |
| 107 | 1 | 0 | -6.10759 | -5.81184 | -0.70778 |
| 108 | 1 | 0 | -4.76537 | -6.44329 | 0.253 |
| 109 | 1 | 0 | -6.22308 | -5.81876 | 1.051346 |
| 110 | 8 | 0 | -1.27916 | -0.55346 | 0.906211 |
| 111 | 1 | 0 | -0.86949 | -0.07999 | 1.645657 |
| 112 | 1 | 0 | -0.31136 | -0.40717 | -0.11308 |
| 113 | 1 | 0 | 4.378057 | -0.88535 | -4.16836 |

**Table S41.** Cartesian coordinates for $Ru^{III}Ru^{IV}$-5pic-2$OH_2$-A.

| Center number | Atomic number | Atomic type | Coordinates (Ångström) | | |
|---|---|---|---|---|---|
| | | | X | Y | Z |
| 1 | 6 | 0 | -3.67069 | -0.24345 | -2.80255 |
| 2 | 6 | 0 | -3.29088 | -0.62919 | -4.10399 |
| 3 | 6 | 0 | -4.19945 | -0.81717 | -5.14536 |
| 4 | 6 | 0 | -5.54151 | -0.60252 | -4.84783 |
| 5 | 6 | 0 | -5.94313 | -0.19893 | -3.56446 |
| 6 | 6 | 0 | -5.03017 | 0.002322 | -2.52551 |
| 7 | 6 | 0 | -1.49233 | -0.47699 | -2.78709 |
| 8 | 6 | 0 | -5.54654 | 0.517026 | -1.22466 |
| 9 | 8 | 0 | -6.73263 | 0.542265 | -0.95988 |
| 10 | 8 | 0 | -4.64596 | 1.025945 | -0.36428 |
| 11 | 7 | 0 | -2.5349 | -0.17285 | -1.98772 |
| 12 | 7 | 0 | -1.91521 | -0.75671 | -4.05122 |
| 13 | 6 | 0 | 1.078162 | -0.65108 | -3.24618 |

| | | | | | |
|---|---|---|---|---|---|
| 14 | 6 | 0 | 2.13194 | -0.59913 | -2.32332 |
| 15 | 7 | 0 | 0.282806 | -0.39933 | -1.15588 |
| 16 | 7 | 0 | 1.59574 | -0.43584 | -1.06767 |
| 17 | 6 | 0 | -0.0808 | -0.51696 | -2.44956 |
| 18 | 6 | 0 | 5.444097 | -0.60763 | -1.10833 |
| 19 | 6 | 0 | 5.816499 | -0.79727 | -2.45258 |
| 20 | 6 | 0 | 7.166394 | -0.90365 | -2.79547 |
| 21 | 6 | 0 | 8.076921 | -0.80589 | -1.73966 |
| 22 | 6 | 0 | 7.683583 | -0.60669 | -0.39725 |
| 23 | 6 | 0 | 6.339856 | -0.49863 | -0.04126 |
| 24 | 6 | 0 | 3.585601 | -0.66705 | -2.2695 |
| 25 | 7 | 0 | 4.085685 | -0.54091 | -1.04548 |
| 26 | 7 | 0 | 4.605068 | -0.82867 | -3.1669 |
| 27 | 6 | 0 | 5.90495 | -0.26644 | 1.398695 |
| 28 | 8 | 0 | 6.764122 | -0.19519 | 2.260255 |
| 29 | 8 | 0 | 4.613425 | -0.13613 | 1.701281 |
| 30 | 44 | 0 | 2.874791 | -0.21364 | 0.613814 |
| 31 | 44 | 0 | -2.89952 | 0.289215 | 0.148682 |
| 32 | 6 | 0 | 3.204369 | -2.63642 | 2.34193 |
| 33 | 6 | 0 | 2.588016 | -3.28751 | 0.207284 |
| 34 | 6 | 0 | 3.27275 | -3.95788 | 2.760047 |
| 35 | 6 | 0 | 2.64201 | -4.62885 | 0.559548 |
| 36 | 6 | 0 | 2.996548 | -4.99964 | 1.864323 |
| 37 | 6 | 0 | 3.585008 | 2.580955 | 1.446762 |
| 38 | 6 | 0 | 2.995378 | 2.582771 | -0.79087 |
| 39 | 6 | 0 | 3.882861 | 3.936297 | 1.403395 |
| 40 | 6 | 0 | 3.281489 | 3.937028 | -0.90514 |
| 41 | 6 | 0 | 3.750964 | 4.652295 | 0.205388 |
| 42 | 6 | 0 | -2.6292 | 3.139607 | -0.90767 |
| 43 | 6 | 0 | -1.03659 | 2.686218 | 0.706793 |
| 44 | 6 | 0 | -2.179 | 4.445428 | -1.03584 |
| 45 | 6 | 0 | -0.53741 | 3.979399 | 0.626721 |
| 46 | 6 | 0 | -1.11094 | 4.906131 | -0.25354 |
| 47 | 6 | 0 | -5.00128 | -1.80396 | 0.863408 |
| 48 | 6 | 0 | -3.05586 | -2.76154 | 0.048231 |
| 49 | 6 | 0 | -5.5879 | -3.0499 | 1.025222 |
| 50 | 6 | 0 | -3.58445 | -4.03672 | 0.188993 |
| 51 | 6 | 0 | -4.88444 | -4.21372 | 0.683076 |
| 52 | 6 | 0 | -4.20719 | 1.840198 | 2.395505 |
| 53 | 6 | 0 | -3.00631 | 0.021373 | 3.185709 |
| 54 | 6 | 0 | -4.58526 | 2.18945 | 3.684387 |
| 55 | 6 | 0 | -3.3483 | 0.319972 | 4.496375 |
| 56 | 6 | 0 | -4.15498 | 1.431462 | 4.781914 |
| 57 | 7 | 0 | -3.74846 | -1.64991 | 0.374084 |
| 58 | 7 | 0 | -2.07546 | 2.259161 | -0.04276 |
| 59 | 7 | 0 | -3.41804 | 0.773216 | 2.139285 |
| 60 | 7 | 0 | 2.86565 | -2.29855 | 1.078752 |
| 61 | 7 | 0 | 3.150157 | 1.903829 | 0.361497 |
| 62 | 1 | 0 | 7.50344 | -1.0521 | -3.81571 |
| 63 | 1 | 0 | 9.136031 | -0.88369 | -1.96264 |
| 64 | 1 | 0 | 8.429704 | -0.53142 | 0.386389 |
| 65 | 1 | 0 | 1.150868 | -0.74983 | -4.31958 |
| 66 | 1 | 0 | -3.87778 | -1.11505 | -6.13791 |
| 67 | 1 | 0 | -6.28912 | -0.73673 | -5.62211 |
| 68 | 1 | 0 | -6.99018 | -0.01431 | -3.35068 |

| | | | | | |
|---|---|---|---|---|---|
| 69 | 1 | 0 | 2.318017 | -2.98481 | -0.7974 |
| 70 | 1 | 0 | 3.442155 | -1.81516 | 3.006391 |
| 71 | 1 | 0 | 3.554385 | -4.16885 | 3.786663 |
| 72 | 1 | 0 | 2.414045 | -5.38254 | -0.18754 |
| 73 | 1 | 0 | 3.717187 | 1.998013 | 2.349764 |
| 74 | 1 | 0 | 2.632903 | 2.017788 | -1.64139 |
| 75 | 1 | 0 | 4.239053 | 4.423746 | 2.305394 |
| 76 | 1 | 0 | 3.14853 | 4.427345 | -1.86437 |
| 77 | 1 | 0 | -2.67321 | 5.103207 | -1.7438 |
| 78 | 1 | 0 | 0.299567 | 4.255706 | 1.259642 |
| 79 | 1 | 0 | -0.58612 | 1.970887 | 1.382819 |
| 80 | 1 | 0 | -3.46924 | 2.776295 | -1.48475 |
| 81 | 1 | 0 | -5.53962 | -0.89721 | 1.110227 |
| 82 | 1 | 0 | -2.04919 | -2.60835 | -0.31905 |
| 83 | 1 | 0 | -6.59889 | -3.10536 | 1.415772 |
| 84 | 1 | 0 | -2.97766 | -4.89276 | -0.08777 |
| 85 | 1 | 0 | -4.5584 | 2.391022 | 1.532509 |
| 86 | 1 | 0 | -2.39603 | -0.84183 | 2.947899 |
| 87 | 1 | 0 | -5.22769 | 3.052731 | 3.824897 |
| 88 | 1 | 0 | -2.9886 | -0.32348 | 5.292824 |
| 89 | 8 | 0 | 1.542759 | 0.092524 | 1.796771 |
| 90 | 6 | 0 | 3.106269 | -6.44188 | 2.276615 |
| 91 | 1 | 0 | 2.516936 | -7.094 | 1.627586 |
| 92 | 1 | 0 | 4.150338 | -6.77211 | 2.217282 |
| 93 | 1 | 0 | 2.780315 | -6.58716 | 3.310002 |
| 94 | 6 | 0 | 4.128179 | 6.105902 | 0.112156 |
| 95 | 1 | 0 | 5.210961 | 6.204358 | -0.03091 |
| 96 | 1 | 0 | 3.640505 | 6.598693 | -0.7326 |
| 97 | 1 | 0 | 3.876532 | 6.645237 | 1.02946 |
| 98 | 6 | 0 | -0.6292 | 6.328188 | -0.33734 |
| 99 | 1 | 0 | -0.69587 | 6.713868 | -1.35815 |
| 100 | 1 | 0 | -1.25022 | 6.975185 | 0.293896 |
| 101 | 1 | 0 | 0.402471 | 6.42439 | 0.009113 |
| 102 | 6 | 0 | -4.52692 | 1.797958 | 6.191583 |
| 103 | 1 | 0 | -4.57682 | 0.916418 | 6.835414 |
| 104 | 1 | 0 | -3.77411 | 2.472608 | 6.617445 |
| 105 | 1 | 0 | -5.4887 | 2.315032 | 6.230116 |
| 106 | 6 | 0 | -5.50459 | -5.57592 | 0.822688 |
| 107 | 1 | 0 | -6.21615 | -5.75255 | 0.00725 |
| 108 | 1 | 0 | -4.7536 | -6.36808 | 0.787709 |
| 109 | 1 | 0 | -6.06194 | -5.6632 | 1.759631 |
| 110 | 8 | 0 | -1.12045 | -0.53771 | 0.88649 |
| 111 | 1 | 0 | -0.54304 | -0.14642 | 1.572434 |
| 112 | 1 | 0 | -0.48724 | -0.53223 | 0.033995 |
| 113 | 1 | 0 | 4.508641 | -0.95608 | -4.16358 |
| 114 | 1 | 0 | -1.31575 | -1.04483 | -4.81006 |

**Table S42.** Cartesian coordinates for Ru$^{III}$Ru$^{IV}$-4pic-2OH-A.

| Center number | Atomic number | Atomic type | Coordinates (Ångström) | | |
|---|---|---|---|---|---|
| | | | X | Y | Z |

| | | | | | |
|---|---|---|---|---|---|
| 1 | 6 | 0 | 4.542095 | 0.398502 | 1.417653 |
| 2 | 6 | 0 | 4.671873 | 0.44147 | 2.82931 |
| 3 | 6 | 0 | 5.958939 | 0.515697 | 3.363935 |
| 4 | 6 | 0 | 7.037608 | 0.531039 | 2.464293 |
| 5 | 6 | 0 | 6.868043 | 0.465841 | 1.069089 |
| 6 | 6 | 0 | 5.590988 | 0.391391 | 0.496959 |
| 7 | 6 | 0 | 2.575057 | 0.298609 | 2.400222 |
| 8 | 6 | 0 | 5.378934 | 0.293619 | -1.00188 |
| 9 | 8 | 0 | 6.357727 | 0.337945 | -1.74165 |
| 10 | 8 | 0 | 4.149011 | 0.168397 | -1.48855 |
| 11 | 7 | 0 | 3.210326 | 0.32202 | 1.16659 |
| 12 | 7 | 0 | 3.400681 | 0.374069 | 3.426825 |
| 13 | 6 | 0 | 0.038664 | -0.00424 | 3.105733 |
| 14 | 6 | 0 | -1.05673 | -0.14146 | 2.249945 |
| 15 | 7 | 0 | 0.707442 | 0.096677 | 0.940218 |
| 16 | 7 | 0 | -0.62377 | -0.06672 | 0.943093 |
| 17 | 6 | 0 | 1.134884 | 0.151599 | 2.250849 |
| 18 | 6 | 0 | -4.44343 | -0.45667 | 1.425872 |
| 19 | 6 | 0 | -4.57678 | -0.52402 | 2.839503 |
| 20 | 6 | 0 | -5.86902 | -0.65116 | 3.365617 |
| 21 | 6 | 0 | -6.94017 | -0.69266 | 2.464305 |
| 22 | 6 | 0 | -6.76696 | -0.60362 | 1.067521 |
| 23 | 6 | 0 | -5.49264 | -0.47694 | 0.505055 |
| 24 | 6 | 0 | -2.49735 | -0.31695 | 2.391318 |
| 25 | 7 | 0 | -3.11968 | -0.33982 | 1.170485 |
| 26 | 7 | 0 | -3.31555 | -0.42977 | 3.431946 |
| 27 | 6 | 0 | -5.26838 | -0.34704 | -0.98157 |
| 28 | 8 | 0 | -6.2215 | -0.39829 | -1.74952 |
| 29 | 8 | 0 | -4.03186 | -0.1646 | -1.4554 |
| 30 | 44 | 0 | -2.17372 | -0.05887 | -0.58566 |
| 31 | 44 | 0 | 2.297852 | 0.111539 | -0.49481 |
| 32 | 6 | 0 | -2.40829 | -2.62157 | -2.10486 |
| 33 | 6 | 0 | -1.73323 | -3.04067 | 0.070731 |
| 34 | 6 | 0 | -2.44001 | -3.97896 | -2.39266 |
| 35 | 6 | 0 | -1.74819 | -4.41218 | -0.15065 |
| 36 | 6 | 0 | -2.11175 | -4.91603 | -1.40491 |
| 37 | 6 | 0 | -2.86374 | 2.685561 | -1.55934 |
| 38 | 6 | 0 | -2.56041 | 2.731914 | 0.734736 |
| 39 | 6 | 0 | -3.23197 | 4.024281 | -1.57222 |
| 40 | 6 | 0 | -2.91838 | 4.073842 | 0.791946 |
| 41 | 6 | 0 | -3.27637 | 4.754773 | -0.3782 |
| 42 | 6 | 0 | 1.133272 | 2.954768 | -0.24274 |
| 43 | 6 | 0 | 2.974685 | 2.841252 | -1.64127 |
| 44 | 6 | 0 | 1.001154 | 4.318571 | -0.46847 |
| 45 | 6 | 0 | 2.896306 | 4.20119 | -1.90938 |
| 46 | 6 | 0 | 1.897001 | 4.98173 | -1.31535 |
| 47 | 6 | 0 | 3.109979 | -2.6044 | -1.45547 |
| 48 | 6 | 0 | 2.149042 | -2.7626 | 0.644377 |
| 49 | 6 | 0 | 3.332982 | -3.97294 | -1.49449 |
| 50 | 6 | 0 | 2.35218 | -4.13711 | 0.672415 |
| 51 | 6 | 0 | 2.957246 | -4.78015 | -0.41262 |
| 52 | 7 | 0 | 2.517904 | -1.99999 | -0.40227 |
| 53 | 7 | 0 | 2.106221 | 2.216886 | -0.81428 |
| 54 | 7 | 0 | -2.05632 | -2.15684 | -0.88938 |
| 55 | 7 | 0 | -2.52997 | 2.044085 | -0.42143 |

| | | | | | |
|---|---|---|---|---|---|
| 56 | 1 | 0 | -6.03618 | -0.71046 | 4.43702 |
| 57 | 1 | 0 | -7.94897 | -0.79014 | 2.855881 |
| 58 | 1 | 0 | -7.62349 | -0.62643 | 0.401831 |
| 59 | 1 | 0 | 0.037263 | -0.01578 | 4.184526 |
| 60 | 1 | 0 | 6.122375 | 0.552831 | 4.436548 |
| 61 | 1 | 0 | 8.046881 | 0.587427 | 2.86233 |
| 62 | 1 | 0 | 7.726078 | 0.466955 | 0.405289 |
| 63 | 1 | 0 | -1.46467 | -2.62495 | 1.033725 |
| 64 | 1 | 0 | -2.68009 | -1.87009 | -2.83551 |
| 65 | 1 | 0 | -2.73397 | -4.29934 | -3.38722 |
| 66 | 1 | 0 | -1.4831 | -5.08151 | 0.661611 |
| 67 | 1 | 0 | -2.8307 | 2.087466 | -2.46148 |
| 68 | 1 | 0 | -2.29964 | 2.183608 | 1.63146 |
| 69 | 1 | 0 | -3.49787 | 4.48773 | -2.51717 |
| 70 | 1 | 0 | -2.93126 | 4.575086 | 1.754789 |
| 71 | 1 | 0 | 0.191599 | 4.854098 | 0.016991 |
| 72 | 1 | 0 | 3.623722 | 4.646048 | -2.58133 |
| 73 | 1 | 0 | 3.740547 | 2.206905 | -2.07402 |
| 74 | 1 | 0 | 0.448409 | 2.418919 | 0.399685 |
| 75 | 1 | 0 | 3.426022 | -1.94783 | -2.25583 |
| 76 | 1 | 0 | 1.685914 | -2.24881 | 1.477439 |
| 77 | 1 | 0 | 3.817525 | -4.40139 | -2.36639 |
| 78 | 1 | 0 | 2.043222 | -4.69767 | 1.549065 |
| 79 | 8 | 0 | -1.15154 | 0.234373 | -2.07889 |
| 80 | 8 | 0 | 1.274603 | -0.22186 | -2.35679 |
| 81 | 1 | 0 | 0.251289 | -0.01681 | -2.29439 |
| 82 | 6 | 0 | 1.807577 | 6.465083 | -1.5576 |
| 83 | 1 | 0 | 0.792925 | 6.837498 | -1.39403 |
| 84 | 1 | 0 | 2.471937 | 7.003631 | -0.87092 |
| 85 | 1 | 0 | 2.113858 | 6.722232 | -2.5754 |
| 86 | 6 | 0 | -3.72427 | 6.192695 | -0.35021 |
| 87 | 1 | 0 | -4.81809 | 6.247785 | -0.29425 |
| 88 | 1 | 0 | -3.32463 | 6.722028 | 0.518891 |
| 89 | 1 | 0 | -3.41865 | 6.725396 | -1.2553 |
| 90 | 6 | 0 | 3.215481 | -6.2639 | -0.41862 |
| 91 | 1 | 0 | 2.748036 | -6.75843 | 0.436002 |
| 92 | 1 | 0 | 2.835611 | -6.72531 | -1.3363 |
| 93 | 1 | 0 | 4.291742 | -6.46745 | -0.37985 |
| 94 | 6 | 0 | -2.18012 | -6.39645 | -1.67418 |
| 95 | 1 | 0 | -1.58636 | -6.96432 | -0.95322 |
| 96 | 1 | 0 | -3.21571 | -6.7496 | -1.60149 |
| 97 | 1 | 0 | -1.82633 | -6.63467 | -2.68159 |
| 98 | 1 | 0 | 1.655635 | 0.24555 | -3.11113 |

**Table S43.** Cartesian coordinates for $Ru^{III}Ru^{IV}$-4pic-OH-$OH_2$-A.

| Center number | Atomic number | Atomic type | Coordinates (Ångström) | | |
|---|---|---|---|---|---|
| | | | X | Y | Z |
| 1 | 6 | 0 | 4.512483 | 0.344946 | 1.441374 |
| 2 | 6 | 0 | 4.630215 | 0.39392 | 2.852236 |
| 3 | 6 | 0 | 5.912373 | 0.468568 | 3.399633 |

| | | | | | |
|---|---|---|---|---|---|
| 4 | 6 | 0 | 6.996594 | 0.480153 | 2.50739 |
| 5 | 6 | 0 | 6.838365 | 0.411972 | 1.109956 |
| 6 | 6 | 0 | 5.566535 | 0.336427 | 0.527783 |
| 7 | 6 | 0 | 2.538328 | 0.260159 | 2.402654 |
| 8 | 6 | 0 | 5.36215 | 0.239331 | -0.97147 |
| 9 | 8 | 0 | 6.337681 | 0.287468 | -1.70872 |
| 10 | 8 | 0 | 4.131445 | 0.101774 | -1.46634 |
| 11 | 7 | 0 | 3.182314 | 0.2719 | 1.176191 |
| 12 | 7 | 0 | 3.351216 | 0.334981 | 3.437175 |
| 13 | 6 | 0 | 0.00318 | -0.006 | 3.101093 |
| 14 | 6 | 0 | -1.09237 | -0.12519 | 2.239705 |
| 15 | 7 | 0 | 0.667845 | 0.082667 | 0.932024 |
| 16 | 7 | 0 | -0.66046 | -0.05557 | 0.928159 |
| 17 | 6 | 0 | 1.097682 | 0.129638 | 2.244438 |
| 18 | 6 | 0 | -4.51944 | -0.40473 | 1.456071 |
| 19 | 6 | 0 | -4.7011 | -0.50045 | 2.848351 |
| 20 | 6 | 0 | -5.98443 | -0.63783 | 3.37784 |
| 21 | 6 | 0 | -7.03486 | -0.66511 | 2.453563 |
| 22 | 6 | 0 | -6.83427 | -0.55542 | 1.06214 |
| 23 | 6 | 0 | -5.55477 | -0.41768 | 0.521074 |
| 24 | 6 | 0 | -2.52377 | -0.28075 | 2.355709 |
| 25 | 7 | 0 | -3.18449 | -0.28191 | 1.204558 |
| 26 | 7 | 0 | -3.40605 | -0.41573 | 3.391911 |
| 27 | 6 | 0 | -5.32068 | -0.27674 | -0.97238 |
| 28 | 8 | 0 | -6.28258 | -0.31819 | -1.72248 |
| 29 | 8 | 0 | -4.08807 | -0.10116 | -1.44271 |
| 30 | 44 | 0 | -2.21454 | -0.03194 | -0.60402 |
| 31 | 44 | 0 | 2.295299 | 0.079183 | -0.49481 |
| 32 | 6 | 0 | -2.67017 | -2.6125 | -2.05729 |
| 33 | 6 | 0 | -1.61827 | -3.02594 | -0.03525 |
| 34 | 6 | 0 | -2.68803 | -3.96547 | -2.36232 |
| 35 | 6 | 0 | -1.60892 | -4.39335 | -0.2783 |
| 36 | 6 | 0 | -2.15486 | -4.90011 | -1.46426 |
| 37 | 6 | 0 | -3.02996 | 2.682924 | -1.56745 |
| 38 | 6 | 0 | -2.34371 | 2.831937 | 0.637683 |
| 39 | 6 | 0 | -3.34976 | 4.033061 | -1.59371 |
| 40 | 6 | 0 | -2.64746 | 4.186682 | 0.680668 |
| 41 | 6 | 0 | -3.17228 | 4.824533 | -0.45115 |
| 42 | 6 | 0 | 2.968038 | 2.732731 | -1.78486 |
| 43 | 6 | 0 | 1.408936 | 3.015697 | -0.09801 |
| 44 | 6 | 0 | 2.973835 | 4.094215 | -2.05344 |
| 45 | 6 | 0 | 1.374915 | 4.387054 | -0.30995 |
| 46 | 6 | 0 | 2.171373 | 4.96621 | -1.30622 |
| 47 | 6 | 0 | 3.058979 | -2.64087 | -1.49244 |
| 48 | 6 | 0 | 2.230948 | -2.80096 | 0.661493 |
| 49 | 6 | 0 | 3.325418 | -4.00265 | -1.52717 |
| 50 | 6 | 0 | 2.482632 | -4.16546 | 0.696902 |
| 51 | 6 | 0 | 3.049097 | -4.8055 | -0.4137 |
| 52 | 7 | 0 | 2.51077 | -2.03855 | -0.41476 |
| 53 | 7 | 0 | 2.19215 | 2.188375 | -0.81887 |
| 54 | 7 | 0 | -2.14241 | -2.14347 | -0.90559 |
| 55 | 7 | 0 | -2.53372 | 2.082971 | -0.4643 |
| 56 | 1 | 0 | -6.1723 | -0.71722 | 4.443338 |
| 57 | 1 | 0 | -8.04859 | -0.77044 | 2.826306 |
| 58 | 1 | 0 | -7.67849 | -0.57266 | 0.381461 |

| | | | | | |
|---|---|---|---|---|---|
| 59 | 1 | 0 | 0.021474 | -0.01689 | 4.180533 |
| 60 | 1 | 0 | 6.06829 | 0.510323 | 4.472766 |
| 61 | 1 | 0 | 8.00249 | 0.537298 | 2.912118 |
| 62 | 1 | 0 | 7.703211 | 0.412752 | 0.455203 |
| 63 | 1 | 0 | -1.19305 | -2.61354 | 0.871238 |
| 64 | 1 | 0 | -3.09519 | -1.86982 | -2.72101 |
| 65 | 1 | 0 | -3.12662 | -4.28525 | -3.30217 |
| 66 | 1 | 0 | -1.17303 | -5.0584 | 0.460046 |
| 67 | 1 | 0 | -3.17816 | 2.044218 | -2.42909 |
| 68 | 1 | 0 | -1.93535 | 2.324842 | 1.503606 |
| 69 | 1 | 0 | -3.7491 | 4.459638 | -2.5083 |
| 70 | 1 | 0 | -2.48021 | 4.737723 | 1.600899 |
| 71 | 1 | 0 | 3.620607 | 4.470448 | -2.8398 |
| 72 | 1 | 0 | 0.725814 | 5.000272 | 0.306624 |
| 73 | 1 | 0 | 0.799863 | 2.549038 | 0.664742 |
| 74 | 1 | 0 | 3.611285 | 2.038287 | -2.3145 |
| 75 | 1 | 0 | 3.308179 | -1.99223 | -2.32153 |
| 76 | 1 | 0 | 1.80579 | -2.29174 | 1.517397 |
| 77 | 1 | 0 | 3.770928 | -4.42692 | -2.4213 |
| 78 | 1 | 0 | 2.249994 | -4.72228 | 1.599316 |
| 79 | 8 | 0 | -1.20451 | 0.218727 | -2.07827 |
| 80 | 6 | 0 | -2.18884 | -6.37541 | -1.75872 |
| 81 | 1 | 0 | -1.55195 | -6.93948 | -1.07357 |
| 82 | 1 | 0 | -3.21067 | -6.75994 | -1.66067 |
| 83 | 1 | 0 | -1.86544 | -6.58039 | -2.78371 |
| 84 | 6 | 0 | -3.55323 | 6.280276 | -0.43682 |
| 85 | 1 | 0 | -4.63721 | 6.386434 | -0.31031 |
| 86 | 1 | 0 | -3.07055 | 6.81672 | 0.383616 |
| 87 | 1 | 0 | -3.2896 | 6.76962 | -1.37872 |
| 88 | 6 | 0 | 2.191263 | 6.451457 | -1.54545 |
| 89 | 1 | 0 | 3.085208 | 6.895845 | -1.09192 |
| 90 | 1 | 0 | 2.22371 | 6.681421 | -2.61427 |
| 91 | 1 | 0 | 1.318643 | 6.942952 | -1.10865 |
| 92 | 6 | 0 | 3.38203 | -6.27299 | -0.39904 |
| 93 | 1 | 0 | 4.439941 | -6.41722 | -0.14894 |
| 94 | 1 | 0 | 2.792094 | -6.81411 | 0.345014 |
| 95 | 1 | 0 | 3.216472 | -6.73008 | -1.37855 |
| 96 | 8 | 0 | 1.300219 | -0.22761 | -2.39177 |
| 97 | 1 | 0 | 1.694274 | 0.210683 | -3.15746 |
| 98 | 1 | 0 | 0.310609 | -0.02632 | -2.3557 |
| 99 | 1 | 0 | -3.16558 | -0.45479 | 4.371242 |

**Table S44.** Cartesian coordinates for $Ru^{III}Ru^{IV}$-4pic-2$OH_2$-A.

| Center number | Atomic number | Atomic type | Coordinates (Ångström) | | |
|---|---|---|---|---|---|
| | | | X | Y | Z |
| 1 | 6 | 0 | -4.58302 | 0.151333 | 1.458338 |
| 2 | 6 | 0 | -4.78159 | 0.083261 | 2.848897 |
| 3 | 6 | 0 | -6.07807 | 0.098903 | 3.368288 |
| 4 | 6 | 0 | -7.11787 | 0.177177 | 2.436819 |
| 5 | 6 | 0 | -6.89985 | 0.238286 | 1.043156 |

| | | | | | |
|---|---|---|---|---|---|
| 6 | 6 | 0 | -5.60914 | 0.232965 | 0.514566 |
| 7 | 6 | 0 | -2.59117 | 0.028908 | 2.376553 |
| 8 | 6 | 0 | -5.33564 | 0.281054 | -0.96812 |
| 9 | 8 | 0 | -6.23793 | 0.141979 | -1.76893 |
| 10 | 8 | 0 | -4.07613 | 0.520187 | -1.39133 |
| 11 | 7 | 0 | -3.24057 | 0.116199 | 1.219761 |
| 12 | 7 | 0 | -3.49302 | 0.006785 | 3.403711 |
| 13 | 6 | 0 | -0.05207 | -0.04615 | 3.109001 |
| 14 | 6 | 0 | 1.047312 | -0.04513 | 2.244863 |
| 15 | 7 | 0 | -0.71981 | -0.01586 | 0.934063 |
| 16 | 7 | 0 | 0.615549 | -0.02403 | 0.935589 |
| 17 | 6 | 0 | -1.1531 | -0.02762 | 2.250463 |
| 18 | 6 | 0 | 4.49699 | -0.15887 | 1.50698 |
| 19 | 6 | 0 | 4.668234 | -0.1428 | 2.903564 |
| 20 | 6 | 0 | 5.952227 | -0.17507 | 3.452799 |
| 21 | 6 | 0 | 7.009384 | -0.22421 | 2.540238 |
| 22 | 6 | 0 | 6.818605 | -0.24344 | 1.140435 |
| 23 | 6 | 0 | 5.541543 | -0.21208 | 0.58236 |
| 24 | 6 | 0 | 2.490471 | -0.08265 | 2.381756 |
| 25 | 7 | 0 | 3.160231 | -0.11848 | 1.238001 |
| 26 | 7 | 0 | 3.365872 | -0.09529 | 3.430858 |
| 27 | 6 | 0 | 5.31474 | -0.24086 | -0.91532 |
| 28 | 8 | 0 | 6.273911 | -0.27777 | -1.66206 |
| 29 | 8 | 0 | 4.069357 | -0.23563 | -1.40568 |
| 30 | 44 | 0 | 2.215481 | -0.12664 | -0.59801 |
| 31 | 44 | 0 | -2.31249 | 0.143708 | -0.53003 |
| 32 | 6 | 0 | 2.935589 | 2.450449 | -1.93559 |
| 33 | 6 | 0 | 2.261134 | 2.877475 | 0.238692 |
| 34 | 6 | 0 | 3.225159 | 3.790909 | -2.14349 |
| 35 | 6 | 0 | 2.535689 | 4.230729 | 0.098049 |
| 36 | 6 | 0 | 3.039808 | 4.724044 | -1.11375 |
| 37 | 6 | 0 | 2.7148 | -2.83071 | -1.78004 |
| 38 | 6 | 0 | 2.009765 | -3.083 | 0.410851 |
| 39 | 6 | 0 | 2.892991 | -4.19994 | -1.91476 |
| 40 | 6 | 0 | 2.171832 | -4.46007 | 0.344631 |
| 41 | 6 | 0 | 2.632417 | -5.05812 | -0.83726 |
| 42 | 6 | 0 | -3.45349 | -2.39573 | -1.73583 |
| 43 | 6 | 0 | -2.11702 | -2.8818 | 0.091588 |
| 44 | 6 | 0 | -3.71988 | -3.73999 | -1.94249 |
| 45 | 6 | 0 | -2.34498 | -4.24298 | -0.0606 |
| 46 | 6 | 0 | -3.16658 | -4.71177 | -1.09439 |
| 47 | 6 | 0 | -2.39856 | 2.888235 | -1.72301 |
| 48 | 6 | 0 | -1.75652 | 3.026664 | 0.49576 |
| 49 | 6 | 0 | -2.39701 | 4.269746 | -1.84414 |
| 50 | 6 | 0 | -1.74124 | 4.413094 | 0.443833 |
| 51 | 6 | 0 | -2.07516 | 5.077688 | -0.74492 |
| 52 | 7 | 0 | -2.08204 | 2.262453 | -0.56681 |
| 53 | 7 | 0 | -2.6589 | -1.95688 | -0.72823 |
| 54 | 7 | 0 | 2.459566 | 1.9922 | -0.75651 |
| 55 | 7 | 0 | 2.278172 | -2.27127 | -0.62979 |
| 56 | 1 | 0 | 6.133446 | -0.16381 | 4.522097 |
| 57 | 1 | 0 | 8.023474 | -0.25047 | 2.925194 |
| 58 | 1 | 0 | 7.671717 | -0.2849 | 0.471835 |
| 59 | 1 | 0 | -0.05 | -0.05538 | 4.188957 |
| 60 | 1 | 0 | -6.28218 | 0.051984 | 4.432504 |

| | | | | | |
|---|---|---|---|---|---|
| 61 | 1 | 0 | -8.13917 | 0.189614 | 2.802643 |
| 62 | 1 | 0 | -7.74093 | 0.290552 | 0.360203 |
| 63 | 1 | 0 | 1.875953 | 2.479997 | 1.169946 |
| 64 | 1 | 0 | 3.096228 | 1.707912 | -2.70691 |
| 65 | 1 | 0 | 3.609585 | 4.099631 | -3.11024 |
| 66 | 1 | 0 | 2.364928 | 4.896362 | 0.938087 |
| 67 | 1 | 0 | 2.936985 | -2.14611 | -2.58888 |
| 68 | 1 | 0 | 1.662566 | -2.60669 | 1.320264 |
| 69 | 1 | 0 | 3.251326 | -4.59077 | -2.86168 |
| 70 | 1 | 0 | 1.950668 | -5.06296 | 1.219742 |
| 71 | 1 | 0 | -4.37129 | -4.02388 | -2.76291 |
| 72 | 1 | 0 | -1.88167 | -4.93635 | 0.633726 |
| 73 | 1 | 0 | -1.48166 | -2.50766 | 0.884821 |
| 74 | 1 | 0 | -3.90028 | -1.6322 | -2.36269 |
| 75 | 1 | 0 | -2.66974 | 2.249809 | -2.55245 |
| 76 | 1 | 0 | -1.50691 | 2.504154 | 1.410805 |
| 77 | 1 | 0 | -2.66431 | 4.711566 | -2.79869 |
| 78 | 1 | 0 | -1.48107 | 4.97295 | 1.336476 |
| 79 | 8 | 0 | 1.179828 | -0.12668 | -2.0769 |
| 80 | 6 | 0 | 3.392874 | 6.174254 | -1.29602 |
| 81 | 1 | 0 | 2.916304 | 6.806048 | -0.54301 |
| 82 | 1 | 0 | 4.477128 | 6.311388 | -1.20661 |
| 83 | 1 | 0 | 3.105613 | 6.531928 | -2.28872 |
| 84 | 6 | 0 | 2.865788 | -6.53998 | -0.93973 |
| 85 | 1 | 0 | 3.935439 | -6.75977 | -0.83978 |
| 86 | 1 | 0 | 2.34049 | -7.08932 | -0.15516 |
| 87 | 1 | 0 | 2.549077 | -6.92625 | -1.91255 |
| 88 | 6 | 0 | -3.45997 | -6.17342 | -1.28387 |
| 89 | 1 | 0 | -4.5152 | -6.38016 | -1.07197 |
| 90 | 1 | 0 | -3.27738 | -6.47843 | -2.31913 |
| 91 | 1 | 0 | -2.85437 | -6.7986 | -0.62476 |
| 92 | 6 | 0 | -2.1179 | 6.577853 | -0.83315 |
| 93 | 1 | 0 | -3.15543 | 6.927996 | -0.77497 |
| 94 | 1 | 0 | -1.56373 | 7.048749 | -0.01809 |
| 95 | 1 | 0 | -1.71335 | 6.931982 | -1.78542 |
| 96 | 8 | 0 | -1.28263 | 0.138414 | -2.38976 |
| 97 | 1 | 0 | -1.58943 | -0.48902 | -3.05851 |
| 98 | 1 | 0 | -0.25686 | 0.042449 | -2.30106 |
| 99 | 1 | 0 | -3.274 | -0.06791 | 4.386465 |
| 100 | 1 | 0 | 3.124625 | -0.07008 | 4.411082 |

**Table S45.** Cartesian coordinates for $Ru^{III}Ru^{IV}$-4pic-$H^{+}$-$2OH_2$-A.

| Center number | Atomic number | Atomic type | Coordinates (Ångström) | | |
|---|---|---|---|---|---|
| | | | X | Y | Z |
| 1 | 6 | 0 | 4.471925 | 0.428028 | 1.466258 |
| 2 | 6 | 0 | 4.651731 | 0.5587 | 2.857687 |
| 3 | 6 | 0 | 5.941468 | 0.673572 | 3.385258 |
| 4 | 6 | 0 | 6.988425 | 0.636448 | 2.461682 |
| 5 | 6 | 0 | 6.789242 | 0.485655 | 1.067707 |
| 6 | 6 | 0 | 5.509647 | 0.373299 | 0.528137 |

| | | | | | |
|---|---|---|---|---|---|
| 7 | 6 | 0 | 2.470799 | 0.389909 | 2.374671 |
| 8 | 6 | 0 | 5.278955 | 0.189934 | -0.95298 |
| 9 | 8 | 0 | 6.215144 | 0.16101 | -1.72058 |
| 10 | 8 | 0 | 4.015827 | 0.056768 | -1.44448 |
| 11 | 7 | 0 | 3.138019 | 0.342277 | 1.222094 |
| 12 | 7 | 0 | 3.359785 | 0.526089 | 3.404748 |
| 13 | 6 | 0 | -0.05226 | 0.089837 | 3.101073 |
| 14 | 6 | 0 | -1.14275 | -0.06974 | 2.248304 |
| 15 | 7 | 0 | 0.61543 | 0.159161 | 0.917737 |
| 16 | 7 | 0 | -0.7171 | 0.003887 | 0.930261 |
| 17 | 6 | 0 | 1.039403 | 0.236964 | 2.234865 |
| 18 | 6 | 0 | -4.53742 | -0.54741 | 1.437712 |
| 19 | 6 | 0 | -4.72687 | -0.64472 | 2.828168 |
| 20 | 6 | 0 | -6.00597 | -0.86633 | 3.34551 |
| 21 | 6 | 0 | -7.0431 | -0.97529 | 2.413633 |
| 22 | 6 | 0 | -6.84148 | -0.86539 | 1.019434 |
| 23 | 6 | 0 | -5.56723 | -0.64121 | 0.497318 |
| 24 | 6 | 0 | -2.56477 | -0.29443 | 2.367186 |
| 25 | 7 | 0 | -3.2107 | -0.34865 | 1.200847 |
| 26 | 7 | 0 | -3.44927 | -0.47508 | 3.386364 |
| 27 | 6 | 0 | -5.30412 | -0.49286 | -0.96295 |
| 28 | 8 | 0 | -6.13905 | -0.63721 | -1.81704 |
| 29 | 8 | 0 | -4.02217 | -0.13336 | -1.34977 |
| 30 | 44 | 0 | -2.26457 | 0.007504 | -0.55899 |
| 31 | 44 | 0 | 2.266194 | 0.043746 | -0.51258 |
| 32 | 6 | 0 | -2.15173 | -2.40307 | -2.30487 |
| 33 | 6 | 0 | -1.79147 | -3.00676 | -0.0874 |
| 34 | 6 | 0 | -2.13321 | -3.72903 | -2.70227 |
| 35 | 6 | 0 | -1.76241 | -4.35042 | -0.42315 |
| 36 | 6 | 0 | -1.94762 | -4.75228 | -1.75725 |
| 37 | 6 | 0 | -3.35719 | 2.702905 | -1.37996 |
| 38 | 6 | 0 | -2.32285 | 2.883918 | 0.688799 |
| 39 | 6 | 0 | -3.7297 | 4.036903 | -1.34843 |
| 40 | 6 | 0 | -2.67259 | 4.222436 | 0.783482 |
| 41 | 6 | 0 | -3.4027 | 4.841712 | -0.24417 |
| 42 | 6 | 0 | 2.777689 | 2.592428 | -2.07323 |
| 43 | 6 | 0 | 1.634918 | 3.063721 | -0.11512 |
| 44 | 6 | 0 | 2.841347 | 3.937064 | -2.40106 |
| 45 | 6 | 0 | 1.667254 | 4.424529 | -0.38291 |
| 46 | 6 | 0 | 2.286468 | 4.904405 | -1.54768 |
| 47 | 6 | 0 | 3.071031 | -2.73352 | -1.32943 |
| 48 | 6 | 0 | 2.24733 | -2.76937 | 0.834394 |
| 49 | 6 | 0 | 3.378697 | -4.08339 | -1.27063 |
| 50 | 6 | 0 | 2.528178 | -4.12118 | 0.957983 |
| 51 | 6 | 0 | 3.123315 | -4.82106 | -0.10443 |
| 52 | 7 | 0 | 2.513163 | -2.06652 | -0.28978 |
| 53 | 7 | 0 | 2.177353 | 2.142897 | -0.94247 |
| 54 | 7 | 0 | -1.98742 | -2.03729 | -1.00932 |
| 55 | 7 | 0 | -2.659 | 2.11733 | -0.37305 |
| 56 | 1 | 0 | -6.20075 | -0.95022 | 4.409268 |
| 57 | 1 | 0 | -8.05004 | -1.1481 | 2.778512 |
| 58 | 1 | 0 | -7.68301 | -0.95023 | 0.339859 |
| 59 | 1 | 0 | -0.0472 | 0.086961 | 4.181266 |
| 60 | 1 | 0 | 6.134399 | 0.781102 | 4.447065 |
| 61 | 1 | 0 | 8.005326 | 0.720369 | 2.830538 |

| | | | | | |
|---|---|---|---|---|---|
| 62 | 1 | 0 | 7.642149 | 0.45125 | 0.398083 |
| 63 | 1 | 0 | -1.6639 | -2.68255 | 0.937879 |
| 64 | 1 | 0 | -2.31279 | -1.60146 | -3.01479 |
| 65 | 1 | 0 | -2.28118 | -3.96318 | -3.7516 |
| 66 | 1 | 0 | -1.61147 | -5.08715 | 0.358962 |
| 67 | 1 | 0 | -3.64532 | 2.06244 | -2.20588 |
| 68 | 1 | 0 | -1.75991 | 2.402851 | 1.478992 |
| 69 | 1 | 0 | -4.29307 | 4.441597 | -2.18333 |
| 70 | 1 | 0 | -2.38232 | 4.781742 | 1.667169 |
| 71 | 1 | 0 | 3.346546 | 4.229113 | -3.31634 |
| 72 | 1 | 0 | 1.223299 | 5.11376 | 0.32806 |
| 73 | 1 | 0 | 1.169201 | 2.685921 | 0.786311 |
| 74 | 1 | 0 | 3.257755 | 1.843707 | -2.69141 |
| 75 | 1 | 0 | 3.30104 | -2.14699 | -2.20838 |
| 76 | 1 | 0 | 1.800254 | -2.22434 | 1.656214 |
| 77 | 1 | 0 | 3.838775 | -4.55261 | -2.13437 |
| 78 | 1 | 0 | 2.298561 | -4.62587 | 1.891065 |
| 79 | 8 | 0 | -1.13621 | 0.425329 | -2.12901 |
| 80 | 6 | 0 | -1.97553 | -6.19822 | -2.15595 |
| 81 | 1 | 0 | -1.57873 | -6.84674 | -1.3726 |
| 82 | 1 | 0 | -3.00868 | -6.50889 | -2.35554 |
| 83 | 1 | 0 | -1.4114 | -6.36639 | -3.07795 |
| 84 | 6 | 0 | -3.83738 | 6.275208 | -0.16164 |
| 85 | 1 | 0 | -4.90183 | 6.326569 | 0.099408 |
| 86 | 1 | 0 | -3.28234 | 6.827601 | 0.599101 |
| 87 | 1 | 0 | -3.72254 | 6.781647 | -1.12417 |
| 88 | 6 | 0 | 2.383933 | 6.370114 | -1.85669 |
| 89 | 1 | 0 | 3.415674 | 6.714603 | -1.71711 |
| 90 | 1 | 0 | 2.121249 | 6.572531 | -2.89936 |
| 91 | 1 | 0 | 1.741221 | 6.968464 | -1.20809 |
| 92 | 6 | 0 | 3.499738 | -6.26966 | 0.00961 |
| 93 | 1 | 0 | 4.558943 | -6.35697 | 0.282511 |
| 94 | 1 | 0 | 2.919861 | -6.78247 | 0.780207 |
| 95 | 1 | 0 | 3.37152 | -6.79345 | -0.9412 |
| 96 | 1 | 0 | -1.1781 | 1.367963 | -2.35292 |
| 97 | 8 | 0 | 1.317757 | -0.4125 | -2.39823 |
| 98 | 1 | 0 | 1.785745 | -0.16133 | -3.20717 |
| 99 | 1 | 0 | 0.366766 | -0.10622 | -2.43814 |
| 100 | 1 | 0 | -3.22824 | -0.4925 | 4.373313 |
| 101 | 1 | 0 | 3.133744 | 0.609183 | 4.386448 |

**Table S46.** Cartesian coordinates for $Ru^{IV}Ru^{IV}$-4pic-O-OH-A.

| Center number | Atomic number | Atomic type | Coordinates (Ångström) | | |
|---|---|---|---|---|---|
| | | | X | Y | Z |
| 1 | 6 | 0 | 4.527677 | -0.36453 | 1.427196 |
| 2 | 6 | 0 | 4.645493 | -0.4878 | 2.83587 |
| 3 | 6 | 0 | 5.926982 | -0.64511 | 3.371338 |
| 4 | 6 | 0 | 7.010961 | -0.66817 | 2.480495 |
| 5 | 6 | 0 | 6.855309 | -0.53374 | 1.088152 |
| 6 | 6 | 0 | 5.585456 | -0.37368 | 0.52022 |

| | | | | | |
|---|---|---|---|---|---|
| 7 | 6 | 0 | 2.565358 | -0.25591 | 2.388615 |
| 8 | 6 | 0 | 5.365569 | -0.20561 | -0.95796 |
| 9 | 8 | 0 | 6.302055 | -0.25526 | -1.74128 |
| 10 | 8 | 0 | 4.120932 | 0.022582 | -1.39903 |
| 11 | 7 | 0 | 3.203801 | -0.21951 | 1.165544 |
| 12 | 7 | 0 | 3.37537 | -0.41258 | 3.421533 |
| 13 | 6 | 0 | 0.026043 | -0.00333 | 3.086636 |
| 14 | 6 | 0 | -1.06712 | 0.127547 | 2.226856 |
| 15 | 7 | 0 | 0.703214 | -0.04323 | 0.928799 |
| 16 | 7 | 0 | -0.63432 | 0.093742 | 0.919955 |
| 17 | 6 | 0 | 1.126965 | -0.11208 | 2.236741 |
| 18 | 6 | 0 | -4.47707 | 0.285289 | 1.465292 |
| 19 | 6 | 0 | -4.58729 | 0.341007 | 2.881603 |
| 20 | 6 | 0 | -5.87522 | 0.404524 | 3.431142 |
| 21 | 6 | 0 | -6.96225 | 0.397654 | 2.548948 |
| 22 | 6 | 0 | -6.81007 | 0.321928 | 1.148146 |
| 23 | 6 | 0 | -5.54201 | 0.258534 | 0.562869 |
| 24 | 6 | 0 | -2.51158 | 0.2376 | 2.390185 |
| 25 | 7 | 0 | -3.15515 | 0.232744 | 1.184414 |
| 26 | 7 | 0 | -3.31263 | 0.304528 | 3.449427 |
| 27 | 6 | 0 | -5.33625 | 0.147076 | -0.92809 |
| 28 | 8 | 0 | -6.30392 | 0.156515 | -1.67923 |
| 29 | 8 | 0 | -4.10106 | 0.02966 | -1.42551 |
| 30 | 44 | 0 | -2.22553 | 0.014946 | -0.59381 |
| 31 | 44 | 0 | 2.268456 | 0.031792 | -0.56295 |
| 32 | 6 | 0 | -2.6724 | -2.77094 | -1.60226 |
| 33 | 6 | 0 | -2.47692 | -2.80035 | 0.703167 |
| 34 | 6 | 0 | -2.93642 | -4.13384 | -1.63147 |
| 35 | 6 | 0 | -2.73314 | -4.1653 | 0.744035 |
| 36 | 6 | 0 | -2.98088 | -4.86863 | -0.4406 |
| 37 | 6 | 0 | -2.64214 | 2.57776 | -2.07352 |
| 38 | 6 | 0 | -1.86947 | 3.00879 | 0.067126 |
| 39 | 6 | 0 | -2.72687 | 3.935145 | -2.35243 |
| 40 | 6 | 0 | -1.93401 | 4.379992 | -0.14635 |
| 41 | 6 | 0 | -2.37497 | 4.877522 | -1.3785 |
| 42 | 6 | 0 | 2.441706 | 2.843447 | 0.74458 |
| 43 | 6 | 0 | 2.745649 | 2.816479 | -1.55147 |
| 44 | 6 | 0 | 2.66277 | 4.213422 | 0.793911 |
| 45 | 6 | 0 | 2.97724 | 4.185139 | -1.57058 |
| 46 | 6 | 0 | 2.946607 | 4.921548 | -0.38026 |
| 47 | 6 | 0 | 2.608344 | -2.51628 | -2.08708 |
| 48 | 6 | 0 | 1.792021 | -2.95676 | 0.037884 |
| 49 | 6 | 0 | 2.624194 | -3.86852 | -2.39782 |
| 50 | 6 | 0 | 1.787259 | -4.32315 | -0.2093 |
| 51 | 6 | 0 | 2.212807 | -4.81381 | -1.44986 |
| 52 | 7 | 0 | 2.196468 | -2.06508 | -0.88503 |
| 53 | 7 | 0 | 2.481416 | 2.151887 | -0.40917 |
| 54 | 7 | 0 | -2.44455 | -2.10859 | -0.45049 |
| 55 | 7 | 0 | -2.21637 | 2.119286 | -0.87978 |
| 56 | 1 | 0 | -6.02638 | 0.452069 | 4.505532 |
| 57 | 1 | 0 | -7.9676 | 0.445142 | 2.958339 |
| 58 | 1 | 0 | -7.67879 | 0.306075 | 0.498211 |
| 59 | 1 | 0 | 0.018179 | -0.02125 | 4.165167 |
| 60 | 1 | 0 | 6.079957 | -0.7434 | 4.441648 |
| 61 | 1 | 0 | 8.012999 | -0.79109 | 2.881271 |

| | | | | | |
|---|---|---|---|---|---|
| 62 | 1 | 0 | 7.717411 | -0.54966 | 0.429786 |
| 63 | 1 | 0 | -2.30197 | -2.23677 | 1.610816 |
| 64 | 1 | 0 | -2.63485 | -2.17027 | -2.5027 |
| 65 | 1 | 0 | -3.11857 | -4.61394 | -2.58791 |
| 66 | 1 | 0 | -2.75093 | -4.66939 | 1.705339 |
| 67 | 1 | 0 | -2.92893 | 1.82129 | -2.79338 |
| 68 | 1 | 0 | -1.53883 | 2.597836 | 1.012791 |
| 69 | 1 | 0 | -3.07878 | 4.250368 | -3.32966 |
| 70 | 1 | 0 | -1.64631 | 5.053651 | 0.654676 |
| 71 | 1 | 0 | 2.619281 | 4.719687 | 1.753065 |
| 72 | 1 | 0 | 3.187837 | 4.669349 | -2.51883 |
| 73 | 1 | 0 | 2.760621 | 2.217856 | -2.45358 |
| 74 | 1 | 0 | 2.230873 | 2.277956 | 1.643299 |
| 75 | 1 | 0 | 2.935513 | -1.76045 | -2.79015 |
| 76 | 1 | 0 | 1.467583 | -2.5525 | 0.988305 |
| 77 | 1 | 0 | 2.965371 | -4.17785 | -3.38055 |
| 78 | 1 | 0 | 1.452245 | -4.99881 | 0.571026 |
| 79 | 8 | 0 | -1.22436 | -0.2099 | -2.10486 |
| 80 | 8 | 0 | 1.233166 | 0.298203 | -2.11611 |
| 81 | 1 | 0 | 0.225458 | 0.069042 | -2.16009 |
| 82 | 6 | 0 | 3.235958 | 6.39923 | -0.35932 |
| 83 | 1 | 0 | 2.732887 | 6.894657 | 0.475432 |
| 84 | 1 | 0 | 4.312548 | 6.573687 | -0.2438 |
| 85 | 1 | 0 | 2.925305 | 6.880991 | -1.2904 |
| 86 | 6 | 0 | -2.49866 | 6.356596 | -1.6355 |
| 87 | 1 | 0 | -3.52981 | 6.688102 | -1.46369 |
| 88 | 1 | 0 | -1.8518 | 6.93556 | -0.97103 |
| 89 | 1 | 0 | -2.24618 | 6.604143 | -2.67057 |
| 90 | 6 | 0 | 2.255919 | -6.29 | -1.74391 |
| 91 | 1 | 0 | 1.583626 | -6.85002 | -1.08898 |
| 92 | 1 | 0 | 1.983805 | -6.49693 | -2.78282 |
| 93 | 1 | 0 | 3.269517 | -6.67887 | -1.58898 |
| 94 | 6 | 0 | -3.3151 | -6.33743 | -0.43157 |
| 95 | 1 | 0 | -2.87545 | -6.8444 | 0.431783 |
| 96 | 1 | 0 | -4.40118 | -6.47938 | -0.37678 |
| 97 | 1 | 0 | -2.96757 | -6.8333 | -1.34226 |

**Table S47.** Cartesian coordinates for $Ru^{IV}Ru^{IV}$-4pic-2OH-A.

| Center number | Atomic number | Atomic type | Coordinates (Ångström) | | |
|---|---|---|---|---|---|
| | | | X | Y | Z |
| 1 | 6 | 0 | 4.533659 | 0.329214 | 1.505591 |
| 2 | 6 | 0 | 4.691977 | 0.417445 | 2.901355 |
| 3 | 6 | 0 | 5.969348 | 0.513377 | 3.455015 |
| 4 | 6 | 0 | 7.036173 | 0.50561 | 2.55007 |
| 5 | 6 | 0 | 6.858252 | 0.399251 | 1.154613 |
| 6 | 6 | 0 | 5.585876 | 0.30316 | 0.589734 |
| 7 | 6 | 0 | 2.521565 | 0.264801 | 2.36404 |
| 8 | 6 | 0 | 5.375419 | 0.158745 | -0.9077 |
| 9 | 8 | 0 | 6.353877 | 0.168863 | -1.63743 |
| 10 | 8 | 0 | 4.149475 | 0.01211 | -1.40279 |

| | | | | | |
|---|---|---|---|---|---|
| 11 | 7 | 0 | 3.201115 | 0.25065 | 1.227909 |
| 12 | 7 | 0 | 3.385488 | 0.370729 | 3.419636 |
| 13 | 6 | 0 | -0.00154 | -0.01096 | 3.089773 |
| 14 | 6 | 0 | -1.09726 | -0.12743 | 2.234912 |
| 15 | 7 | 0 | 0.657614 | 0.102024 | 0.914396 |
| 16 | 7 | 0 | -0.6731 | -0.04137 | 0.928888 |
| 17 | 6 | 0 | 1.086914 | 0.138248 | 2.225935 |
| 18 | 6 | 0 | -4.49989 | -0.43808 | 1.458007 |
| 19 | 6 | 0 | -4.59721 | -0.58447 | 2.864519 |
| 20 | 6 | 0 | -5.86709 | -0.77592 | 3.416256 |
| 21 | 6 | 0 | -6.96089 | -0.80884 | 2.538058 |
| 22 | 6 | 0 | -6.82709 | -0.64982 | 1.145295 |
| 23 | 6 | 0 | -5.56871 | -0.45442 | 0.564417 |
| 24 | 6 | 0 | -2.53016 | -0.30019 | 2.394642 |
| 25 | 7 | 0 | -3.18277 | -0.26068 | 1.178057 |
| 26 | 7 | 0 | -3.31967 | -0.48869 | 3.434248 |
| 27 | 6 | 0 | -5.36772 | -0.2551 | -0.91011 |
| 28 | 8 | 0 | -6.29319 | -0.33503 | -1.69636 |
| 29 | 8 | 0 | -4.13073 | 0.055682 | -1.34732 |
| 30 | 44 | 0 | -2.28724 | 0.051707 | -0.55396 |
| 31 | 44 | 0 | 2.258966 | 0.005221 | -0.60248 |
| 32 | 6 | 0 | -2.60219 | -2.42039 | -2.1976 |
| 33 | 6 | 0 | -1.85695 | -2.97155 | -0.07178 |
| 34 | 6 | 0 | -2.6497 | -3.75772 | -2.56335 |
| 35 | 6 | 0 | -1.88666 | -4.32552 | -0.37402 |
| 36 | 6 | 0 | -2.29492 | -4.75404 | -1.64453 |
| 37 | 6 | 0 | -2.77019 | 2.855087 | -1.49432 |
| 38 | 6 | 0 | -2.61539 | 2.832887 | 0.816519 |
| 39 | 6 | 0 | -3.0715 | 4.209768 | -1.49371 |
| 40 | 6 | 0 | -2.91014 | 4.187652 | 0.885971 |
| 41 | 6 | 0 | -3.15869 | 4.91416 | -0.28608 |
| 42 | 6 | 0 | 1.70474 | 3.018311 | -0.10653 |
| 43 | 6 | 0 | 2.807998 | 2.548667 | -2.08858 |
| 44 | 6 | 0 | 1.723447 | 4.3806 | -0.37612 |
| 45 | 6 | 0 | 2.855214 | 3.89511 | -2.41935 |
| 46 | 6 | 0 | 2.311233 | 4.855143 | -1.55563 |
| 47 | 6 | 0 | 3.026683 | -2.74447 | -1.5022 |
| 48 | 6 | 0 | 2.216017 | -2.85707 | 0.662177 |
| 49 | 6 | 0 | 3.281642 | -4.10849 | -1.51282 |
| 50 | 6 | 0 | 2.44976 | -4.22503 | 0.719121 |
| 51 | 6 | 0 | 3.003092 | -4.88912 | -0.38319 |
| 52 | 7 | 0 | 2.502136 | -2.11911 | -0.42638 |
| 53 | 7 | 0 | 2.241165 | 2.110924 | -0.94344 |
| 54 | 7 | 0 | -2.20934 | -2.02911 | -0.96673 |
| 55 | 7 | 0 | -2.54402 | 2.170602 | -0.35391 |
| 56 | 1 | 0 | -6.00502 | -0.89145 | 4.486437 |
| 57 | 1 | 0 | -7.95446 | -0.9589 | 2.948862 |
| 58 | 1 | 0 | -7.6988 | -0.67392 | 0.500156 |
| 59 | 1 | 0 | -0.0154 | -0.03529 | 4.168899 |
| 60 | 1 | 0 | 6.140229 | 0.586403 | 4.523811 |
| 61 | 1 | 0 | 8.045895 | 0.577832 | 2.941216 |
| 62 | 1 | 0 | 7.71549 | 0.385148 | 0.490345 |
| 63 | 1 | 0 | -1.55345 | -2.61873 | 0.905948 |
| 64 | 1 | 0 | -2.88878 | -1.62989 | -2.87984 |
| 65 | 1 | 0 | -2.9749 | -4.0159 | -3.56588 |

| | | | | | |
|---|---|---|---|---|---|
| 66 | 1 | 0 | -1.5992 | -5.04306 | 0.387661 |
| 67 | 1 | 0 | -2.69746 | 2.284668 | -2.41168 |
| 68 | 1 | 0 | -2.44261 | 2.254579 | 1.715551 |
| 69 | 1 | 0 | -3.24973 | 4.707609 | -2.44145 |
| 70 | 1 | 0 | -2.95986 | 4.667043 | 1.858468 |
| 71 | 1 | 0 | 1.277798 | 5.066966 | 0.336691 |
| 72 | 1 | 0 | 3.324037 | 4.189407 | -3.3529 |
| 73 | 1 | 0 | 3.238456 | 1.786304 | -2.72605 |
| 74 | 1 | 0 | 1.245437 | 2.630172 | 0.794147 |
| 75 | 1 | 0 | 3.249749 | -2.11536 | -2.35475 |
| 76 | 1 | 0 | 1.786145 | -2.33148 | 1.506322 |
| 77 | 1 | 0 | 3.707792 | -4.55492 | -2.40562 |
| 78 | 1 | 0 | 2.203029 | -4.7668 | 1.626815 |
| 79 | 8 | 0 | -1.26247 | 0.396835 | -2.12039 |
| 80 | 8 | 0 | 1.289769 | -0.25407 | -2.09347 |
| 81 | 1 | 0 | -0.31384 | 0.096501 | -2.18953 |
| 82 | 6 | 0 | 2.376605 | 6.323824 | -1.8773 |
| 83 | 1 | 0 | 1.717056 | 6.908798 | -1.23215 |
| 84 | 1 | 0 | 3.397747 | 6.698485 | -1.74079 |
| 85 | 1 | 0 | 2.101727 | 6.512038 | -2.91943 |
| 86 | 6 | 0 | -3.53553 | 6.370559 | -0.24861 |
| 87 | 1 | 0 | -4.62697 | 6.476904 | -0.2571 |
| 88 | 1 | 0 | -3.16542 | 6.860093 | 0.655712 |
| 89 | 1 | 0 | -3.15115 | 6.906244 | -1.12078 |
| 90 | 6 | 0 | 3.308774 | -6.36231 | -0.35147 |
| 91 | 1 | 0 | 2.764212 | -6.87132 | 0.447419 |
| 92 | 1 | 0 | 3.06104 | -6.84013 | -1.30361 |
| 93 | 1 | 0 | 4.379491 | -6.52342 | -0.17872 |
| 94 | 6 | 0 | -2.38086 | -6.214 | -1.99794 |
| 95 | 1 | 0 | -1.74612 | -6.82391 | -1.35051 |
| 96 | 1 | 0 | -3.4115 | -6.57003 | -1.88157 |
| 97 | 1 | 0 | -2.093 | -6.38949 | -3.03807 |
| 98 | 1 | 0 | 3.127346 | 0.416936 | 4.394111 |

**Table S48.** Cartesian coordinates for $Ru^{IV}Ru^{IV}$-4pic-OH-$OH_2$-A.

| Center number | Atomic number | Atomic type | Coordinates (Ångström) | | |
|---|---|---|---|---|---|
| | | | X | Y | Z |
| 1 | 6 | 0 | 4.575507 | -0.11769 | 1.483973 |
| 2 | 6 | 0 | 4.748067 | -0.17488 | 2.877738 |
| 3 | 6 | 0 | 6.031094 | -0.24226 | 3.424485 |
| 4 | 6 | 0 | 7.091144 | -0.24946 | 2.513082 |
| 5 | 6 | 0 | 6.901582 | -0.19143 | 1.115633 |
| 6 | 6 | 0 | 5.622579 | -0.12187 | 0.56315 |
| 7 | 6 | 0 | 2.567108 | -0.07978 | 2.372215 |
| 8 | 6 | 0 | 5.386194 | -0.05497 | -0.92362 |
| 9 | 8 | 0 | 6.313838 | -0.09371 | -1.70342 |
| 10 | 8 | 0 | 4.122671 | 0.068325 | -1.37436 |
| 11 | 7 | 0 | 3.234454 | -0.06096 | 1.220831 |
| 12 | 7 | 0 | 3.446108 | -0.1471 | 3.410237 |
| 13 | 6 | 0 | 0.028869 | 0.004205 | 3.104457 |

| | | | | | |
|---|---|---|---|---|---|
| 14 | 6 | 0 | -1.06951 | 0.045061 | 2.241597 |
| 15 | 7 | 0 | 0.695132 | -0.01117 | 0.934088 |
| 16 | 7 | 0 | -0.63835 | 0.030005 | 0.930983 |
| 17 | 6 | 0 | 1.128394 | -0.03279 | 2.244144 |
| 18 | 6 | 0 | -4.52485 | 0.094837 | 1.521734 |
| 19 | 6 | 0 | -4.68698 | 0.138161 | 2.918669 |
| 20 | 6 | 0 | -5.96788 | 0.176584 | 3.474915 |
| 21 | 6 | 0 | -7.03132 | 0.166344 | 2.56856 |
| 22 | 6 | 0 | -6.84992 | 0.117671 | 1.168139 |
| 23 | 6 | 0 | -5.57608 | 0.079084 | 0.603408 |
| 24 | 6 | 0 | -2.51249 | 0.082977 | 2.384462 |
| 25 | 7 | 0 | -3.18923 | 0.065337 | 1.245262 |
| 26 | 7 | 0 | -3.38086 | 0.127944 | 3.438608 |
| 27 | 6 | 0 | -5.35925 | 0.018355 | -0.89441 |
| 28 | 8 | 0 | -6.3218 | 0.015683 | -1.63697 |
| 29 | 8 | 0 | -4.11649 | -0.03756 | -1.39065 |
| 30 | 44 | 0 | -2.25871 | -0.01897 | -0.59629 |
| 31 | 44 | 0 | 2.284881 | 0.034152 | -0.584 |
| 32 | 6 | 0 | -2.86169 | -2.70624 | -1.76435 |
| 33 | 6 | 0 | -2.23477 | -2.9686 | 0.449217 |
| 34 | 6 | 0 | -3.10445 | -4.06631 | -1.89041 |
| 35 | 6 | 0 | -2.4621 | -4.3365 | 0.391675 |
| 36 | 6 | 0 | -2.91797 | -4.92394 | -0.79745 |
| 37 | 6 | 0 | -2.86216 | 2.574518 | -1.9616 |
| 38 | 6 | 0 | -2.05804 | 2.999221 | 0.168999 |
| 39 | 6 | 0 | -3.04875 | 3.928853 | -2.19602 |
| 40 | 6 | 0 | -2.22546 | 4.366688 | 0.000528 |
| 41 | 6 | 0 | -2.73986 | 4.86926 | -1.20336 |
| 42 | 6 | 0 | 2.876914 | 2.73184 | -1.73239 |
| 43 | 6 | 0 | 2.176207 | 2.973446 | 0.464587 |
| 44 | 6 | 0 | 3.075244 | 4.098768 | -1.85313 |
| 45 | 6 | 0 | 2.359132 | 4.347577 | 0.410383 |
| 46 | 6 | 0 | 2.827305 | 4.950072 | -0.76666 |
| 47 | 6 | 0 | 2.873269 | -2.56372 | -1.94635 |
| 48 | 6 | 0 | 2.009705 | -2.97382 | 0.167387 |
| 49 | 6 | 0 | 3.018366 | -3.92133 | -2.18554 |
| 50 | 6 | 0 | 2.135061 | -4.34429 | -0.00827 |
| 51 | 6 | 0 | 2.65626 | -4.85687 | -1.20533 |
| 52 | 7 | 0 | 2.377718 | -2.08938 | -0.78061 |
| 53 | 7 | 0 | 2.434362 | 2.169339 | -0.58539 |
| 54 | 7 | 0 | -2.4326 | -2.15658 | -0.60676 |
| 55 | 7 | 0 | -2.37407 | 2.108552 | -0.79045 |
| 56 | 1 | 0 | -6.14222 | 0.211545 | 4.544836 |
| 57 | 1 | 0 | -8.04325 | 0.195093 | 2.958975 |
| 58 | 1 | 0 | -7.708 | 0.108057 | 0.504684 |
| 59 | 1 | 0 | 0.027958 | 0.000947 | 4.184455 |
| 60 | 1 | 0 | 6.210114 | -0.28722 | 4.493262 |
| 61 | 1 | 0 | 8.104153 | -0.30153 | 2.897846 |
| 62 | 1 | 0 | 7.754846 | -0.19927 | 0.44591 |
| 63 | 1 | 0 | -1.89057 | -2.49933 | 1.363213 |
| 64 | 1 | 0 | -3.01926 | -2.02224 | -2.58864 |
| 65 | 1 | 0 | -3.45242 | -4.44976 | -2.84412 |
| 66 | 1 | 0 | -2.29417 | -4.94005 | 1.278033 |
| 67 | 1 | 0 | -3.11667 | 1.828636 | -2.70396 |
| 68 | 1 | 0 | -1.66316 | 2.594345 | 1.093267 |

| | | | | | |
|---|---|---|---|---|---|
| 69 | 1 | 0 | -3.44753 | 4.243881 | -3.15487 |
| 70 | 1 | 0 | -1.96214 | 5.037398 | 0.812121 |
| 71 | 1 | 0 | 3.433521 | 4.493824 | -2.7982 |
| 72 | 1 | 0 | 2.142255 | 4.945654 | 1.289584 |
| 73 | 1 | 0 | 1.815709 | 2.494301 | 1.366745 |
| 74 | 1 | 0 | 3.077835 | 2.055343 | -2.55339 |
| 75 | 1 | 0 | 3.162841 | -1.82381 | -2.68184 |
| 76 | 1 | 0 | 1.604258 | -2.56194 | 1.083502 |
| 77 | 1 | 0 | 3.423442 | -4.24388 | -3.13919 |
| 78 | 1 | 0 | 1.829283 | -5.01059 | 0.791727 |
| 79 | 8 | 0 | -1.2218 | -0.09912 | -2.07286 |
| 80 | 6 | 0 | -3.22137 | -6.39371 | -0.89132 |
| 81 | 1 | 0 | -2.71812 | -6.96309 | -0.10644 |
| 82 | 1 | 0 | -4.29957 | -6.5622 | -0.78317 |
| 83 | 1 | 0 | -2.92915 | -6.79943 | -1.86368 |
| 84 | 6 | 0 | -2.97523 | 6.339141 | -1.41498 |
| 85 | 1 | 0 | -4.04195 | 6.569162 | -1.30747 |
| 86 | 1 | 0 | -2.43024 | 6.9463 | -0.68867 |
| 87 | 1 | 0 | -2.68118 | 6.647272 | -2.42228 |
| 88 | 6 | 0 | 3.07639 | 6.429541 | -0.85695 |
| 89 | 1 | 0 | 4.149122 | 6.636315 | -0.76142 |
| 90 | 1 | 0 | 2.758571 | 6.827155 | -1.82479 |
| 91 | 1 | 0 | 2.561323 | 6.977312 | -0.06475 |
| 92 | 6 | 0 | 2.840221 | -6.33211 | -1.42546 |
| 93 | 1 | 0 | 3.901281 | -6.59592 | -1.34251 |
| 94 | 1 | 0 | 2.290866 | -6.92426 | -0.69052 |
| 95 | 1 | 0 | 2.515301 | -6.62562 | -2.42782 |
| 96 | 8 | 0 | 1.243603 | 0.121343 | -2.11163 |
| 97 | 1 | 0 | 0.196789 | 0.022702 | -2.12749 |
| 98 | 1 | 0 | -3.13342 | 0.155805 | 4.417192 |
| 99 | 1 | 0 | 3.206404 | -0.17855 | 4.391119 |

**Table S49.** Cartesian coordinates for Ru$^{IV}$Ru$^{V}$-4pic-2O-A.

| Center number | Atomic number | Atomic type | Coordinates (Ångström) | | |
|---|---|---|---|---|---|
| | | | X | Y | Z |
| 1 | 6 | 0 | -4.3195 | -1.03171 | 1.532062 |
| 2 | 6 | 0 | -4.37529 | -1.04708 | 2.953885 |
| 3 | 6 | 0 | -5.61903 | -1.29083 | 3.554755 |
| 4 | 6 | 0 | -6.72144 | -1.48508 | 2.71561 |
| 5 | 6 | 0 | -6.6296 | -1.43259 | 1.307606 |
| 6 | 6 | 0 | -5.40779 | -1.19601 | 0.671829 |
| 7 | 6 | 0 | -2.36648 | -0.62624 | 2.374081 |
| 8 | 6 | 0 | -5.28002 | -1.07952 | -0.82845 |
| 9 | 8 | 0 | -6.27161 | -1.23791 | -1.53206 |
| 10 | 8 | 0 | -4.10112 | -0.79872 | -1.38646 |
| 11 | 7 | 0 | -3.03085 | -0.79086 | 1.195598 |
| 12 | 7 | 0 | -3.10921 | -0.77876 | 3.469122 |
| 13 | 6 | 0 | 0.090346 | -0.01334 | 3.054178 |
| 14 | 6 | 0 | 1.166255 | 0.269421 | 2.220555 |
| 15 | 7 | 0 | -0.55874 | -0.11583 | 0.875238 |

| | | | | | |
|---|---|---|---|---|---|
| 16 | 7 | 0 | 0.758922 | 0.166437 | 0.90901 |
| 17 | 6 | 0 | -0.97192 | -0.25885 | 2.180405 |
| 18 | 6 | 0 | 4.476436 | 0.960982 | 1.406814 |
| 19 | 6 | 0 | 4.636274 | 0.868246 | 2.815189 |
| 20 | 6 | 0 | 5.929454 | 0.984764 | 3.33179 |
| 21 | 6 | 0 | 6.991038 | 1.14891 | 2.426462 |
| 22 | 6 | 0 | 6.803688 | 1.18933 | 1.033836 |
| 23 | 6 | 0 | 5.516853 | 1.09103 | 0.486743 |
| 24 | 6 | 0 | 2.561491 | 0.615326 | 2.381401 |
| 25 | 7 | 0 | 3.146718 | 0.857728 | 1.157673 |
| 26 | 7 | 0 | 3.39358 | 0.636451 | 3.409625 |
| 27 | 6 | 0 | 5.240614 | 1.063886 | -0.97672 |
| 28 | 8 | 0 | 6.115607 | 1.030548 | -1.82238 |
| 29 | 8 | 0 | 3.936959 | 1.144735 | -1.35852 |
| 30 | 44 | 0 | 2.242792 | 0.434315 | -0.62066 |
| 31 | 44 | 0 | -2.24314 | -0.34697 | -0.63178 |
| 32 | 6 | 0 | 0.380865 | 2.590045 | -1.67085 |
| 33 | 6 | 0 | 2.191942 | 3.460001 | -0.49679 |
| 34 | 6 | 0 | -0.06673 | 3.872045 | -1.97001 |
| 35 | 6 | 0 | 1.788255 | 4.759721 | -0.76169 |
| 36 | 6 | 0 | 0.636142 | 4.995702 | -1.52321 |
| 37 | 6 | 0 | 3.948702 | -1.96407 | -1.37485 |
| 38 | 6 | 0 | 2.868886 | -2.34827 | 0.638707 |
| 39 | 6 | 0 | 4.544475 | -3.217 | -1.33534 |
| 40 | 6 | 0 | 3.432368 | -3.61368 | 0.738026 |
| 41 | 6 | 0 | 4.299522 | -4.07795 | -0.2581 |
| 42 | 6 | 0 | -2.04982 | -3.3851 | -0.22842 |
| 43 | 6 | 0 | -1.00536 | -2.65539 | -2.16047 |
| 44 | 6 | 0 | -1.76739 | -4.71088 | -0.53182 |
| 45 | 6 | 0 | -0.69072 | -3.96016 | -2.52015 |
| 46 | 6 | 0 | -1.07574 | -5.0312 | -1.70545 |
| 47 | 6 | 0 | -3.71654 | 2.145623 | -1.39742 |
| 48 | 6 | 0 | -2.83398 | 2.394275 | 0.727624 |
| 49 | 6 | 0 | -4.30146 | 3.404517 | -1.33762 |
| 50 | 6 | 0 | -3.39359 | 3.659938 | 0.853145 |
| 51 | 6 | 0 | -4.15607 | 4.195655 | -0.19149 |
| 52 | 7 | 0 | -2.98917 | 1.644731 | -0.37832 |
| 53 | 7 | 0 | -1.6753 | -2.3665 | -1.02688 |
| 54 | 7 | 0 | 1.500195 | 2.392473 | -0.94448 |
| 55 | 7 | 0 | 3.127102 | -1.53027 | -0.39801 |
| 56 | 1 | 0 | 6.111564 | 0.936026 | 4.400944 |
| 57 | 1 | 0 | 8.001174 | 1.230325 | 2.816876 |
| 58 | 1 | 0 | 7.651516 | 1.284377 | 0.363644 |
| 59 | 1 | 0 | 0.073964 | -0.03238 | 4.132434 |
| 60 | 1 | 0 | -5.72543 | -1.31919 | 4.63525 |
| 61 | 1 | 0 | -7.69335 | -1.67246 | 3.164071 |
| 62 | 1 | 0 | -7.51251 | -1.56614 | 0.691134 |
| 63 | 1 | 0 | 3.081065 | 3.249462 | 0.082875 |
| 64 | 1 | 0 | -0.16163 | 1.703523 | -1.99896 |
| 65 | 1 | 0 | -0.97444 | 3.978112 | -2.55531 |
| 66 | 1 | 0 | 2.380288 | 5.583013 | -0.3748 |
| 67 | 1 | 0 | 4.129895 | -1.2769 | -2.19311 |
| 68 | 1 | 0 | 2.191357 | -1.97499 | 1.396274 |
| 69 | 1 | 0 | 5.202871 | -3.51176 | -2.14612 |
| 70 | 1 | 0 | 3.18966 | -4.23111 | 1.596831 |

| | | | | | |
|---|---|---|---|---|---|
| 71 | 1 | 0 | -2.10037 | -5.48825 | 0.148972 |
| 72 | 1 | 0 | -0.15073 | -4.13285 | -3.44618 |
| 73 | 1 | 0 | -0.73095 | -1.79929 | -2.76532 |
| 74 | 1 | 0 | -2.59347 | -3.11144 | 0.667149 |
| 75 | 1 | 0 | -3.83063 | 1.494903 | -2.25593 |
| 76 | 1 | 0 | -2.24925 | 1.958989 | 1.52824 |
| 77 | 1 | 0 | -4.8846 | 3.754206 | -2.18398 |
| 78 | 1 | 0 | -3.23787 | 4.217069 | 1.771731 |
| 79 | 8 | 0 | 1.259758 | -0.31168 | -1.83826 |
| 80 | 8 | 0 | -1.48321 | 0.198186 | -2.17721 |
| 81 | 6 | 0 | -0.79268 | -6.46044 | -2.08905 |
| 82 | 1 | 0 | -0.65178 | -7.09179 | -1.20707 |
| 83 | 1 | 0 | -1.63333 | -6.87675 | -2.65724 |
| 84 | 1 | 0 | 0.097912 | -6.53795 | -2.71885 |
| 85 | 6 | 0 | 4.958719 | -5.42813 | -0.16439 |
| 86 | 1 | 0 | 5.950208 | -5.33447 | 0.294913 |
| 87 | 1 | 0 | 4.374764 | -6.11736 | 0.450844 |
| 88 | 1 | 0 | 5.098303 | -5.87384 | -1.15306 |
| 89 | 6 | 0 | 0.193322 | 6.39376 | -1.86466 |
| 90 | 1 | 0 | 0.433548 | 7.096682 | -1.06209 |
| 91 | 1 | 0 | 0.70412 | 6.743603 | -2.76995 |
| 92 | 1 | 0 | -0.8818 | 6.436389 | -2.05668 |
| 93 | 6 | 0 | -4.81932 | 5.543192 | -0.07369 |
| 94 | 1 | 0 | -5.82761 | 5.435337 | 0.344031 |
| 95 | 1 | 0 | -4.26061 | 6.209322 | 0.589625 |
| 96 | 1 | 0 | -4.92148 | 6.026543 | -1.04935 |

**Table S50.** Cartesian coordinates for $Ru^{IV}Ru^{V}$-4pic-O-OH-A.

| Center number | Atomic number | Atomic type | Coordinates (Ångström) | | |
|---|---|---|---|---|---|
| | | | X | Y | Z |
| 1 | 6 | 0 | 4.378426 | -1.00609 | 1.57428 |
| 2 | 6 | 0 | 4.476988 | -1.12416 | 2.97391 |
| 3 | 6 | 0 | 5.702552 | -1.42968 | 3.567362 |
| 4 | 6 | 0 | 6.785143 | -1.59343 | 2.697216 |
| 5 | 6 | 0 | 6.67211 | -1.45265 | 1.29811 |
| 6 | 6 | 0 | 5.452223 | -1.14657 | 0.693943 |
| 7 | 6 | 0 | 2.38211 | -0.6153 | 2.369304 |
| 8 | 6 | 0 | 5.325922 | -0.95157 | -0.80707 |
| 9 | 8 | 0 | 6.317459 | -1.12502 | -1.49938 |
| 10 | 8 | 0 | 4.170171 | -0.58027 | -1.34615 |
| 11 | 7 | 0 | 3.085144 | -0.71215 | 1.254858 |
| 12 | 7 | 0 | 3.18159 | -0.86577 | 3.451996 |
| 13 | 6 | 0 | -0.07622 | -0.02466 | 3.080945 |
| 14 | 6 | 0 | -1.14599 | 0.257407 | 2.236203 |
| 15 | 7 | 0 | 0.581118 | -0.12251 | 0.897401 |
| 16 | 7 | 0 | -0.7297 | 0.164527 | 0.931935 |
| 17 | 6 | 0 | 0.98783 | -0.26385 | 2.207564 |
| 18 | 6 | 0 | -4.48146 | 0.820319 | 1.422935 |
| 19 | 6 | 0 | -4.63579 | 0.7211 | 2.829308 |
| 20 | 6 | 0 | -5.93304 | 0.769133 | 3.350033 |

| | | | | | |
|---|---|---|---|---|---|
| 21 | 6 | 0 | -7.00108 | 0.867386 | 2.445199 |
| 22 | 6 | 0 | -6.81691 | 0.90493 | 1.050767 |
| 23 | 6 | 0 | -5.52821 | 0.877314 | 0.501942 |
| 24 | 6 | 0 | -2.55231 | 0.566403 | 2.394557 |
| 25 | 7 | 0 | -3.14567 | 0.780986 | 1.171826 |
| 26 | 7 | 0 | -3.38124 | 0.545661 | 3.422614 |
| 27 | 6 | 0 | -5.25512 | 0.831461 | -0.96164 |
| 28 | 8 | 0 | -6.11359 | 0.711572 | -1.80842 |
| 29 | 8 | 0 | -3.94879 | 1.017158 | -1.34113 |
| 30 | 44 | 0 | -2.25612 | 0.370529 | -0.61176 |
| 31 | 44 | 0 | 2.283456 | -0.2318 | -0.60861 |
| 32 | 6 | 0 | -3.63627 | -2.14542 | -1.54646 |
| 33 | 6 | 0 | -3.20962 | -2.31243 | 0.724319 |
| 34 | 6 | 0 | -4.25657 | -3.38608 | -1.55751 |
| 35 | 6 | 0 | -3.81297 | -3.5614 | 0.780236 |
| 36 | 6 | 0 | -4.3666 | -4.12931 | -0.37471 |
| 37 | 6 | 0 | -0.57487 | 2.582297 | -1.84437 |
| 38 | 6 | 0 | -2.27275 | 3.404242 | -0.48337 |
| 39 | 6 | 0 | -0.2279 | 3.871152 | -2.2298 |
| 40 | 6 | 0 | -1.96594 | 4.711704 | -0.82826 |
| 41 | 6 | 0 | -0.92864 | 4.976671 | -1.73296 |
| 42 | 6 | 0 | 2.456856 | 2.601993 | 0.711522 |
| 43 | 6 | 0 | 3.787046 | 2.324579 | -1.16043 |
| 44 | 6 | 0 | 2.884427 | 3.913935 | 0.86907 |
| 45 | 6 | 0 | 4.252538 | 3.629404 | -1.06242 |
| 46 | 6 | 0 | 3.812053 | 4.462488 | -0.02624 |
| 47 | 6 | 0 | 2.257196 | -2.69877 | -2.29142 |
| 48 | 6 | 0 | 1.300968 | -3.14243 | -0.23019 |
| 49 | 6 | 0 | 2.071468 | -4.01257 | -2.69694 |
| 50 | 6 | 0 | 1.089858 | -4.47199 | -0.57203 |
| 51 | 6 | 0 | 1.480632 | -4.94286 | -1.83198 |
| 52 | 7 | 0 | 1.877683 | -2.26469 | -1.07096 |
| 53 | 7 | 0 | 2.90192 | 1.808102 | -0.28113 |
| 54 | 7 | 0 | -3.12331 | -1.60771 | -0.41962 |
| 55 | 7 | 0 | -1.5884 | 2.353949 | -0.98258 |
| 56 | 1 | 0 | -6.11093 | 0.717869 | 4.419442 |
| 57 | 1 | 0 | -8.01382 | 0.895527 | 2.834778 |
| 58 | 1 | 0 | -7.67101 | 0.941198 | 0.382879 |
| 59 | 1 | 0 | -0.08972 | -0.04305 | 4.16014 |
| 60 | 1 | 0 | 5.823653 | -1.53272 | 4.640513 |
| 61 | 1 | 0 | 7.756304 | -1.83149 | 3.119158 |
| 62 | 1 | 0 | 7.541168 | -1.57421 | 0.660752 |
| 63 | 1 | 0 | -2.78875 | -1.85844 | 1.612366 |
| 64 | 1 | 0 | -3.54638 | -1.55304 | -2.44906 |
| 65 | 1 | 0 | -4.65943 | -3.76292 | -2.49192 |
| 66 | 1 | 0 | -3.85712 | -4.08205 | 1.731415 |
| 67 | 1 | 0 | -0.03776 | 1.712812 | -2.21145 |
| 68 | 1 | 0 | -3.08012 | 3.173014 | 0.199514 |
| 69 | 1 | 0 | 0.590322 | 3.999151 | -2.93115 |
| 70 | 1 | 0 | -2.54974 | 5.518979 | -0.3979 |
| 71 | 1 | 0 | 2.489366 | 4.503743 | 1.690302 |
| 72 | 1 | 0 | 4.966914 | 3.987052 | -1.79729 |
| 73 | 1 | 0 | 4.125655 | 1.652362 | -1.93985 |
| 74 | 1 | 0 | 1.730411 | 2.168019 | 1.387413 |
| 75 | 1 | 0 | 2.722645 | -1.96116 | -2.93286 |

| | | | | | |
|---|---|---|---|---|---|
| 76 | 1 | 0 | 1.001012 | -2.75698 | 0.736537 |
| 77 | 1 | 0 | 2.396784 | -4.30573 | -3.68997 |
| 78 | 1 | 0 | 0.623364 | -5.13652 | 0.148258 |
| 79 | 8 | 0 | -1.20864 | -0.37083 | -1.77157 |
| 80 | 8 | 0 | 1.554653 | 0.288334 | -2.15564 |
| 81 | 6 | 0 | 4.329099 | 5.867305 | 0.13044 |
| 82 | 1 | 0 | 3.621919 | 6.498746 | 0.674282 |
| 83 | 1 | 0 | 5.269467 | 5.864364 | 0.694731 |
| 84 | 1 | 0 | 4.535912 | 6.327455 | -0.83954 |
| 85 | 6 | 0 | -0.60891 | 6.38005 | -2.1716 |
| 86 | 1 | 0 | -1.24916 | 6.665646 | -3.01494 |
| 87 | 1 | 0 | -0.78741 | 7.101273 | -1.36948 |
| 88 | 1 | 0 | 0.428495 | 6.471266 | -2.50274 |
| 89 | 6 | 0 | -5.07127 | -5.4579 | -0.34341 |
| 90 | 1 | 0 | -4.72997 | -6.07546 | 0.490963 |
| 91 | 1 | 0 | -6.15094 | -5.30774 | -0.22313 |
| 92 | 1 | 0 | -4.92231 | -6.01108 | -1.2745 |
| 93 | 6 | 0 | 1.298832 | -6.38184 | -2.23443 |
| 94 | 1 | 0 | 2.256493 | -6.91369 | -2.18939 |
| 95 | 1 | 0 | 0.598847 | -6.90056 | -1.57507 |
| 96 | 1 | 0 | 0.934003 | -6.46205 | -3.26263 |
| 97 | 1 | 0 | 2.890063 | -0.86584 | 4.41795 |

**Table S51.** Cartesian coordinates for Ru$^{IV}$Ru$^{V}$-4pic-2OH-A.

| Center number | Atomic number | Atomic type | Coordinates (Ångström) | | |
|---|---|---|---|---|---|
| | | | **X** | **Y** | **Z** |
| 1 | 6 | 0 | 4.52253 | -0.00064 | 1.519101 |
| 2 | 6 | 0 | 4.689922 | -0.00071 | 2.918776 |
| 3 | 6 | 0 | 5.972933 | -0.00076 | 3.470408 |
| 4 | 6 | 0 | 7.032033 | -0.00073 | 2.558731 |
| 5 | 6 | 0 | 6.845847 | -0.00064 | 1.156205 |
| 6 | 6 | 0 | 5.571202 | -0.00061 | 0.595557 |
| 7 | 6 | 0 | 2.515414 | -0.00054 | 2.390628 |
| 8 | 6 | 0 | 5.347212 | -0.00051 | -0.90228 |
| 9 | 8 | 0 | 6.30621 | -0.00017 | -1.64862 |
| 10 | 8 | 0 | 4.100943 | -0.00094 | -1.39425 |
| 11 | 7 | 0 | 3.189375 | -0.00053 | 1.246541 |
| 12 | 7 | 0 | 3.387433 | -0.00061 | 3.442418 |
| 13 | 6 | 0 | -0.0164 | -0.00019 | 3.10825 |
| 14 | 6 | 0 | -1.12249 | 0.000037 | 2.247546 |
| 15 | 7 | 0 | 0.639372 | -0.00024 | 0.923547 |
| 16 | 7 | 0 | -0.6792 | 0.00003 | 0.924223 |
| 17 | 6 | 0 | 1.076992 | -0.00035 | 2.254634 |
| 18 | 6 | 0 | -4.51001 | 0.000614 | 1.50215 |
| 19 | 6 | 0 | -4.59899 | 0.000364 | 2.951863 |
| 20 | 6 | 0 | -5.89727 | 0.000286 | 3.536537 |
| 21 | 6 | 0 | -6.97472 | 0.000379 | 2.674647 |
| 22 | 6 | 0 | -6.83584 | 0.000533 | 1.241788 |
| 23 | 6 | 0 | -5.58425 | 0.000678 | 0.624053 |
| 24 | 6 | 0 | -2.53907 | 0.000293 | 2.398401 |

| | | | | | |
|---|---|---|---|---|---|
| 25 | 7 | 0 | -3.19874 | 0.000585 | 1.20315 |
| 26 | 7 | 0 | -3.3566 | 0.00017 | 3.486452 |
| 27 | 6 | 0 | -5.3941 | 0.000783 | -0.8721 |
| 28 | 8 | 0 | -6.35203 | 0.000442 | -1.6157 |
| 29 | 8 | 0 | -4.14034 | 0.00145 | -1.35727 |
| 30 | 44 | 0 | -2.28639 | 0.000666 | -0.59128 |
| 31 | 44 | 0 | 2.246726 | -0.00061 | -0.59229 |
| 32 | 6 | 0 | -2.79492 | -2.64863 | -1.88362 |
| 33 | 6 | 0 | -2.20739 | -2.97671 | 0.336144 |
| 34 | 6 | 0 | -2.99117 | -4.00896 | -2.06602 |
| 35 | 6 | 0 | -2.39015 | -4.34689 | 0.219583 |
| 36 | 6 | 0 | -2.79967 | -4.90213 | -1.00186 |
| 37 | 6 | 0 | -2.79462 | 2.650696 | -1.88217 |
| 38 | 6 | 0 | -2.20402 | 2.977679 | 0.336947 |
| 39 | 6 | 0 | -2.98993 | 4.011256 | -2.06396 |
| 40 | 6 | 0 | -2.38577 | 4.348035 | 0.220989 |
| 41 | 6 | 0 | -2.79635 | 4.903959 | -0.9998 |
| 42 | 6 | 0 | 2.10534 | 2.989188 | 0.311234 |
| 43 | 6 | 0 | 2.865216 | 2.645237 | -1.84998 |
| 44 | 6 | 0 | 2.293183 | 4.359554 | 0.199973 |
| 45 | 6 | 0 | 3.071624 | 4.005394 | -2.02784 |
| 46 | 6 | 0 | 2.796385 | 4.906298 | -0.98964 |
| 47 | 6 | 0 | 2.86584 | -2.64704 | -1.84851 |
| 48 | 6 | 0 | 2.102477 | -2.99017 | 0.311609 |
| 49 | 6 | 0 | 3.071447 | -4.00739 | -2.02591 |
| 50 | 6 | 0 | 2.289457 | -4.36068 | 0.200788 |
| 51 | 6 | 0 | 2.794004 | -4.90796 | -0.98803 |
| 52 | 7 | 0 | 2.388685 | -2.13711 | -0.69117 |
| 53 | 7 | 0 | 2.389365 | 2.135802 | -0.69188 |
| 54 | 7 | 0 | -2.40805 | -2.13237 | -0.6954 |
| 55 | 7 | 0 | -2.40673 | 2.133788 | -0.69457 |
| 56 | 1 | 0 | -6.0249 | 0.000139 | 4.613291 |
| 57 | 1 | 0 | -7.98023 | 0.000313 | 3.082075 |
| 58 | 1 | 0 | -7.72284 | 0.000528 | 0.615385 |
| 59 | 1 | 0 | -0.03885 | -0.00024 | 4.187751 |
| 60 | 1 | 0 | 6.152288 | -0.00081 | 4.539963 |
| 61 | 1 | 0 | 8.045847 | -0.00077 | 2.945365 |
| 62 | 1 | 0 | 7.702481 | -0.0006 | 0.490787 |
| 63 | 1 | 0 | -1.89945 | -2.53418 | 1.275573 |
| 64 | 1 | 0 | -2.95018 | -1.9406 | -2.68791 |
| 65 | 1 | 0 | -3.30374 | -4.36607 | -3.0419 |
| 66 | 1 | 0 | -2.22131 | -4.97798 | 1.086099 |
| 67 | 1 | 0 | -2.95156 | 1.943016 | -2.68644 |
| 68 | 1 | 0 | -1.8952 | 2.534632 | 1.275841 |
| 69 | 1 | 0 | -3.30338 | 4.368903 | -3.03936 |
| 70 | 1 | 0 | -2.21529 | 4.978754 | 1.087457 |
| 71 | 1 | 0 | 2.054303 | 4.997645 | 1.044762 |
| 72 | 1 | 0 | 3.459593 | 4.35554 | -2.9789 |
| 73 | 1 | 0 | 3.092826 | 1.92928 | -2.62958 |
| 74 | 1 | 0 | 1.720344 | 2.55307 | 1.225465 |
| 75 | 1 | 0 | 3.095222 | -1.93134 | -2.62783 |
| 76 | 1 | 0 | 1.716373 | -2.55367 | 1.225189 |
| 77 | 1 | 0 | 3.460513 | -4.35794 | -2.97638 |
| 78 | 1 | 0 | 2.048817 | -4.9985 | 1.045288 |
| 79 | 8 | 0 | -1.265 | 0.000639 | -2.14389 |

| | | | | | |
|---|---|---|---|---|---|
| 80 | 8 | 0 | 1.227735 | -0.00066 | -2.0772 |
| 81 | 1 | 0 | -0.22853 | 0.000061 | -2.16315 |
| 82 | 6 | 0 | 3.055252 | 6.379687 | -1.13906 |
| 83 | 1 | 0 | 2.504772 | 6.965573 | -0.3995 |
| 84 | 1 | 0 | 4.122712 | 6.589498 | -1.00083 |
| 85 | 1 | 0 | 2.786198 | 6.731532 | -2.13879 |
| 86 | 6 | 0 | -3.04431 | 6.377928 | -1.15701 |
| 87 | 1 | 0 | -4.11947 | 6.585918 | -1.09709 |
| 88 | 1 | 0 | -2.55119 | 6.958132 | -0.37396 |
| 89 | 1 | 0 | -2.70201 | 6.73709 | -2.13139 |
| 90 | 6 | 0 | 3.052026 | -6.38155 | -1.13681 |
| 91 | 1 | 0 | 2.49826 | -6.96707 | -0.3994 |
| 92 | 1 | 0 | 2.786391 | -6.73296 | -2.1376 |
| 93 | 1 | 0 | 4.118763 | -6.59239 | -0.99457 |
| 94 | 6 | 0 | -3.04865 | -6.37585 | -1.15982 |
| 95 | 1 | 0 | -2.55782 | -6.95662 | -0.37576 |
| 96 | 1 | 0 | -4.12413 | -6.58285 | -1.1024 |
| 97 | 1 | 0 | -2.70447 | -6.73518 | -2.13348 |
| 98 | 1 | 0 | 3.141778 | -0.00081 | 4.42244 |
